# PERK/ATF4-Dependent ZFAS1 Upregulation Is Associated with Sorafenib Resistance in Hepatocellular Carcinoma Cells

**DOI:** 10.3390/ijms22115848

**Published:** 2021-05-29

**Authors:** Jiunn-Chang Lin, Pei-Ming Yang, Tsang-Pai Liu

**Affiliations:** 1 Department of Surgery, MacKay Memorial Hospital, Taipei 10449, Taiwan; steven4375@gmail.com; 2 MacKay Junior College of Medicine, Nursing, and Management, New Taipei City 11260, Taiwan; 3 Department of Medicine, MacKay Medical College, New Taipei City 25245, Taiwan; 4 Liver Medical Center, MacKay Memorial Hospital, Taipei 10449, Taiwan; 5 PhD Program for Cancer Molecular Biology and Drug Discovery, College of Medical Science and Technology, Taipei Medical University and Academia Sinica, Taipei 11031, Taiwan; 6 Graduate Institute of Cancer Biology and Drug Discovery, College of Medical Science and Technology, Taipei Medical University, Taipei 11031, Taiwan; 7 TMU Research Center of Cancer Translational Medicine, Taipei 11031, Taiwan; 8 Cancer Center, Wan Fang Hospital, Taipei Medical University, Taipei 11696, Taiwan

**Keywords:** drug resistance, ER stress, hepatocellular carcinoma, long non-coding RNA, sorafenib, unfolded protein response

## Abstract

Sorafenib, a multi-kinase inhibitor, is the first-line treatment for advanced hepatocellular carcinoma (HCC) patients. However, this drug only provides a short improvement of patients’ overall survival, and drug resistance is commonly developed. Thus, the identification of resistant factor(s) or biomarker(s) is needed to develop more efficient therapeutic strategies. Long, non-coding RNAs (lncRNAs) have recently been viewed as attractive cancer biomarkers and drive many important cancer phenotypes. A lncRNA, *ZFAS1* (ZNFX1 antisense RNA 1) has been found to promote HCC metastasis. This study found that sorafenib induced *ZFAS1* expression specifically in sorafenib-resistant HCC cells. Although *ZFAS1* knockdown did not restore the sensitivity of HCC cells to sorafenib, its expression may act as a resistant biomarker for sorafenib therapy. Bioinformatics analysis predicted that sorafenib tended to induce pathways related to endoplasmic reticulum (ER) stress and the unfolded protein response (UPR) in sorafenib-resistant HCC cells. In vitro experimental evidence suggested that sorafenib induced protein kinase RNA-like ER kinase (PERK)/activating transcription factor 4 (ATF4)-dependent *ZFAS1* expression, and sorafenib resistance could be overcome by PERK/ATF inhibitors. Therefore, PERK/ATF4/*ZFAS1* signaling axis might be an attractive therapeutic and prognostic biomarker for sorafenib therapy in HCC.

## 1. Introduction

Multi-kinase inhibitors have been approved for treating hepatocellular carcinoma (HCC) in recent years, including sorafenib, regorafenib, lenvatinib, and cabozantinib [1,2,3,4]. However, they only provide a short improvement of HCC patients’ overall survival [1,2,3,4]. Therefore, it is urgently needed to identify sensitive and/or resistant factors to develop more effective therapeutic protocols. Sorafenib has been approved for treating advanced HCC since 2007. However, it only benefits about 30% of patients, and acquired resistance usually develops within six months [1,5,6]. Although the clinical inefficiency of sorafenib has raised concerns by researchers, the drug resistance mechanism is still elusive [5]. A recent review article summarizes the potential resistant factors, including activation of EGFR (epidermal growth factor receptor), AKT and c-Jun oncogenic signaling pathways, autophagy, epithelial–mesenchymal transition (EMT), hypoxia, cancer stemness, dysregulation of the cell cycle, and apoptosis resistance. Drugs targeting these resistant factors might be able to improve the sorafenib efficacy or treat sorafenib-refractory HCC patients [7].

The accumulation of unfolded or misfolded proteins within the endoplasmic reticulum (ER), the site for protein synthesis and folding, activates the unfolded protein response (UPR) through three signal arms: PERK (protein kinase RNA-like ER kinase), IRE1 (serine/threonine kinases inositol-requiring enzyme-1), and ATF6 (activating transcription factor 6) [8,9]. The activation of PERK to phosphorylate eIF2α (eukaryotic translation initiation factor alpha) is an immediate response of ER stress/UPR, which attenuates global translation, but selectively induces translation of certain mRNAs, such as *ATF4* and *CHOP* (CCAAT/enhancer binding protein (C/EBP) homologous protein) [10]. ATF4 encodes a basic zipper (bZIP) transcriptional activator for genes associated with protein folding and assembly. It also promotes the expression of *CHOP* (also a bZIP transcription factor) that directs cell fate to apoptosis [10]. Glucose-regulated protein 78 (GRP78) is also a central modulator for ER stress/UPR by acting as an ER chaperone and controlling the activation of IRE1, PERK, and ATF6 through a binding-release mechanism [11].

Up to 70% of the human genomic DNA sequence are transcribed into RNA with no protein-coding potential. These RNA molecules are named noncoding RNAs (ncRNAs). According to their lengths, ncRNAs are classified as small ncRNAs (such as microRNAs (miRNAs) with 18–25 nucleotides) and long ncRNAs (lncRNAs with more than 200 nucleotides). In recent years, lncRNAs have been found to participate in various biological processes and human disease pathogenesis through binding with macromolecules, including DNA, chromatin, RNA, signaling and regulatory proteins [12]. LncRNAs have been linked to sorafenib resistance. For example, knockdown of lncRNA *TUC338* (transcribed ultra-conserved region 338) enhances the anticancer activity of sorafenib in HCC cells [13]. *LncRNA-SRLR* (sorafenib resistance-associated lncRNA in RCC) elicits intrinsic sorafenib resistance in renal cell carcinoma (RCC) [14]. *ZFAS1* (ZNFX1 antisense RNA 1), a lncRNA newly identified in 2011, is shown to be dysregulated in breast cancer [15]. Later, it was found to be an oncogenic lncRNA in multiple human cancers through the regulation of EMT and EMT-regulated genes/miRNAs [16]. The *ZFAS1* gene is frequently amplified in HCC, which promotes metastasis through sponging tumor-suppressive *miR-150* and then activating *ZEB1* (zinc finger E-box-binding homeobox 1), *MMP14* (matrix metallopeptidase 14), and *MMP16* [17]. Thus, *ZFAS1* is viewed as a novel prognostic biomarker for HCC [17,18,19]. However, the role of *ZFAS1* in drug resistance of HCC has not been investigated.

Here, we identified that sorafenib induced *ZFAS1* expression, specifically in sorafenib-resistant HCC cells via the PERK/ATF4-dependent pathway, which is associated with the sorafenib resistance in HCC cells. Inhibition of PERK/ATF4, but not *ZFAS1*, could overcome sorafenib resistance. We concluded that the PERK/ATF4/*ZFAS1* signaling axis may be used as a therapeutic and prognostic biomarker to improve the clinical efficacy of sorafenib in HCC.

## 2. Results

### 2.1. RNA-Sequencing Identifies ZFAS1 as a Sorafenib-Resistant lncRNA

**Figure 1:**
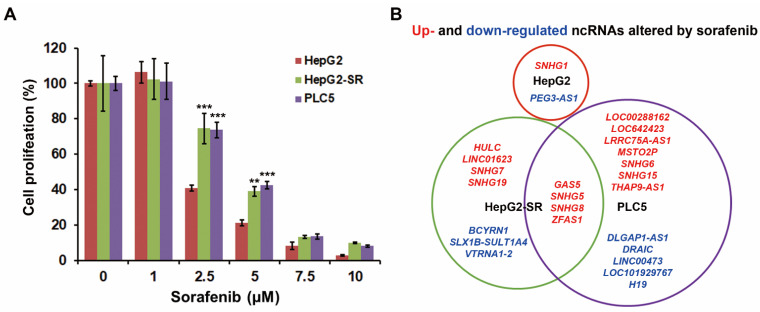
Identification of sorafenib-induced lncRNAs in sorafenib-resistant HCC cells. (**A**) HepG2, HepG2-SR, and PLC5 cells were treated with 0–10 μM sorafenib for 48 h. The cell proliferation was examined by BrdU incorporation assay. ** *p* < 0.01 and *** *p* < 0.001 indicated the statistically significant difference compared to sorafenib-treated HepG2 cells. (**B**) HepG2, HepG2-SR, and PLC5 cells were treated with 5 μM sorafenib for 24 h. Total RNAs were subjected to RNA-sequencing. The altered lncRNAs were shown as a Venn diagram. Genes in red or blue indicated that they were upregulated or downregulated by sorafenib, respectively.

**Figure 2:**
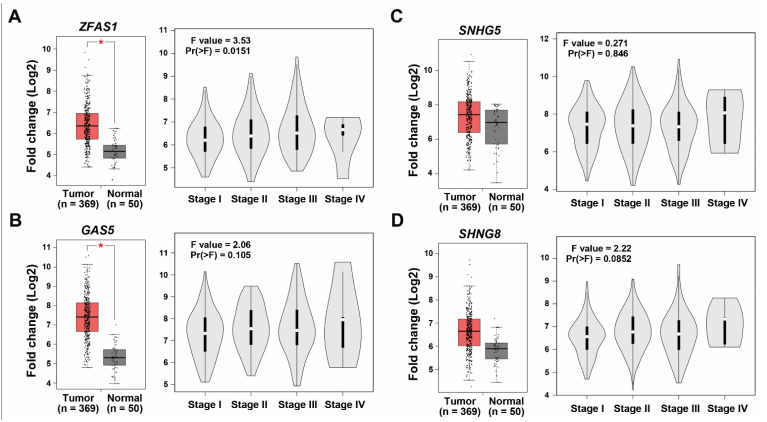
The expression of lncRNAs in HCC. The expressions of *ZFAS1* (**A**), *GAS5* (**B**), *SNHG5* (**C**), and *SNHG8* (**D**) in normal and cancer tissues (left part), and during tumor stage (right part) in HCC patients were obtained from the GEPIA database. * *p* < 0.05 indicated the statistically significant difference compared to the normal group.

**Figure 3:**
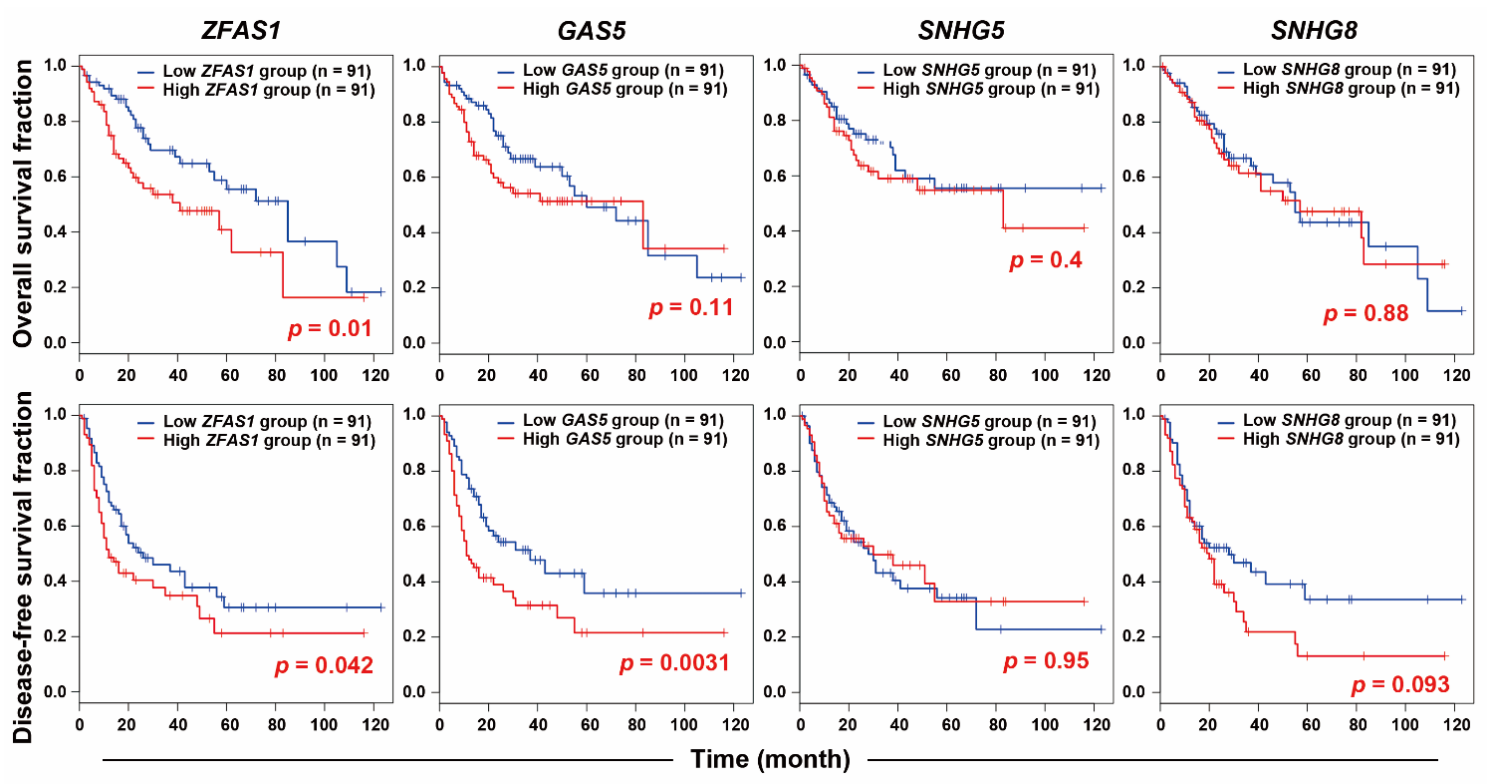
The prognostic values of lncRNAs in HCC. The Kaplan–Meier plots for the relationship between lncRNA expression and overall/disease-free survivals were generated from the GEPIA database. The group cut-off value was set as “quartile”.

In an attempt to elucidate the potential resistant factors or biomarkers for sorafenib resistance, cell models of both primary and acquired sorafenib resistance were employed. It has been reported that HepG2 is a sorafenib-sensitive HCC cell line, whereas PLC5 cells are a sorafenib-resistant HCC cell line [20]. Indeed, our results also showed the resistance of PLC5 cells to sorafenib (Figure 1A), which will be used as a cell model for primary sorafenib resistance. The acquired sorafenib-resistant HepG2-SR cells were established in our previous study [21]. The resistance of HepG2-SR cells to sorafenib was also confirmed in this study (Figure 1A). To identify the potential lncRNAs contributing to sorafenib resistance, RNA-sequencing was performed for HepG2, HepG2-SR, and PLC5 cells treated with or without 5 μM sorafenib for 24 h. The differentially expressed genes (DEGs) are listed in Table S1. The differentially expressed lncRNAs induced by sorafenib in these cells were visualized as a Venn diagram (Figure 1B). We found that sorafenib induced many lncRNAs in sorafenib-resistant HepG2-SR and PLC5 cells, and there are four commonly upregulated lncRNAs, including *GAS5* (growth arrest specific 5), *SNHG5* (small nucleolar RNA host gene 5), *SNHG8*, and *ZFAS1* (Figure 1B). To identify the most important lncRNA(s) in HCC, their gene expressions and prognostic values were analyzed by mining The Cancer Genome Atlas-Liver Hepatocellular Carcinoma (TCGA-LIHC) data via the GEPIA website [22]. As shown in Figure 2, *ZFAS1* and *GAS5*, but not *SNHG5* and *SNHG8* were significantly upregulated in cancer tissues in HCC patients. Only *ZFAS1* was significantly associated with tumor progression (Figure 2), and its high expression predicted poorer overall (*p* = 0.01) and disease-free (*p* = 0.042) survival in HCC patients (Figure 3). Other lncRNAs did not show prognostic values in HCC patients, except for *GAS5*, which had an unfavorable prognostic value in overall survival (*p* = 0.0031) (Figure 3). Therefore, we will focus on investigating the role of *ZFAS1* in sorafenib resistance in HCC.

### 2.2. ZFAS1 Knockdown Does Not Reverse Sorafenib Resistance in HCC Cells

**Figure 4:**
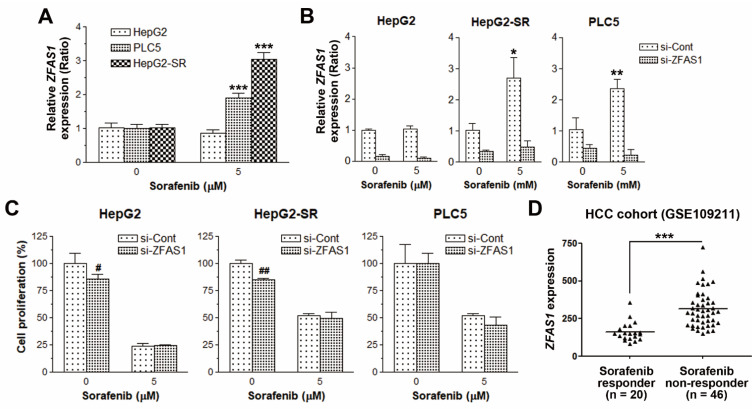
The effect of *ZFAS1* knockdown on sorafenib resistance in HCC cells. (**A**) HepG2, HepG2-SR, and PLC5 cells were treated with 5 μM sorafenib for 18 h, and then *ZFAS1* expression was analyzed by real-time qPCR. *** *p* < 0.001 indicated the statistically significant difference between sorafenib-treated and untreated cells. (**B**,**C**) HepG2, HepG2-SR, and PLC5 cells were transfected with si-ZFAS1 or its negative control siRNA (si-Cont) for 24 h, and then *ZFAS1* expression was analyzed by real-time qPCR (**B**). The transfected cells were treated with 5 μM sorafenib for 48 h. The cell proliferation was examined by BrdU incorporation assay (**C**). * *p* < 0.05 and ** *p* < 0.01 indicated the statistically significant difference between sorafenib-treated and untreated cells. ^#^ *p* < 0.05 and ^##^ *p* < 0.01 indicated the statistically significant difference between si-Cont and si-ZFAS1-transfected cells. (**D**) The *ZFAS1* mRNA expression levels in sorafenib responsive and non-responsive HCC patients were obtained from the microarray data set (GSE109211). *** *p* < 0.001 indicated the statistically significant difference between sorafenib responders and non-responders.

To validate whether sorafenib induced *ZFAS1* expression specifically in sorafenib-resistant HCC cells, a real-time quantitative polymerase chain reaction (qPCR) was performed. As shown in Figure 4A, *ZFAS1* was indeed induced by sorafenib in sorafenib-resistant HepG2-SR and PLC5 cells, but not sorafenib-sensitive HepG2 cells. To confirm the role of *ZFAS1* in sorafenib resistance, its expression was knocked down by siRNA (Figure 4B). However, *ZFAS1* knockdown was not sufficient to reverse sorafenib resistance in both HepG2-SR and PLC5 cells (Figure 4C). Therefore, we hypothesized that, although *ZFAS1* itself did not contribute to the sorafenib resistance, its upregulation by sorafenib may act as a predictive prognostic biomarker in HCC patients receiving sorafenib therapy. To demonstrate this hypothesis, an HCC cohort containing sorafenib responders and non-responders (GSE109211 [23]) was employed. Indeed, *ZFAS1* tended to express higher in sorafenib non-responders (Figure 4D).

### 2.3. Pathway Enrichment Predicts a Potential Role of UPR in Sorafenib Resistance

**Figure 5:**
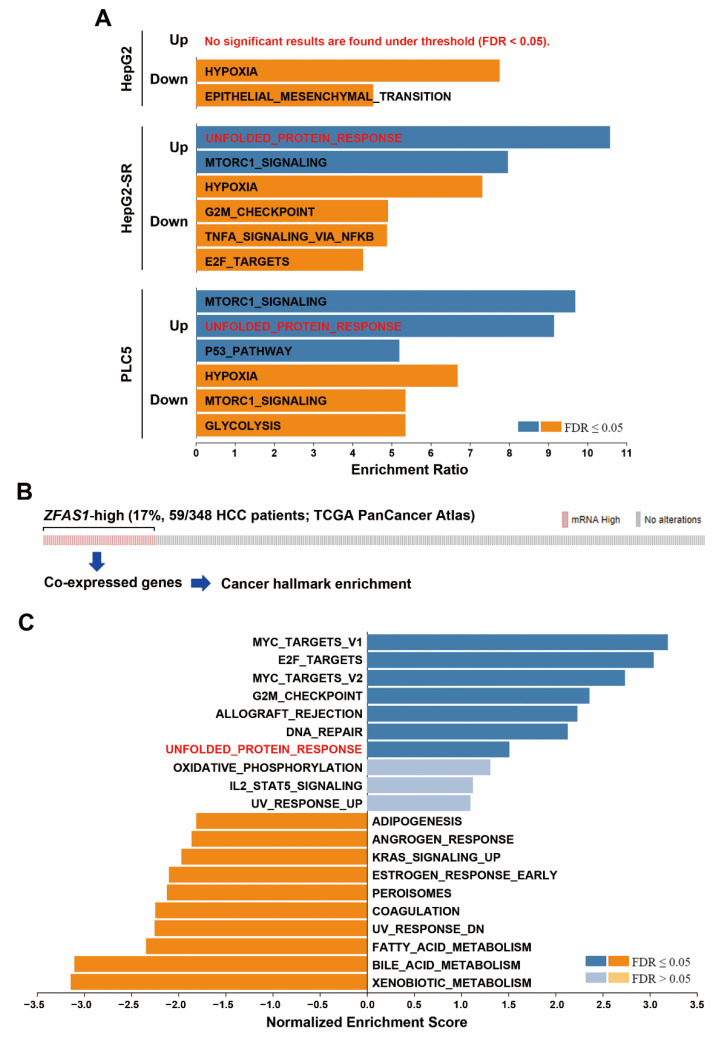
Pathway enrichment for *ZFAS1*-associated cancer hallmark. (**A**) The RNA-sequencing data in Table S1 were analyzed using the WebGestalt for cancer hallmark analysis. (**B**) The OncoPrint for *ZFAS1* mRNA expression in TCGA-LIHC dataset. (**C**) The over- and under-expressed genes in *ZFAS1*-high expressing HCC patients were obtained from the cBioPortal website and these genes were analyzed using the WebGestalt for cancer hallmark analysis.

**Figure 6:**
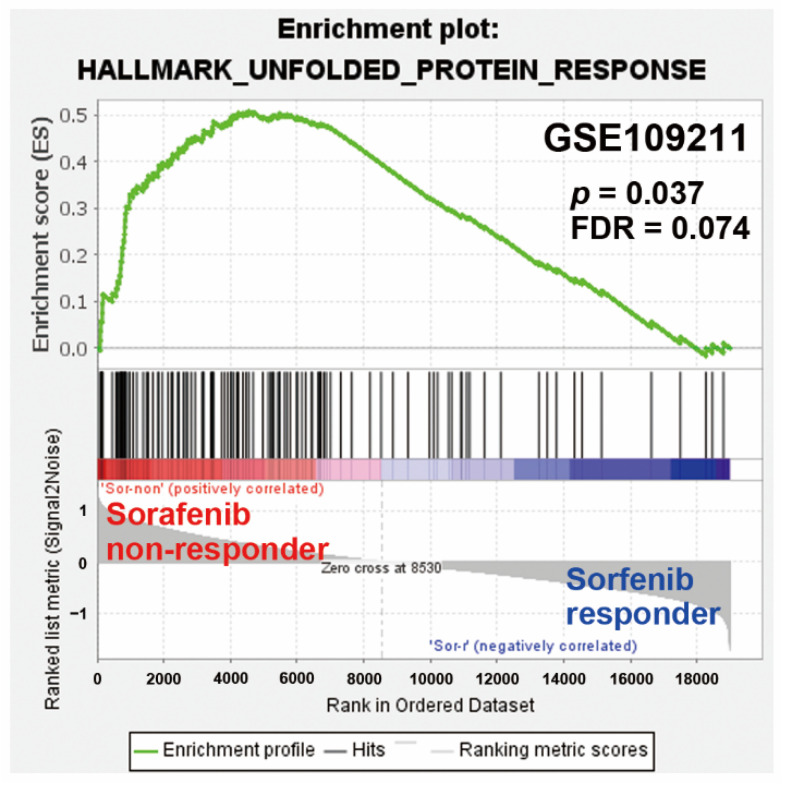
GSEA for UPR genes in HCC patients receiving sorafenib therapy. The microarray dataset (GSE109211) of sorafenib responsive and non-responsive HCC patients were analyzed by GSEA for the enrichment of the UPR cancer hallmark.

To identify the potential signaling pathways linking to the upregulation of *ZFAS1* in response to sorafenib in sorafenib-resistant HCC cells, the RNA-sequencing data (Table S1) were further analyzed, using the WebGestalt for cancer hallmark enrichment [24,25]. As shown in Figure 5A, UPR and mTORC1 (mammalian target of rapamycin complex 1) signaling were two commonly enriched cancer hallmarks upregulated by sorafenib in HepG2-SR and PLC5 cells, implying their roles in sorafenib resistance. To further investigate the relationship of *ZFAS1* with these cancer hallmarks, *ZFAS1*-high expressing HCC patients were selected from the TCGA-LIHC dataset (via the cBioPortal website [26,27]) to obtain the *ZFAS1*-associated over- and under-expressed genes. As shown in Figure 5B, 59 out of 348 (17%) HCC patients exhibited higher *ZFAS1* expression. The *ZFAS1*-associated genes (Table S2) were also analyzed by the WebGestalt and it was found that UPR was one of the enriched cancer hallmarks (Figure 4C). Furthermore, like *ZFAS1*, UPR was also enriched in sorafenib non-responders (Figure 6). Therefore, *ZFAS1* upregulation may be associated with ER stress/UPR in sorafenib-resistant HCC.

### 2.4. PERK/ATF4 Inhibitors Inhibits ZFAS1 Expression and Reverses Sorafenib Resistance

**Figure 7:**
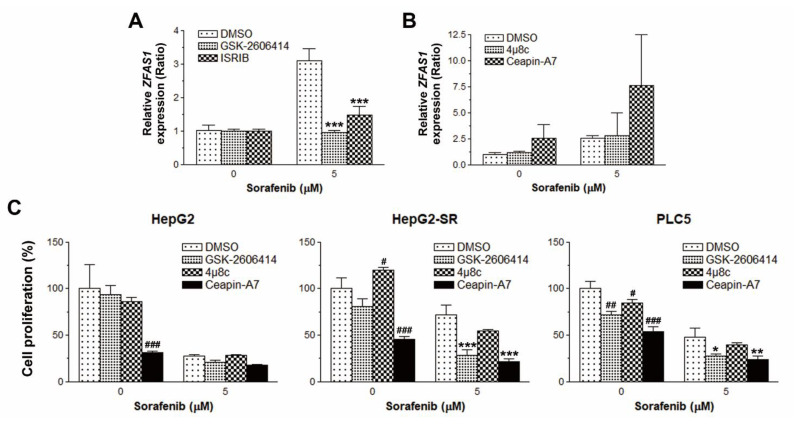
The effect of UPR inhibitors on sorafenib-induced *ZFAS1*. (**A**,**B**) PLC5 cells were pretreated with 10 μM GSK-2606414, 0.2 μM ISRIB, 10 μM 4μ8C, or 10 μM Ceapin-A7 for 0.5 h, and then exposed to 5 μM sorafenib for 18 h. *ZFAS1* expression was analyzed by real-time qPCR. *** *p* < 0.001 indicated the statistically significant difference between sorafenib-treated and UPR inhibitor/sorafenib-treated cells. (**C**) HepG2, HepG2-SR, and PLC5 cells were treated with 5 μM sorafenib for 48 h in the absence or presence of 10 μM GSK-2606414, 10 μM 4μ8C, or 5 μM Ceapin-A7. The cell proliferation was examined by BrdU incorporation assay. ^#^ *p* < 0.05, ^##^ *p* < 0.01, and ^###^ *p* < 0.001 indicated the statistically significant difference between untreated and UPR inhibitor-treated cells. * *p* < 0.05, ** *p* < 0.01, and *** *p* < 0.001 indicated the statistically significant difference between sorafenib-treated and UPR inhibitor/sorafenib-treated cells.

To investigate whether ER stress/UPR was responsible for the induction of *ZFAS1* by sorafenib, chemical inhibitors of PERK (GSK-2606414), ATF4 (ISRIB), IRE1 (4μ8c), and ATF6 (Ceapin-A7) were used. As shown in Figure 7A,B, only GSK-2606414 and ISRIB could suppress sorafenib-induced *ZFAS1* expression in PLC5 cells, suggesting that the PERK/ATF4 arm of UPR is responsible for the upregulation of *ZFAS1* by sorafenib in sorafenib-resistant HCC cells. To ascertain the role of three arms of UPR in sorafenib resistance, HepG2, HepG2-SR, and PLC5 cells were pretreated with GSK-2606414, 4μ8c, and Ceapin-A7, and then exposed to sorafenib. The cell proliferation assay indicated that GSK-2606414 enhanced the anticancer activity of sorafenib in HepG2-SR and PLC5 cells (Figure 7C). Therefore, the PERK/ATF4 arm of UPR is responsible for sorafenib resistance in HCC cells.

## 3. Discussion

ER stress/UPR was identified to participate in hepatocarcinogenesis. The expression of *GRP78* and *ATF6* mRNAs and the splicing of *XBP1* mRNA were elevated in HCC tissues with increased histological grading [28]. In a hepatitis B virus (HBV) surface antigen (HBsAg)-driven HCC model, prolonged ER stress leads to the accumulation of DNA damage in hepatocytes and promotes HCC incidence [29]. *CHOP*-knockout mice develop smaller tumor nodules, relative to wild-type mice, in a carcinogen-induced HCC model [30]. Tauroursodeoxycholic acid (TUDCA), a well-known chemical chaperone, retards *Mst1*/*2* (macrophage-stimulating 1/2) mutant-driven liver tumorigenesis in mice [31]. Therefore, inhibition of ER stress/UPR may have clinical benefits for HCC.

It was found that sorafenib activates the PERK and IRE1 arms of UPR but inhibits the ATF6 arm in HCC cells [32,33]. However, the inhibitors of PERK (GSK-2606414) and IRE1 (4μ8c) had no or minimal inhibitory effect on the clonogenic growth of HCC cells exposed to sorafenib [34]. In another study, sorafenib activated three UPR arms and ER stress inhibitors, TUDCA and 4-phenylbutyrate (4-PBA), and enhanced sorafenib-induced apoptosis in HCC cells, suggesting a protective role of ER stress/UPR [35]. Moreover, it was characterized that sorafenib-induced IRE1 arm of UPR is responsible for the induction of autophagy that counteracts with sorafenib-induced ER stress-dependent apoptosis in HCC cells [36]. In addition, early induction of ER stress/UPR by sorafenib is correlated with the induction of pro-survival autophagy, whereas prolonged ER stress/UPR during sustained sorafenib treatment leads to the shift from autophagy to apoptosis in HCC cells [37]. Given the ambiguous role of ER stress/UPR in the sorafenib sensitivity of HCC cells, modulation of ER stress/UPR by genetic manipulation and pharmacological intervention can either sensitize or block the anticancer activity of sorafenib in HCC cells [35,38,39,40,41,42,43]. Therefore, the exact role of ER stress/UPR in sorafenib sensitivity of HCC warrants further investigations.

How *ZFAS1* is transcriptionally regulated is still largely unclear. The only experimentally confirmed transcription factor binding site on the *ZFAS1* promoter is the SP1 site [44,45]. In addition, there are two potential upstream transcription factor 1 (USF1)-binding sites on the *ZFAS1* promoter, which was predicted in a previous study without any experimental evidence [46]. Therefore, more investigations are needed to understand how to regulate *ZFAS1* gene transcription. Our results suggest that *ZFAS1* may also be transcriptionally regulated by ATF4. Supportively, two potential ATF4-binding sites on *ZFAS1* gene promoter were predicted using the JASPAR database (http://jaspar.genereg.net/, last accessed on 27 March 2021) [47] (Figure S1), which warrants further investigation.

One limitation in this study is that the RNA-sequencing data were obtained from two biological replicates, which may cause false-positive or false-negative results. It is proposed that at least 6 biological replicates should be used for RNA-sequencing experiments, and the numbers should be increased to 12 when identifying significant DEGs [48]. In contrast, according to the “ENCODE Experimental Guidelines for ENCODE3 RNA-seq” at https://www.encodeproject.org/about/experiment-guidelines/ (last accessed on 26 April 2021), two or more biological replicates should be performed for RNA-sequencing experiments. Although the RNA-sequencing experimental design in this study was not the most optimized, subsequent in vitro experiments could support the conclusion.

In conclusion, this study identifies *ZFAS1* as a sorafenib-inducible lncRNA, specifically in sorafenib-resistant HCC cells. Mechanistically, sorafenib induces *ZFAS1* via the activation of the PERK/ATF4 arm of UPR. Interestingly, inhibition of PERK/ATF4, but not *ZFAS1*, could overcome sorafenib resistance. Our results provide a molecular basis for using *ZFAS1* as a therapeutic and prognostic biomarker to predict the clinical efficacy of sorafenib therapy in HCC. In addition, the inhibition of PERK/ATF4 may be an attractive strategy to overcome sorafenib resistance.

## 4. Materials and Methods

### 4.1. Chemicals and Reagents

The RNeasy Kit, RT^2^ First Strand Kit, RT^2^ SYBR Green ROX qPCR Mastermix, and RT^2^ lncRNA qPCR Assay for Human ZFAS1 were purchased from Qiagen (Valencia, CA, USA). The BrdU Cell Proliferation Assay Kit was purchased from BioVision (Mountain View, CA, USA). The sorafenib was purchased from LC Laboratories (Woburn, MA, USA). The GSK-2606414 was purchased from APExBIO (Boston, MA, USA). The trans-ISRIB was purchased from Tocris Bioscience (Ellisville, MO, USA). Ceapin-A7 and 4μ8c were purchased from Sigma-Aldrich (St. Louis, MO, USA). Silencer Select pre-designed siRNA for human *ZFAS1*, negative control siRNA, and RNAiMAX transfection reagent were purchased from Thermo Fisher Scientific (Wilmington, DE, USA).

### 4.2. Cell Culture and Cell Proliferation Assay

Human HCC cells, HepG2 and PLC/PRF/5 (PLC5), were purchased from the Bioresource Collection and Research Center (Hsinchu, Taiwan). The sorafenib-resistant HepG2 (HepG2-SR) cells were established in our previous study [21]. Cells were cultured in DMEM with the following supplements: 10% fetal bovine serum (FBS), 1% L-glutamine, 1 mM sodium pyruvate, and 1% antibiotic-antimycotic solution (100 units/mL penicillin, 100 µg/mL streptomycin, and 0.25 µg/mL of Gibco Amphotericin B). The cell proliferation was examined using the bromodeoxyuridine (BrdU) cell proliferation assay kit, which is based on the ability of proliferating cells to incorporate BrdU, the thymidine analog, into newly synthesized DNA strands.

### 4.3. RNA-Sequencing and Real-Time Quantitative Polymerase Chain Reaction (qPCR)

HepG2 and PLC5 cells were treated with 5 μM sorafenib for 24 h. Total RNA was isolated by the RNeasy Kit. The extracted RNA was quantified by the Nanodrop (Thermo Fisher Scientific, Wilmington, DE, USA) and the RNA quality was checked by the ratio of absorbance at 260 nm and 280 nm. Sequencing (two biological replicates) was performed using the BGISEQ-500 platform (Beijing Genomics Institute, Beijing, China) to averagely generate 23,568,563 clean reads. Gene expression levels were quantified by a software package called RSEM [49]. DEGs were screened using the NOISeq method [50] according to the following criteria: |fold-change| ≥ 2 and diverge probability ≥ 0.8. The full DEGs are shown in Table S1. The VENNY 2.1 online tool [51] was used to compare the overlapped genes and generate the Venn diagram. For the determination of *ZFAS1* expression, the first-strand cDNA was synthesized using the RT^2^ First Strand Kit. Then, PCR amplification was performed in triplicate on the Applied Biosystems ABI 7500 Real-Time PCR System (Foster City, CA, USA), using the RT^2^ lncRNA qPCR Assay for human *ZFAS1* gene and the RT^2^ SYBR Green ROX qPCR Mastermix for human 18S ribosomal (r)RNA (forward 5′-CGGCGACGACCCATTCGAAC-3′ and reverse 5′-GAATCGAACCCTGATTCCCCGTC-3′). No-revere transcription controls were included in each run to detect potential contamination. Gene expression fold-changes were calculated by the comparative CT method, using the 18S rRNA as the reference gene and untreated cells as the calibrator.

### 4.4. siRNA Knockdown Analysis

HepG2 and PLC5 cells were transfected with *ZFAS1* siRNA and the negative control siRNA using RNAiMAX transfection reagent according to the manufacturer’s instruction. Twenty-four hours later, the transfection mixture was replaced with the regular medium, and cells were prepared for further experiments.

### 4.5. Bioinformatics Tools

The GEPIA (Gene Expression Profiling Interactive Analysis) database at the website (http://gepia2.cancer-pku.cn/ [22]) was used to compare the lncRNA levels in normal/cancer liver tissues and their impacts on cancer patients’ overall/disease-free survivals in TCGA-LIHC data set. The cBioPortal (http://www.cbioportal.org/ [25,26]) was used to obtain the over- and under-expressed genes (Table S2) associated with *ZFAS1*-high expressing patients in TCGA-LIHC (PanCancer Atlas) dataset. Genes in Tables S1 and S2 were subjected to cancer hallmark enrichment using the WebGestalt (WEB-based Gene SeT AnaLysis Toolkit; http://www.webgestalt.org/ [13,24]). The over-representation analysis (ORA) and gene set enrichment analysis (GSEA) approaches were used for analyzing sorafenib-induced DEGs (Table S1) and *ZFAS1*-associated genes (Table S2), respectively. The GSEA v3.0 software (http://www.broadinstitute.org/gsea/ [14,15]) was used for the enrichment of *ZFAS1* and cancer hallmarks in an HCC patient cohort (GSE109211 [27]). The last accessed date for the above analyses was 23 March 2021.

### 4.6. Statistical Analysis

All in vitro experimental results (cell proliferation assay and qPCR) were represented by the mean ± standard deviation of at least three independent experiments. Statistical analysis was performed using a two-way analysis of variance (ANOVA) with the Bonferroni post-test. A *p*-value less than 0.05 was considered statistically significant.

## Data Availability

The datasets used in this article are publicly available as described in Materials and Methods.

## Supplementary Materials

The following are available online at https://www.mdpi.com/article/10.3390/ijms22115848/s1.
