# Supplementary material for: PERK/ATF4-Dependent ZFAS1 Upregulation Is Associated with Sorafenib Resistance in Hepatocellular Carcinoma Cells
Source: Int J Mol Sci. 2021 May 29;22(11):5848. doi: 10.3390/ijms22115848 (PMC8199104; doi:10.3390/ijms22115848)
Supplement: Supplementary file 1 [file ijms-22-05848-s001.zip › ijms-1180112-supplementary.pdf]

Table S1. The differentially expressed genes in sorafenib-treated HCC cells. HepG2, HepG2-SR, and PLC5 cells were treated with 5 μM sorafenib for 24 h, and then total RNAs were analyzed by the RNA-sequencing (RNA-Seq). The differentially expressed genes (DEGs) with absolute log2 fold-change ratio more than 1 were shown.

**HepG2**		**HepG2-SR**		**PLC5**	
**Up**	**Down**	**Up**	**Down**	**Up**	**Down**
RNU6-2	HSPA5	U2AF1L5	CCL20	FAM129A	HNRNPA1
RNU6-9	AFP	CAPN3	LYZ	AKNA	CXCL16
CSNK2A3	PCNA	NUPR1	CSE1L	ALDH1L2	CXCL5
NUPR1	MAGED1	JDP2	MATN3	GADD45A	STMN1
CAPN3	BMP4	BEX2	TMSB10	GAS5	PGAM1
ADGRG1	LDHA	ASNS	HMGA1	TRIB3	TFRC
DPEP1	PEG3-AS1	SLC6A9	RNU6-1	ZFAS1	TESC
ABCC3	PGK1	TRIM50	UBE2C	LOC642423	NTS
CYP4F12	INSIG2	DDIT3	BHLHE40	SLC22A15	CD24
HAMP	TGFBR2	INHBE	TUBA1C	LAMP3	MARCKSL1
GSTA1	PET100	SLC17A2	SAA4	BEX2	HNRNPM
DIO1	IL1RN	MTHFD2	UHRF1	NUPR1	FGA
SERPINC1	CKB	TRIB3	GINS2	PABPC1L	MAGEA4
COL16A1	APOM	GPAM	IGFBP1	INHBE	RPL36A-
SLC22A7	SERPINA11	CEBPB	TUBA1B	MSTO2P	HNRNPH2
HKDC1	PLOD2	SHBG	S100A6	CLGN	TGFBR2
ETNK2	SLC6A8	SAT2	MYBL2	HSPA1B	BLOC1S1
ANGPTL8	SAA4	CBS	SLC25A10	PCK2	ALDOA
ENO3	C17orf96	IFRD1	C8orf4	PPP1R15A	TUBB4B
GPD1	BNIP3L	ARL2-SNX15	LOC729083	TUBE1	AURKA
GTF2IRD1	RNU6-1	A1BG	LGALS3	SLC3A2	BNIP3
CES1	C5	NR1H4	UBE2T	C6orf48	KIAA0101
KHDRBS3	SEC61G	CCPG1	ZWINT	PHGDH	MAP3K8
ASS1	ERRFI1	PCSK9	MKI67	ANKRD1	PPP2R1B
UNC13D	HNRNPA2B1	INPP1	CTGF	SNHG15	NR2F2
PPDPF	SLC38A2	CEBPG	S100A11	SLC1A4	CCL20
SAT2	NDRG1	SNHG8	IER3	ABLIM3	PGK1
PRAP1	HSD17B2	CYP4F12	G6PC3	HSPA1A	HMGB3
ITPKA	CP	PHGDH	RRM2	ARHGEF2	H2AFX
SMIM24	ACSL4	GAS5	RNU6-2	NPIPA1	NUSAP1
SNHG1	TGFBR3	SARS	RNU6-9	LHPP	RDH10
MTSS1L	AKAP12	ASS1	LGALS1	TCEA1	DHRS3
INHBE	DUSP6	YARS	FEN1	ATF3	SFPQ
LOC102724652	THBS1	MARS	CITED2	HKDC1	SLC2A3
AARS	RAB42	SHMT2	CEMIP	U2AF1L4	LDHA
FAM3B	PEG10	RN7SL2	MIXL1	CCNB1IP1	C1GALT1C1
SIAH2	RRM2	HULC	ANKRD1	WARS	PCNA-AS1
DGAT2	CCL15	SERPINC1	TK1	AKR1C2	IDI1
SEMA3B	FILIP1L	XPOT	JUN	ANXA3	AFP
ASNS	C8orf4	AKR1C2	CDC45	AKR1C1	NR4A2
CHCHD10	LYZ	ZFAS1	SFN	SNHG8	SCD
DCXR	LCN15	SNHG19	SH3BGRL3	ASNS	PCDH9
TCEA3	SERPINA3	MAP1LC3B	PCK1	ALDH2	PDK4
SMARCB1	PFKFB4	SNHG7	THBS1	ZRSR2	TP53INP2
	EGR1	BRI3	KRT23	CHCHD10	CXCL12
	MATN3	ASAH1	ZFP36	SARS	ERO1A
	C10orf10	ATF4	MT2A	CEBPG	FST
	FST	LINC01623	CDC6	EIF4EBP1	CXCL1
	CTGF	SNHG5	NME1-NME2	LOC100288162	MUC13
	SLC2A3		TIMP1	THAP9-AS1	IGFBP3

TUBB1	TAGLN	PKD1P6-NPIPP1	ADGRL2
IGFBP3	AKAP12	ZFAND1	FGB
LOX	DUSP1	PSAT1	DLGAP1-AS1
CA9	ADM	MTRNR2L9	AHSG
CCL20	CYR61	TES	PLIN2
SLC6A14	EGR1	TAF1D	CGA
IGFBP1	SNX15	DDIT3	CP
EDN1	BCYRN1	GARS	SSX2
	FOS	LRRC75A-AS1	RRM2
	VTRNA1-2	RP9	FUT11
	SLX1B-SULT1A4	MTRNR2L10	KIF20A
		ZFAND2A	ALB
		MTRNR2L6	C4orf3
		SHMT2	AGTR1
		SNHG5	SPTSSB
		SNHG6	PLOD2
		MTHFD2	TM4SF4
		MTRNR2L1	IGFBP1
		GFPT1	CRH
		TSEN15	LINC00473
		YARS	FABP1
		MARS	DRAIC
		HERPUD1	EGR1
		MTRNR2L8	H19
		PYCR1	HOTS
		RPL12	ADM
		SAT1	HILPDA
		IDH1	CITED2
			LOC101929767
			ALDOC
			ANGPTL4
			BNIP3L
			SLC1A6
			FAM13A
			PFKFB4
			INSIG1
			CYP26A1
			CA9
			NDRG1
			ANKRD37
			HAL

Table S2. The ZFAS1-associated genes in TCGA-LIHC data set. Over- and under-expressed genes in ZFAS1-high-expressing HCC patients were obtained from the cBioPortal website using the "Comparison" tool.

Gene	Log Ratio	p-Value	q-Value	Tendency
SPHK1	2.48	3.75E-10	7.51E-09	Over-expressed
PDE9A	2.17	4.06E-12	1.54E-10	Over-expressed
PYCR1	2.11	2.12E-07	1.70E-06	Over-expressed
MMP9	2.1	2.26E-12	9.52E-11	Over-expressed
LSP1P5	2.08	1.95E-12	8.43E-11	Over-expressed
ZFAS1	2.05	5.18E-40	6.24E-36	Over-expressed
TESC	1.99	1.16E-07	1.01E-06	Over-expressed
SNHG6	1.93	4.28E-23	5.42E-20	Over-expressed
SLC39A4	1.93	2.89E-06	1.59E-05	Over-expressed
LSP1P4	1.91	6.09E-16	6.33E-14	Over-expressed
SNHG3	1.88	1.33E-17	2.29E-15	Over-expressed
GAS5	1.84	2.37E-26	1.43E-22	Over-expressed
MFSD2B	1.81	3.14E-18	7.27E-16	Over-expressed
H2AFY2	1.81	7.46E-09	9.57E-08	Over-expressed
TMSB10	1.79	5.22E-16	5.57E-14	Over-expressed
GGTLC2	1.77	1.07E-07	9.45E-07	Over-expressed
MEG3	1.77	2.22E-05	9.40E-05	Over-expressed
MAPK13	1.76	6.54E-08	6.18E-07	Over-expressed
TRNP1	1.76	8.71E-08	7.95E-07	Over-expressed
NAT14	1.73	2.92E-15	2.65E-13	Over-expressed
PSMC3IP	1.72	5.60E-13	2.79E-11	Over-expressed
SH2D3A	1.72	6.96E-09	9.01E-08	Over-expressed
SLC29A4	1.71	1.43E-05	6.39E-05	Over-expressed
NMB	1.66	1.31E-17	2.29E-15	Over-expressed
ANKRD19P	1.66	2.04E-14	1.61E-12	Over-expressed
MDK	1.65	1.79E-09	2.79E-08	Over-expressed
RPL8	1.64	1.89E-18	4.84E-16	Over-expressed
RPL39	1.63	1.83E-16	2.22E-14	Over-expressed
AURKB	1.63	3.79E-11	1.09E-09	Over-expressed
BIRC5	1.63	3.56E-10	7.15E-09	Over-expressed
RPL13AP20	1.61	1.55E-17	2.58E-15	Over-expressed
SLC6A8	1.6	1.72E-07	1.43E-06	Over-expressed
SPP1	1.59	8.81E-04	2.33E-03	Over-expressed
CYTOR	1.57	7.33E-14	5.02E-12	Over-expressed
TMEM74B	1.57	4.01E-10	7.96E-09	Over-expressed
TMED3	1.57	2.07E-07	1.66E-06	Over-expressed
RPS21	1.55	5.32E-20	2.67E-17	Over-expressed
RPS19	1.55	3.58E-19	1.31E-16	Over-expressed
ZNF296	1.55	6.28E-09	8.24E-08	Over-expressed
ASNS	1.55	6.72E-08	6.33E-07	Over-expressed
SNHG29	1.52	2.67E-21	1.89E-18	Over-expressed
PRR7	1.52	1.59E-13	9.92E-12	Over-expressed
SNHG5	1.51	2.97E-13	1.70E-11	Over-expressed
PAFAH1B3	1.51	1.07E-11	3.60E-10	Over-expressed
CYBA	1.5	2.16E-08	2.40E-07	Over-expressed
LTB	1.5	5.71E-08	5.49E-07	Over-expressed
MYBL2	1.5	1.83E-07	1.50E-06	Over-expressed
LRRC1	1.49	2.45E-09	3.67E-08	Over-expressed
DNM1	1.49	1.33E-06	8.11E-06	Over-expressed
RPS28	1.49	9.02E-04	2.38E-03	Over-expressed
RPL13AP3	1.48	5.86E-13	2.87E-11	Over-expressed
MAFG-DT	1.48	1.63E-10	3.71E-09	Over-expressed

UBD	1.48	1.64E-08	1.88E-07	Over-expressed
RAB34	1.48	4.52E-06	2.35E-05	Over-expressed
RPL37A	1.47	3.34E-21	2.24E-18	Over-expressed
RPL23A	1.47	8.48E-20	3.78E-17	Over-expressed
RPL28	1.47	4.11E-19	1.46E-16	Over-expressed
PTTG1	1.46	2.70E-11	8.15E-10	Over-expressed
SAPCD2	1.46	4.75E-09	6.50E-08	Over-expressed
RPLP2	1.45	9.25E-19	2.59E-16	Over-expressed
RPS24	1.44	1.24E-23	3.00E-20	Over-expressed
LINC02381	1.44	1.52E-04	5.05E-04	Over-expressed
CD7	1.43	9.50E-06	4.49E-05	Over-expressed
GNAZ	1.42	6.33E-08	6.00E-07	Over-expressed
RPL26	1.41	9.94E-22	7.98E-19	Over-expressed
RPL27A	1.41	2.60E-19	1.03E-16	Over-expressed
RPS12	1.41	6.98E-17	9.34E-15	Over-expressed
SNHG7	1.41	6.04E-15	5.27E-13	Over-expressed
HMGA1	1.41	3.28E-14	2.46E-12	Over-expressed
DBN1	1.41	1.18E-08	1.41E-07	Over-expressed
UBE2C	1.41	1.51E-08	1.75E-07	Over-expressed
RPL13	1.4	1.95E-16	2.32E-14	Over-expressed
MSS51	1.4	1.96E-16	2.32E-14	Over-expressed
TEAD4	1.4	2.23E-08	2.48E-07	Over-expressed
RPS20	1.39	3.66E-17	5.45E-15	Over-expressed
KCTD17	1.39	4.54E-08	4.52E-07	Over-expressed
NQO1	1.39	5.22E-03	0.011	Over-expressed
RPS10	1.38	3.63E-22	3.36E-19	Over-expressed
EEF1D	1.38	1.47E-15	1.39E-13	Over-expressed
SH3D21	1.38	5.02E-13	2.55E-11	Over-expressed
S100A6	1.38	4.52E-07	3.24E-06	Over-expressed
RPL36	1.37	1.34E-15	1.28E-13	Over-expressed
NPM3	1.37	5.51E-13	2.76E-11	Over-expressed
PTP4A3	1.37	2.69E-07	2.08E-06	Over-expressed
SLC29A2	1.37	3.56E-07	2.64E-06	Over-expressed
RPSA	1.36	2.53E-18	5.97E-16	Over-expressed
FBL	1.36	2.85E-16	3.21E-14	Over-expressed
SLC16A3	1.36	1.61E-06	9.60E-06	Over-expressed
EPB41L4A-AS1	1.35	5.77E-24	2.32E-20	Over-expressed
RPS17	1.35	3.06E-23	5.42E-20	Over-expressed
RPS2	1.35	3.15E-23	5.42E-20	Over-expressed
ZNF581	1.35	2.48E-20	1.36E-17	Over-expressed
RPL18A	1.35	1.88E-17	2.95E-15	Over-expressed
RPL38	1.34	3.32E-17	5.00E-15	Over-expressed
TYRO3	1.34	2.03E-05	8.69E-05	Over-expressed
KLC3	1.34	2.19E-05	9.28E-05	Over-expressed
RPL37	1.33	1.49E-20	8.54E-18	Over-expressed
C19ORF48	1.33	7.34E-16	7.56E-14	Over-expressed
SELENOM	1.33	4.05E-06	2.14E-05	Over-expressed
GPR35	1.33	1.46E-04	4.88E-04	Over-expressed
RPL35	1.32	2.30E-16	2.65E-14	Over-expressed
RPLP0	1.31	2.66E-22	2.68E-19	Over-expressed
RPL34	1.31	4.53E-18	9.58E-16	Over-expressed
RPL30	1.31	2.09E-16	2.44E-14	Over-expressed
DANCR	1.31	2.51E-14	1.92E-12	Over-expressed
RPL22L1	1.31	7.16E-11	1.86E-09	Over-expressed
TMEM132A	1.31	2.13E-05	9.09E-05	Over-expressed
RPS9	1.3	5.28E-19	1.63E-16	Over-expressed
SYNGR1	1.3	1.51E-05	6.69E-05	Over-expressed

CD24	1.3	4.80E-04	1.38E-03	Over-expressed
RPS8	1.29	4.29E-23	5.42E-20	Over-expressed
RPL32	1.29	5.41E-19	1.63E-16	Over-expressed
RPL18	1.29	6.39E-18	1.24E-15	Over-expressed
SMOX	1.29	1.72E-09	2.71E-08	Over-expressed
RECQL4	1.29	5.53E-09	7.40E-08	Over-expressed
CDC20	1.29	9.65E-08	8.68E-07	Over-expressed
PKIB	1.29	3.73E-04	1.11E-03	Over-expressed
SNHG17	1.28	4.54E-19	1.51E-16	Over-expressed
RPSAP58	1.28	2.16E-18	5.20E-16	Over-expressed
SNHG8	1.28	7.47E-16	7.56E-14	Over-expressed
ENO2	1.28	1.52E-05	6.75E-05	Over-expressed
ARID3A	1.28	3.23E-05	1.30E-04	Over-expressed
RPS27A	1.27	8.48E-20	3.78E-17	Over-expressed
RPS16	1.27	9.98E-18	1.79E-15	Over-expressed
CD3D	1.27	9.18E-06	4.36E-05	Over-expressed
TUBB3	1.26	4.97E-09	6.76E-08	Over-expressed
RPL23	1.25	5.65E-20	2.72E-17	Over-expressed
RPL27	1.25	1.99E-18	4.90E-16	Over-expressed
RPL13A	1.25	5.90E-18	1.23E-15	Over-expressed
RPL14	1.25	6.38E-18	1.24E-15	Over-expressed
RPS5	1.25	1.88E-15	1.76E-13	Over-expressed
HSPB1P1	1.25	2.62E-11	7.93E-10	Over-expressed
SLC1A5	1.24	2.51E-07	1.96E-06	Over-expressed
PHLDA2	1.24	5.49E-05	2.07E-04	Over-expressed
RPS27	1.24	6.48E-05	2.39E-04	Over-expressed
RPL31	1.23	9.90E-24	2.98E-20	Over-expressed
RPS3	1.23	6.28E-18	1.24E-15	Over-expressed
SNHG12	1.23	4.74E-17	6.81E-15	Over-expressed
RPS29	1.23	4.45E-14	3.19E-12	Over-expressed
SNRPD2	1.23	3.49E-13	1.88E-11	Over-expressed
RANGRF	1.23	9.25E-13	4.36E-11	Over-expressed
BGLAP	1.23	3.52E-10	7.09E-09	Over-expressed
WDR54	1.23	1.46E-08	1.71E-07	Over-expressed
TMC6	1.23	2.35E-07	1.84E-06	Over-expressed
SOX4	1.23	2.60E-07	2.02E-06	Over-expressed
RPLP1	1.22	4.13E-17	6.07E-15	Over-expressed
CENPM	1.22	1.04E-07	9.25E-07	Over-expressed
LPAR2	1.22	1.23E-05	5.60E-05	Over-expressed
RPL9	1.22	4.82E-05	1.85E-04	Over-expressed
RPS14	1.21	1.69E-19	7.29E-17	Over-expressed
CLIC1	1.21	6.11E-18	1.24E-15	Over-expressed
RPS15	1.21	2.31E-16	2.65E-14	Over-expressed
EVA1B	1.21	1.42E-14	1.16E-12	Over-expressed
MYL6B	1.21	9.76E-14	6.43E-12	Over-expressed
SELENOW	1.21	8.08E-09	1.02E-07	Over-expressed
EFNA3	1.21	7.48E-08	6.93E-07	Over-expressed
TROAP	1.21	2.53E-07	1.97E-06	Over-expressed
LOC606724	1.21	5.15E-07	3.62E-06	Over-expressed
ISYNA1	1.21	6.69E-05	2.46E-04	Over-expressed
CKB	1.21	2.48E-04	7.75E-04	Over-expressed
CAPG	1.2	2.02E-08	2.26E-07	Over-expressed
RHPN1	1.2	3.51E-07	2.61E-06	Over-expressed
LIMD2	1.19	1.41E-08	1.65E-07	Over-expressed
RPS23	1.18	1.24E-20	7.46E-18	Over-expressed
RPS6	1.18	8.91E-19	2.56E-16	Over-expressed
CDT1	1.18	3.05E-08	3.24E-07	Over-expressed

IPW	1.18	5.15E-04	1.46E-03	Over-expressed
PFDN4	1.17	4.86E-21	3.08E-18	Over-expressed
RPL24	1.17	3.23E-18	7.31E-16	Over-expressed
RPS11	1.17	1.72E-17	2.77E-15	Over-expressed
RPL35A	1.17	1.75E-17	2.78E-15	Over-expressed
CMTM7	1.17	3.33E-16	3.68E-14	Over-expressed
FKBP1B	1.17	4.73E-04	1.36E-03	Over-expressed
RPL12	1.16	1.21E-18	3.17E-16	Over-expressed
TMEM91	1.16	8.25E-12	2.86E-10	Over-expressed
MIR4435-2HG	1.16	4.74E-10	9.18E-09	Over-expressed
EXOSC4	1.16	8.85E-09	1.10E-07	Over-expressed
ZP3	1.16	7.23E-08	6.74E-07	Over-expressed
PSTPIP1	1.16	1.66E-06	9.83E-06	Over-expressed
PYCARD	1.16	2.56E-06	1.43E-05	Over-expressed
ADAP1	1.16	1.34E-04	4.55E-04	Over-expressed
RAP1GAP2	1.16	3.42E-04	1.03E-03	Over-expressed
CTHRC1	1.16	1.33E-03	3.34E-03	Over-expressed
RPS7	1.15	9.75E-19	2.67E-16	Over-expressed
RPL41	1.15	1.56E-17	2.58E-15	Over-expressed
SAPCD1	1.15	1.07E-14	8.86E-13	Over-expressed
NUDT1	1.15	7.15E-11	1.86E-09	Over-expressed
LPCAT1	1.15	2.99E-09	4.37E-08	Over-expressed
S100A4	1.15	8.93E-08	8.13E-07	Over-expressed
KIF2C	1.15	8.76E-07	5.71E-06	Over-expressed
RPL4	1.14	4.02E-22	3.46E-19	Over-expressed
RPL7	1.14	3.28E-13	1.80E-11	Over-expressed
TLCD1	1.14	2.44E-11	7.48E-10	Over-expressed
MARCKSL1	1.14	2.75E-10	5.78E-09	Over-expressed
SEMA4A	1.14	3.97E-09	5.58E-08	Over-expressed
PLEKHG4	1.14	6.17E-04	1.71E-03	Over-expressed
C20ORF204	1.14	5.23E-03	0.011	Over-expressed
RACK1	1.13	4.92E-23	5.42E-20	Over-expressed
RPS18	1.13	5.15E-20	2.67E-17	Over-expressed
RPL7A	1.13	1.15E-18	3.08E-16	Over-expressed
MZT2A	1.13	2.97E-13	1.70E-11	Over-expressed
UQCRBP1	1.13	7.76E-12	2.72E-10	Over-expressed
CDCA3	1.13	5.13E-08	5.05E-07	Over-expressed
ZNF233	1.13	2.69E-07	2.08E-06	Over-expressed
SYTL1	1.13	4.54E-07	3.25E-06	Over-expressed
RPPH1	1.13	3.95E-06	2.09E-05	Over-expressed
SNRPN	1.13	6.57E-06	3.26E-05	Over-expressed
RPS15A	1.12	7.66E-17	1.01E-14	Over-expressed
IMPDH2	1.12	2.03E-15	1.88E-13	Over-expressed
GOLGA2P10	1.12	2.14E-13	1.29E-11	Over-expressed
ROMO1	1.12	3.54E-12	1.38E-10	Over-expressed
IL18	1.12	6.92E-06	3.40E-05	Over-expressed
MMP11	1.12	2.23E-05	9.43E-05	Over-expressed
CLDN4	1.12	0.0223	0.0394	Over-expressed
SNHG32	1.11	2.60E-13	1.53E-11	Over-expressed
DHRS13	1.11	2.02E-12	8.64E-11	Over-expressed
NME2P1	1.11	7.56E-12	2.66E-10	Over-expressed
IFI27L2	1.11	7.83E-07	5.18E-06	Over-expressed
YBX3	1.11	1.00E-05	4.71E-05	Over-expressed
GDF15	1.11	6.92E-05	2.53E-04	Over-expressed
S100A11	1.1	6.61E-06	3.27E-05	Over-expressed
HSPB1	1.09	8.44E-11	2.15E-09	Over-expressed
MFSD10	1.09	4.32E-09	5.98E-08	Over-expressed

SH3BP1	1.09	2.06E-07	1.66E-06	Over-expressed
JAK3	1.09	4.66E-07	3.31E-06	Over-expressed
RPS4X	1.08	8.44E-18	1.61E-15	Over-expressed
RBIS	1.08	1.68E-12	7.46E-11	Over-expressed
TPRN	1.08	3.37E-09	4.85E-08	Over-expressed
PKM	1.08	2.36E-05	9.91E-05	Over-expressed
LAG3	1.08	2.51E-04	7.83E-04	Over-expressed
CD27	1.08	5.35E-04	1.51E-03	Over-expressed
ITPA	1.07	4.62E-19	1.51E-16	Over-expressed
RPL29	1.07	2.38E-14	1.84E-12	Over-expressed
OBSL1	1.07	1.94E-05	8.34E-05	Over-expressed
NT5DC2	1.07	6.05E-05	2.26E-04	Over-expressed
CRACR2B	1.07	1.83E-04	5.97E-04	Over-expressed
RPL17	1.06	6.65E-17	9.11E-15	Over-expressed
NOP56	1.06	1.57E-14	1.26E-12	Over-expressed
GOLGA2P7	1.06	1.09E-10	2.66E-09	Over-expressed
GADD45GIP1	1.06	1.32E-10	3.11E-09	Over-expressed
ZNF90	1.06	9.72E-09	1.20E-07	Over-expressed
PAQR6	1.06	7.62E-07	5.06E-06	Over-expressed
MRNIP	1.06	6.72E-06	3.32E-05	Over-expressed
HES4	1.06	1.32E-05	5.99E-05	Over-expressed
GNLY	1.06	1.48E-05	6.60E-05	Over-expressed
RPL5	1.05	2.23E-21	1.68E-18	Over-expressed
PPP1R14B	1.05	4.31E-14	3.13E-12	Over-expressed
DPCD	1.05	1.76E-11	5.54E-10	Over-expressed
NACA2	1.05	3.80E-11	1.09E-09	Over-expressed
CMTM3	1.05	2.25E-08	2.49E-07	Over-expressed
TMEM141	1.05	5.49E-08	5.33E-07	Over-expressed
LST1	1.05	9.73E-08	8.73E-07	Over-expressed
DNLZ	1.05	2.93E-07	2.23E-06	Over-expressed
BMF	1.05	4.85E-06	2.50E-05	Over-expressed
IER3	1.05	5.54E-06	2.80E-05	Over-expressed
TM4SF1	1.05	5.95E-06	2.99E-05	Over-expressed
CHST10	1.05	2.36E-05	9.90E-05	Over-expressed
HSH2D	1.05	4.24E-05	1.66E-04	Over-expressed
FIGNL2	1.05	1.71E-04	5.61E-04	Over-expressed
SPINT2	1.05	8.54E-03	0.017	Over-expressed
RPL6	1.04	4.95E-23	5.42E-20	Over-expressed
NOP53	1.04	8.98E-16	8.94E-14	Over-expressed
RP9P	1.04	1.43E-13	9.09E-12	Over-expressed
B9D1	1.04	5.95E-09	7.87E-08	Over-expressed
UBE2S	1.04	7.67E-09	9.81E-08	Over-expressed
VEGFB	1.04	4.35E-08	4.36E-07	Over-expressed
LENG9	1.04	4.15E-07	3.01E-06	Over-expressed
DUSP2	1.04	5.03E-06	2.58E-05	Over-expressed
HID1	1.04	5.96E-06	2.99E-05	Over-expressed
EBF4	1.04	2.20E-04	6.97E-04	Over-expressed
GGT1	1.04	6.81E-04	1.87E-03	Over-expressed
RPL10A	1.03	2.82E-19	1.06E-16	Over-expressed
NAP1L1	1.03	1.62E-15	1.52E-13	Over-expressed
ZNF579	1.03	1.14E-10	2.74E-09	Over-expressed
DBNDD2	1.03	2.73E-08	2.94E-07	Over-expressed
LAPTM4B	1.03	1.00E-06	6.40E-06	Over-expressed
RPL36A	1.03	2.67E-06	1.48E-05	Over-expressed
RMRP	1.03	1.18E-05	5.43E-05	Over-expressed
MAPK12	1.03	1.59E-04	5.27E-04	Over-expressed
RPS13	1.02	2.66E-19	1.03E-16	Over-expressed

ITM2C	1.02	5.71E-11	1.54E-09	Over-expressed
RNASET2	1.02	1.01E-08	1.24E-07	Over-expressed
C11ORF80	1.02	3.29E-08	3.46E-07	Over-expressed
STK32C	1.02	3.44E-08	3.58E-07	Over-expressed
FKBP11	1.02	4.78E-07	3.39E-06	Over-expressed
JAML	1.02	1.71E-06	1.01E-05	Over-expressed
SNHG1	1.01	1.89E-12	8.25E-11	Over-expressed
TMEM256	1.01	1.46E-10	3.37E-09	Over-expressed
TOP1MT	1.01	1.71E-10	3.86E-09	Over-expressed
ATP5F1EP2	1.01	3.22E-09	4.66E-08	Over-expressed
FGFR4	1.01	1.67E-07	1.40E-06	Over-expressed
CDC25C	1.01	2.03E-05	8.68E-05	Over-expressed
MROH6	1.01	2.02E-04	6.50E-04	Over-expressed
RAB3D	1.01	3.25E-04	9.79E-04	Over-expressed
S100A9	1.01	8.43E-04	2.25E-03	Over-expressed
ELMO3	1.01	1.20E-03	3.05E-03	Over-expressed
MT1F	1.01	5.23E-03	0.011	Over-expressed
LSM2	1	9.21E-18	1.73E-15	Over-expressed
EEF1G	1	5.19E-17	7.35E-15	Over-expressed
RPS3A	1	1.28E-16	1.59E-14	Over-expressed
TAF1D	1	5.43E-16	5.74E-14	Over-expressed
NT5C	1	6.17E-14	4.28E-12	Over-expressed
UQCRB	1	2.40E-12	9.92E-11	Over-expressed
ATP5F1E	1	1.29E-11	4.25E-10	Over-expressed
COPS9	1	6.75E-11	1.76E-09	Over-expressed
LAMTOR4	1	1.41E-08	1.66E-07	Over-expressed
AP1G2	1	2.60E-08	2.82E-07	Over-expressed
MGC57346	1	4.21E-07	3.04E-06	Over-expressed
PLP2	1	1.38E-05	6.23E-05	Over-expressed
LDLRAD2	1	2.70E-05	1.12E-04	Over-expressed
TMEM51	1	4.07E-05	1.60E-04	Over-expressed
EEF1B2	0.99	1.97E-18	4.90E-16	Over-expressed
RPL19	0.99	4.19E-18	9.02E-16	Over-expressed
SRC	0.99	2.25E-07	1.79E-06	Over-expressed
BLMH	0.99	6.26E-07	4.29E-06	Over-expressed
HIST2H2AC	0.99	1.28E-06	7.86E-06	Over-expressed
NDRG1	0.99	4.64E-06	2.41E-05	Over-expressed
CAPN12	0.99	7.09E-06	3.48E-05	Over-expressed
RAD54L	0.99	2.06E-05	8.80E-05	Over-expressed
GSTP1	0.99	1.75E-04	5.73E-04	Over-expressed
NRSN2	0.99	4.09E-04	1.20E-03	Over-expressed
QSOX1	0.99	5.86E-04	1.63E-03	Over-expressed
LY6E	0.99	7.86E-04	2.12E-03	Over-expressed
FKBP10	0.99	2.25E-03	5.30E-03	Over-expressed
ITGAE	0.98	8.78E-11	2.20E-09	Over-expressed
H2AFX	0.98	1.09E-10	2.66E-09	Over-expressed
SLC52A2	0.98	6.47E-10	1.18E-08	Over-expressed
RGS10	0.98	5.74E-08	5.51E-07	Over-expressed
CD300LF	0.98	1.49E-07	1.27E-06	Over-expressed
HOMER3	0.98	4.01E-06	2.12E-05	Over-expressed
PAX8	0.98	5.99E-06	3.00E-05	Over-expressed
RIN1	0.98	7.39E-06	3.61E-05	Over-expressed
RPL39L	0.98	3.46E-03	7.70E-03	Over-expressed
TMC4	0.98	0.026	0.0453	Over-expressed
EIF4A1	0.97	1.17E-16	1.47E-14	Over-expressed
NLE1	0.97	4.49E-16	4.83E-14	Over-expressed
PTDSS2	0.97	8.54E-15	7.24E-13	Over-expressed

LBHD1	0.97	1.37E-12	6.21E-11	Over-expressed
MTHFD1L	0.97	1.05E-11	3.54E-10	Over-expressed
MZT2B	0.97	1.33E-10	3.12E-09	Over-expressed
RIPK2	0.97	3.53E-10	7.09E-09	Over-expressed
SURF2	0.97	4.76E-09	6.50E-08	Over-expressed
TRIP13	0.97	1.46E-05	6.51E-05	Over-expressed
S100A13	0.97	3.32E-05	1.34E-04	Over-expressed
TICRR	0.97	5.39E-05	2.04E-04	Over-expressed
SLC2A1	0.97	1.52E-04	5.06E-04	Over-expressed
EMG1	0.96	9.36E-18	1.74E-15	Over-expressed
RPL11	0.96	4.74E-17	6.81E-15	Over-expressed
NANOS1	0.96	1.90E-16	2.29E-14	Over-expressed
C17ORF49	0.96	2.81E-13	1.63E-11	Over-expressed
UBA52	0.96	1.62E-12	7.20E-11	Over-expressed
BOLA2	0.96	4.47E-10	8.76E-09	Over-expressed
NDUFAF8	0.96	2.02E-08	2.26E-07	Over-expressed
EME1	0.96	3.70E-07	2.72E-06	Over-expressed
SINHCAF	0.96	5.13E-06	2.63E-05	Over-expressed
CRIP1	0.96	5.51E-06	2.79E-05	Over-expressed
RENBP	0.96	1.73E-04	5.66E-04	Over-expressed
LSM7	0.95	2.88E-12	1.16E-10	Over-expressed
SEC61G	0.95	5.32E-12	1.97E-10	Over-expressed
HRAS	0.95	7.21E-12	2.56E-10	Over-expressed
CCNB1	0.95	1.50E-06	9.01E-06	Over-expressed
IL4I1	0.95	9.81E-06	4.62E-05	Over-expressed
P2RX5	0.95	1.35E-05	6.07E-05	Over-expressed
PIF1	0.95	3.85E-05	1.52E-04	Over-expressed
SNHG9	0.95	1.69E-04	5.56E-04	Over-expressed
LYRM4	0.94	8.43E-19	2.48E-16	Over-expressed
RAB24	0.94	1.12E-16	1.41E-14	Over-expressed
PFDN5	0.94	3.91E-16	4.25E-14	Over-expressed
MXD3	0.94	1.15E-13	7.41E-12	Over-expressed
POU2F1	0.94	2.18E-11	6.81E-10	Over-expressed
TAS2R20	0.94	5.68E-11	1.54E-09	Over-expressed
PABPC1	0.94	1.53E-10	3.51E-09	Over-expressed
CCDC167	0.94	3.36E-10	6.82E-09	Over-expressed
NAA38	0.94	2.00E-09	3.07E-08	Over-expressed
SOX12	0.94	2.79E-08	2.98E-07	Over-expressed
CRYGS	0.94	5.46E-08	5.31E-07	Over-expressed
TLCD5	0.94	1.62E-07	1.37E-06	Over-expressed
FAM71E1	0.94	3.62E-05	1.44E-04	Over-expressed
FCGBP	0.94	4.23E-04	1.23E-03	Over-expressed
ADAM6	0.94	0.0279	0.0482	Over-expressed
SNRPB	0.93	1.27E-15	1.22E-13	Over-expressed
NME2	0.93	7.69E-13	3.72E-11	Over-expressed
PKN1	0.93	9.01E-11	2.25E-09	Over-expressed
DDAH2	0.93	2.27E-10	4.90E-09	Over-expressed
RPL23P8	0.93	1.38E-09	2.25E-08	Over-expressed
NDUFB9	0.93	5.42E-09	7.30E-08	Over-expressed
PLEKHO1	0.93	1.51E-08	1.75E-07	Over-expressed
MIF	0.93	8.33E-07	5.45E-06	Over-expressed
CCNB2	0.93	3.82E-05	1.51E-04	Over-expressed
PTPRCAP	0.93	4.90E-05	1.88E-04	Over-expressed
PALM	0.93	2.85E-04	8.74E-04	Over-expressed
ZNF580	0.92	5.89E-10	1.09E-08	Over-expressed
UQCC3	0.92	8.84E-10	1.54E-08	Over-expressed
SUPT3H	0.92	1.38E-09	2.25E-08	Over-expressed

JPT1	0.92	2.66E-09	3.95E-08	Over-expressed
ARL2	0.92	2.92E-09	4.28E-08	Over-expressed
SMAGP	0.92	5.85E-09	7.77E-08	Over-expressed
NPIPB3	0.92	2.30E-08	2.53E-07	Over-expressed
ARHGAP39	0.92	3.64E-07	2.68E-06	Over-expressed
TRAIP	0.92	7.45E-07	4.97E-06	Over-expressed
COMTD1	0.92	2.78E-06	1.54E-05	Over-expressed
AIF1	0.92	5.12E-06	2.62E-05	Over-expressed
DNASE1	0.92	1.17E-05	5.39E-05	Over-expressed
PLTP	0.92	2.54E-04	7.90E-04	Over-expressed
PTK7	0.92	0.0179	0.0325	Over-expressed
SNRPD1	0.91	5.65E-15	5.00E-13	Over-expressed
MRPL23	0.91	4.92E-11	1.37E-09	Over-expressed
WRAP53	0.91	8.77E-11	2.20E-09	Over-expressed
TRIP10	0.91	4.46E-10	8.76E-09	Over-expressed
PUSL1	0.91	6.29E-10	1.15E-08	Over-expressed
KRT8	0.91	8.74E-10	1.53E-08	Over-expressed
PSMG3	0.91	3.99E-09	5.60E-08	Over-expressed
VPS28	0.91	7.18E-09	9.25E-08	Over-expressed
KRT18	0.91	9.03E-09	1.12E-07	Over-expressed
TONSL	0.91	1.56E-07	1.32E-06	Over-expressed
RAB3IL1	0.91	3.70E-07	2.72E-06	Over-expressed
C2ORF81	0.91	1.78E-06	1.05E-05	Over-expressed
SCAMP5	0.91	8.97E-05	3.19E-04	Over-expressed
FZD2	0.91	5.27E-04	1.49E-03	Over-expressed
TMEM200B	0.91	3.54E-03	7.85E-03	Over-expressed
RNASEK	0.9	3.01E-13	1.71E-11	Over-expressed
TOMM7	0.9	2.29E-12	9.60E-11	Over-expressed
GEMIN7	0.9	3.75E-11	1.08E-09	Over-expressed
NDUFB11	0.9	5.56E-11	1.52E-09	Over-expressed
EIF3E	0.9	1.40E-10	3.27E-09	Over-expressed
MTFP1	0.9	1.16E-09	1.95E-08	Over-expressed
STX1A	0.9	6.61E-08	6.24E-07	Over-expressed
COX6B1	0.9	9.60E-08	8.65E-07	Over-expressed
C1ORF54	0.9	3.06E-07	2.32E-06	Over-expressed
P3H4	0.9	1.83E-05	7.95E-05	Over-expressed
IMPDH1	0.9	2.43E-05	1.02E-04	Over-expressed
BCAM	0.9	3.40E-05	1.37E-04	Over-expressed
WSCD1	0.9	1.03E-04	3.59E-04	Over-expressed
STMN3	0.9	7.67E-04	2.07E-03	Over-expressed
PLA2G7	0.9	8.90E-04	2.35E-03	Over-expressed
TMEM54	0.9	2.22E-03	5.23E-03	Over-expressed
SDCBP2	0.9	2.44E-03	5.67E-03	Over-expressed
HNRNPA1	0.89	4.52E-19	1.51E-16	Over-expressed
ZNRD1	0.89	2.13E-17	3.29E-15	Over-expressed
NPM1	0.89	1.73E-12	7.63E-11	Over-expressed
YDJC	0.89	2.85E-12	1.16E-10	Over-expressed
PPP1R35	0.89	3.03E-12	1.20E-10	Over-expressed
TMEM147	0.89	1.30E-11	4.29E-10	Over-expressed
TIMM13	0.89	4.49E-09	6.18E-08	Over-expressed
PHPT1	0.89	5.19E-08	5.10E-07	Over-expressed
TTLL4	0.89	8.08E-07	5.31E-06	Over-expressed
HCST	0.89	3.42E-05	1.38E-04	Over-expressed
IER5L	0.89	3.78E-05	1.50E-04	Over-expressed
SEPTIN5	0.89	1.60E-04	5.29E-04	Over-expressed
LINGO1	0.89	7.29E-03	0.0148	Over-expressed
ACHE	0.89	8.64E-03	0.0172	Over-expressed

POLR2H	0.88	9.88E-18	1.79E-15	Over-expressed
RPL22	0.88	2.37E-17	3.61E-15	Over-expressed
NOP16	0.88	4.54E-12	1.70E-10	Over-expressed
SLC25A6	0.88	6.43E-12	2.33E-10	Over-expressed
WASH8P	0.88	8.08E-11	2.06E-09	Over-expressed
TXNDC17	0.88	2.86E-10	5.94E-09	Over-expressed
MRPL52	0.88	4.77E-09	6.51E-08	Over-expressed
TAX1BP3	0.88	1.85E-07	1.51E-06	Over-expressed
NDUFA3	0.88	6.07E-07	4.17E-06	Over-expressed
ETV2	0.88	1.33E-06	8.09E-06	Over-expressed
CDCA5	0.88	2.66E-06	1.48E-05	Over-expressed
TPM2	0.88	6.79E-06	3.35E-05	Over-expressed
CARD9	0.88	1.23E-05	5.61E-05	Over-expressed
G6PD	0.88	7.68E-05	2.78E-04	Over-expressed
HAPLN3	0.88	8.16E-05	2.93E-04	Over-expressed
CEP55	0.88	9.06E-05	3.22E-04	Over-expressed
LGALS3BP	0.88	2.20E-04	6.98E-04	Over-expressed
SLC7A1	0.88	4.22E-04	1.23E-03	Over-expressed
CDKN1C	0.88	4.32E-03	9.37E-03	Over-expressed
EGLN3	0.88	8.51E-03	0.017	Over-expressed
SLFN13	0.88	0.0101	0.0198	Over-expressed
DYNLRB1	0.87	2.09E-13	1.27E-11	Over-expressed
ZNF692	0.87	3.33E-13	1.82E-11	Over-expressed
SNRPF	0.87	5.78E-13	2.85E-11	Over-expressed
WDYHV1	0.87	9.71E-12	3.34E-10	Over-expressed
TGIF2	0.87	3.36E-10	6.82E-09	Over-expressed
RFXANK	0.87	7.08E-10	1.27E-08	Over-expressed
CCZ1P-OR7E38P	0.87	4.54E-07	3.25E-06	Over-expressed
REC8	0.87	1.88E-06	1.10E-05	Over-expressed
CHD3	0.87	3.93E-06	2.08E-05	Over-expressed
FGD3	0.87	1.32E-05	5.99E-05	Over-expressed
BAMBI	0.87	2.03E-05	8.69E-05	Over-expressed
ELF3	0.87	3.35E-04	1.01E-03	Over-expressed
GAPDH	0.86	1.32E-11	4.34E-10	Over-expressed
UQCC2	0.86	1.81E-11	5.68E-10	Over-expressed
PPCDC	0.86	3.19E-11	9.38E-10	Over-expressed
COMMD6	0.86	3.89E-11	1.11E-09	Over-expressed
ZNF444	0.86	6.45E-11	1.70E-09	Over-expressed
TMEM44	0.86	1.89E-10	4.15E-09	Over-expressed
C12ORF57	0.86	2.44E-10	5.21E-09	Over-expressed
NME1	0.86	4.52E-10	8.84E-09	Over-expressed
PLEKHJ1	0.86	4.77E-10	9.22E-09	Over-expressed
WDR34	0.86	6.12E-09	8.08E-08	Over-expressed
B9D2	0.86	2.33E-08	2.55E-07	Over-expressed
CDRT4	0.86	1.85E-07	1.51E-06	Over-expressed
BLVRA	0.86	6.97E-07	4.70E-06	Over-expressed
COX6C	0.86	1.90E-06	1.11E-05	Over-expressed
CCDC88B	0.86	3.03E-06	1.65E-05	Over-expressed
FCHSD1	0.86	5.40E-06	2.74E-05	Over-expressed
OVGP1	0.86	4.30E-05	1.68E-04	Over-expressed
NBL1	0.86	4.96E-04	1.42E-03	Over-expressed
FCGR1B	0.86	5.57E-04	1.56E-03	Over-expressed
S100A1	0.86	5.63E-03	0.0118	Over-expressed
MAL2	0.86	5.94E-03	0.0124	Over-expressed
BAIAP2L2	0.86	0.0134	0.0253	Over-expressed
RPL10	0.85	5.64E-15	5.00E-13	Over-expressed
MAPKAPK5-AS1	0.85	1.55E-13	9.70E-12	Over-expressed

TRAPPC1	0.85	1.26E-11	4.17E-10	Over-expressed
MARCKS	0.85	2.18E-09	3.32E-08	Over-expressed
NFKBIE	0.85	2.90E-09	4.25E-08	Over-expressed
CDC42EP2	0.85	5.23E-07	3.66E-06	Over-expressed
NREP	0.85	2.27E-05	9.57E-05	Over-expressed
SPC25	0.85	3.01E-05	1.23E-04	Over-expressed
PIGZ	0.85	1.45E-04	4.84E-04	Over-expressed
GDF11	0.85	3.41E-04	1.02E-03	Over-expressed
ACSS1	0.85	4.99E-04	1.42E-03	Over-expressed
CERCAM	0.85	2.36E-03	5.51E-03	Over-expressed
P3H3	0.85	8.18E-03	0.0164	Over-expressed
VWCE	0.85	0.0245	0.0429	Over-expressed
RPL15	0.84	3.28E-18	7.31E-16	Over-expressed
ATP5MC2	0.84	1.55E-17	2.58E-15	Over-expressed
PTRHD1	0.84	2.39E-14	1.84E-12	Over-expressed
HSF1	0.84	2.24E-11	6.98E-10	Over-expressed
MAF1	0.84	2.27E-11	7.02E-10	Over-expressed
EIF3H	0.84	3.53E-11	1.03E-09	Over-expressed
ENY2	0.84	1.01E-09	1.73E-08	Over-expressed
OVCA2	0.84	2.70E-09	4.00E-08	Over-expressed
SH3BGRL3	0.84	7.98E-09	1.01E-07	Over-expressed
C11ORF49	0.84	5.24E-08	5.14E-07	Over-expressed
TNFRSF4	0.84	3.22E-07	2.42E-06	Over-expressed
INAFM1	0.84	3.58E-07	2.65E-06	Over-expressed
EMILIN2	0.84	9.71E-07	6.22E-06	Over-expressed
RAD51	0.84	3.20E-05	1.29E-04	Over-expressed
CD52	0.84	3.54E-05	1.42E-04	Over-expressed
C7ORF31	0.84	5.23E-05	1.99E-04	Over-expressed
UNC13D	0.84	1.48E-04	4.93E-04	Over-expressed
CFD	0.84	1.63E-04	5.36E-04	Over-expressed
MEX3A	0.84	2.06E-04	6.59E-04	Over-expressed
SEMA3B	0.84	2.04E-03	4.85E-03	Over-expressed
GSPT2	0.84	7.37E-03	0.0149	Over-expressed
NXT1	0.83	9.81E-14	6.43E-12	Over-expressed
RPS25	0.83	3.97E-13	2.06E-11	Over-expressed
WASH5P	0.83	5.00E-13	2.55E-11	Over-expressed
IDI2	0.83	2.41E-12	9.95E-11	Over-expressed
METTL1	0.83	3.36E-12	1.32E-10	Over-expressed
EMC6	0.83	2.48E-11	7.58E-10	Over-expressed
TIGD5	0.83	5.43E-10	1.03E-08	Over-expressed
CTU1	0.83	1.23E-09	2.05E-08	Over-expressed
PGLS	0.83	3.21E-09	4.65E-08	Over-expressed
LSM8	0.83	1.08E-08	1.32E-07	Over-expressed
STAC3	0.83	3.33E-07	2.49E-06	Over-expressed
MCRIP1	0.83	1.12E-06	7.04E-06	Over-expressed
TLCD3A	0.83	7.34E-05	2.67E-04	Over-expressed
LAD1	0.83	0.019	0.0343	Over-expressed
IGF2BP3	0.83	0.0191	0.0344	Over-expressed
SNRPE	0.82	2.30E-13	1.37E-11	Over-expressed
LSM4	0.82	2.48E-11	7.58E-10	Over-expressed
EIF6	0.82	3.08E-11	9.13E-10	Over-expressed
SHARPIN	0.82	3.83E-11	1.09E-09	Over-expressed
ELOC	0.82	1.30E-10	3.08E-09	Over-expressed
EIF3K	0.82	1.68E-09	2.66E-08	Over-expressed
ATP5MD	0.82	2.05E-09	3.15E-08	Over-expressed
UBALD2	0.82	7.72E-09	9.84E-08	Over-expressed
CSKMT	0.82	1.93E-08	2.17E-07	Over-expressed

KRTCAP2	0.82	3.17E-08	3.34E-07	Over-expressed
ANO7	0.82	7.70E-07	5.10E-06	Over-expressed
CENPW	0.82	1.90E-06	1.10E-05	Over-expressed
DEF6	0.82	2.08E-06	1.19E-05	Over-expressed
TNFAIP8L2	0.82	7.59E-06	3.69E-05	Over-expressed
PLXNA3	0.82	1.34E-05	6.04E-05	Over-expressed
PACSIN3	0.82	7.66E-05	2.77E-04	Over-expressed
IKBKE	0.82	2.44E-04	7.63E-04	Over-expressed
PLK1	0.82	3.95E-04	1.16E-03	Over-expressed
ACAP1	0.82	4.17E-04	1.22E-03	Over-expressed
HSPA6	0.82	5.70E-04	1.59E-03	Over-expressed
NKG7	0.82	3.13E-03	7.07E-03	Over-expressed
GSTO2	0.82	6.75E-03	0.0139	Over-expressed
BOP1	0.82	9.58E-03	0.0188	Over-expressed
PELP1	0.81	5.42E-14	3.84E-12	Over-expressed
WASH2P	0.81	8.88E-14	5.94E-12	Over-expressed
RPP21	0.81	1.36E-12	6.21E-11	Over-expressed
PCBP4	0.81	7.21E-12	2.56E-10	Over-expressed
TFPT	0.81	1.82E-10	4.04E-09	Over-expressed
BUD31	0.81	4.24E-10	8.37E-09	Over-expressed
DPM2	0.81	1.86E-09	2.89E-08	Over-expressed
DNMT3A	0.81	3.62E-09	5.14E-08	Over-expressed
HDAC11	0.81	4.45E-09	6.14E-08	Over-expressed
BCAS4	0.81	4.61E-09	6.32E-08	Over-expressed
SAC3D1	0.81	8.89E-09	1.11E-07	Over-expressed
SPATA24	0.81	9.36E-09	1.16E-07	Over-expressed
JUND	0.81	1.25E-08	1.49E-07	Over-expressed
NDUFAF2	0.81	2.46E-08	2.68E-07	Over-expressed
SNHG11	0.81	1.49E-07	1.27E-06	Over-expressed
NDUFS6	0.81	1.65E-07	1.38E-06	Over-expressed
KIFC2	0.81	4.85E-06	2.50E-05	Over-expressed
CHKB-DT	0.81	5.61E-06	2.83E-05	Over-expressed
RAC2	0.81	2.99E-05	1.22E-04	Over-expressed
PCLAF	0.81	4.60E-05	1.77E-04	Over-expressed
MYC	0.81	4.35E-04	1.26E-03	Over-expressed
CENPV	0.81	1.12E-03	2.87E-03	Over-expressed
SV2A	0.81	3.48E-03	7.75E-03	Over-expressed
PLBD1	0.81	9.62E-03	0.0189	Over-expressed
TOMM6	0.8	2.50E-15	2.29E-13	Over-expressed
PRELID1	0.8	1.11E-12	5.13E-11	Over-expressed
EEF1A1	0.8	2.86E-12	1.16E-10	Over-expressed
NHP2	0.8	1.35E-11	4.42E-10	Over-expressed
EXOSC5	0.8	1.59E-11	5.12E-10	Over-expressed
RRP12	0.8	2.25E-11	6.99E-10	Over-expressed
PIK3R6	0.8	1.52E-06	9.09E-06	Over-expressed
SYP	0.8	7.17E-04	1.95E-03	Over-expressed
UAP1L1	0.8	1.41E-03	3.51E-03	Over-expressed
RGS2	0.8	1.72E-03	4.17E-03	Over-expressed
HKDC1	0.8	0.013	0.0245	Over-expressed
BMS1P20	0.8	0.0238	0.0419	Over-expressed
NAT9	0.79	3.48E-14	2.59E-12	Over-expressed
SNRPA	0.79	6.17E-13	3.00E-11	Over-expressed
CCDC12	0.79	1.25E-12	5.71E-11	Over-expressed
PPIH	0.79	1.04E-11	3.53E-10	Over-expressed
TOMM40	0.79	7.26E-10	1.30E-08	Over-expressed
SDHAF1	0.79	1.25E-09	2.07E-08	Over-expressed
B3GNTL1	0.79	6.66E-07	4.54E-06	Over-expressed

CD320	0.79	1.71E-06	1.01E-05	Over-expressed
SLC4A5	0.79	7.70E-06	3.74E-05	Over-expressed
ALDOA	0.79	9.71E-06	4.58E-05	Over-expressed
TNFRSF21	0.79	9.35E-05	3.31E-04	Over-expressed
FCER1G	0.79	1.15E-04	3.97E-04	Over-expressed
PTPN7	0.79	1.95E-04	6.30E-04	Over-expressed
CALHM6	0.79	1.17E-03	2.98E-03	Over-expressed
CDC25A	0.79	1.37E-03	3.42E-03	Over-expressed
SMARCD3	0.79	2.41E-03	5.62E-03	Over-expressed
MRGBP	0.78	8.20E-17	1.06E-14	Over-expressed
GABARAP	0.78	1.16E-11	3.87E-10	Over-expressed
DPH7	0.78	1.72E-11	5.45E-10	Over-expressed
PTOV1	0.78	2.37E-11	7.29E-10	Over-expressed
BAX	0.78	1.82E-10	4.04E-09	Over-expressed
CKS1B	0.78	5.37E-09	7.24E-08	Over-expressed
RNASEH2C	0.78	7.69E-09	9.81E-08	Over-expressed
NOSIP	0.78	1.17E-08	1.40E-07	Over-expressed
CPNE1	0.78	1.20E-08	1.44E-07	Over-expressed
FANCI	0.78	1.07E-07	9.42E-07	Over-expressed
SEM1	0.78	1.14E-07	1.00E-06	Over-expressed
NDUFA13	0.78	8.91E-07	5.78E-06	Over-expressed
CCDC85B	0.78	4.22E-06	2.22E-05	Over-expressed
ATP5ME	0.78	6.90E-06	3.39E-05	Over-expressed
CEBPD	0.78	8.89E-06	4.24E-05	Over-expressed
MMP24	0.78	1.41E-04	4.72E-04	Over-expressed
STK39	0.78	5.23E-04	1.48E-03	Over-expressed
CDC45	0.78	6.88E-04	1.88E-03	Over-expressed
CD6	0.78	7.50E-04	2.03E-03	Over-expressed
GZMB	0.78	3.13E-03	7.07E-03	Over-expressed
SLAMF7	0.78	4.65E-03	9.99E-03	Over-expressed
DDR1	0.78	0.0116	0.0222	Over-expressed
PDRG1	0.77	5.96E-16	6.25E-14	Over-expressed
SNRPC	0.77	5.63E-14	3.95E-12	Over-expressed
PQBP1	0.77	9.10E-13	4.30E-11	Over-expressed
DTNBP1	0.77	3.51E-11	1.02E-09	Over-expressed
MRPL14	0.77	2.49E-10	5.30E-09	Over-expressed
PPAN	0.77	1.29E-09	2.13E-08	Over-expressed
RBPJ	0.77	3.49E-09	5.01E-08	Over-expressed
ALYREF	0.77	4.62E-09	6.32E-08	Over-expressed
DTYMK	0.77	1.00E-08	1.23E-07	Over-expressed
TIAF1	0.77	1.04E-06	6.63E-06	Over-expressed
ATAD3B	0.77	3.19E-06	1.73E-05	Over-expressed
GLI4	0.77	4.16E-06	2.19E-05	Over-expressed
MRPS17	0.77	6.12E-06	3.06E-05	Over-expressed
ZNF593	0.77	9.31E-06	4.42E-05	Over-expressed
TMEM160	0.77	1.07E-05	5.00E-05	Over-expressed
TEDC1	0.77	1.12E-05	5.20E-05	Over-expressed
ST6GALNAC4	0.77	1.72E-05	7.51E-05	Over-expressed
MIR17HG	0.77	1.94E-04	6.28E-04	Over-expressed
LRRC56	0.77	2.07E-04	6.62E-04	Over-expressed
ANKRD13B	0.77	2.30E-04	7.24E-04	Over-expressed
MESP1	0.77	4.84E-04	1.39E-03	Over-expressed
SKA1	0.77	7.52E-04	2.03E-03	Over-expressed
APOBEC3D	0.77	9.07E-04	2.39E-03	Over-expressed
BMP6	0.77	1.44E-03	3.56E-03	Over-expressed
OLFML3	0.77	6.21E-03	0.0129	Over-expressed
EIF5A	0.76	2.24E-13	1.35E-11	Over-expressed

PIGU	0.76	3.43E-12	1.34E-10	Over-expressed
CDK4	0.76	1.27E-11	4.20E-10	Over-expressed
CDKN2AIPNL	0.76	5.83E-11	1.56E-09	Over-expressed
GTF3C6	0.76	5.89E-10	1.09E-08	Over-expressed
PRMT1	0.76	3.57E-09	5.10E-08	Over-expressed
POLR2I	0.76	5.68E-08	5.48E-07	Over-expressed
SPRN	0.76	7.75E-08	7.18E-07	Over-expressed
VAMP8	0.76	8.54E-08	7.81E-07	Over-expressed
MRPS12	0.76	1.62E-07	1.37E-06	Over-expressed
C20ORF27	0.76	1.90E-07	1.55E-06	Over-expressed
PAM16	0.76	1.22E-06	7.58E-06	Over-expressed
BEND3	0.76	3.42E-06	1.84E-05	Over-expressed
FAM228B	0.76	4.57E-05	1.76E-04	Over-expressed
IL2RG	0.76	9.57E-04	2.51E-03	Over-expressed
FGFR3	0.76	1.18E-03	3.00E-03	Over-expressed
BATF	0.76	4.02E-03	8.77E-03	Over-expressed
CA11	0.76	5.02E-03	0.0107	Over-expressed
SEL1L3	0.76	6.44E-03	0.0133	Over-expressed
NACA	0.75	4.80E-19	1.52E-16	Over-expressed
NACA4P	0.75	2.34E-13	1.39E-11	Over-expressed
TMEM258	0.75	1.15E-12	5.29E-11	Over-expressed
MRTO4	0.75	1.61E-10	3.67E-09	Over-expressed
TRIM28	0.75	1.84E-10	4.06E-09	Over-expressed
ATP5MPL	0.75	6.48E-09	8.48E-08	Over-expressed
SERF2	0.75	6.83E-09	8.86E-08	Over-expressed
C19ORF53	0.75	1.39E-08	1.64E-07	Over-expressed
BCL7C	0.75	8.77E-08	8.00E-07	Over-expressed
RASSF7	0.75	5.10E-06	2.61E-05	Over-expressed
ARHGAP22	0.75	1.43E-04	4.80E-04	Over-expressed
AGPAT2	0.75	2.83E-04	8.67E-04	Over-expressed
CCL3	0.75	5.70E-04	1.59E-03	Over-expressed
TBC1D10C	0.75	2.36E-03	5.51E-03	Over-expressed
IGSF3	0.75	2.40E-03	5.60E-03	Over-expressed
SLC45A4	0.75	5.14E-03	0.0109	Over-expressed
IL17RE	0.75	6.19E-03	0.0128	Over-expressed
DLG3	0.75	8.52E-03	0.017	Over-expressed
EIF3F	0.74	3.08E-16	3.43E-14	Over-expressed
NR2C2AP	0.74	3.10E-13	1.74E-11	Over-expressed
WASH7P	0.74	2.94E-12	1.17E-10	Over-expressed
TCOF1	0.74	2.31E-11	7.11E-10	Over-expressed
YWHAZ	0.74	1.12E-10	2.70E-09	Over-expressed
CKLF	0.74	3.41E-10	6.91E-09	Over-expressed
DDX39A	0.74	9.28E-10	1.60E-08	Over-expressed
MRPL13	0.74	1.69E-09	2.67E-08	Over-expressed
FHL3	0.74	1.93E-09	2.99E-08	Over-expressed
CYHR1	0.74	2.38E-09	3.59E-08	Over-expressed
BRMS1	0.74	2.88E-09	4.24E-08	Over-expressed
WDR4	0.74	3.76E-08	3.88E-07	Over-expressed
FBXL6	0.74	1.06E-07	9.40E-07	Over-expressed
ZGLP1	0.74	6.99E-07	4.71E-06	Over-expressed
TACC3	0.74	1.18E-05	5.43E-05	Over-expressed
TYROBP	0.74	1.43E-05	6.42E-05	Over-expressed
SFXN3	0.74	2.25E-05	9.50E-05	Over-expressed
AURKC	0.74	2.90E-05	1.19E-04	Over-expressed
MFSD3	0.74	1.96E-04	6.33E-04	Over-expressed
WDR62	0.74	6.92E-04	1.89E-03	Over-expressed
C1QA	0.74	1.08E-03	2.78E-03	Over-expressed

SNHG15	0.74	1.48E-03	3.65E-03	Over-expressed
CD2	0.74	2.35E-03	5.50E-03	Over-expressed
NCS1	0.74	2.53E-03	5.86E-03	Over-expressed
TIMP1	0.74	6.20E-03	0.0128	Over-expressed
EXOSC1	0.73	2.64E-16	3.00E-14	Over-expressed
EIF3B	0.73	1.36E-11	4.45E-10	Over-expressed
HIGD2A	0.73	4.46E-11	1.25E-09	Over-expressed
ARL16	0.73	1.47E-10	3.38E-09	Over-expressed
TMA7	0.73	7.74E-10	1.38E-08	Over-expressed
SCNM1	0.73	1.51E-09	2.41E-08	Over-expressed
EEF1E1	0.73	1.51E-09	2.41E-08	Over-expressed
PSENEN	0.73	4.11E-09	5.75E-08	Over-expressed
KPTN	0.73	1.10E-08	1.33E-07	Over-expressed
RASA4CP	0.73	7.86E-08	7.26E-07	Over-expressed
P2RX4	0.73	1.16E-07	1.02E-06	Over-expressed
ANXA2	0.73	3.27E-07	2.45E-06	Over-expressed
CENPP	0.73	1.54E-06	9.21E-06	Over-expressed
MICAL1	0.73	7.78E-06	3.77E-05	Over-expressed
IGFBP6	0.73	2.14E-04	6.83E-04	Over-expressed
CAPS	0.73	3.44E-04	1.03E-03	Over-expressed
TOR4A	0.73	5.64E-04	1.58E-03	Over-expressed
CST7	0.73	2.78E-03	6.37E-03	Over-expressed
CCL5	0.73	5.15E-03	0.0109	Over-expressed
NUTF2	0.72	3.68E-13	1.96E-11	Over-expressed
SNRPA1	0.72	9.64E-12	3.33E-10	Over-expressed
SFXN4	0.72	1.40E-11	4.55E-10	Over-expressed
MRPL11	0.72	2.80E-11	8.42E-10	Over-expressed
BNIP1	0.72	3.18E-11	9.36E-10	Over-expressed
SSBP4	0.72	5.76E-10	1.08E-08	Over-expressed
SNRPGP15	0.72	7.08E-10	1.27E-08	Over-expressed
MRPS24	0.72	1.01E-08	1.24E-07	Over-expressed
KLHDC4	0.72	5.57E-08	5.38E-07	Over-expressed
SF3B5	0.72	6.15E-08	5.86E-07	Over-expressed
TUBA1C	0.72	1.28E-07	1.11E-06	Over-expressed
SSR4	0.72	3.30E-07	2.47E-06	Over-expressed
ATP5MF	0.72	8.29E-07	5.43E-06	Over-expressed
LRRC61	0.72	1.16E-06	7.24E-06	Over-expressed
RRP7A	0.72	1.51E-06	9.07E-06	Over-expressed
PLEKHG2	0.72	1.65E-06	9.77E-06	Over-expressed
KCNMB3	0.72	4.31E-06	2.26E-05	Over-expressed
C17ORF53	0.72	1.82E-05	7.91E-05	Over-expressed
SMYD3	0.72	1.85E-05	8.01E-05	Over-expressed
ATP5F1D	0.72	2.77E-05	1.14E-04	Over-expressed
LY86	0.72	5.47E-05	2.07E-04	Over-expressed
PSMB10	0.72	6.44E-05	2.38E-04	Over-expressed
TRPM2	0.72	3.69E-04	1.10E-03	Over-expressed
TEDC2	0.72	5.77E-04	1.61E-03	Over-expressed
SKA3	0.72	5.86E-04	1.63E-03	Over-expressed
PLAUR	0.72	1.03E-03	2.68E-03	Over-expressed
RPP25	0.72	1.26E-03	3.17E-03	Over-expressed
CD72	0.72	3.61E-03	7.98E-03	Over-expressed
SPECC1	0.72	0.0151	0.028	Over-expressed
FCHO1	0.72	0.0211	0.0376	Over-expressed
SARNP	0.71	1.56E-14	1.26E-12	Over-expressed
ERGIC3	0.71	9.78E-14	6.43E-12	Over-expressed
NFKBIL1	0.71	1.46E-13	9.26E-12	Over-expressed
NELFE	0.71	3.69E-13	1.96E-11	Over-expressed

ATIC	0.71	9.68E-13	4.52E-11	Over-expressed
PFN1	0.71	1.52E-11	4.91E-10	Over-expressed
TMEM101	0.71	1.05E-10	2.58E-09	Over-expressed
RP9	0.71	4.68E-10	9.09E-09	Over-expressed
PDCD5	0.71	6.15E-10	1.13E-08	Over-expressed
KIAA0930	0.71	7.80E-10	1.39E-08	Over-expressed
WDR74	0.71	9.24E-10	1.60E-08	Over-expressed
TXNL4A	0.71	1.29E-09	2.13E-08	Over-expressed
PUS1	0.71	1.37E-09	2.23E-08	Over-expressed
PPIAL4G	0.71	6.90E-09	8.95E-08	Over-expressed
C8ORF44	0.71	7.74E-09	9.86E-08	Over-expressed
SPSB2	0.71	1.53E-08	1.77E-07	Over-expressed
RPSAP9	0.71	1.97E-08	2.21E-07	Over-expressed
RPF2	0.71	3.41E-08	3.55E-07	Over-expressed
PUF60	0.71	5.43E-08	5.28E-07	Over-expressed
HGH1	0.71	8.83E-08	8.05E-07	Over-expressed
BCL2L12	0.71	1.03E-07	9.15E-07	Over-expressed
ZNF775	0.71	6.50E-07	4.44E-06	Over-expressed
FXYD5	0.71	4.86E-05	1.86E-04	Over-expressed
ARHGAP4	0.71	5.28E-05	2.00E-04	Over-expressed
NCF4	0.71	7.88E-05	2.85E-04	Over-expressed
OSBPL7	0.71	1.40E-04	4.70E-04	Over-expressed
RHOH	0.71	2.30E-03	5.40E-03	Over-expressed
TRIM7	0.71	3.16E-03	7.12E-03	Over-expressed
EPOP	0.71	7.75E-03	0.0156	Over-expressed
FKBP1A	0.7	1.48E-13	9.36E-12	Over-expressed
PPIA	0.7	2.73E-12	1.11E-10	Over-expressed
PRR3	0.7	4.28E-12	1.61E-10	Over-expressed
SMIM26	0.7	9.95E-12	3.41E-10	Over-expressed
TARBP2	0.7	1.15E-11	3.84E-10	Over-expressed
DGUOK	0.7	4.48E-11	1.25E-09	Over-expressed
RRP1	0.7	4.29E-10	8.47E-09	Over-expressed
FAU	0.7	1.98E-09	3.05E-08	Over-expressed
NDUFS5	0.7	1.60E-08	1.85E-07	Over-expressed
FAM110A	0.7	4.21E-08	4.26E-07	Over-expressed
RNF181	0.7	4.97E-08	4.91E-07	Over-expressed
GOLGA6L9	0.7	9.32E-08	8.43E-07	Over-expressed
TBCB	0.7	1.13E-07	9.95E-07	Over-expressed
KRT10	0.7	1.74E-07	1.44E-06	Over-expressed
PDCD2L	0.7	1.82E-07	1.50E-06	Over-expressed
STYXL1	0.7	3.04E-07	2.30E-06	Over-expressed
EDF1	0.7	8.59E-07	5.61E-06	Over-expressed
SLIRP	0.7	1.08E-06	6.81E-06	Over-expressed
NSUN5P1	0.7	1.16E-05	5.35E-05	Over-expressed
C19ORF24	0.7	1.21E-05	5.56E-05	Over-expressed
LOC401052	0.7	2.17E-05	9.20E-05	Over-expressed
CKS2	0.7	2.65E-05	1.10E-04	Over-expressed
LAT	0.7	3.41E-05	1.37E-04	Over-expressed
PGF	0.7	6.50E-05	2.40E-04	Over-expressed
NUDT14	0.7	1.50E-04	4.99E-04	Over-expressed
ORAI2	0.7	1.83E-04	5.95E-04	Over-expressed
CCDC28B	0.7	6.25E-04	1.73E-03	Over-expressed
CCNE1	0.7	1.48E-03	3.66E-03	Over-expressed
CITED4	0.7	1.74E-03	4.21E-03	Over-expressed
PFKFB4	0.7	2.15E-03	5.10E-03	Over-expressed
TRIM16	0.7	3.75E-03	8.25E-03	Over-expressed
CGREF1	0.7	0.0156	0.0288	Over-expressed

CSNK2B	0.69	6.04E-17	8.37E-15	Over-expressed
MRPS26	0.69	1.03E-11	3.51E-10	Over-expressed
PES1	0.69	7.97E-11	2.04E-09	Over-expressed
MRPL51	0.69	6.28E-10	1.15E-08	Over-expressed
FKBPL	0.69	3.72E-09	5.27E-08	Over-expressed
NCBP2AS2	0.69	6.26E-09	8.23E-08	Over-expressed
HSPBP1	0.69	6.72E-09	8.76E-08	Over-expressed
AARSD1	0.69	7.02E-09	9.06E-08	Over-expressed
SAMD10	0.69	1.43E-08	1.67E-07	Over-expressed
RANBP1	0.69	3.14E-08	3.32E-07	Over-expressed
HYPK	0.69	3.94E-08	4.04E-07	Over-expressed
STX8	0.69	6.28E-08	5.96E-07	Over-expressed
PSMG4	0.69	1.13E-07	9.91E-07	Over-expressed
GMNN	0.69	6.75E-07	4.58E-06	Over-expressed
MDP1	0.69	7.97E-07	5.25E-06	Over-expressed
FDX2	0.69	9.02E-07	5.84E-06	Over-expressed
NEURL2	0.69	9.93E-07	6.35E-06	Over-expressed
RRS1	0.69	2.05E-06	1.18E-05	Over-expressed
DENND6B	0.69	2.45E-06	1.38E-05	Over-expressed
CYC1	0.69	2.80E-06	1.55E-05	Over-expressed
SWI5	0.69	5.20E-06	2.65E-05	Over-expressed
CENPH	0.69	5.92E-06	2.97E-05	Over-expressed
APRT	0.69	8.44E-06	4.05E-05	Over-expressed
NRM	0.69	1.03E-05	4.80E-05	Over-expressed
ADAT3	0.69	1.48E-05	6.58E-05	Over-expressed
S100A10	0.69	2.24E-05	9.48E-05	Over-expressed
MAGED1	0.69	4.20E-05	1.64E-04	Over-expressed
SHC2	0.69	8.28E-05	2.97E-04	Over-expressed
ISG20	0.69	1.61E-04	5.32E-04	Over-expressed
SMIM4	0.69	1.94E-04	6.28E-04	Over-expressed
LPCAT4	0.69	5.03E-04	1.43E-03	Over-expressed
EBI3	0.69	6.29E-04	1.73E-03	Over-expressed
ID1	0.69	7.40E-03	0.015	Over-expressed
CEACAM21	0.69	9.63E-03	0.0189	Over-expressed
SLC38A1	0.69	9.96E-03	0.0194	Over-expressed
LY96	0.69	0.0131	0.0246	Over-expressed
TPD52L2	0.68	8.75E-14	5.89E-12	Over-expressed
BTF3	0.68	2.65E-13	1.54E-11	Over-expressed
CTDNEP1	0.68	1.84E-12	8.08E-11	Over-expressed
ARFGAP1	0.68	4.81E-12	1.79E-10	Over-expressed
C12ORF73	0.68	2.86E-11	8.54E-10	Over-expressed
WASH3P	0.68	5.64E-11	1.53E-09	Over-expressed
MRPL17	0.68	8.65E-11	2.19E-09	Over-expressed
AP4M1	0.68	5.24E-10	9.98E-09	Over-expressed
SMPD2	0.68	6.89E-10	1.24E-08	Over-expressed
TRIR	0.68	2.88E-09	4.24E-08	Over-expressed
COX19	0.68	4.47E-09	6.16E-08	Over-expressed
SSNA1	0.68	8.03E-09	1.02E-07	Over-expressed
FRAT2	0.68	9.52E-09	1.18E-07	Over-expressed
ANKS3	0.68	2.47E-08	2.69E-07	Over-expressed
PSMB6	0.68	4.15E-08	4.20E-07	Over-expressed
CHCHD7	0.68	3.23E-07	2.43E-06	Over-expressed
FBXL15	0.68	3.31E-07	2.48E-06	Over-expressed
TAPT1-AS1	0.68	7.96E-07	5.24E-06	Over-expressed
UBXN11	0.68	1.25E-06	7.70E-06	Over-expressed
SRM	0.68	1.38E-06	8.39E-06	Over-expressed
HSBP1L1	0.68	4.66E-06	2.41E-05	Over-expressed

NENF	0.68	8.67E-06	4.15E-05	Over-expressed
ARHGEF2	0.68	1.84E-05	7.97E-05	Over-expressed
HILPDA	0.68	8.49E-05	3.04E-04	Over-expressed
CHMP4A	0.68	1.11E-04	3.84E-04	Over-expressed
SEMA4D	0.68	2.08E-04	6.65E-04	Over-expressed
ARHGAP9	0.68	2.38E-04	7.48E-04	Over-expressed
ZNF165	0.68	3.68E-04	1.10E-03	Over-expressed
OIP5	0.68	5.28E-04	1.50E-03	Over-expressed
TEAD2	0.68	7.05E-04	1.92E-03	Over-expressed
BCL2A1	0.68	4.13E-03	8.99E-03	Over-expressed
BEX3	0.68	8.75E-03	0.0174	Over-expressed
SKAP1	0.68	0.0121	0.0231	Over-expressed
MUSTN1	0.68	0.0139	0.0261	Over-expressed
TIMM9	0.67	4.20E-14	3.08E-12	Over-expressed
COMMD7	0.67	5.52E-13	2.76E-11	Over-expressed
UBXN1	0.67	3.94E-12	1.51E-10	Over-expressed
PRPF6	0.67	4.52E-12	1.70E-10	Over-expressed
EIF3D	0.67	7.78E-12	2.72E-10	Over-expressed
HM13	0.67	5.90E-11	1.57E-09	Over-expressed
BANF1	0.67	1.20E-10	2.86E-09	Over-expressed
UQCRH	0.67	1.35E-10	3.17E-09	Over-expressed
TMEM234	0.67	5.02E-10	9.59E-09	Over-expressed
EFNA4	0.67	8.24E-10	1.46E-08	Over-expressed
UCK2	0.67	1.90E-08	2.16E-07	Over-expressed
PTGES2	0.67	1.07E-07	9.44E-07	Over-expressed
ZNF524	0.67	5.05E-07	3.56E-06	Over-expressed
FAM207A	0.67	1.39E-06	8.41E-06	Over-expressed
MRPL27	0.67	5.16E-06	2.64E-05	Over-expressed
ACVR2B	0.67	7.77E-06	3.77E-05	Over-expressed
ELOB	0.67	9.98E-06	4.69E-05	Over-expressed
PDLIM7	0.67	1.79E-05	7.76E-05	Over-expressed
SNX22	0.67	8.57E-05	3.07E-04	Over-expressed
RNF227	0.67	1.46E-04	4.87E-04	Over-expressed
APOBEC3F	0.67	1.78E-04	5.80E-04	Over-expressed
H2AFJ	0.67	2.14E-04	6.83E-04	Over-expressed
EMP3	0.67	2.15E-04	6.83E-04	Over-expressed
CPT1B	0.67	2.96E-04	9.04E-04	Over-expressed
GBGT1	0.67	3.57E-04	1.07E-03	Over-expressed
PWWP2B	0.67	4.58E-04	1.32E-03	Over-expressed
NCAPG	0.67	6.16E-04	1.70E-03	Over-expressed
CHAF1B	0.67	6.69E-04	1.84E-03	Over-expressed
KIFC1	0.67	9.15E-04	2.41E-03	Over-expressed
CCL4L1	0.67	3.68E-03	8.10E-03	Over-expressed
P2RY6	0.67	6.92E-03	0.0142	Over-expressed
SPAG4	0.67	9.28E-03	0.0183	Over-expressed
EIF2S2	0.66	5.45E-17	7.63E-15	Over-expressed
RPAIN	0.66	1.75E-11	5.52E-10	Over-expressed
REXO4	0.66	2.86E-10	5.94E-09	Over-expressed
PRPF31	0.66	5.74E-10	1.07E-08	Over-expressed
TYSND1	0.66	1.53E-09	2.44E-08	Over-expressed
TPI1	0.66	6.05E-09	7.99E-08	Over-expressed
PFDN6	0.66	2.22E-08	2.47E-07	Over-expressed
UBE2M	0.66	2.26E-08	2.49E-07	Over-expressed
RPL3	0.66	2.78E-08	2.98E-07	Over-expressed
VOPP1	0.66	4.12E-08	4.18E-07	Over-expressed
AGTRAP	0.66	5.89E-08	5.65E-07	Over-expressed
HDDC3	0.66	5.97E-08	5.71E-07	Over-expressed

ZBTB8OS	0.66	1.69E-07	1.41E-06	Over-expressed
COMMD5	0.66	1.72E-07	1.43E-06	Over-expressed
RPS19BP1	0.66	3.37E-07	2.51E-06	Over-expressed
SCAND1	0.66	4.72E-07	3.35E-06	Over-expressed
U2AF1L4	0.66	1.24E-06	7.64E-06	Over-expressed
NDUFA11	0.66	1.31E-06	7.98E-06	Over-expressed
RRP7BP	0.66	1.53E-06	9.17E-06	Over-expressed
GSDMD	0.66	1.86E-06	1.08E-05	Over-expressed
PIM2	0.66	3.03E-06	1.66E-05	Over-expressed
METTL26	0.66	3.14E-06	1.71E-05	Over-expressed
ANAPC11	0.66	3.91E-06	2.08E-05	Over-expressed
LAGE3	0.66	9.13E-06	4.34E-05	Over-expressed
DSCC1	0.66	2.00E-05	8.57E-05	Over-expressed
TIGD1	0.66	3.22E-05	1.30E-04	Over-expressed
OSCAR	0.66	4.47E-05	1.73E-04	Over-expressed
LHFPL2	0.66	5.66E-05	2.13E-04	Over-expressed
DNAH17	0.66	4.08E-04	1.20E-03	Over-expressed
SPIRE2	0.66	6.10E-04	1.69E-03	Over-expressed
PKMYT1	0.66	1.92E-03	4.59E-03	Over-expressed
BLM	0.66	3.26E-03	7.32E-03	Over-expressed
ABCA3	0.66	6.41E-03	0.0132	Over-expressed
CARD11	0.66	7.46E-03	0.0151	Over-expressed
GZMA	0.66	8.60E-03	0.0171	Over-expressed
TET1	0.66	8.84E-03	0.0175	Over-expressed
METTL27	0.66	9.00E-03	0.0178	Over-expressed
ARPC3	0.65	4.11E-14	3.04E-12	Over-expressed
NOL7	0.65	4.21E-14	3.08E-12	Over-expressed
WDR46	0.65	2.94E-12	1.17E-10	Over-expressed
ANP32B	0.65	3.80E-11	1.09E-09	Over-expressed
MKKS	0.65	5.79E-11	1.55E-09	Over-expressed
RALY	0.65	1.32E-10	3.11E-09	Over-expressed
ZNF232	0.65	1.47E-10	3.39E-09	Over-expressed
C1QBP	0.65	1.41E-09	2.28E-08	Over-expressed
MGC16275	0.65	2.84E-09	4.18E-08	Over-expressed
SPAG7	0.65	1.68E-08	1.93E-07	Over-expressed
TCIRG1	0.65	3.89E-08	3.99E-07	Over-expressed
ZFAND2B	0.65	1.78E-07	1.47E-06	Over-expressed
LZTS2	0.65	1.83E-07	1.50E-06	Over-expressed
COA3	0.65	2.05E-07	1.65E-06	Over-expressed
ERCC1	0.65	2.38E-07	1.87E-06	Over-expressed
TRAPPC2L	0.65	1.21E-06	7.51E-06	Over-expressed
DOK1	0.65	4.32E-06	2.27E-05	Over-expressed
PSMB3	0.65	7.74E-06	3.76E-05	Over-expressed
TUBB6	0.65	2.51E-05	1.05E-04	Over-expressed
C9ORF16	0.65	4.08E-05	1.60E-04	Over-expressed
GMFG	0.65	1.15E-04	3.97E-04	Over-expressed
COTL1	0.65	1.71E-04	5.61E-04	Over-expressed
TKT	0.65	2.01E-04	6.48E-04	Over-expressed
IL1R2	0.65	8.82E-04	2.34E-03	Over-expressed
PLEKHA4	0.65	1.24E-03	3.12E-03	Over-expressed
NFE2L3	0.65	1.39E-03	3.47E-03	Over-expressed
MND1	0.65	1.57E-03	3.86E-03	Over-expressed
LINC01134	0.65	2.34E-03	5.49E-03	Over-expressed
ORC6	0.65	2.88E-03	6.57E-03	Over-expressed
FLVCR1-DT	0.65	3.56E-03	7.89E-03	Over-expressed
TGFB1	0.65	4.57E-03	9.85E-03	Over-expressed
CD8A	0.65	0.0191	0.0344	Over-expressed

SNU13	0.64	3.42E-12	1.34E-10	Over-expressed
FAM136A	0.64	5.67E-11	1.54E-09	Over-expressed
JMJD6	0.64	8.79E-11	2.20E-09	Over-expressed
STK19	0.64	4.14E-10	8.20E-09	Over-expressed
TRMT1	0.64	5.42E-10	1.03E-08	Over-expressed
SSR2	0.64	1.30E-09	2.13E-08	Over-expressed
RUVBL2	0.64	5.40E-09	7.28E-08	Over-expressed
HDDC2	0.64	6.57E-09	8.59E-08	Over-expressed
TIMM50	0.64	4.12E-08	4.18E-07	Over-expressed
NDRG3	0.64	4.86E-08	4.81E-07	Over-expressed
RBM42	0.64	5.10E-08	5.03E-07	Over-expressed
SVBP	0.64	7.78E-08	7.19E-07	Over-expressed
BAK1	0.64	2.13E-07	1.71E-06	Over-expressed
BZW2	0.64	2.30E-07	1.82E-06	Over-expressed
AHCY	0.64	3.05E-07	2.31E-06	Over-expressed
SMG9	0.64	6.71E-07	4.56E-06	Over-expressed
ANKRD13D	0.64	1.06E-06	6.71E-06	Over-expressed
SRI	0.64	2.85E-06	1.57E-05	Over-expressed
COX4I1	0.64	1.96E-05	8.44E-05	Over-expressed
CCNB1IP1	0.64	3.13E-05	1.27E-04	Over-expressed
IRF7	0.64	5.64E-05	2.13E-04	Over-expressed
PAXX	0.64	6.08E-05	2.26E-04	Over-expressed
UCP2	0.64	4.11E-04	1.20E-03	Over-expressed
DOK2	0.64	7.10E-04	1.93E-03	Over-expressed
E2F2	0.64	8.59E-04	2.29E-03	Over-expressed
ARL4C	0.64	9.14E-04	2.41E-03	Over-expressed
A2M-AS1	0.64	9.22E-04	2.43E-03	Over-expressed
TCTEX1D2	0.64	1.22E-03	3.08E-03	Over-expressed
SLC7A7	0.64	1.54E-03	3.78E-03	Over-expressed
KCNQ1OT1	0.64	2.01E-03	4.79E-03	Over-expressed
ADGRE5	0.64	2.33E-03	5.46E-03	Over-expressed
CDCA2	0.64	2.63E-03	6.07E-03	Over-expressed
DAGLA	0.64	4.24E-03	9.20E-03	Over-expressed
CPVL	0.64	9.33E-03	0.0184	Over-expressed
TPPP3	0.64	0.0102	0.0198	Over-expressed
WTIP	0.64	0.0114	0.0218	Over-expressed
PRR13	0.63	3.87E-13	2.03E-11	Over-expressed
GAR1	0.63	2.00E-11	6.26E-10	Over-expressed
RNF149	0.63	2.85E-11	8.54E-10	Over-expressed
PPP2R1A	0.63	1.10E-10	2.68E-09	Over-expressed
CLTA	0.63	2.44E-10	5.21E-09	Over-expressed
RAN	0.63	1.06E-09	1.80E-08	Over-expressed
CDK7	0.63	1.68E-09	2.66E-08	Over-expressed
TIMM22	0.63	3.37E-09	4.85E-08	Over-expressed
POP7	0.63	3.83E-09	5.40E-08	Over-expressed
MYL6	0.63	5.05E-09	6.84E-08	Over-expressed
ELP5	0.63	7.22E-09	9.30E-08	Over-expressed
PIH1D1	0.63	1.67E-08	1.91E-07	Over-expressed
SNRNP35	0.63	7.03E-08	6.58E-07	Over-expressed
TAF10	0.63	8.16E-08	7.49E-07	Over-expressed
COX7C	0.63	9.11E-08	8.27E-07	Over-expressed
CPSF4	0.63	9.63E-08	8.67E-07	Over-expressed
RPS26	0.63	1.02E-07	9.12E-07	Over-expressed
CLEC2D	0.63	1.32E-07	1.14E-06	Over-expressed
C8ORF33	0.63	1.72E-07	1.43E-06	Over-expressed
FAAP20	0.63	2.25E-07	1.78E-06	Over-expressed
TRMU	0.63	4.17E-07	3.02E-06	Over-expressed

MTG1	0.63	4.92E-07	3.48E-06	Over-expressed
ARL6IP4	0.63	5.19E-07	3.64E-06	Over-expressed
ZNRD2	0.63	1.01E-06	6.41E-06	Over-expressed
RABL6	0.63	1.06E-06	6.71E-06	Over-expressed
THAP3	0.63	1.10E-06	6.90E-06	Over-expressed
TIMM8B	0.63	1.42E-06	8.59E-06	Over-expressed
NME3	0.63	2.44E-06	1.37E-05	Over-expressed
DDIT3	0.63	4.55E-06	2.37E-05	Over-expressed
ENO1	0.63	7.95E-06	3.84E-05	Over-expressed
TBKBP1	0.63	1.76E-05	7.65E-05	Over-expressed
CST3	0.63	2.40E-05	1.01E-04	Over-expressed
RNF24	0.63	2.65E-05	1.10E-04	Over-expressed
PGP	0.63	4.91E-05	1.88E-04	Over-expressed
PYCR3	0.63	5.23E-05	1.99E-04	Over-expressed
MCM7	0.63	7.42E-05	2.70E-04	Over-expressed
PSRC1	0.63	1.29E-04	4.40E-04	Over-expressed
AGER	0.63	1.50E-04	5.00E-04	Over-expressed
C1ORF162	0.63	3.51E-04	1.05E-03	Over-expressed
FAM83H	0.63	5.14E-04	1.46E-03	Over-expressed
TSPAN15	0.63	6.54E-03	0.0134	Over-expressed
DIAPH3	0.63	8.33E-03	0.0167	Over-expressed
EMID1	0.63	0.0163	0.0299	Over-expressed
GSTM3	0.63	0.0196	0.0352	Over-expressed
POLL	0.62	7.83E-16	7.86E-14	Over-expressed
POLR1C	0.62	1.97E-12	8.49E-11	Over-expressed
C18ORF21	0.62	2.36E-12	9.80E-11	Over-expressed
FLOT1	0.62	2.48E-12	1.02E-10	Over-expressed
QARS	0.62	2.95E-12	1.17E-10	Over-expressed
SMYD5	0.62	3.97E-12	1.51E-10	Over-expressed
MANBAL	0.62	5.67E-12	2.08E-10	Over-expressed
ARFRP1	0.62	5.75E-11	1.55E-09	Over-expressed
TRMT112	0.62	6.07E-11	1.61E-09	Over-expressed
MFSD5	0.62	8.68E-11	2.19E-09	Over-expressed
CHCHD1	0.62	9.08E-11	2.26E-09	Over-expressed
TIMM17B	0.62	3.12E-10	6.40E-09	Over-expressed
COA8	0.62	1.33E-09	2.18E-08	Over-expressed
B3GALT6	0.62	1.70E-09	2.68E-08	Over-expressed
ATP6V1F	0.62	2.80E-09	4.14E-08	Over-expressed
TMEM11	0.62	3.02E-09	4.41E-08	Over-expressed
NSUN5	0.62	2.42E-08	2.64E-07	Over-expressed
SF3A2	0.62	7.43E-08	6.90E-07	Over-expressed
UBL5	0.62	1.03E-07	9.17E-07	Over-expressed
FKBP4	0.62	1.07E-07	9.45E-07	Over-expressed
SELENOH	0.62	1.61E-07	1.35E-06	Over-expressed
SMIM20	0.62	5.20E-07	3.65E-06	Over-expressed
NSMCE2	0.62	5.94E-07	4.10E-06	Over-expressed
ANAPC15	0.62	8.94E-07	5.80E-06	Over-expressed
FBXW9	0.62	1.68E-06	9.97E-06	Over-expressed
FAM41C	0.62	2.06E-06	1.18E-05	Over-expressed
TRAPPC5	0.62	8.11E-06	3.91E-05	Over-expressed
USE1	0.62	8.64E-06	4.14E-05	Over-expressed
RABAC1	0.62	1.06E-05	4.94E-05	Over-expressed
GPAA1	0.62	1.14E-05	5.28E-05	Over-expressed
ARHGEF39	0.62	2.71E-05	1.12E-04	Over-expressed
H1FX-AS1	0.62	4.30E-05	1.68E-04	Over-expressed
EIF4EBP1	0.62	1.29E-04	4.39E-04	Over-expressed
RCN1	0.62	1.35E-04	4.56E-04	Over-expressed

9-Mar	0.62	1.75E-04	5.72E-04	Over-expressed
LGALS9	0.62	1.94E-04	6.28E-04	Over-expressed
LGALS1	0.62	4.03E-04	1.18E-03	Over-expressed
PPP1R16A	0.62	4.51E-04	1.31E-03	Over-expressed
FANCD2	0.62	8.22E-04	2.20E-03	Over-expressed
CEACAM19	0.62	8.35E-04	2.23E-03	Over-expressed
TSPO	0.62	1.61E-03	3.93E-03	Over-expressed
GALNT6	0.62	1.99E-03	4.75E-03	Over-expressed
MVD	0.62	2.03E-03	4.83E-03	Over-expressed
PTPN14	0.62	3.65E-03	8.05E-03	Over-expressed
HJURP	0.62	6.77E-03	0.0139	Over-expressed
WDR97	0.62	8.68E-03	0.0173	Over-expressed
OSBP2	0.62	0.0122	0.0233	Over-expressed
KIF15	0.62	0.0264	0.0458	Over-expressed
TRIM16L	0.62	0.0285	0.0492	Over-expressed
VDAC2	0.61	3.76E-13	1.98E-11	Over-expressed
PCED1A	0.61	6.80E-12	2.45E-10	Over-expressed
CCDC58	0.61	1.11E-11	3.74E-10	Over-expressed
BYSL	0.61	1.64E-10	3.72E-09	Over-expressed
CHRAC1	0.61	1.67E-09	2.64E-08	Over-expressed
CD63	0.61	2.06E-09	3.15E-08	Over-expressed
EXOSC8	0.61	2.42E-09	3.65E-08	Over-expressed
CCDC137	0.61	4.50E-09	6.19E-08	Over-expressed
UQCRHL	0.61	7.56E-09	9.68E-08	Over-expressed
LCMT1	0.61	1.20E-08	1.44E-07	Over-expressed
EIF3G	0.61	1.43E-08	1.68E-07	Over-expressed
PTRH2	0.61	3.05E-08	3.24E-07	Over-expressed
DNPH1	0.61	4.45E-08	4.45E-07	Over-expressed
PRDX5	0.61	2.08E-07	1.67E-06	Over-expressed
TWNK	0.61	7.45E-07	4.97E-06	Over-expressed
ZNF358	0.61	1.04E-06	6.61E-06	Over-expressed
MIIP	0.61	1.16E-06	7.23E-06	Over-expressed
NT5C3B	0.61	1.45E-06	8.70E-06	Over-expressed
MRPS21	0.61	2.13E-06	1.22E-05	Over-expressed
C15ORF39	0.61	2.20E-06	1.25E-05	Over-expressed
RPL19P12	0.61	2.71E-06	1.50E-05	Over-expressed
NDUFS8	0.61	3.15E-06	1.71E-05	Over-expressed
TEN1	0.61	3.49E-06	1.87E-05	Over-expressed
DCAF13	0.61	3.70E-06	1.98E-05	Over-expressed
ARPC1B	0.61	1.42E-05	6.37E-05	Over-expressed
RDH13	0.61	2.16E-05	9.19E-05	Over-expressed
CHEK1	0.61	3.11E-05	1.26E-04	Over-expressed
MBLAC1	0.61	6.17E-05	2.29E-04	Over-expressed
NDUFB7	0.61	1.60E-04	5.29E-04	Over-expressed
RTL8A	0.61	1.99E-04	6.42E-04	Over-expressed
RAB6B	0.61	3.40E-04	1.02E-03	Over-expressed
VILL	0.61	1.39E-03	3.47E-03	Over-expressed
PRELID2	0.61	3.28E-03	7.37E-03	Over-expressed
EGFL7	0.61	3.57E-03	7.91E-03	Over-expressed
TMEM86B	0.61	7.01E-03	0.0143	Over-expressed
FABP3	0.61	8.23E-03	0.0165	Over-expressed
NUF2	0.61	8.36E-03	0.0167	Over-expressed
DLGAP5	0.61	0.013	0.0246	Over-expressed
LRFN4	0.61	0.0219	0.0388	Over-expressed
DYNLL1	0.6	2.20E-14	1.71E-12	Over-expressed
SNRPB2	0.6	9.14E-14	6.08E-12	Over-expressed
UFC1	0.6	7.33E-12	2.60E-10	Over-expressed

EIF5AL1	0.6	1.13E-10	2.72E-09	Over-expressed
ATF4	0.6	2.44E-10	5.21E-09	Over-expressed
AKAP8L	0.6	2.59E-10	5.49E-09	Over-expressed
ASB1	0.6	1.70E-09	2.67E-08	Over-expressed
MRPS18A	0.6	2.57E-09	3.83E-08	Over-expressed
THOC5	0.6	5.01E-09	6.80E-08	Over-expressed
UXT	0.6	5.45E-09	7.33E-08	Over-expressed
SLC25A19	0.6	1.11E-08	1.35E-07	Over-expressed
MED27	0.6	1.27E-08	1.51E-07	Over-expressed
NDUFB2-AS1	0.6	3.95E-08	4.04E-07	Over-expressed
SIRT6	0.6	3.99E-08	4.07E-07	Over-expressed
ZNF485	0.6	4.51E-08	4.49E-07	Over-expressed
MRPL33	0.6	5.71E-08	5.49E-07	Over-expressed
SFT2D1	0.6	9.46E-08	8.53E-07	Over-expressed
H2AFZ	0.6	1.60E-07	1.35E-06	Over-expressed
MCAT	0.6	1.77E-07	1.46E-06	Over-expressed
TATDN1	0.6	2.57E-07	1.99E-06	Over-expressed
ZMAT5	0.6	2.75E-07	2.12E-06	Over-expressed
SERGEF	0.6	3.34E-07	2.50E-06	Over-expressed
POLR2J	0.6	3.51E-07	2.61E-06	Over-expressed
OAZ1	0.6	4.43E-07	3.18E-06	Over-expressed
CEP89	0.6	5.81E-07	4.03E-06	Over-expressed
MPDU1	0.6	7.82E-07	5.17E-06	Over-expressed
INO80C	0.6	5.73E-06	2.88E-05	Over-expressed
HAUS7	0.6	6.03E-06	3.02E-05	Over-expressed
LMCD1	0.6	1.68E-05	7.38E-05	Over-expressed
TBC1D3B	0.6	3.14E-05	1.27E-04	Over-expressed
C7ORF13	0.6	5.01E-05	1.91E-04	Over-expressed
LOC728554	0.6	6.49E-05	2.39E-04	Over-expressed
RTL8C	0.6	7.68E-05	2.78E-04	Over-expressed
UQCR11	0.6	7.85E-05	2.84E-04	Over-expressed
LRRC23	0.6	1.18E-04	4.06E-04	Over-expressed
MMP25	0.6	2.07E-04	6.62E-04	Over-expressed
CCDC24	0.6	3.85E-04	1.14E-03	Over-expressed
PDE6G	0.6	6.15E-04	1.70E-03	Over-expressed
CARD16	0.6	8.38E-04	2.23E-03	Over-expressed
E2F1	0.6	1.73E-03	4.20E-03	Over-expressed
DIPK1B	0.6	2.44E-03	5.68E-03	Over-expressed
TCTN2	0.6	7.51E-03	0.0152	Over-expressed
MEAK7	0.6	0.0112	0.0216	Over-expressed
SNRPG	0.59	3.05E-13	1.73E-11	Over-expressed
PA2G4	0.59	2.20E-12	9.32E-11	Over-expressed
CHMP4B	0.59	3.98E-11	1.13E-09	Over-expressed
RBM3	0.59	5.55E-11	1.52E-09	Over-expressed
OST4	0.59	6.48E-11	1.70E-09	Over-expressed
CNPY2	0.59	9.67E-11	2.40E-09	Over-expressed
MRPL47	0.59	3.02E-10	6.22E-09	Over-expressed
CDPF1	0.59	1.85E-09	2.88E-08	Over-expressed
TTLL1	0.59	3.15E-08	3.32E-07	Over-expressed
SNF8	0.59	3.24E-07	2.44E-06	Over-expressed
MED30	0.59	4.62E-07	3.29E-06	Over-expressed
MRPL21	0.59	7.84E-07	5.18E-06	Over-expressed
CIB1	0.59	2.92E-06	1.60E-05	Over-expressed
FTH1	0.59	3.27E-06	1.77E-05	Over-expressed
GFER	0.59	6.51E-06	3.23E-05	Over-expressed
CKAP4	0.59	6.55E-06	3.25E-05	Over-expressed
NDUFA2	0.59	1.11E-05	5.15E-05	Over-expressed

TRAPPC2B	0.59	1.13E-05	5.22E-05	Over-expressed
DGAT1	0.59	1.75E-05	7.62E-05	Over-expressed
POLE4	0.59	3.98E-05	1.57E-04	Over-expressed
C7ORF50	0.59	5.83E-05	2.19E-04	Over-expressed
POLR2L	0.59	6.78E-05	2.49E-04	Over-expressed
FKBP2	0.59	7.22E-05	2.63E-04	Over-expressed
FAM118A	0.59	9.45E-05	3.34E-04	Over-expressed
QPCTL	0.59	9.81E-05	3.45E-04	Over-expressed
LMNB2	0.59	1.59E-04	5.27E-04	Over-expressed
CD300A	0.59	1.72E-04	5.64E-04	Over-expressed
HAVCR2	0.59	1.92E-03	4.60E-03	Over-expressed
VAV1	0.59	4.64E-03	9.98E-03	Over-expressed
ARMC9	0.59	6.05E-03	0.0126	Over-expressed
MYO1G	0.59	6.34E-03	0.0131	Over-expressed
BRSK1	0.59	0.0104	0.0202	Over-expressed
C15ORF40	0.58	6.13E-13	2.99E-11	Over-expressed
EHMT2	0.58	5.33E-11	1.47E-09	Over-expressed
TRIM65	0.58	1.13E-10	2.72E-09	Over-expressed
TRIM11	0.58	6.09E-10	1.12E-08	Over-expressed
URM1	0.58	2.36E-09	3.57E-08	Over-expressed
CCT3	0.58	7.00E-09	9.05E-08	Over-expressed
C1ORF35	0.58	5.36E-08	5.24E-07	Over-expressed
TSEN54	0.58	1.81E-07	1.49E-06	Over-expressed
TMEM216	0.58	2.17E-07	1.73E-06	Over-expressed
CPSF1	0.58	2.20E-07	1.75E-06	Over-expressed
HCG18	0.58	3.63E-07	2.68E-06	Over-expressed
HSCB	0.58	3.79E-07	2.77E-06	Over-expressed
NDUFS4	0.58	1.85E-06	1.08E-05	Over-expressed
JRK	0.58	2.06E-06	1.18E-05	Over-expressed
RCN3	0.58	2.14E-06	1.22E-05	Over-expressed
MRPS34	0.58	2.72E-06	1.50E-05	Over-expressed
KLF16	0.58	3.84E-06	2.04E-05	Over-expressed
NAA10	0.58	6.47E-06	3.22E-05	Over-expressed
COX5B	0.58	7.88E-06	3.81E-05	Over-expressed
CEMP1	0.58	1.08E-05	5.04E-05	Over-expressed
TMEM164	0.58	1.27E-05	5.79E-05	Over-expressed
RELB	0.58	1.32E-05	5.99E-05	Over-expressed
CHKA	0.58	1.61E-05	7.09E-05	Over-expressed
STMN1	0.58	5.63E-05	2.12E-04	Over-expressed
CDCA4	0.58	5.66E-05	2.13E-04	Over-expressed
REX1BD	0.58	8.79E-05	3.13E-04	Over-expressed
PABPC1L	0.58	2.08E-04	6.66E-04	Over-expressed
CHEK2	0.58	3.12E-04	9.47E-04	Over-expressed
ZFAND2A	0.58	3.45E-04	1.03E-03	Over-expressed
UPP1	0.58	3.78E-04	1.12E-03	Over-expressed
SFI1	0.58	4.66E-04	1.34E-03	Over-expressed
SNAI3-AS1	0.58	4.71E-04	1.35E-03	Over-expressed
IFI30	0.58	5.55E-04	1.56E-03	Over-expressed
CATSPER3	0.58	5.83E-04	1.62E-03	Over-expressed
C8ORF82	0.58	7.35E-04	1.99E-03	Over-expressed
NFATC4	0.58	3.68E-03	8.10E-03	Over-expressed
CDK1	0.58	4.24E-03	9.20E-03	Over-expressed
ETFB	0.58	4.66E-03	0.01	Over-expressed
MAP4K1	0.58	4.77E-03	0.0102	Over-expressed
CDKN3	0.58	5.95E-03	0.0124	Over-expressed
REP15	0.58	7.80E-03	0.0157	Over-expressed
HIC2	0.58	7.92E-03	0.0159	Over-expressed

CTSW	0.58	0.0283	0.0488	Over-expressed
CERS5	0.57	1.66E-11	5.31E-10	Over-expressed
ARPC4	0.57	1.59E-10	3.63E-09	Over-expressed
RBM34	0.57	2.80E-10	5.85E-09	Over-expressed
THOC6	0.57	7.85E-10	1.39E-08	Over-expressed
GPS2	0.57	1.81E-09	2.83E-08	Over-expressed
NUTM2A-AS1	0.57	5.35E-09	7.23E-08	Over-expressed
NOB1	0.57	1.31E-08	1.56E-07	Over-expressed
TRAF2	0.57	1.76E-08	2.01E-07	Over-expressed
ZNF32	0.57	1.86E-08	2.12E-07	Over-expressed
WDR83OS	0.57	2.14E-08	2.39E-07	Over-expressed
SARS2	0.57	3.69E-08	3.82E-07	Over-expressed
SMUG1	0.57	4.03E-08	4.11E-07	Over-expressed
NME6	0.57	4.10E-08	4.17E-07	Over-expressed
MRPS2	0.57	2.11E-07	1.69E-06	Over-expressed
TSSC4	0.57	2.97E-07	2.26E-06	Over-expressed
SERPINB6	0.57	6.00E-07	4.13E-06	Over-expressed
PPIB	0.57	1.09E-06	6.86E-06	Over-expressed
BORCS6	0.57	1.30E-06	7.96E-06	Over-expressed
RANGAP1	0.57	2.63E-06	1.46E-05	Over-expressed
ZNF428	0.57	2.82E-06	1.55E-05	Over-expressed
NDUFB2	0.57	9.09E-06	4.33E-05	Over-expressed
ARID5A	0.57	1.29E-05	5.86E-05	Over-expressed
KMT5C	0.57	1.41E-05	6.33E-05	Over-expressed
CHCHD5	0.57	1.42E-05	6.38E-05	Over-expressed
LYSMD4	0.57	2.16E-05	9.17E-05	Over-expressed
SOGA1	0.57	2.26E-05	9.54E-05	Over-expressed
NME4	0.57	2.90E-05	1.19E-04	Over-expressed
FBXO46	0.57	3.16E-05	1.28E-04	Over-expressed
KIF9	0.57	3.76E-05	1.49E-04	Over-expressed
CD68	0.57	6.33E-05	2.34E-04	Over-expressed
EMC9	0.57	7.27E-05	2.65E-04	Over-expressed
GNB1L	0.57	8.72E-05	3.11E-04	Over-expressed
KLHDC8B	0.57	9.07E-05	3.22E-04	Over-expressed
DYRK1B	0.57	1.20E-04	4.13E-04	Over-expressed
LOC728743	0.57	2.39E-04	7.49E-04	Over-expressed
GBAP1	0.57	5.96E-04	1.65E-03	Over-expressed
CCDC107	0.57	7.02E-04	1.92E-03	Over-expressed
DDX11	0.57	1.28E-03	3.21E-03	Over-expressed
ASMTL-AS1	0.57	1.98E-03	4.71E-03	Over-expressed
IGFLR1	0.57	2.25E-03	5.30E-03	Over-expressed
FTL	0.57	5.67E-03	0.0119	Over-expressed
TPX2	0.57	7.89E-03	0.0159	Over-expressed
OSBPL10	0.57	0.0146	0.0272	Over-expressed
SLC16A5	0.57	0.0165	0.0303	Over-expressed
CXCR3	0.57	0.0174	0.0317	Over-expressed
CSF3R	0.57	0.0199	0.0357	Over-expressed
DNTTIP1	0.56	2.33E-12	9.72E-11	Over-expressed
DNAJC7	0.56	4.77E-11	1.33E-09	Over-expressed
GNL1	0.56	1.88E-09	2.91E-08	Over-expressed
TRAPPC4	0.56	2.58E-09	3.84E-08	Over-expressed
IGBP1	0.56	2.99E-08	3.18E-07	Over-expressed
RBCK1	0.56	7.31E-08	6.80E-07	Over-expressed
NOP10	0.56	1.23E-07	1.07E-06	Over-expressed
MRPL53	0.56	3.85E-07	2.81E-06	Over-expressed
METTL16	0.56	8.85E-07	5.75E-06	Over-expressed
VASP	0.56	1.18E-06	7.31E-06	Over-expressed

MIER2	0.56	1.24E-06	7.65E-06	Over-expressed
RCC2	0.56	3.27E-06	1.76E-05	Over-expressed
DCTPP1	0.56	4.49E-06	2.34E-05	Over-expressed
LOC388955	0.56	6.63E-06	3.28E-05	Over-expressed
MFSD12	0.56	7.40E-06	3.61E-05	Over-expressed
C8ORF76	0.56	7.93E-06	3.83E-05	Over-expressed
DCLRE1C	0.56	9.93E-06	4.66E-05	Over-expressed
UQCR10	0.56	1.10E-05	5.12E-05	Over-expressed
ATP8B5P	0.56	3.15E-05	1.27E-04	Over-expressed
LOC728613	0.56	6.77E-05	2.48E-04	Over-expressed
EEF1A1P9	0.56	7.37E-05	2.68E-04	Over-expressed
YBEY	0.56	7.63E-05	2.77E-04	Over-expressed
TMSB4XP8	0.56	1.01E-04	3.54E-04	Over-expressed
NUDT18	0.56	6.05E-04	1.68E-03	Over-expressed
GNA15	0.56	8.17E-04	2.19E-03	Over-expressed
RNASE6	0.56	1.98E-03	4.72E-03	Over-expressed
GSDMB	0.56	2.78E-03	6.37E-03	Over-expressed
RMI2	0.56	3.54E-03	7.86E-03	Over-expressed
MKI67	0.56	4.66E-03	0.01	Over-expressed
GPRIN1	0.56	5.56E-03	0.0117	Over-expressed
ACP5	0.56	9.73E-03	0.0191	Over-expressed
CD3E	0.56	0.0188	0.0339	Over-expressed
CREB5	0.56	0.0189	0.0341	Over-expressed
NOP58	0.55	3.77E-10	7.52E-09	Over-expressed
EXOSC7	0.55	6.57E-10	1.19E-08	Over-expressed
RABGGTB	0.55	1.15E-09	1.93E-08	Over-expressed
MRPL22	0.55	4.18E-09	5.82E-08	Over-expressed
B4GALT2	0.55	4.52E-09	6.21E-08	Over-expressed
ZNF446	0.55	5.47E-09	7.34E-08	Over-expressed
THUMPD2	0.55	8.15E-09	1.03E-07	Over-expressed
KDELR1	0.55	9.23E-09	1.14E-07	Over-expressed
PABPC4	0.55	1.36E-08	1.60E-07	Over-expressed
ABHD12	0.55	3.37E-08	3.52E-07	Over-expressed
CNOT11	0.55	4.20E-08	4.24E-07	Over-expressed
FBXO22-AS1	0.55	5.22E-08	5.12E-07	Over-expressed
DMAP1	0.55	8.50E-08	7.78E-07	Over-expressed
R3HDM4	0.55	1.28E-07	1.11E-06	Over-expressed
STK11IP	0.55	3.58E-07	2.65E-06	Over-expressed
GET4	0.55	3.79E-07	2.77E-06	Over-expressed
ADSL	0.55	4.32E-07	3.11E-06	Over-expressed
RRP9	0.55	5.85E-07	4.04E-06	Over-expressed
HMGN1	0.55	7.99E-07	5.26E-06	Over-expressed
NANS	0.55	8.64E-07	5.64E-06	Over-expressed
IRF3	0.55	8.86E-07	5.75E-06	Over-expressed
MAP2K2	0.55	1.08E-06	6.81E-06	Over-expressed
C12ORF76	0.55	2.74E-06	1.51E-05	Over-expressed
NDUFAF4	0.55	3.54E-06	1.89E-05	Over-expressed
RPL36AL	0.55	5.54E-06	2.80E-05	Over-expressed
OXLD1	0.55	8.87E-06	4.23E-05	Over-expressed
NTMT1	0.55	1.47E-05	6.53E-05	Over-expressed
AP2S1	0.55	1.61E-05	7.10E-05	Over-expressed
PSMC1	0.55	1.99E-05	8.53E-05	Over-expressed
MRPL12	0.55	2.76E-05	1.14E-04	Over-expressed
AURKAIP1	0.55	5.99E-05	2.24E-04	Over-expressed
BRI3	0.55	6.43E-05	2.38E-04	Over-expressed
ALKBH7	0.55	3.16E-04	9.59E-04	Over-expressed
NUTM2A	0.55	3.44E-04	1.03E-03	Over-expressed

PP7080	0.55	1.25E-03	3.16E-03	Over-expressed
HES6	0.55	1.26E-03	3.19E-03	Over-expressed
TMEM243	0.55	1.94E-03	4.64E-03	Over-expressed
CERKL	0.55	2.78E-03	6.38E-03	Over-expressed
NBDY	0.55	2.90E-03	6.61E-03	Over-expressed
CCDC150	0.55	3.23E-03	7.26E-03	Over-expressed
MMAB	0.55	3.33E-03	7.46E-03	Over-expressed
CHMP4C	0.55	3.56E-03	7.89E-03	Over-expressed
APOBEC3G	0.55	5.97E-03	0.0124	Over-expressed
MC1R	0.55	7.36E-03	0.0149	Over-expressed
C1QB	0.55	0.0135	0.0255	Over-expressed
ALOX5AP	0.55	0.014	0.0262	Over-expressed
PDE4C	0.55	0.0203	0.0363	Over-expressed
ZSWIM1	0.54	2.10E-10	4.55E-09	Over-expressed
EIF2S3	0.54	2.73E-10	5.75E-09	Over-expressed
RBX1	0.54	3.79E-09	5.35E-08	Over-expressed
CHCHD2	0.54	1.58E-08	1.83E-07	Over-expressed
ITGB1BP1	0.54	1.95E-08	2.19E-07	Over-expressed
DNAJB2	0.54	2.26E-08	2.49E-07	Over-expressed
PSMD13	0.54	2.54E-08	2.76E-07	Over-expressed
SNRPD3	0.54	3.81E-08	3.92E-07	Over-expressed
TUSC2	0.54	4.29E-08	4.32E-07	Over-expressed
ORMDL2	0.54	5.09E-08	5.03E-07	Over-expressed
IMMP1L	0.54	5.71E-08	5.49E-07	Over-expressed
C15ORF61	0.54	9.26E-08	8.39E-07	Over-expressed
ACTG1	0.54	2.27E-07	1.80E-06	Over-expressed
EIF3L	0.54	2.72E-07	2.10E-06	Over-expressed
QTRT1	0.54	1.31E-06	7.99E-06	Over-expressed
COA6	0.54	3.12E-06	1.70E-05	Over-expressed
COMMD4	0.54	5.32E-06	2.71E-05	Over-expressed
TCF3	0.54	5.92E-06	2.98E-05	Over-expressed
SLC35D2	0.54	6.47E-06	3.22E-05	Over-expressed
CLTB	0.54	8.00E-06	3.86E-05	Over-expressed
ZSWIM7	0.54	8.71E-06	4.17E-05	Over-expressed
PSMB1	0.54	1.33E-05	6.00E-05	Over-expressed
NUDT2	0.54	1.87E-05	8.07E-05	Over-expressed
POLR2F	0.54	5.75E-05	2.16E-04	Over-expressed
HCFC1R1	0.54	5.94E-05	2.22E-04	Over-expressed
STAG3L4	0.54	9.97E-05	3.50E-04	Over-expressed
GTF3A	0.54	1.06E-04	3.68E-04	Over-expressed
TRAPPC6A	0.54	3.86E-04	1.14E-03	Over-expressed
DNAJC18	0.54	8.92E-04	2.36E-03	Over-expressed
UBE2T	0.54	1.18E-03	2.99E-03	Over-expressed
KIF11	0.54	2.47E-03	5.75E-03	Over-expressed
SIGLEC10	0.54	8.33E-03	0.0167	Over-expressed
NCAPH	0.54	0.0154	0.0285	Over-expressed
LCK	0.54	0.0196	0.0352	Over-expressed
EIF3M	0.53	7.16E-11	1.86E-09	Over-expressed
C12ORF10	0.53	7.28E-11	1.89E-09	Over-expressed
NIFK	0.53	3.70E-10	7.42E-09	Over-expressed
HSBP1	0.53	4.68E-10	9.09E-09	Over-expressed
SNRNP70	0.53	9.46E-10	1.63E-08	Over-expressed
POLR2G	0.53	1.50E-09	2.40E-08	Over-expressed
CCT5	0.53	1.12E-08	1.36E-07	Over-expressed
DRAP1	0.53	1.87E-08	2.13E-07	Over-expressed
KDM1A	0.53	2.52E-08	2.74E-07	Over-expressed
KLHDC3	0.53	4.28E-08	4.31E-07	Over-expressed

NABP2	0.53	5.13E-08	5.05E-07	Over-expressed
MBOAT7	0.53	7.32E-08	6.81E-07	Over-expressed
MORC2-AS1	0.53	1.47E-07	1.26E-06	Over-expressed
INTS8	0.53	3.70E-07	2.72E-06	Over-expressed
TOMM5	0.53	5.17E-07	3.63E-06	Over-expressed
DCUN1D5	0.53	5.31E-07	3.71E-06	Over-expressed
TTC28-AS1	0.53	1.26E-06	7.75E-06	Over-expressed
NARF	0.53	1.42E-06	8.59E-06	Over-expressed
TRAF4	0.53	1.46E-06	8.78E-06	Over-expressed
TUBA1B	0.53	2.19E-06	1.25E-05	Over-expressed
NT5C3A	0.53	2.20E-06	1.25E-05	Over-expressed
SUZ12P1	0.53	3.77E-06	2.01E-05	Over-expressed
TSNARE1	0.53	6.78E-06	3.34E-05	Over-expressed
RGS19	0.53	9.63E-06	4.55E-05	Over-expressed
TBX19	0.53	1.70E-05	7.44E-05	Over-expressed
C20ORF96	0.53	2.28E-05	9.60E-05	Over-expressed
TWF2	0.53	3.67E-05	1.46E-04	Over-expressed
POLD1	0.53	5.88E-05	2.20E-04	Over-expressed
NSUN5P2	0.53	6.71E-05	2.46E-04	Over-expressed
MRPL28	0.53	9.39E-05	3.32E-04	Over-expressed
CHMP2A	0.53	1.18E-04	4.05E-04	Over-expressed
FANCE	0.53	1.95E-04	6.31E-04	Over-expressed
LOC374443	0.53	2.62E-04	8.12E-04	Over-expressed
PHC1	0.53	3.99E-04	1.17E-03	Over-expressed
FGFBP3	0.53	5.17E-04	1.47E-03	Over-expressed
IL18BP	0.53	5.58E-04	1.57E-03	Over-expressed
FAM50A	0.53	8.28E-04	2.21E-03	Over-expressed
DLGAP1-AS1	0.53	1.33E-03	3.34E-03	Over-expressed
DENND2D	0.53	1.41E-03	3.51E-03	Over-expressed
LINC00926	0.53	2.26E-03	5.32E-03	Over-expressed
RRM2	0.53	3.53E-03	7.84E-03	Over-expressed
BFSP1	0.53	5.13E-03	0.0109	Over-expressed
PARVB	0.53	6.78E-03	0.0139	Over-expressed
NDC80	0.53	0.0111	0.0214	Over-expressed
BCAT1	0.53	0.0183	0.0331	Over-expressed
USP27X	0.53	0.0194	0.0349	Over-expressed
HLA-DMA	0.53	0.0221	0.0391	Over-expressed
GPBAR1	0.53	0.0222	0.0394	Over-expressed
GTSE1	0.53	0.029	0.0499	Over-expressed
MRPL43	0.52	7.84E-15	6.70E-13	Over-expressed
COPZ1	0.52	2.06E-12	8.76E-11	Over-expressed
ILKAP	0.52	8.83E-11	2.21E-09	Over-expressed
JAGN1	0.52	4.09E-09	5.73E-08	Over-expressed
SREK1IP1	0.52	1.66E-08	1.90E-07	Over-expressed
DDX56	0.52	4.32E-08	4.33E-07	Over-expressed
ZNF622	0.52	2.17E-07	1.73E-06	Over-expressed
DERL2	0.52	2.40E-07	1.88E-06	Over-expressed
BLOC1S4	0.52	2.57E-07	1.99E-06	Over-expressed
RCE1	0.52	2.85E-07	2.18E-06	Over-expressed
MYDGF	0.52	7.80E-07	5.17E-06	Over-expressed
GEMIN6	0.52	1.32E-06	8.06E-06	Over-expressed
CCDC73	0.52	1.60E-06	9.52E-06	Over-expressed
MED22	0.52	1.97E-06	1.14E-05	Over-expressed
SDHAP3	0.52	2.39E-06	1.35E-05	Over-expressed
CUTA	0.52	4.72E-06	2.44E-05	Over-expressed
LENG1	0.52	5.28E-06	2.69E-05	Over-expressed
ATP6V0B	0.52	2.37E-05	9.94E-05	Over-expressed

ARRB2	0.52	2.41E-05	1.01E-04	Over-expressed
COX14	0.52	4.98E-05	1.90E-04	Over-expressed
RAB32	0.52	6.71E-05	2.46E-04	Over-expressed
HDAC7	0.52	1.07E-04	3.70E-04	Over-expressed
CLEC4A	0.52	1.34E-04	4.54E-04	Over-expressed
CD151	0.52	1.35E-04	4.57E-04	Over-expressed
NSMCE1	0.52	1.44E-04	4.81E-04	Over-expressed
RPL23AP7	0.52	1.61E-04	5.31E-04	Over-expressed
RNASEH2A	0.52	1.61E-04	5.32E-04	Over-expressed
CDC25B	0.52	1.69E-04	5.55E-04	Over-expressed
TRIB3	0.52	4.36E-04	1.27E-03	Over-expressed
MRPL54	0.52	6.14E-04	1.70E-03	Over-expressed
ADAM8	0.52	6.29E-04	1.73E-03	Over-expressed
ZNHIT1	0.52	6.64E-04	1.83E-03	Over-expressed
CD79B	0.52	1.02E-03	2.65E-03	Over-expressed
LAPTM5	0.52	1.17E-03	2.97E-03	Over-expressed
DUSP23	0.52	1.54E-03	3.79E-03	Over-expressed
NFE2L3P2	0.52	3.07E-03	6.94E-03	Over-expressed
EIF4EBP3	0.52	3.39E-03	7.58E-03	Over-expressed
SH2B2	0.52	4.07E-03	8.88E-03	Over-expressed
CORO1A	0.52	6.54E-03	0.0134	Over-expressed
SYTL3	0.52	6.99E-03	0.0143	Over-expressed
ASF1B	0.52	7.51E-03	0.0152	Over-expressed
TMEM99	0.52	7.58E-03	0.0153	Over-expressed
TPM3P9	0.52	8.54E-03	0.017	Over-expressed
KIF18B	0.52	0.0228	0.0403	Over-expressed
SMAD5-AS1	0.52	0.0245	0.043	Over-expressed
DAZAP1	0.51	3.07E-14	2.31E-12	Over-expressed
METTL5	0.51	4.24E-11	1.20E-09	Over-expressed
MCRS1	0.51	8.66E-11	2.19E-09	Over-expressed
DDX27	0.51	2.91E-10	6.01E-09	Over-expressed
ANKZF1	0.51	1.55E-09	2.47E-08	Over-expressed
SURF6	0.51	2.17E-09	3.31E-08	Over-expressed
BOD1	0.51	2.28E-09	3.47E-08	Over-expressed
TOMM22	0.51	3.49E-09	5.00E-08	Over-expressed
TBRG4	0.51	5.60E-09	7.47E-08	Over-expressed
NDUFAF5	0.51	9.04E-09	1.12E-07	Over-expressed
PDCD11	0.51	3.80E-08	3.92E-07	Over-expressed
ENTPD6	0.51	4.36E-08	4.37E-07	Over-expressed
GPANK1	0.51	8.23E-08	7.56E-07	Over-expressed
PPA1	0.51	8.38E-08	7.68E-07	Over-expressed
KAT2A	0.51	4.38E-07	3.15E-06	Over-expressed
FLOT2	0.51	4.65E-07	3.31E-06	Over-expressed
NOC4L	0.51	7.66E-07	5.08E-06	Over-expressed
CAPNS1	0.51	8.63E-07	5.63E-06	Over-expressed
LYPLA2	0.51	1.08E-06	6.81E-06	Over-expressed
DUSP22	0.51	1.27E-06	7.80E-06	Over-expressed
HSPE1	0.51	1.39E-06	8.45E-06	Over-expressed
COPRS	0.51	2.08E-06	1.19E-05	Over-expressed
LRRC14	0.51	2.90E-06	1.59E-05	Over-expressed
BRD3OS	0.51	2.91E-06	1.60E-05	Over-expressed
TUBB	0.51	2.99E-06	1.64E-05	Over-expressed
SNHG20	0.51	3.05E-06	1.67E-05	Over-expressed
MRPS25	0.51	4.11E-06	2.17E-05	Over-expressed
CIAO2B	0.51	2.25E-05	9.50E-05	Over-expressed
TIMM10	0.51	1.65E-04	5.42E-04	Over-expressed
EID2B	0.51	3.86E-04	1.14E-03	Over-expressed

AACS	0.51	5.46E-04	1.54E-03	Over-expressed
C12ORF49	0.51	5.58E-04	1.57E-03	Over-expressed
FAM117B	0.51	1.49E-03	3.68E-03	Over-expressed
LSP1	0.51	1.76E-03	4.25E-03	Over-expressed
TP53TG1	0.51	2.35E-03	5.51E-03	Over-expressed
CATSPER2	0.51	2.82E-03	6.46E-03	Over-expressed
DSC2	0.51	5.06E-03	0.0107	Over-expressed
KCTD15	0.51	0.0244	0.0429	Over-expressed
RSL1D1	0.5	3.88E-12	1.49E-10	Over-expressed
MRPS16	0.5	7.34E-10	1.31E-08	Over-expressed
RBM4	0.5	3.58E-09	5.11E-08	Over-expressed
UBE2I	0.5	5.51E-09	7.39E-08	Over-expressed
FARSB	0.5	5.57E-09	7.44E-08	Over-expressed
EIF1	0.5	9.38E-09	1.16E-07	Over-expressed
DEDD2	0.5	1.06E-08	1.29E-07	Over-expressed
LSM3	0.5	3.28E-08	3.45E-07	Over-expressed
TSEN34	0.5	3.87E-08	3.98E-07	Over-expressed
CSE1L	0.5	6.14E-08	5.85E-07	Over-expressed
ATP5MG	0.5	6.78E-08	6.38E-07	Over-expressed
IFT20	0.5	1.23E-07	1.07E-06	Over-expressed
NSA2	0.5	1.25E-07	1.08E-06	Over-expressed
UBOX5	0.5	1.64E-07	1.38E-06	Over-expressed
MAGOHB	0.5	1.68E-07	1.40E-06	Over-expressed
TP53RK	0.5	9.06E-07	5.86E-06	Over-expressed
TRMT61A	0.5	9.52E-07	6.11E-06	Over-expressed
PHB	0.5	9.70E-07	6.22E-06	Over-expressed
SRRD	0.5	1.15E-06	7.16E-06	Over-expressed
TFAP4	0.5	1.82E-06	1.06E-05	Over-expressed
JMJD4	0.5	2.50E-06	1.40E-05	Over-expressed
PRMT2	0.5	3.16E-06	1.71E-05	Over-expressed
ZNF787	0.5	3.52E-06	1.89E-05	Over-expressed
ACD	0.5	3.77E-06	2.01E-05	Over-expressed
DYRK4	0.5	5.45E-06	2.76E-05	Over-expressed
G6PC3	0.5	9.67E-06	4.56E-05	Over-expressed
DDRGK1	0.5	1.22E-05	5.60E-05	Over-expressed
DAPK3	0.5	1.50E-05	6.64E-05	Over-expressed
PUDP	0.5	1.75E-05	7.64E-05	Over-expressed
MRM3	0.5	3.68E-05	1.46E-04	Over-expressed
ACOT8	0.5	4.37E-05	1.70E-04	Over-expressed
CCDC124	0.5	8.33E-05	2.99E-04	Over-expressed
PRR5	0.5	1.95E-04	6.31E-04	Over-expressed
NUDCD1	0.5	2.39E-04	7.48E-04	Over-expressed
BBC3	0.5	2.60E-04	8.05E-04	Over-expressed
ADCK5	0.5	3.24E-04	9.79E-04	Over-expressed
FIS1	0.5	4.80E-04	1.37E-03	Over-expressed
ATP5MC1	0.5	4.98E-04	1.42E-03	Over-expressed
MICOS13	0.5	5.53E-04	1.55E-03	Over-expressed
GPSM3	0.5	5.77E-04	1.61E-03	Over-expressed
BLOC1S1	0.5	5.83E-04	1.62E-03	Over-expressed
SRRM5	0.5	7.16E-04	1.95E-03	Over-expressed
PIGL	0.5	8.46E-04	2.25E-03	Over-expressed
ENKD1	0.5	8.76E-04	2.32E-03	Over-expressed
ARRB1	0.5	1.81E-03	4.37E-03	Over-expressed
DRAM1	0.5	2.39E-03	5.57E-03	Over-expressed
RIBC1	0.5	2.41E-03	5.63E-03	Over-expressed
TBXAS1	0.5	3.03E-03	6.88E-03	Over-expressed
P4HA1	0.5	3.42E-03	7.63E-03	Over-expressed

TNFRSF12A	0.5	5.00E-03	0.0106	Over-expressed
MSX1	0.5	6.71E-03	0.0138	Over-expressed
GPR160	0.5	6.85E-03	0.014	Over-expressed
ZNF69	0.5	9.23E-03	0.0182	Over-expressed
IGFBP3	0.5	9.93E-03	0.0194	Over-expressed
LFNG	0.5	0.0137	0.0257	Over-expressed
CD48	0.5	0.0148	0.0275	Over-expressed
RRP36	0.49	8.86E-10	1.54E-08	Over-expressed
AIMP1	0.49	2.45E-09	3.67E-08	Over-expressed
POLR1D	0.49	3.06E-09	4.46E-08	Over-expressed
RRP8	0.49	4.19E-09	5.83E-08	Over-expressed
CFL1	0.49	1.01E-08	1.24E-07	Over-expressed
POLR3C	0.49	1.34E-08	1.59E-07	Over-expressed
TMEM223	0.49	1.41E-08	1.66E-07	Over-expressed
CAPN10	0.49	4.09E-08	4.17E-07	Over-expressed
CYTH2	0.49	5.13E-08	5.05E-07	Over-expressed
UCKL1	0.49	5.63E-08	5.43E-07	Over-expressed
H3F3A	0.49	7.22E-08	6.74E-07	Over-expressed
U2AF1	0.49	1.29E-07	1.12E-06	Over-expressed
BRIX1	0.49	1.62E-07	1.36E-06	Over-expressed
NDUFS3	0.49	2.25E-07	1.79E-06	Over-expressed
PPRC1	0.49	3.63E-07	2.68E-06	Over-expressed
EIF4B	0.49	5.65E-07	3.92E-06	Over-expressed
ZNF821	0.49	7.04E-07	4.74E-06	Over-expressed
CNTROB	0.49	7.61E-07	5.06E-06	Over-expressed
LUC7L	0.49	8.27E-07	5.42E-06	Over-expressed
ZNF706	0.49	9.40E-07	6.05E-06	Over-expressed
POP5	0.49	1.06E-06	6.71E-06	Over-expressed
ZMYND19	0.49	1.13E-06	7.06E-06	Over-expressed
CD2BP2	0.49	1.23E-06	7.59E-06	Over-expressed
TBCA	0.49	1.92E-06	1.11E-05	Over-expressed
HAUS8	0.49	2.79E-06	1.54E-05	Over-expressed
DNAJC9	0.49	4.24E-06	2.23E-05	Over-expressed
SERTAD3	0.49	5.90E-06	2.97E-05	Over-expressed
PPP2R3B	0.49	9.71E-06	4.58E-05	Over-expressed
MBD3	0.49	1.15E-05	5.31E-05	Over-expressed
MRPL20	0.49	1.52E-05	6.74E-05	Over-expressed
ZNF696	0.49	2.65E-05	1.10E-04	Over-expressed
LSM5	0.49	3.03E-05	1.23E-04	Over-expressed
IFNGR2	0.49	3.48E-05	1.40E-04	Over-expressed
KIAA1522	0.49	5.41E-05	2.05E-04	Over-expressed
CSTB	0.49	9.54E-05	3.36E-04	Over-expressed
PDF	0.49	9.82E-05	3.45E-04	Over-expressed
TPT1-AS1	0.49	1.02E-04	3.55E-04	Over-expressed
LIN37	0.49	1.07E-04	3.72E-04	Over-expressed
DICER1-AS1	0.49	1.46E-04	4.87E-04	Over-expressed
MICOS10	0.49	1.52E-04	5.06E-04	Over-expressed
PRKCD	0.49	2.09E-04	6.67E-04	Over-expressed
ZNF789	0.49	3.52E-04	1.05E-03	Over-expressed
NDUFB10	0.49	5.66E-04	1.59E-03	Over-expressed
C1ORF122	0.49	8.79E-04	2.33E-03	Over-expressed
ALDH3B1	0.49	2.49E-03	5.78E-03	Over-expressed
SEPTIN1	0.49	3.07E-03	6.94E-03	Over-expressed
CCNF	0.49	3.07E-03	6.94E-03	Over-expressed
TUBB1	0.49	3.55E-03	7.88E-03	Over-expressed
CD86	0.49	3.61E-03	7.98E-03	Over-expressed
PNMA1	0.49	4.90E-03	0.0105	Over-expressed

MORN4	0.49	5.60E-03	0.0117	Over-expressed
LIN7B	0.49	6.27E-03	0.013	Over-expressed
LOC155060	0.49	6.65E-03	0.0136	Over-expressed
HSD11B2	0.49	7.61E-03	0.0154	Over-expressed
LINC02591	0.49	0.0105	0.0204	Over-expressed
N4BP3	0.49	0.0124	0.0235	Over-expressed
ZNF496	0.49	0.0133	0.025	Over-expressed
HOXB5	0.49	0.014	0.0262	Over-expressed
PSMB9	0.49	0.0184	0.0333	Over-expressed
GINS1	0.49	0.021	0.0374	Over-expressed
KIF4A	0.49	0.0221	0.0391	Over-expressed
METRNL	0.49	0.0229	0.0405	Over-expressed
RSL24D1	0.48	2.30E-13	1.37E-11	Over-expressed
ANP32A	0.48	2.78E-10	5.82E-09	Over-expressed
ZDHHC16	0.48	4.88E-10	9.38E-09	Over-expressed
ATG4B	0.48	4.97E-10	9.51E-09	Over-expressed
WDR70	0.48	1.38E-09	2.25E-08	Over-expressed
MOB2	0.48	4.34E-09	6.01E-08	Over-expressed
MRPS5	0.48	1.41E-07	1.21E-06	Over-expressed
B4GALT7	0.48	1.46E-07	1.25E-06	Over-expressed
DYNLT1	0.48	1.92E-07	1.56E-06	Over-expressed
SCO2	0.48	5.62E-07	3.90E-06	Over-expressed
ARPC5L	0.48	6.63E-07	4.52E-06	Over-expressed
BOLA3	0.48	2.00E-06	1.15E-05	Over-expressed
AIP	0.48	2.30E-06	1.30E-05	Over-expressed
ANKRD39	0.48	4.81E-06	2.48E-05	Over-expressed
CCHCR1	0.48	7.77E-06	3.77E-05	Over-expressed
BCAP31	0.48	7.88E-06	3.81E-05	Over-expressed
HINT1	0.48	8.71E-06	4.17E-05	Over-expressed
DVL2	0.48	1.46E-05	6.51E-05	Over-expressed
SMS	0.48	1.76E-05	7.65E-05	Over-expressed
COX5A	0.48	3.07E-05	1.25E-04	Over-expressed
ZCCHC3	0.48	3.30E-05	1.33E-04	Over-expressed
C1ORF50	0.48	4.81E-05	1.84E-04	Over-expressed
PSMB7	0.48	5.38E-05	2.04E-04	Over-expressed
TSR3	0.48	5.91E-05	2.21E-04	Over-expressed
GPX4	0.48	7.13E-05	2.60E-04	Over-expressed
SCRIB	0.48	7.15E-05	2.61E-04	Over-expressed
FANCG	0.48	1.46E-04	4.88E-04	Over-expressed
CCDC61	0.48	1.53E-04	5.06E-04	Over-expressed
BSG	0.48	2.04E-04	6.54E-04	Over-expressed
TTC21A	0.48	5.47E-04	1.54E-03	Over-expressed
MICA	0.48	5.68E-04	1.59E-03	Over-expressed
TSTA3	0.48	5.94E-04	1.65E-03	Over-expressed
ADA	0.48	7.02E-04	1.92E-03	Over-expressed
DTD1	0.48	9.89E-04	2.58E-03	Over-expressed
ACYP1	0.48	1.10E-03	2.82E-03	Over-expressed
KMT2E-AS1	0.48	1.19E-03	3.02E-03	Over-expressed
MANF	0.48	1.36E-03	3.41E-03	Over-expressed
ANKDD1A	0.48	1.48E-03	3.66E-03	Over-expressed
NDUFB1	0.48	1.53E-03	3.77E-03	Over-expressed
DPM3	0.48	5.21E-03	0.011	Over-expressed
UFSP1	0.48	6.63E-03	0.0136	Over-expressed
TK1	0.48	6.79E-03	0.0139	Over-expressed
OTOA	0.48	0.0124	0.0235	Over-expressed
DRC3	0.48	0.0149	0.0277	Over-expressed
MIR600HG	0.48	0.0149	0.0277	Over-expressed

TYMS	0.48	0.015	0.0279	Over-expressed
CCL4	0.48	0.0269	0.0466	Over-expressed
CCDC59	0.47	6.61E-11	1.73E-09	Over-expressed
RPP30	0.47	1.17E-10	2.81E-09	Over-expressed
C14ORF93	0.47	3.44E-09	4.95E-08	Over-expressed
TPRKB	0.47	3.59E-09	5.12E-08	Over-expressed
MED7	0.47	4.42E-09	6.11E-08	Over-expressed
OLA1	0.47	2.24E-08	2.48E-07	Over-expressed
MAVS	0.47	1.39E-07	1.20E-06	Over-expressed
RAC1	0.47	1.41E-07	1.21E-06	Over-expressed
CUTC	0.47	1.57E-07	1.33E-06	Over-expressed
HIKESHI	0.47	2.12E-07	1.70E-06	Over-expressed
TBC1D22A	0.47	2.83E-07	2.17E-06	Over-expressed
ZNF7	0.47	2.99E-07	2.27E-06	Over-expressed
FUNDC2	0.47	3.12E-07	2.36E-06	Over-expressed
ZNF16	0.47	5.82E-07	4.03E-06	Over-expressed
IMP4	0.47	8.84E-07	5.75E-06	Over-expressed
TBC1D7	0.47	1.24E-06	7.66E-06	Over-expressed
SYNGR2	0.47	1.28E-06	7.85E-06	Over-expressed
DRG2	0.47	1.59E-06	9.47E-06	Over-expressed
DHX34	0.47	1.67E-06	9.89E-06	Over-expressed
STOML2	0.47	2.30E-06	1.30E-05	Over-expressed
ATG101	0.47	2.30E-06	1.30E-05	Over-expressed
RWDD1	0.47	2.39E-06	1.35E-05	Over-expressed
DEAF1	0.47	3.71E-06	1.98E-05	Over-expressed
PMF1	0.47	4.41E-06	2.31E-05	Over-expressed
NCAPH2	0.47	1.13E-05	5.25E-05	Over-expressed
B3GAT3	0.47	1.27E-05	5.79E-05	Over-expressed
RAB5IF	0.47	2.86E-05	1.17E-04	Over-expressed
ATP5PO	0.47	3.88E-05	1.53E-04	Over-expressed
ALDH18A1	0.47	3.95E-05	1.56E-04	Over-expressed
DNAJC17	0.47	4.34E-05	1.69E-04	Over-expressed
PRAF2	0.47	5.43E-05	2.05E-04	Over-expressed
MRPS15	0.47	7.33E-05	2.67E-04	Over-expressed
ZNF589	0.47	8.36E-05	3.00E-04	Over-expressed
RPL23AP82	0.47	1.21E-04	4.13E-04	Over-expressed
GLA	0.47	1.88E-04	6.10E-04	Over-expressed
C12ORF45	0.47	2.09E-04	6.68E-04	Over-expressed
COX7A2	0.47	2.49E-04	7.78E-04	Over-expressed
SNHG10	0.47	3.20E-04	9.68E-04	Over-expressed
PMM1	0.47	4.39E-04	1.27E-03	Over-expressed
ZNF385A	0.47	5.29E-04	1.50E-03	Over-expressed
LINC00623	0.47	5.30E-04	1.50E-03	Over-expressed
WDR13	0.47	1.04E-03	2.69E-03	Over-expressed
ZNF547	0.47	1.08E-03	2.79E-03	Over-expressed
TTLL3	0.47	1.22E-03	3.08E-03	Over-expressed
IFI35	0.47	2.97E-03	6.75E-03	Over-expressed
LRRC75B	0.47	3.08E-03	6.96E-03	Over-expressed
VMO1	0.47	4.14E-03	9.01E-03	Over-expressed
GSTO1	0.47	4.42E-03	9.56E-03	Over-expressed
PIK3CD	0.47	6.58E-03	0.0135	Over-expressed
TMCC2	0.47	0.0209	0.0374	Over-expressed
GPR132	0.47	0.0213	0.0379	Over-expressed
B3GALT4	0.47	0.0233	0.0411	Over-expressed
MEST	0.47	0.0246	0.0431	Over-expressed
SAP30BP	0.46	2.81E-10	5.86E-09	Over-expressed
SYS1	0.46	1.39E-09	2.25E-08	Over-expressed

MEA1	0.46	6.76E-09	8.80E-08	Over-expressed
GTF2F2	0.46	9.18E-09	1.14E-07	Over-expressed
RAE1	0.46	1.52E-08	1.76E-07	Over-expressed
GRK6	0.46	5.37E-08	5.25E-07	Over-expressed
RHOG	0.46	6.50E-08	6.14E-07	Over-expressed
RBMX2	0.46	7.64E-08	7.08E-07	Over-expressed
THADA	0.46	9.91E-08	8.87E-07	Over-expressed
PDCL3	0.46	1.01E-07	9.06E-07	Over-expressed
TMED9	0.46	1.06E-07	9.40E-07	Over-expressed
DGKZ	0.46	1.30E-07	1.13E-06	Over-expressed
GATD1	0.46	2.87E-07	2.20E-06	Over-expressed
DDX51	0.46	6.84E-07	4.63E-06	Over-expressed
PDCD2	0.46	8.24E-07	5.41E-06	Over-expressed
GPATCH3	0.46	1.81E-06	1.06E-05	Over-expressed
C6ORF136	0.46	2.20E-06	1.25E-05	Over-expressed
CLNS1A	0.46	2.22E-06	1.26E-05	Over-expressed
FBXW4	0.46	2.62E-06	1.46E-05	Over-expressed
RPL26L1	0.46	4.25E-06	2.24E-05	Over-expressed
ZNF337	0.46	4.90E-06	2.52E-05	Over-expressed
TMEM189	0.46	5.57E-06	2.81E-05	Over-expressed
MPV17	0.46	5.60E-06	2.83E-05	Over-expressed
NOP2	0.46	6.64E-06	3.29E-05	Over-expressed
CNPY3	0.46	9.66E-06	4.56E-05	Over-expressed
RILPL1	0.46	1.67E-05	7.33E-05	Over-expressed
MRPL38	0.46	1.89E-05	8.15E-05	Over-expressed
THUMPD3-AS1	0.46	2.06E-05	8.81E-05	Over-expressed
CDC34	0.46	2.43E-05	1.02E-04	Over-expressed
PLIN3	0.46	3.01E-05	1.23E-04	Over-expressed
SLC29A3	0.46	3.91E-05	1.54E-04	Over-expressed
COPE	0.46	5.27E-05	2.00E-04	Over-expressed
STK17A	0.46	6.63E-05	2.44E-04	Over-expressed
NUDC	0.46	1.14E-04	3.95E-04	Over-expressed
UNC119	0.46	1.84E-04	6.00E-04	Over-expressed
NDUFA7	0.46	5.92E-04	1.64E-03	Over-expressed
ZNF814	0.46	6.63E-04	1.82E-03	Over-expressed
SPATA2L	0.46	7.00E-04	1.91E-03	Over-expressed
POC1A	0.46	9.75E-04	2.54E-03	Over-expressed
BIN1	0.46	1.22E-03	3.08E-03	Over-expressed
TLE3	0.46	1.23E-03	3.12E-03	Over-expressed
PHF19	0.46	1.66E-03	4.05E-03	Over-expressed
MSH5	0.46	2.04E-03	4.85E-03	Over-expressed
TRAF5	0.46	2.06E-03	4.89E-03	Over-expressed
DNAH14	0.46	2.26E-03	5.31E-03	Over-expressed
UBE3D	0.46	2.80E-03	6.42E-03	Over-expressed
ZNF530	0.46	3.03E-03	6.87E-03	Over-expressed
GINS2	0.46	3.19E-03	7.19E-03	Over-expressed
JOSD2	0.46	3.27E-03	7.34E-03	Over-expressed
AURKA	0.46	3.57E-03	7.90E-03	Over-expressed
GUCA1B	0.46	6.13E-03	0.0127	Over-expressed
STX3	0.46	6.19E-03	0.0128	Over-expressed
MPST	0.46	7.19E-03	0.0146	Over-expressed
EFNA1	0.46	8.42E-03	0.0168	Over-expressed
SYK	0.46	0.0103	0.0201	Over-expressed
TRPV2	0.46	0.0105	0.0204	Over-expressed
RNF144A	0.46	0.0123	0.0235	Over-expressed
GRAMD1A	0.46	0.0162	0.0298	Over-expressed
SLAMF8	0.46	0.017	0.0311	Over-expressed

RARS	0.45	1.89E-10	4.15E-09	Over-expressed
EIF2B4	0.45	2.18E-09	3.32E-08	Over-expressed
SLC25A3	0.45	1.90E-08	2.15E-07	Over-expressed
EIF4E2	0.45	1.92E-08	2.16E-07	Over-expressed
AATF	0.45	3.68E-08	3.81E-07	Over-expressed
MRPL9	0.45	5.40E-08	5.26E-07	Over-expressed
GTF3C5	0.45	8.96E-08	8.15E-07	Over-expressed
SNX5	0.45	9.15E-08	8.30E-07	Over-expressed
ZNF394	0.45	1.12E-07	9.83E-07	Over-expressed
OTUB1	0.45	1.14E-07	1.00E-06	Over-expressed
IDH3B	0.45	1.67E-07	1.40E-06	Over-expressed
SSBP1	0.45	1.75E-07	1.45E-06	Over-expressed
LETMD1	0.45	2.75E-07	2.12E-06	Over-expressed
MRPL48	0.45	2.98E-07	2.27E-06	Over-expressed
CCT4	0.45	3.77E-07	2.76E-06	Over-expressed
DRG1	0.45	3.98E-07	2.90E-06	Over-expressed
KARS	0.45	9.13E-07	5.90E-06	Over-expressed
PNKP	0.45	1.89E-06	1.10E-05	Over-expressed
ZNF133	0.45	1.97E-06	1.14E-05	Over-expressed
PSMA7	0.45	2.69E-06	1.49E-05	Over-expressed
RNF113A	0.45	2.86E-06	1.58E-05	Over-expressed
TPT1	0.45	4.69E-06	2.43E-05	Over-expressed
CNIH4	0.45	5.42E-06	2.75E-05	Over-expressed
NDUFB8	0.45	5.67E-06	2.86E-05	Over-expressed
CHST12	0.45	1.07E-05	4.98E-05	Over-expressed
COPS6	0.45	1.14E-05	5.25E-05	Over-expressed
WDR18	0.45	1.20E-05	5.50E-05	Over-expressed
NPEPL1	0.45	1.28E-05	5.84E-05	Over-expressed
ZNF251	0.45	1.73E-05	7.55E-05	Over-expressed
MIDN	0.45	2.38E-05	9.98E-05	Over-expressed
NOL12	0.45	2.76E-05	1.14E-04	Over-expressed
CIDECP1	0.45	3.45E-05	1.39E-04	Over-expressed
MTX1	0.45	3.63E-05	1.45E-04	Over-expressed
JTB	0.45	3.79E-05	1.50E-04	Over-expressed
NPC2	0.45	6.99E-05	2.55E-04	Over-expressed
SERPINB1	0.45	8.08E-05	2.91E-04	Over-expressed
PSMD4	0.45	1.33E-04	4.52E-04	Over-expressed
CNTD1	0.45	2.16E-04	6.88E-04	Over-expressed
CBX8	0.45	2.18E-04	6.92E-04	Over-expressed
RAVER1	0.45	2.57E-04	7.99E-04	Over-expressed
SPATA33	0.45	2.91E-04	8.90E-04	Over-expressed
EXT1	0.45	3.74E-04	1.11E-03	Over-expressed
C19ORF25	0.45	4.32E-04	1.26E-03	Over-expressed
TSSC2	0.45	7.98E-04	2.14E-03	Over-expressed
LOC642846	0.45	8.29E-04	2.21E-03	Over-expressed
MYBBP1A	0.45	9.59E-04	2.51E-03	Over-expressed
MRPL24	0.45	1.63E-03	3.98E-03	Over-expressed
TMEM219	0.45	1.83E-03	4.40E-03	Over-expressed
DOK3	0.45	1.87E-03	4.47E-03	Over-expressed
PPT2	0.45	2.29E-03	5.36E-03	Over-expressed
TRIP6	0.45	2.67E-03	6.15E-03	Over-expressed
HSD11B1L	0.45	3.18E-03	7.17E-03	Over-expressed
CSPP1	0.45	3.56E-03	7.88E-03	Over-expressed
MAN1B1-DT	0.45	4.74E-03	0.0102	Over-expressed
GMDS	0.45	5.90E-03	0.0123	Over-expressed
ATOX1	0.45	7.13E-03	0.0145	Over-expressed
ENDOG	0.45	8.73E-03	0.0174	Over-expressed

TMEM198	0.45	0.012	0.0229	Over-expressed
MINDY4	0.45	0.0148	0.0275	Over-expressed
CCDC102A	0.45	0.0196	0.0352	Over-expressed
EXO1	0.45	0.0255	0.0445	Over-expressed
UBE2V1	0.44	5.82E-11	1.56E-09	Over-expressed
NFU1	0.44	1.18E-09	1.98E-08	Over-expressed
CWF19L1	0.44	1.29E-09	2.13E-08	Over-expressed
SRSF9	0.44	5.90E-09	7.82E-08	Over-expressed
HTRA2	0.44	2.69E-08	2.90E-07	Over-expressed
DCTN2	0.44	3.08E-08	3.26E-07	Over-expressed
NEDD8	0.44	3.85E-08	3.96E-07	Over-expressed
LAMTOR5	0.44	1.23E-07	1.07E-06	Over-expressed
RTRAF	0.44	1.44E-07	1.23E-06	Over-expressed
PPIE	0.44	2.05E-07	1.65E-06	Over-expressed
HDGFL2	0.44	3.34E-07	2.49E-06	Over-expressed
USF1	0.44	4.92E-07	3.48E-06	Over-expressed
BRD9	0.44	5.99E-07	4.13E-06	Over-expressed
GLRX3	0.44	1.36E-06	8.29E-06	Over-expressed
DNAJC2	0.44	1.80E-06	1.05E-05	Over-expressed
MOB3A	0.44	6.91E-06	3.40E-05	Over-expressed
C1ORF159	0.44	7.24E-06	3.54E-05	Over-expressed
THG1L	0.44	8.95E-06	4.26E-05	Over-expressed
PFDN2	0.44	1.14E-05	5.28E-05	Over-expressed
ZSCAN16	0.44	5.39E-05	2.04E-04	Over-expressed
GPATCH4	0.44	6.07E-05	2.26E-04	Over-expressed
PRRT3	0.44	1.03E-04	3.61E-04	Over-expressed
PIN4	0.44	1.04E-04	3.64E-04	Over-expressed
PHETA1	0.44	1.16E-04	4.00E-04	Over-expressed
LMNA	0.44	1.23E-04	4.22E-04	Over-expressed
COX6A1	0.44	1.53E-04	5.07E-04	Over-expressed
TUBB4B	0.44	4.05E-04	1.19E-03	Over-expressed
MSRB2	0.44	8.85E-04	2.34E-03	Over-expressed
CCDC134	0.44	9.56E-04	2.51E-03	Over-expressed
CTDSPL	0.44	1.17E-03	2.98E-03	Over-expressed
SULT1A3	0.44	2.40E-03	5.61E-03	Over-expressed
CCDC34	0.44	2.42E-03	5.65E-03	Over-expressed
FERMT3	0.44	4.11E-03	8.95E-03	Over-expressed
CDC42EP1	0.44	4.11E-03	8.96E-03	Over-expressed
ATP1B3	0.44	4.71E-03	0.0101	Over-expressed
MRPL55	0.44	5.15E-03	0.0109	Over-expressed
LY6G5C	0.44	7.35E-03	0.0149	Over-expressed
UBE2E2	0.44	7.70E-03	0.0155	Over-expressed
CYTH4	0.44	8.93E-03	0.0177	Over-expressed
ANXA2R	0.44	0.0112	0.0215	Over-expressed
ABHD11	0.44	0.0114	0.0218	Over-expressed
HSD17B1	0.44	0.0147	0.0273	Over-expressed
TP53	0.44	0.0207	0.0369	Over-expressed
POU2F2	0.44	0.0241	0.0423	Over-expressed
LAT2	0.44	0.0247	0.0432	Over-expressed
ZNF526	0.43	8.22E-09	1.04E-07	Over-expressed
KXD1	0.43	3.33E-08	3.49E-07	Over-expressed
PRKRIP1	0.43	8.31E-08	7.62E-07	Over-expressed
YWHAE	0.43	9.30E-08	8.42E-07	Over-expressed
ZNF511	0.43	1.37E-07	1.18E-06	Over-expressed
SDHAF2	0.43	1.67E-07	1.40E-06	Over-expressed
VPS72	0.43	4.42E-07	3.17E-06	Over-expressed
PBDC1	0.43	4.99E-07	3.52E-06	Over-expressed

GRWD1	0.43	8.97E-07	5.81E-06	Over-expressed
PSMA4	0.43	1.17E-06	7.30E-06	Over-expressed
CAMLG	0.43	1.18E-06	7.31E-06	Over-expressed
EIPR1	0.43	2.45E-06	1.38E-05	Over-expressed
DHX37	0.43	2.70E-06	1.49E-05	Over-expressed
CUEDC2	0.43	3.05E-06	1.67E-05	Over-expressed
SENP3	0.43	3.13E-06	1.70E-05	Over-expressed
ARF5	0.43	3.88E-06	2.06E-05	Over-expressed
ZFPL1	0.43	5.02E-06	2.58E-05	Over-expressed
SERF1A	0.43	8.33E-06	4.00E-05	Over-expressed
YBX1	0.43	9.87E-06	4.64E-05	Over-expressed
POLR2K	0.43	1.55E-05	6.84E-05	Over-expressed
CLASRP	0.43	1.73E-05	7.55E-05	Over-expressed
FUNDC1	0.43	1.88E-05	8.10E-05	Over-expressed
SAAL1	0.43	2.40E-05	1.01E-04	Over-expressed
XPO5	0.43	3.12E-05	1.27E-04	Over-expressed
VARS	0.43	4.53E-05	1.75E-04	Over-expressed
ARL3	0.43	4.92E-05	1.88E-04	Over-expressed
MALSU1	0.43	5.01E-05	1.91E-04	Over-expressed
GEMIN4	0.43	6.04E-05	2.25E-04	Over-expressed
CD276	0.43	6.15E-05	2.29E-04	Over-expressed
AKT1S1	0.43	8.55E-05	3.06E-04	Over-expressed
PPIL1	0.43	1.62E-04	5.34E-04	Over-expressed
MORN2	0.43	1.83E-04	5.97E-04	Over-expressed
LYAR	0.43	1.96E-04	6.32E-04	Over-expressed
PACS1	0.43	1.99E-04	6.42E-04	Over-expressed
CHCHD6	0.43	2.31E-04	7.26E-04	Over-expressed
NAXE	0.43	2.52E-04	7.84E-04	Over-expressed
AIFM2	0.43	3.18E-04	9.64E-04	Over-expressed
EGLN2	0.43	3.35E-04	1.01E-03	Over-expressed
DNAJC4	0.43	3.88E-04	1.14E-03	Over-expressed
CACFD1	0.43	5.40E-04	1.52E-03	Over-expressed
SHLD1	0.43	5.51E-04	1.55E-03	Over-expressed
VAT1	0.43	6.89E-04	1.88E-03	Over-expressed
FAM49B	0.43	1.06E-03	2.74E-03	Over-expressed
P2RY11	0.43	1.52E-03	3.73E-03	Over-expressed
ABHD16B	0.43	1.57E-03	3.84E-03	Over-expressed
TSPAN4	0.43	2.37E-03	5.53E-03	Over-expressed
PDE7A	0.43	2.48E-03	5.77E-03	Over-expressed
PSME2	0.43	3.72E-03	8.18E-03	Over-expressed
C17ORF100	0.43	3.79E-03	8.32E-03	Over-expressed
GPD1L	0.43	7.09E-03	0.0144	Over-expressed
SLC26A6	0.43	0.0171	0.0312	Over-expressed
PPARG	0.43	0.0199	0.0357	Over-expressed
MATK	0.43	0.0226	0.0399	Over-expressed
ATP2A3	0.43	0.0268	0.0465	Over-expressed
RBM22	0.42	1.02E-10	2.51E-09	Over-expressed
PABPN1	0.42	1.44E-10	3.34E-09	Over-expressed
SLC35C2	0.42	1.48E-09	2.37E-08	Over-expressed
SUMO2	0.42	2.45E-09	3.67E-08	Over-expressed
CBX3	0.42	3.79E-09	5.36E-08	Over-expressed
DIABLO	0.42	5.52E-08	5.35E-07	Over-expressed
MRPS30	0.42	2.84E-07	2.17E-06	Over-expressed
ADPRHL2	0.42	3.74E-07	2.74E-06	Over-expressed
SEC11A	0.42	5.21E-07	3.65E-06	Over-expressed
DUSP12	0.42	5.42E-07	3.78E-06	Over-expressed
IMP3	0.42	9.41E-07	6.06E-06	Over-expressed

MRPS23	0.42	9.63E-07	6.18E-06	Over-expressed
MED10	0.42	1.36E-06	8.26E-06	Over-expressed
TMEM9	0.42	1.76E-06	1.04E-05	Over-expressed
CCT7	0.42	1.99E-06	1.15E-05	Over-expressed
ING5	0.42	2.33E-06	1.32E-05	Over-expressed
DOHH	0.42	2.48E-06	1.39E-05	Over-expressed
GARS	0.42	2.80E-06	1.55E-05	Over-expressed
PSMB4	0.42	4.85E-06	2.50E-05	Over-expressed
COA4	0.42	6.22E-06	3.10E-05	Over-expressed
RAD9A	0.42	7.57E-06	3.68E-05	Over-expressed
DCTN5	0.42	8.43E-06	4.05E-05	Over-expressed
MRPL2	0.42	1.01E-05	4.73E-05	Over-expressed
BLOC1S3	0.42	1.16E-05	5.34E-05	Over-expressed
PSMC5	0.42	1.19E-05	5.47E-05	Over-expressed
IRGQ	0.42	1.21E-05	5.56E-05	Over-expressed
PPIAL4C	0.42	1.85E-05	8.02E-05	Over-expressed
EXOSC6	0.42	2.45E-05	1.02E-04	Over-expressed
NUP37	0.42	3.30E-05	1.33E-04	Over-expressed
MYPOP	0.42	3.40E-05	1.37E-04	Over-expressed
MLST8	0.42	4.35E-05	1.69E-04	Over-expressed
EIF3CL	0.42	4.48E-05	1.73E-04	Over-expressed
CDK16	0.42	4.62E-05	1.78E-04	Over-expressed
DDX49	0.42	7.31E-05	2.66E-04	Over-expressed
SYMPK	0.42	1.05E-04	3.65E-04	Over-expressed
MFNG	0.42	1.90E-04	6.16E-04	Over-expressed
METTL9	0.42	1.99E-04	6.41E-04	Over-expressed
MAPRE1	0.42	2.21E-04	7.00E-04	Over-expressed
RFC4	0.42	3.24E-04	9.77E-04	Over-expressed
FTH1P3	0.42	5.48E-04	1.54E-03	Over-expressed
PMS2P3	0.42	5.76E-04	1.61E-03	Over-expressed
LIPT2	0.42	5.78E-04	1.61E-03	Over-expressed
TAGLN2	0.42	7.76E-04	2.09E-03	Over-expressed
ABCA11P	0.42	8.65E-04	2.30E-03	Over-expressed
SMDT1	0.42	1.35E-03	3.37E-03	Over-expressed
ARHGAP45	0.42	1.41E-03	3.51E-03	Over-expressed
SMIM29	0.42	1.43E-03	3.54E-03	Over-expressed
RBPMS	0.42	1.44E-03	3.56E-03	Over-expressed
MTMR2	0.42	1.71E-03	4.15E-03	Over-expressed
ASH1L-AS1	0.42	1.90E-03	4.54E-03	Over-expressed
CLBA1	0.42	2.37E-03	5.53E-03	Over-expressed
BTG1	0.42	2.86E-03	6.54E-03	Over-expressed
REEP4	0.42	2.95E-03	6.72E-03	Over-expressed
TXN	0.42	3.42E-03	7.64E-03	Over-expressed
SEC11C	0.42	4.68E-03	0.0101	Over-expressed
MYL5	0.42	7.07E-03	0.0144	Over-expressed
NTHL1	0.42	8.99E-03	0.0178	Over-expressed
L3MBTL1	0.42	9.01E-03	0.0178	Over-expressed
GLIPR2	0.42	9.25E-03	0.0182	Over-expressed
S1PR5	0.42	9.87E-03	0.0193	Over-expressed
PERP	0.42	0.0136	0.0255	Over-expressed
ZNF600	0.42	0.0196	0.0353	Over-expressed
DUSP8	0.42	0.0216	0.0384	Over-expressed
CDC6	0.42	0.0219	0.0389	Over-expressed
MAD2L1	0.42	0.0285	0.0492	Over-expressed
PDCD6	0.41	1.42E-10	3.31E-09	Over-expressed
BCCIP	0.41	2.00E-09	3.07E-08	Over-expressed
C14ORF119	0.41	9.38E-09	1.16E-07	Over-expressed

LOC643387	0.41	4.06E-08	4.14E-07	Over-expressed
SUPV3L1	0.41	7.79E-08	7.19E-07	Over-expressed
EIF5B	0.41	1.72E-07	1.43E-06	Over-expressed
SUB1	0.41	6.85E-07	4.63E-06	Over-expressed
CBY1	0.41	6.89E-07	4.66E-06	Over-expressed
LTA4H	0.41	7.15E-07	4.81E-06	Over-expressed
EIF3I	0.41	7.90E-07	5.22E-06	Over-expressed
PDAP1	0.41	9.43E-07	6.07E-06	Over-expressed
COPS3	0.41	1.09E-06	6.84E-06	Over-expressed
ALKBH4	0.41	1.64E-06	9.75E-06	Over-expressed
PHF23	0.41	2.15E-06	1.23E-05	Over-expressed
RNF216P1	0.41	2.24E-06	1.27E-05	Over-expressed
ZNF408	0.41	3.28E-06	1.77E-05	Over-expressed
CSNK2A2	0.41	3.72E-06	1.98E-05	Over-expressed
GYG1	0.41	4.32E-06	2.26E-05	Over-expressed
FRA10AC1	0.41	4.97E-06	2.55E-05	Over-expressed
CCT2	0.41	6.15E-06	3.07E-05	Over-expressed
PAF1	0.41	1.02E-05	4.79E-05	Over-expressed
NDUFB3	0.41	1.24E-05	5.68E-05	Over-expressed
NDUFC1	0.41	1.38E-05	6.22E-05	Over-expressed
TMED1	0.41	1.43E-05	6.42E-05	Over-expressed
MRPL36	0.41	1.46E-05	6.51E-05	Over-expressed
PPP4C	0.41	2.01E-05	8.63E-05	Over-expressed
TRNAU1AP	0.41	2.06E-05	8.81E-05	Over-expressed
ZFAND1	0.41	3.10E-05	1.26E-04	Over-expressed
BUD23	0.41	3.19E-05	1.29E-04	Over-expressed
SUGT1	0.41	4.32E-05	1.68E-04	Over-expressed
MTA3	0.41	4.80E-05	1.84E-04	Over-expressed
RFC2	0.41	5.06E-05	1.93E-04	Over-expressed
ZNRF1	0.41	5.85E-05	2.19E-04	Over-expressed
MRPL15	0.41	7.44E-05	2.70E-04	Over-expressed
ATP6V0E1	0.41	1.41E-04	4.71E-04	Over-expressed
C1ORF216	0.41	2.20E-04	6.98E-04	Over-expressed
CLPP	0.41	2.39E-04	7.50E-04	Over-expressed
RPUSD1	0.41	2.67E-04	8.24E-04	Over-expressed
NUDT16L1	0.41	3.23E-04	9.75E-04	Over-expressed
SLC7A6	0.41	3.76E-04	1.11E-03	Over-expressed
RHOC	0.41	5.25E-04	1.49E-03	Over-expressed
TMEM120A	0.41	7.52E-04	2.03E-03	Over-expressed
NR2F6	0.41	8.56E-04	2.28E-03	Over-expressed
MIS18A	0.41	8.84E-04	2.34E-03	Over-expressed
GIPC1	0.41	1.28E-03	3.23E-03	Over-expressed
CEP131	0.41	1.35E-03	3.37E-03	Over-expressed
DSE	0.41	1.41E-03	3.51E-03	Over-expressed
ARVCF	0.41	1.43E-03	3.54E-03	Over-expressed
CLSTN1	0.41	3.19E-03	7.19E-03	Over-expressed
RSKR	0.41	3.24E-03	7.28E-03	Over-expressed
PARVG	0.41	8.07E-03	0.0162	Over-expressed
IL27RA	0.41	9.16E-03	0.0181	Over-expressed
KIZ	0.41	0.0157	0.0289	Over-expressed
C5ORF34	0.41	0.017	0.031	Over-expressed
MMD	0.41	0.0173	0.0314	Over-expressed
ZWINT	0.41	0.0185	0.0334	Over-expressed
PRNP	0.41	0.02	0.0358	Over-expressed
PRKAR1B	0.41	0.0224	0.0396	Over-expressed
PARL	0.4	1.56E-10	3.57E-09	Over-expressed
CCDC127	0.4	2.78E-09	4.11E-08	Over-expressed

ZNHIT3	0.4	2.67E-08	2.88E-07	Over-expressed
DDX54	0.4	3.12E-08	3.30E-07	Over-expressed
ANXA11	0.4	1.49E-07	1.27E-06	Over-expressed
UBE2L3	0.4	3.71E-07	2.73E-06	Over-expressed
SCAND2P	0.4	5.72E-07	3.96E-06	Over-expressed
RASSF1	0.4	8.82E-07	5.74E-06	Over-expressed
XRCC6	0.4	1.12E-06	7.04E-06	Over-expressed
THAP4	0.4	1.54E-06	9.19E-06	Over-expressed
PPP1CA	0.4	1.85E-06	1.08E-05	Over-expressed
MRPL45	0.4	3.39E-06	1.82E-05	Over-expressed
UBAC2	0.4	1.21E-05	5.56E-05	Over-expressed
MED11	0.4	6.67E-05	2.45E-04	Over-expressed
GGCT	0.4	6.81E-05	2.50E-04	Over-expressed
SH3GLB2	0.4	7.04E-05	2.57E-04	Over-expressed
FAM86DP	0.4	1.08E-04	3.75E-04	Over-expressed
TRABD	0.4	2.22E-04	7.02E-04	Over-expressed
ZFYVE19	0.4	2.62E-04	8.13E-04	Over-expressed
SS18L2	0.4	3.83E-04	1.13E-03	Over-expressed
PPM1M	0.4	3.88E-04	1.14E-03	Over-expressed
PPT1	0.4	4.25E-04	1.24E-03	Over-expressed
ZC3H3	0.4	4.71E-04	1.35E-03	Over-expressed
STXBP2	0.4	6.09E-04	1.69E-03	Over-expressed
NDOR1	0.4	6.85E-04	1.87E-03	Over-expressed
TMEM161A	0.4	7.01E-04	1.91E-03	Over-expressed
KIFC3	0.4	9.75E-04	2.54E-03	Over-expressed
CASP4	0.4	1.13E-03	2.90E-03	Over-expressed
LINC01011	0.4	1.22E-03	3.10E-03	Over-expressed
TMEM106C	0.4	1.44E-03	3.57E-03	Over-expressed
IFT22	0.4	1.72E-03	4.18E-03	Over-expressed
EEF2KMT	0.4	1.74E-03	4.22E-03	Over-expressed
PM20D2	0.4	9.87E-03	0.0193	Over-expressed
ST20	0.4	0.0128	0.0243	Over-expressed
SELPLG	0.4	0.0171	0.0311	Over-expressed
MOK	0.4	0.0177	0.0321	Over-expressed
SDF2L1	0.4	0.0177	0.0322	Over-expressed
BAIAP2L1	0.4	0.0244	0.0429	Over-expressed
SF3B6	0.39	9.09E-10	1.57E-08	Over-expressed
CPSF3	0.39	2.23E-07	1.77E-06	Over-expressed
DMAC2	0.39	3.28E-07	2.46E-06	Over-expressed
CRNKL1	0.39	3.85E-07	2.81E-06	Over-expressed
ANAPC7	0.39	5.94E-07	4.10E-06	Over-expressed
MMS19	0.39	8.36E-07	5.47E-06	Over-expressed
MAZ	0.39	9.28E-07	5.98E-06	Over-expressed
PSMD9	0.39	9.46E-07	6.08E-06	Over-expressed
ATP5F1C	0.39	2.13E-06	1.22E-05	Over-expressed
MRPL1	0.39	2.58E-06	1.44E-05	Over-expressed
BRK1	0.39	4.14E-06	2.18E-05	Over-expressed
ATG7	0.39	4.98E-06	2.56E-05	Over-expressed
NDUFA9	0.39	8.91E-06	4.25E-05	Over-expressed
ARPC1A	0.39	1.43E-05	6.39E-05	Over-expressed
CFDP1	0.39	1.72E-05	7.51E-05	Over-expressed
WDR83	0.39	1.83E-05	7.93E-05	Over-expressed
VPS51	0.39	2.97E-05	1.21E-04	Over-expressed
DTX2	0.39	3.53E-05	1.41E-04	Over-expressed
FAM104B	0.39	3.86E-05	1.53E-04	Over-expressed
ZBTB11-AS1	0.39	3.90E-05	1.54E-04	Over-expressed
SMIM11A	0.39	5.26E-05	2.00E-04	Over-expressed

HSP90AB1	0.39	9.26E-05	3.28E-04	Over-expressed
ZNF780A	0.39	1.14E-04	3.95E-04	Over-expressed
GMPPA	0.39	1.34E-04	4.53E-04	Over-expressed
TDP2	0.39	1.43E-04	4.78E-04	Over-expressed
MIEN1	0.39	1.63E-04	5.37E-04	Over-expressed
POP4	0.39	2.34E-04	7.37E-04	Over-expressed
ARMC7	0.39	2.61E-04	8.09E-04	Over-expressed
CISD1	0.39	4.46E-04	1.29E-03	Over-expressed
NEU1	0.39	5.20E-04	1.48E-03	Over-expressed
POMP	0.39	6.62E-04	1.82E-03	Over-expressed
PLD2	0.39	6.85E-04	1.87E-03	Over-expressed
PTDSS1	0.39	7.38E-04	2.00E-03	Over-expressed
TVP23C	0.39	8.09E-04	2.17E-03	Over-expressed
ATAD3A	0.39	1.00E-03	2.61E-03	Over-expressed
AGO2	0.39	1.91E-03	4.57E-03	Over-expressed
RRAS	0.39	2.28E-03	5.35E-03	Over-expressed
PRDX1	0.39	2.75E-03	6.31E-03	Over-expressed
SPI1	0.39	7.49E-03	0.0152	Over-expressed
SIL1	0.39	7.61E-03	0.0154	Over-expressed
CEP72	0.39	0.0106	0.0206	Over-expressed
CXCL16	0.39	0.013	0.0245	Over-expressed
SAT2	0.39	0.0144	0.0269	Over-expressed
SCN1B	0.39	0.0146	0.0272	Over-expressed
GSN	0.39	0.0212	0.0377	Over-expressed
SRRM2-AS1	0.39	0.0251	0.0438	Over-expressed
PARPBP	0.39	0.0267	0.0463	Over-expressed
NRGN	0.39	0.0278	0.0481	Over-expressed
KHDRBS1	0.38	1.16E-10	2.77E-09	Over-expressed
SRSF3	0.38	8.80E-10	1.54E-08	Over-expressed
LEMD2	0.38	2.73E-08	2.94E-07	Over-expressed
AUP1	0.38	7.14E-08	6.68E-07	Over-expressed
MRPS9	0.38	2.26E-07	1.79E-06	Over-expressed
BCS1L	0.38	4.12E-07	2.99E-06	Over-expressed
SDF2	0.38	7.24E-07	4.85E-06	Over-expressed
PTMA	0.38	1.05E-06	6.69E-06	Over-expressed
SUPT5H	0.38	3.08E-06	1.68E-05	Over-expressed
FKBP3	0.38	4.74E-06	2.45E-05	Over-expressed
FAM89B	0.38	5.68E-06	2.86E-05	Over-expressed
WDR77	0.38	9.90E-06	4.65E-05	Over-expressed
MRPS7	0.38	1.32E-05	5.97E-05	Over-expressed
UBL7	0.38	2.23E-05	9.43E-05	Over-expressed
INPP5K	0.38	3.02E-05	1.23E-04	Over-expressed
COPS5	0.38	3.09E-05	1.26E-04	Over-expressed
PRPSAP2	0.38	3.51E-05	1.41E-04	Over-expressed
SLC35B2	0.38	3.90E-05	1.54E-04	Over-expressed
ERI3	0.38	4.50E-05	1.74E-04	Over-expressed
FRG1	0.38	6.18E-05	2.29E-04	Over-expressed
SIPA1	0.38	7.22E-05	2.63E-04	Over-expressed
WDR73	0.38	1.03E-04	3.60E-04	Over-expressed
MAGEF1	0.38	1.17E-04	4.03E-04	Over-expressed
THYN1	0.38	1.34E-04	4.55E-04	Over-expressed
CREB3	0.38	1.49E-04	4.95E-04	Over-expressed
GIT1	0.38	1.49E-04	4.96E-04	Over-expressed
SEC61B	0.38	1.63E-04	5.38E-04	Over-expressed
MMP19	0.38	1.91E-04	6.21E-04	Over-expressed
GNPDA1	0.38	2.04E-04	6.55E-04	Over-expressed
ATP5PD	0.38	2.55E-04	7.92E-04	Over-expressed

NDUFV3	0.38	2.65E-04	8.20E-04	Over-expressed
CDK10	0.38	2.99E-04	9.11E-04	Over-expressed
RNF126	0.38	2.99E-04	9.12E-04	Over-expressed
DUS2	0.38	3.16E-04	9.58E-04	Over-expressed
COX8A	0.38	5.02E-04	1.43E-03	Over-expressed
MRPL40	0.38	5.05E-04	1.44E-03	Over-expressed
PDLIM1	0.38	5.33E-04	1.51E-03	Over-expressed
SLC12A9	0.38	5.40E-04	1.52E-03	Over-expressed
THAP7	0.38	5.50E-04	1.55E-03	Over-expressed
ATRIP	0.38	6.17E-04	1.71E-03	Over-expressed
ITGB3BP	0.38	8.84E-04	2.34E-03	Over-expressed
TMEM186	0.38	9.52E-04	2.50E-03	Over-expressed
ZNF784	0.38	1.73E-03	4.20E-03	Over-expressed
TMEM208	0.38	1.95E-03	4.66E-03	Over-expressed
KIF22	0.38	2.03E-03	4.82E-03	Over-expressed
NDUFAF6	0.38	2.21E-03	5.22E-03	Over-expressed
CCT6P1	0.38	2.85E-03	6.51E-03	Over-expressed
PIK3R2	0.38	2.91E-03	6.64E-03	Over-expressed
MAD1L1	0.38	2.96E-03	6.73E-03	Over-expressed
NDUFA1	0.38	3.14E-03	7.10E-03	Over-expressed
PDCD4-AS1	0.38	3.37E-03	7.55E-03	Over-expressed
RTKN	0.38	3.55E-03	7.87E-03	Over-expressed
RBM38	0.38	3.67E-03	8.09E-03	Over-expressed
SLC25A29	0.38	4.49E-03	9.68E-03	Over-expressed
LOC100128288	0.38	6.79E-03	0.0139	Over-expressed
EZH2	0.38	9.17E-03	0.0181	Over-expressed
FLJ44635	0.38	0.0104	0.0202	Over-expressed
AAMDC	0.38	0.0123	0.0235	Over-expressed
GUSBP11	0.38	0.0163	0.03	Over-expressed
TFEB	0.38	0.0177	0.0321	Over-expressed
GRASP	0.38	0.0228	0.0402	Over-expressed
RAB37	0.38	0.0236	0.0416	Over-expressed
UBE2D2	0.37	4.46E-10	8.76E-09	Over-expressed
HDAC3	0.37	6.78E-08	6.38E-07	Over-expressed
CDK5RAP1	0.37	2.64E-07	2.04E-06	Over-expressed
PYM1	0.37	2.96E-07	2.25E-06	Over-expressed
THOC7	0.37	1.11E-06	6.98E-06	Over-expressed
GID8	0.37	1.72E-06	1.01E-05	Over-expressed
B4GALT3	0.37	3.44E-06	1.85E-05	Over-expressed
TMEM120B	0.37	4.06E-06	2.15E-05	Over-expressed
COPS7B	0.37	4.30E-06	2.26E-05	Over-expressed
UBE2J2	0.37	8.00E-06	3.86E-05	Over-expressed
ASXL1	0.37	1.41E-05	6.32E-05	Over-expressed
WDR6	0.37	1.42E-05	6.39E-05	Over-expressed
ILF2	0.37	1.44E-05	6.44E-05	Over-expressed
ACBD6	0.37	2.06E-05	8.79E-05	Over-expressed
VPS25	0.37	2.08E-05	8.89E-05	Over-expressed
DAD1	0.37	2.21E-05	9.38E-05	Over-expressed
CACYBP	0.37	2.70E-05	1.12E-04	Over-expressed
TMEM42	0.37	2.84E-05	1.17E-04	Over-expressed
HDAC1	0.37	3.69E-05	1.47E-04	Over-expressed
DBNL	0.37	3.96E-05	1.56E-04	Over-expressed
ERP29	0.37	4.61E-05	1.78E-04	Over-expressed
SLC25A39	0.37	4.95E-05	1.89E-04	Over-expressed
EXOSC2	0.37	5.02E-05	1.92E-04	Over-expressed
ALG3	0.37	5.49E-05	2.07E-04	Over-expressed
POLRMT	0.37	1.15E-04	3.96E-04	Over-expressed

CTNNBL1	0.37	1.23E-04	4.21E-04	Over-expressed
BORCS8	0.37	1.37E-04	4.62E-04	Over-expressed
DXO	0.37	1.43E-04	4.79E-04	Over-expressed
RIOK1	0.37	1.62E-04	5.34E-04	Over-expressed
RPIA	0.37	1.68E-04	5.53E-04	Over-expressed
CARS2	0.37	1.70E-04	5.57E-04	Over-expressed
PLSCR3	0.37	1.77E-04	5.78E-04	Over-expressed
SMG5	0.37	1.81E-04	5.89E-04	Over-expressed
DNAL4	0.37	1.94E-04	6.27E-04	Over-expressed
PDXK	0.37	2.15E-04	6.84E-04	Over-expressed
SAYSD1	0.37	2.26E-04	7.14E-04	Over-expressed
SIX5	0.37	2.28E-04	7.18E-04	Over-expressed
SIRT7	0.37	2.61E-04	8.10E-04	Over-expressed
CCDC77	0.37	2.78E-04	8.55E-04	Over-expressed
NDUFA8	0.37	2.83E-04	8.67E-04	Over-expressed
TMEM177	0.37	2.97E-04	9.07E-04	Over-expressed
GNL3	0.37	3.51E-04	1.05E-03	Over-expressed
SLC66A1	0.37	3.52E-04	1.05E-03	Over-expressed
MUTYH	0.37	3.52E-04	1.05E-03	Over-expressed
CCDC86	0.37	3.85E-04	1.14E-03	Over-expressed
GYS1	0.37	4.77E-04	1.37E-03	Over-expressed
COX17	0.37	4.79E-04	1.37E-03	Over-expressed
RNASEH2B	0.37	4.82E-04	1.38E-03	Over-expressed
ABRAXAS1	0.37	5.10E-04	1.45E-03	Over-expressed
ATP6V1C1	0.37	5.17E-04	1.47E-03	Over-expressed
THAP8	0.37	5.47E-04	1.54E-03	Over-expressed
CCNQ	0.37	8.61E-04	2.29E-03	Over-expressed
MPZL1	0.37	1.17E-03	2.97E-03	Over-expressed
NSMAF	0.37	1.44E-03	3.58E-03	Over-expressed
TSPAN3	0.37	1.64E-03	3.99E-03	Over-expressed
BRI3BP	0.37	2.26E-03	5.32E-03	Over-expressed
NDUFAB1	0.37	2.41E-03	5.62E-03	Over-expressed
LOC93622	0.37	4.07E-03	8.87E-03	Over-expressed
RPP25L	0.37	4.99E-03	0.0106	Over-expressed
IFT52	0.37	5.56E-03	0.0116	Over-expressed
FAM86HP	0.37	6.17E-03	0.0128	Over-expressed
FLT3LG	0.37	6.62E-03	0.0136	Over-expressed
ILVBL	0.37	8.28E-03	0.0166	Over-expressed
CISD3	0.37	0.0106	0.0206	Over-expressed
EFHC1	0.37	0.0113	0.0216	Over-expressed
CHD7	0.37	0.0117	0.0224	Over-expressed
CHTF18	0.37	0.0144	0.0268	Over-expressed
PPP1R15A	0.37	0.0153	0.0283	Over-expressed
LAMB2P1	0.37	0.0165	0.0302	Over-expressed
ETV5	0.37	0.0209	0.0373	Over-expressed
HLA-A	0.37	0.0242	0.0425	Over-expressed
RNF7	0.36	3.14E-09	4.56E-08	Over-expressed
PPM1G	0.36	2.87E-07	2.19E-06	Over-expressed
TTC27	0.36	3.21E-07	2.42E-06	Over-expressed
PPP1CC	0.36	3.53E-07	2.62E-06	Over-expressed
TMEM18	0.36	1.01E-06	6.44E-06	Over-expressed
PSMB5	0.36	1.48E-06	8.92E-06	Over-expressed
SPPL3	0.36	1.82E-06	1.07E-05	Over-expressed
ATRAID	0.36	1.89E-06	1.10E-05	Over-expressed
SART1	0.36	3.06E-06	1.67E-05	Over-expressed
ZNF628	0.36	7.52E-06	3.66E-05	Over-expressed
MED19	0.36	1.11E-05	5.16E-05	Over-expressed

SSB	0.36	1.25E-05	5.68E-05	Over-expressed
C2ORF68	0.36	1.85E-05	8.00E-05	Over-expressed
POLR2D	0.36	3.30E-05	1.33E-04	Over-expressed
DALRD3	0.36	4.24E-05	1.66E-04	Over-expressed
TOMM20	0.36	4.29E-05	1.67E-04	Over-expressed
SELENOS	0.36	4.54E-05	1.75E-04	Over-expressed
DLGAP4	0.36	4.82E-05	1.85E-04	Over-expressed
METTL23	0.36	5.05E-05	1.92E-04	Over-expressed
E2F6	0.36	5.39E-05	2.04E-04	Over-expressed
STIP1	0.36	1.02E-04	3.58E-04	Over-expressed
ZNF605	0.36	1.30E-04	4.42E-04	Over-expressed
PMM2	0.36	1.33E-04	4.52E-04	Over-expressed
ZNF544	0.36	2.27E-04	7.16E-04	Over-expressed
FAM189B	0.36	3.67E-04	1.09E-03	Over-expressed
NOP14-AS1	0.36	4.03E-04	1.18E-03	Over-expressed
OGFR	0.36	4.14E-04	1.21E-03	Over-expressed
PDCL3P4	0.36	4.26E-04	1.24E-03	Over-expressed
SCAMP3	0.36	5.09E-04	1.45E-03	Over-expressed
HEXD	0.36	5.62E-04	1.58E-03	Over-expressed
NOC2L	0.36	5.90E-04	1.64E-03	Over-expressed
NFKB2	0.36	6.16E-04	1.70E-03	Over-expressed
CS	0.36	7.29E-04	1.98E-03	Over-expressed
ZNF586	0.36	7.40E-04	2.01E-03	Over-expressed
SRPRB	0.36	8.02E-04	2.15E-03	Over-expressed
RUVBL1	0.36	8.84E-04	2.34E-03	Over-expressed
TRMT12	0.36	9.48E-04	2.49E-03	Over-expressed
TMEM201	0.36	1.00E-03	2.61E-03	Over-expressed
GTPBP6	0.36	1.06E-03	2.73E-03	Over-expressed
IQCC	0.36	1.35E-03	3.37E-03	Over-expressed
ADPRM	0.36	2.24E-03	5.27E-03	Over-expressed
SGSM2	0.36	2.33E-03	5.45E-03	Over-expressed
ECE2	0.36	2.57E-03	5.95E-03	Over-expressed
NECTIN2	0.36	4.78E-03	0.0102	Over-expressed
ZNF419	0.36	8.27E-03	0.0166	Over-expressed
HEXIM2	0.36	8.80E-03	0.0175	Over-expressed
KPNA2	0.36	9.71E-03	0.019	Over-expressed
CXCR4	0.36	0.0152	0.0282	Over-expressed
LYL1	0.36	0.0155	0.0286	Over-expressed
LIMK1	0.36	0.0217	0.0386	Over-expressed
CACNB1	0.36	0.0268	0.0465	Over-expressed
BMS1	0.35	6.20E-10	1.14E-08	Over-expressed
SMARCAL1	0.35	5.94E-06	2.98E-05	Over-expressed
CCT6A	0.35	6.88E-06	3.39E-05	Over-expressed
FAM219B	0.35	1.22E-05	5.58E-05	Over-expressed
NAGK	0.35	1.32E-05	5.99E-05	Over-expressed
UBE2F	0.35	1.53E-05	6.77E-05	Over-expressed
RNF25	0.35	2.75E-05	1.14E-04	Over-expressed
OSTC	0.35	2.97E-05	1.21E-04	Over-expressed
ENSA	0.35	6.33E-05	2.34E-04	Over-expressed
ZSCAN2	0.35	6.87E-05	2.52E-04	Over-expressed
BABAM1	0.35	8.30E-05	2.98E-04	Over-expressed
TRMT2A	0.35	9.23E-05	3.27E-04	Over-expressed
ZRSR2	0.35	1.17E-04	4.02E-04	Over-expressed
ADRM1	0.35	1.22E-04	4.18E-04	Over-expressed
NCLN	0.35	1.32E-04	4.46E-04	Over-expressed
TADA3	0.35	1.35E-04	4.57E-04	Over-expressed
ELOF1	0.35	1.70E-04	5.59E-04	Over-expressed

UFD1	0.35	1.86E-04	6.05E-04	Over-expressed
PLEKHA8P1	0.35	2.23E-04	7.05E-04	Over-expressed
TUBD1	0.35	2.63E-04	8.14E-04	Over-expressed
HMGN2	0.35	3.74E-04	1.11E-03	Over-expressed
TRMT6	0.35	3.88E-04	1.15E-03	Over-expressed
PUM3	0.35	4.36E-04	1.27E-03	Over-expressed
SAE1	0.35	4.42E-04	1.28E-03	Over-expressed
CORO7	0.35	4.45E-04	1.29E-03	Over-expressed
UBQLN4	0.35	4.89E-04	1.40E-03	Over-expressed
PARK7	0.35	7.23E-04	1.96E-03	Over-expressed
LMAN2	0.35	7.75E-04	2.09E-03	Over-expressed
GTF2H5	0.35	8.15E-04	2.18E-03	Over-expressed
TBC1D13	0.35	9.96E-04	2.59E-03	Over-expressed
HMGN3	0.35	1.16E-03	2.95E-03	Over-expressed
UBE2V2	0.35	1.23E-03	3.12E-03	Over-expressed
NDUFA6	0.35	1.24E-03	3.12E-03	Over-expressed
TRPT1	0.35	1.33E-03	3.33E-03	Over-expressed
HDAC2	0.35	1.35E-03	3.38E-03	Over-expressed
P3H1	0.35	1.44E-03	3.57E-03	Over-expressed
PTPN2	0.35	1.61E-03	3.94E-03	Over-expressed
JADE2	0.35	2.22E-03	5.23E-03	Over-expressed
CFAP36	0.35	2.26E-03	5.31E-03	Over-expressed
ZDHHC24	0.35	3.27E-03	7.34E-03	Over-expressed
GUSBP4	0.35	3.37E-03	7.53E-03	Over-expressed
PLD6	0.35	3.46E-03	7.70E-03	Over-expressed
DLEU1	0.35	3.75E-03	8.24E-03	Over-expressed
HNRNPA1L2	0.35	5.43E-03	0.0114	Over-expressed
RGS14	0.35	8.71E-03	0.0173	Over-expressed
SERTAD1	0.35	0.0104	0.0202	Over-expressed
TXNRD2	0.35	0.0138	0.0259	Over-expressed
UBE2Q2	0.35	0.0193	0.0348	Over-expressed
CENPX	0.35	0.0275	0.0476	Over-expressed
PWP1	0.34	1.02E-08	1.25E-07	Over-expressed
RBM10	0.34	2.05E-08	2.28E-07	Over-expressed
GRPEL2	0.34	1.14E-06	7.13E-06	Over-expressed
SRRT	0.34	1.80E-06	1.06E-05	Over-expressed
DPY30	0.34	2.28E-06	1.29E-05	Over-expressed
PANK2	0.34	4.56E-06	2.37E-05	Over-expressed
RALA	0.34	5.09E-06	2.61E-05	Over-expressed
STK25	0.34	5.99E-06	3.00E-05	Over-expressed
FIZ1	0.34	6.00E-06	3.01E-05	Over-expressed
PHB2	0.34	7.63E-06	3.71E-05	Over-expressed
PYGO2	0.34	9.23E-06	4.38E-05	Over-expressed
VPS16	0.34	1.26E-05	5.75E-05	Over-expressed
COX7A2L	0.34	1.72E-05	7.51E-05	Over-expressed
PFDN1	0.34	2.31E-05	9.71E-05	Over-expressed
C11ORF68	0.34	2.52E-05	1.05E-04	Over-expressed
DNAJC5	0.34	3.83E-05	1.51E-04	Over-expressed
GGA1	0.34	7.41E-05	2.69E-04	Over-expressed
ACTB	0.34	1.04E-04	3.62E-04	Over-expressed
PPP1R7	0.34	1.25E-04	4.27E-04	Over-expressed
COA1	0.34	1.28E-04	4.38E-04	Over-expressed
LAMTOR1	0.34	1.37E-04	4.62E-04	Over-expressed
FUS	0.34	1.40E-04	4.69E-04	Over-expressed
TMEM126A	0.34	1.42E-04	4.76E-04	Over-expressed
MCTS1	0.34	1.94E-04	6.29E-04	Over-expressed
XAB2	0.34	1.98E-04	6.38E-04	Over-expressed

CYREN	0.34	2.04E-04	6.54E-04	Over-expressed
OGFOD2	0.34	2.04E-04	6.55E-04	Over-expressed
HSPD1	0.34	2.08E-04	6.64E-04	Over-expressed
YKT6	0.34	2.43E-04	7.60E-04	Over-expressed
RPUSD3	0.34	4.43E-04	1.28E-03	Over-expressed
MAN1B1	0.34	4.75E-04	1.36E-03	Over-expressed
ZNF707	0.34	6.03E-04	1.67E-03	Over-expressed
EFHD2	0.34	6.19E-04	1.71E-03	Over-expressed
SLC25A26	0.34	6.27E-04	1.73E-03	Over-expressed
MRPS33	0.34	8.34E-04	2.23E-03	Over-expressed
ZNF576	0.34	9.12E-04	2.40E-03	Over-expressed
STARD3NL	0.34	1.15E-03	2.93E-03	Over-expressed
CAMK1	0.34	1.43E-03	3.55E-03	Over-expressed
TMEM150A	0.34	1.54E-03	3.78E-03	Over-expressed
UROS	0.34	1.60E-03	3.92E-03	Over-expressed
NAPB	0.34	2.93E-03	6.67E-03	Over-expressed
MDH2	0.34	3.82E-03	8.39E-03	Over-expressed
NUP93	0.34	4.00E-03	8.74E-03	Over-expressed
MROH1	0.34	5.73E-03	0.012	Over-expressed
PTRH1	0.34	9.82E-03	0.0192	Over-expressed
FLJ42627	0.34	0.0137	0.0257	Over-expressed
TNFSF12	0.34	0.0147	0.0273	Over-expressed
XRCC4	0.34	0.021	0.0374	Over-expressed
CCDC149	0.34	0.0223	0.0395	Over-expressed
KSR1	0.34	0.0227	0.04	Over-expressed
PILRA	0.34	0.0251	0.0438	Over-expressed
DENR	0.33	2.42E-07	1.89E-06	Over-expressed
DUSP28	0.33	6.19E-07	4.24E-06	Over-expressed
LLPH	0.33	1.30E-06	7.95E-06	Over-expressed
NGRN	0.33	2.51E-06	1.40E-05	Over-expressed
RBMX	0.33	3.39E-06	1.82E-05	Over-expressed
KCTD2	0.33	4.65E-06	2.41E-05	Over-expressed
SYF2	0.33	5.26E-06	2.68E-05	Over-expressed
GPN2	0.33	6.64E-06	3.28E-05	Over-expressed
POLR3F	0.33	7.35E-06	3.59E-05	Over-expressed
EFTUD2	0.33	7.35E-06	3.59E-05	Over-expressed
NRF1	0.33	8.05E-06	3.88E-05	Over-expressed
TMEM183A	0.33	1.26E-05	5.75E-05	Over-expressed
TIMM29	0.33	1.54E-05	6.83E-05	Over-expressed
NVL	0.33	2.99E-05	1.22E-04	Over-expressed
CWC27	0.33	3.39E-05	1.36E-04	Over-expressed
NEURL4	0.33	3.88E-05	1.53E-04	Over-expressed
LMBR1L	0.33	4.02E-05	1.58E-04	Over-expressed
CENPB	0.33	5.16E-05	1.96E-04	Over-expressed
GNG5	0.33	6.47E-05	2.39E-04	Over-expressed
STN1	0.33	9.51E-05	3.35E-04	Over-expressed
MOV10	0.33	1.55E-04	5.14E-04	Over-expressed
HIF1AN	0.33	1.56E-04	5.16E-04	Over-expressed
NOA1	0.33	1.81E-04	5.90E-04	Over-expressed
TMEM203	0.33	2.69E-04	8.30E-04	Over-expressed
LOC90784	0.33	4.38E-04	1.27E-03	Over-expressed
ZNF34	0.33	4.63E-04	1.34E-03	Over-expressed
RNF5	0.33	4.93E-04	1.41E-03	Over-expressed
TTC1	0.33	4.99E-04	1.42E-03	Over-expressed
SLC39A7	0.33	5.12E-04	1.46E-03	Over-expressed
EPN2	0.33	6.71E-04	1.84E-03	Over-expressed
MYCBP	0.33	6.77E-04	1.86E-03	Over-expressed

PSMA5	0.33	7.42E-04	2.01E-03	Over-expressed
CIAPIN1	0.33	7.86E-04	2.12E-03	Over-expressed
MRPS11	0.33	8.12E-04	2.17E-03	Over-expressed
SPIDR	0.33	1.16E-03	2.95E-03	Over-expressed
CALM3	0.33	1.25E-03	3.16E-03	Over-expressed
PTK2	0.33	1.34E-03	3.35E-03	Over-expressed
CETN2	0.33	1.37E-03	3.41E-03	Over-expressed
LIG1	0.33	1.78E-03	4.30E-03	Over-expressed
ZNF517	0.33	1.81E-03	4.36E-03	Over-expressed
C19ORF44	0.33	2.25E-03	5.30E-03	Over-expressed
SLC25A11	0.33	2.68E-03	6.18E-03	Over-expressed
YIF1A	0.33	3.48E-03	7.75E-03	Over-expressed
MRPL4	0.33	4.22E-03	9.17E-03	Over-expressed
RABEP2	0.33	4.56E-03	9.82E-03	Over-expressed
BAG1	0.33	5.72E-03	0.012	Over-expressed
UQCRFS1	0.33	5.90E-03	0.0123	Over-expressed
FEN1	0.33	6.08E-03	0.0126	Over-expressed
CD99	0.33	6.38E-03	0.0132	Over-expressed
MRPS28	0.33	6.52E-03	0.0134	Over-expressed
PRDX4	0.33	8.50E-03	0.017	Over-expressed
PAK4	0.33	0.0102	0.0199	Over-expressed
POMZP3	0.33	0.0105	0.0204	Over-expressed
IRF2BP1	0.33	0.0106	0.0205	Over-expressed
NT5DC3	0.33	0.0111	0.0214	Over-expressed
RPA3	0.33	0.0111	0.0215	Over-expressed
KIAA0753	0.33	0.021	0.0375	Over-expressed
GMIP	0.33	0.0213	0.0379	Over-expressed
FBXO24	0.33	0.0259	0.0451	Over-expressed
TALDO1	0.33	0.0273	0.0473	Over-expressed
AAAS	0.32	1.95E-06	1.13E-05	Over-expressed
SUGP1	0.32	2.52E-06	1.41E-05	Over-expressed
GS1-124K5.11	0.32	6.73E-06	3.32E-05	Over-expressed
GLTP	0.32	8.01E-06	3.86E-05	Over-expressed
RNF114	0.32	9.12E-06	4.34E-05	Over-expressed
UBE2Z	0.32	1.09E-05	5.06E-05	Over-expressed
RPN2	0.32	1.67E-05	7.35E-05	Over-expressed
RNPS1	0.32	2.99E-05	1.22E-04	Over-expressed
DPH5	0.32	3.97E-05	1.56E-04	Over-expressed
FARSA	0.32	5.87E-05	2.20E-04	Over-expressed
SKIV2L	0.32	7.63E-05	2.76E-04	Over-expressed
NDUFA12	0.32	8.08E-05	2.91E-04	Over-expressed
XPA	0.32	8.49E-05	3.04E-04	Over-expressed
INTS11	0.32	8.89E-05	3.16E-04	Over-expressed
SUMO1P3	0.32	1.17E-04	4.03E-04	Over-expressed
DDA1	0.32	1.35E-04	4.57E-04	Over-expressed
CXXC1	0.32	2.03E-04	6.51E-04	Over-expressed
NELFB	0.32	2.38E-04	7.47E-04	Over-expressed
ITPKC	0.32	2.41E-04	7.54E-04	Over-expressed
CHMP1A	0.32	4.14E-04	1.21E-03	Over-expressed
PSMC4	0.32	4.51E-04	1.31E-03	Over-expressed
MRPS18B	0.32	4.75E-04	1.36E-03	Over-expressed
TOMM34	0.32	5.80E-04	1.62E-03	Over-expressed
YIPF2	0.32	7.09E-04	1.93E-03	Over-expressed
MED31	0.32	7.45E-04	2.02E-03	Over-expressed
PIN1	0.32	8.02E-04	2.15E-03	Over-expressed
JPT2	0.32	8.07E-04	2.17E-03	Over-expressed
MYL12B	0.32	9.07E-04	2.39E-03	Over-expressed

ATP5MC3	0.32	9.39E-04	2.47E-03	Over-expressed
MED16	0.32	1.14E-03	2.92E-03	Over-expressed
AP5Z1	0.32	1.41E-03	3.51E-03	Over-expressed
FUCA2	0.32	1.45E-03	3.58E-03	Over-expressed
RAB13	0.32	1.80E-03	4.33E-03	Over-expressed
PBX2	0.32	1.93E-03	4.62E-03	Over-expressed
NAGPA	0.32	2.04E-03	4.86E-03	Over-expressed
TRAP1	0.32	2.16E-03	5.11E-03	Over-expressed
ZDHHC12	0.32	2.48E-03	5.76E-03	Over-expressed
ARRDC1-AS1	0.32	2.51E-03	5.83E-03	Over-expressed
MRM1	0.32	3.02E-03	6.85E-03	Over-expressed
C11ORF74	0.32	3.08E-03	6.96E-03	Over-expressed
DKC1	0.32	3.64E-03	8.04E-03	Over-expressed
COQ4	0.32	4.90E-03	0.0105	Over-expressed
CINP	0.32	5.04E-03	0.0107	Over-expressed
FBXL18	0.32	5.50E-03	0.0115	Over-expressed
GLMP	0.32	6.45E-03	0.0133	Over-expressed
PLEKHB2	0.32	7.34E-03	0.0149	Over-expressed
LINC01089	0.32	7.84E-03	0.0158	Over-expressed
NIPSNAP2	0.32	7.88E-03	0.0159	Over-expressed
IFT43	0.32	9.87E-03	0.0193	Over-expressed
PLXNB1	0.32	0.0101	0.0196	Over-expressed
MAD2L2	0.32	0.0116	0.0222	Over-expressed
YIF1B	0.32	0.012	0.023	Over-expressed
ATG4D	0.32	0.0123	0.0233	Over-expressed
NECAB3	0.32	0.0137	0.0257	Over-expressed
PGPEP1	0.32	0.0139	0.026	Over-expressed
MCM3	0.32	0.0141	0.0263	Over-expressed
EZR	0.32	0.0156	0.0287	Over-expressed
ZSCAN5A	0.32	0.0163	0.03	Over-expressed
POLR3D	0.32	0.0184	0.0333	Over-expressed
RNF145	0.32	0.0196	0.0352	Over-expressed
GCNA	0.32	0.0205	0.0367	Over-expressed
IFFO2	0.32	0.0217	0.0386	Over-expressed
ANXA5	0.32	0.0243	0.0427	Over-expressed
RBMS1	0.32	0.0281	0.0485	Over-expressed
NAP1L4	0.31	1.52E-06	9.09E-06	Over-expressed
ABCF1	0.31	6.28E-06	3.13E-05	Over-expressed
CWC15	0.31	9.33E-06	4.42E-05	Over-expressed
PRCC	0.31	2.30E-05	9.70E-05	Over-expressed
PCGF1	0.31	4.11E-05	1.61E-04	Over-expressed
CARM1	0.31	6.21E-05	2.31E-04	Over-expressed
NUP85	0.31	6.55E-05	2.41E-04	Over-expressed
ILK	0.31	7.13E-05	2.60E-04	Over-expressed
RNPEPL1	0.31	7.44E-05	2.70E-04	Over-expressed
ARFIP2	0.31	8.21E-05	2.95E-04	Over-expressed
NSFL1C	0.31	1.14E-04	3.94E-04	Over-expressed
PRKD2	0.31	1.16E-04	4.00E-04	Over-expressed
CNOT10	0.31	1.27E-04	4.34E-04	Over-expressed
PSMD8	0.31	2.56E-04	7.96E-04	Over-expressed
USF2	0.31	3.70E-04	1.10E-03	Over-expressed
THOC3	0.31	3.71E-04	1.10E-03	Over-expressed
INO80E	0.31	4.39E-04	1.27E-03	Over-expressed
CCDC9	0.31	4.58E-04	1.32E-03	Over-expressed
PA2G4P4	0.31	5.04E-04	1.44E-03	Over-expressed
HSPA4	0.31	5.39E-04	1.52E-03	Over-expressed
TCP1	0.31	7.59E-04	2.05E-03	Over-expressed

PTPN6	0.31	9.94E-04	2.59E-03	Over-expressed
USP22	0.31	1.11E-03	2.85E-03	Over-expressed
FKBP8	0.31	1.42E-03	3.53E-03	Over-expressed
RGL2	0.31	2.11E-03	5.01E-03	Over-expressed
SLC3A2	0.31	2.48E-03	5.76E-03	Over-expressed
TMUB1	0.31	2.64E-03	6.10E-03	Over-expressed
LRRC27	0.31	3.41E-03	7.62E-03	Over-expressed
CDK2AP2	0.31	3.45E-03	7.69E-03	Over-expressed
UNC93B1	0.31	4.62E-03	9.94E-03	Over-expressed
TIPIN	0.31	4.75E-03	0.0102	Over-expressed
SNAPC4	0.31	4.85E-03	0.0104	Over-expressed
RBFA	0.31	4.97E-03	0.0106	Over-expressed
IDH3G	0.31	5.09E-03	0.0108	Over-expressed
EMC10	0.31	5.18E-03	0.011	Over-expressed
CNOT6	0.31	6.06E-03	0.0126	Over-expressed
NTPCR	0.31	6.26E-03	0.0129	Over-expressed
MPG	0.31	6.94E-03	0.0142	Over-expressed
MZT1	0.31	7.08E-03	0.0144	Over-expressed
TLE5	0.31	7.48E-03	0.0151	Over-expressed
TRADD	0.31	7.94E-03	0.016	Over-expressed
CDKN2D	0.31	8.08E-03	0.0162	Over-expressed
RHNO1	0.31	8.15E-03	0.0163	Over-expressed
COMMD1	0.31	9.11E-03	0.018	Over-expressed
SMARCA4	0.31	9.12E-03	0.018	Over-expressed
ABHD17A	0.31	9.36E-03	0.0184	Over-expressed
BORCS5	0.31	0.01	0.0196	Over-expressed
FBXL19	0.31	0.0107	0.0207	Over-expressed
ZNF226	0.31	0.0128	0.0242	Over-expressed
YPEL3	0.31	0.0129	0.0243	Over-expressed
AMDHD2	0.31	0.0136	0.0256	Over-expressed
FAM86JP	0.31	0.0188	0.0339	Over-expressed
MCMDC2	0.31	0.0205	0.0367	Over-expressed
SDC3	0.31	0.0215	0.0381	Over-expressed
TUFT1	0.31	0.0241	0.0423	Over-expressed
ZNF771	0.31	0.025	0.0436	Over-expressed
PMVK	0.31	0.0288	0.0496	Over-expressed
EIF1AD	0.3	9.43E-07	6.07E-06	Over-expressed
HDGF	0.3	5.35E-06	2.72E-05	Over-expressed
PTPMT1	0.3	7.55E-06	3.68E-05	Over-expressed
RBM17	0.3	1.96E-05	8.42E-05	Over-expressed
EXOSC9	0.3	2.63E-05	1.09E-04	Over-expressed
TUBGCP2	0.3	3.07E-05	1.25E-04	Over-expressed
UTP14A	0.3	3.74E-05	1.48E-04	Over-expressed
ERAL1	0.3	7.64E-05	2.77E-04	Over-expressed
DDX41	0.3	8.00E-05	2.89E-04	Over-expressed
SF3A3	0.3	8.69E-05	3.10E-04	Over-expressed
YARS2	0.3	8.94E-05	3.18E-04	Over-expressed
WDR75	0.3	1.06E-04	3.68E-04	Over-expressed
ZNF346	0.3	1.37E-04	4.61E-04	Over-expressed
PHF1	0.3	2.01E-04	6.48E-04	Over-expressed
ELAC2	0.3	2.04E-04	6.54E-04	Over-expressed
COASY	0.3	2.20E-04	6.97E-04	Over-expressed
SUMO3	0.3	2.68E-04	8.27E-04	Over-expressed
ANKLE2	0.3	3.38E-04	1.02E-03	Over-expressed
FAM193B	0.3	3.54E-04	1.06E-03	Over-expressed
TAF7	0.3	8.19E-04	2.19E-03	Over-expressed
NCOA5	0.3	1.08E-03	2.79E-03	Over-expressed

PRMT7	0.3	1.17E-03	2.98E-03	Over-expressed
ANKRD54	0.3	1.21E-03	3.06E-03	Over-expressed
NHEJ1	0.3	1.30E-03	3.27E-03	Over-expressed
POLR2J4	0.3	1.38E-03	3.44E-03	Over-expressed
HNRNPA0	0.3	1.52E-03	3.75E-03	Over-expressed
TSEN2	0.3	1.63E-03	3.97E-03	Over-expressed
AVEN	0.3	2.43E-03	5.66E-03	Over-expressed
FOXP1	0.3	2.75E-03	6.31E-03	Over-expressed
LGALSL	0.3	3.95E-03	8.65E-03	Over-expressed
FANCL	0.3	4.15E-03	9.03E-03	Over-expressed
MTIF3	0.3	4.25E-03	9.22E-03	Over-expressed
SNX8	0.3	4.36E-03	9.43E-03	Over-expressed
DUS3L	0.3	4.38E-03	9.48E-03	Over-expressed
IPO5	0.3	4.69E-03	0.0101	Over-expressed
ZNF629	0.3	4.94E-03	0.0105	Over-expressed
PIDD1	0.3	5.03E-03	0.0107	Over-expressed
PIN4P1	0.3	5.06E-03	0.0107	Over-expressed
AGBL5	0.3	6.52E-03	0.0134	Over-expressed
SMARCB1	0.3	7.24E-03	0.0147	Over-expressed
ORAI1	0.3	8.52E-03	0.017	Over-expressed
BID	0.3	8.53E-03	0.017	Over-expressed
SPATS2	0.3	0.0106	0.0206	Over-expressed
DMWD	0.3	0.0109	0.021	Over-expressed
ALDH16A1	0.3	0.0135	0.0254	Over-expressed
SIRT4	0.3	0.0167	0.0305	Over-expressed
COMT	0.3	0.0192	0.0346	Over-expressed
SLC10A3	0.3	0.02	0.0358	Over-expressed
PON2	0.3	0.0224	0.0397	Over-expressed
ADO	0.29	1.22E-05	5.59E-05	Over-expressed
HNRNPA3	0.29	1.45E-05	6.49E-05	Over-expressed
MRPL10	0.29	2.91E-05	1.19E-04	Over-expressed
C12ORF65	0.29	2.99E-05	1.22E-04	Over-expressed
COMMD9	0.29	4.33E-05	1.69E-04	Over-expressed
HNRNPM	0.29	5.91E-05	2.21E-04	Over-expressed
OXA1L	0.29	8.00E-05	2.89E-04	Over-expressed
ASB6	0.29	1.05E-04	3.65E-04	Over-expressed
THAP11	0.29	1.30E-04	4.42E-04	Over-expressed
VDAC1	0.29	1.52E-04	5.06E-04	Over-expressed
CDC123	0.29	1.90E-04	6.18E-04	Over-expressed
TBC1D10B	0.29	2.18E-04	6.91E-04	Over-expressed
BTF3L4	0.29	2.44E-04	7.63E-04	Over-expressed
KAT14	0.29	3.50E-04	1.05E-03	Over-expressed
RCCD1	0.29	3.58E-04	1.07E-03	Over-expressed
NELFCD	0.29	3.72E-04	1.11E-03	Over-expressed
NCBP2	0.29	4.27E-04	1.24E-03	Over-expressed
MNAT1	0.29	4.64E-04	1.34E-03	Over-expressed
GEMIN8	0.29	4.65E-04	1.34E-03	Over-expressed
PAGR1	0.29	4.77E-04	1.37E-03	Over-expressed
ATP6AP1	0.29	6.78E-04	1.86E-03	Over-expressed
SSU72	0.29	6.85E-04	1.87E-03	Over-expressed
FIBP	0.29	8.62E-04	2.29E-03	Over-expressed
NCL	0.29	9.31E-04	2.45E-03	Over-expressed
EMC8	0.29	9.65E-04	2.52E-03	Over-expressed
TMEM179B	0.29	9.81E-04	2.56E-03	Over-expressed
TAF9	0.29	1.00E-03	2.61E-03	Over-expressed
ATP6V0C	0.29	1.13E-03	2.89E-03	Over-expressed
SEPTIN9	0.29	1.19E-03	3.02E-03	Over-expressed

MRPL37	0.29	1.27E-03	3.20E-03	Over-expressed
ZNF668	0.29	1.48E-03	3.65E-03	Over-expressed
SNAPC2	0.29	1.61E-03	3.93E-03	Over-expressed
GTPBP4	0.29	1.63E-03	3.98E-03	Over-expressed
TAMM41	0.29	1.65E-03	4.02E-03	Over-expressed
THOC1	0.29	1.71E-03	4.16E-03	Over-expressed
EBNA1BP2	0.29	1.83E-03	4.40E-03	Over-expressed
CYBC1	0.29	1.89E-03	4.52E-03	Over-expressed
SNRNP40	0.29	2.47E-03	5.74E-03	Over-expressed
FASTK	0.29	2.58E-03	5.98E-03	Over-expressed
NPRL2	0.29	2.67E-03	6.15E-03	Over-expressed
TXN2	0.29	2.67E-03	6.16E-03	Over-expressed
COX16	0.29	2.74E-03	6.29E-03	Over-expressed
GNB2	0.29	2.77E-03	6.35E-03	Over-expressed
PSMG1	0.29	2.77E-03	6.37E-03	Over-expressed
TSR1	0.29	3.67E-03	8.10E-03	Over-expressed
STK3	0.29	3.98E-03	8.70E-03	Over-expressed
INO80B	0.29	4.03E-03	8.79E-03	Over-expressed
CHMP6	0.29	4.92E-03	0.0105	Over-expressed
CROCC	0.29	4.92E-03	0.0105	Over-expressed
TBC1D22B	0.29	5.36E-03	0.0113	Over-expressed
CMSS1	0.29	5.37E-03	0.0113	Over-expressed
LINC00847	0.29	6.95E-03	0.0142	Over-expressed
ATXN7L2	0.29	9.42E-03	0.0185	Over-expressed
LOC150776	0.29	9.53E-03	0.0187	Over-expressed
TMEM81	0.29	0.0106	0.0206	Over-expressed
H1FX	0.29	0.0108	0.0209	Over-expressed
POLR3K	0.29	0.0111	0.0214	Over-expressed
SRA1	0.29	0.0122	0.0233	Over-expressed
PMPCA	0.29	0.0132	0.0249	Over-expressed
PTCHD3P1	0.29	0.0137	0.0257	Over-expressed
COX7B	0.29	0.0177	0.0321	Over-expressed
RPP40	0.29	0.0288	0.0496	Over-expressed
EIF2B5	0.28	4.56E-07	3.26E-06	Over-expressed
RPP38	0.28	1.22E-06	7.55E-06	Over-expressed
SRP14	0.28	4.15E-06	2.19E-05	Over-expressed
PTGES3	0.28	1.13E-05	5.22E-05	Over-expressed
DDX39B	0.28	5.11E-05	1.94E-04	Over-expressed
MAD2L1BP	0.28	5.14E-05	1.96E-04	Over-expressed
CHCHD4	0.28	6.29E-05	2.33E-04	Over-expressed
GCN1	0.28	7.11E-05	2.60E-04	Over-expressed
DDX47	0.28	9.45E-05	3.34E-04	Over-expressed
CBWD2	0.28	2.26E-04	7.15E-04	Over-expressed
YAE1	0.28	3.63E-04	1.08E-03	Over-expressed
IP6K2	0.28	4.20E-04	1.23E-03	Over-expressed
AGPAT1	0.28	5.03E-04	1.43E-03	Over-expressed
ARAP1	0.28	5.32E-04	1.51E-03	Over-expressed
MRPS18C	0.28	5.82E-04	1.62E-03	Over-expressed
RNF166	0.28	8.56E-04	2.28E-03	Over-expressed
SET	0.28	9.59E-04	2.51E-03	Over-expressed
ZNF473	0.28	9.62E-04	2.52E-03	Over-expressed
MARS	0.28	1.05E-03	2.72E-03	Over-expressed
ZNF691	0.28	1.09E-03	2.79E-03	Over-expressed
STX5	0.28	1.15E-03	2.93E-03	Over-expressed
BABAM2	0.28	1.21E-03	3.07E-03	Over-expressed
HSPB11	0.28	1.31E-03	3.30E-03	Over-expressed
SMARCD1	0.28	1.71E-03	4.15E-03	Over-expressed

FAM53B	0.28	2.21E-03	5.23E-03	Over-expressed
URI1	0.28	2.41E-03	5.62E-03	Over-expressed
ZBTB45	0.28	2.59E-03	6.00E-03	Over-expressed
SCAMP4	0.28	3.38E-03	7.55E-03	Over-expressed
TAZ	0.28	3.87E-03	8.48E-03	Over-expressed
CIRBP	0.28	4.38E-03	9.48E-03	Over-expressed
LSM10	0.28	5.47E-03	0.0115	Over-expressed
ECSIT	0.28	6.08E-03	0.0126	Over-expressed
JUP	0.28	7.52E-03	0.0152	Over-expressed
RWDD2A	0.28	8.02E-03	0.0161	Over-expressed
SPOUT1	0.28	8.34E-03	0.0167	Over-expressed
C16ORF91	0.28	0.0106	0.0205	Over-expressed
IKBIP	0.28	0.0113	0.0217	Over-expressed
SPIN2B	0.28	0.0114	0.0218	Over-expressed
PRDX2	0.28	0.0123	0.0234	Over-expressed
PHF21A	0.28	0.0125	0.0237	Over-expressed
NDUFA4	0.28	0.0153	0.0283	Over-expressed
FOXK1	0.28	0.0184	0.0333	Over-expressed
UCHL3	0.28	0.0191	0.0344	Over-expressed
ATP6V1C2	0.28	0.0214	0.0381	Over-expressed
HEBP2	0.28	0.0239	0.0421	Over-expressed
HNRNPD	0.27	2.89E-05	1.18E-04	Over-expressed
GSK3A	0.27	6.71E-05	2.46E-04	Over-expressed
PRPF19	0.27	8.02E-05	2.89E-04	Over-expressed
NDUFB4	0.27	9.77E-05	3.44E-04	Over-expressed
NECAP2	0.27	1.11E-04	3.84E-04	Over-expressed
SUPT4H1	0.27	1.26E-04	4.30E-04	Over-expressed
RUFY1	0.27	2.34E-04	7.35E-04	Over-expressed
MAGOH	0.27	2.51E-04	7.82E-04	Over-expressed
MTCH1	0.27	2.52E-04	7.85E-04	Over-expressed
YIPF3	0.27	2.63E-04	8.14E-04	Over-expressed
APEX1	0.27	2.86E-04	8.76E-04	Over-expressed
MFF	0.27	3.93E-04	1.16E-03	Over-expressed
ARPC2	0.27	4.30E-04	1.25E-03	Over-expressed
H2AFY	0.27	4.46E-04	1.29E-03	Over-expressed
LMAN2L	0.27	5.09E-04	1.45E-03	Over-expressed
MRPS14	0.27	6.85E-04	1.87E-03	Over-expressed
EXOC3	0.27	7.84E-04	2.11E-03	Over-expressed
CCDC97	0.27	8.75E-04	2.32E-03	Over-expressed
STARD3	0.27	9.33E-04	2.45E-03	Over-expressed
CEP95	0.27	1.10E-03	2.83E-03	Over-expressed
DEF8	0.27	1.25E-03	3.16E-03	Over-expressed
BRAT1	0.27	1.58E-03	3.88E-03	Over-expressed
ZSWIM3	0.27	2.17E-03	5.13E-03	Over-expressed
TOE1	0.27	2.22E-03	5.24E-03	Over-expressed
POLR3H	0.27	2.26E-03	5.32E-03	Over-expressed
CFAP298	0.27	2.88E-03	6.57E-03	Over-expressed
SLC39A1	0.27	3.00E-03	6.81E-03	Over-expressed
STX10	0.27	3.05E-03	6.91E-03	Over-expressed
HHEX	0.27	3.09E-03	6.99E-03	Over-expressed
CIZ1	0.27	3.24E-03	7.29E-03	Over-expressed
ZNF205	0.27	4.47E-03	9.65E-03	Over-expressed
METTL22	0.27	4.65E-03	9.99E-03	Over-expressed
RTN4IP1	0.27	4.87E-03	0.0104	Over-expressed
DSN1	0.27	4.93E-03	0.0105	Over-expressed
ABCC5	0.27	6.32E-03	0.0131	Over-expressed
POP1	0.27	7.67E-03	0.0155	Over-expressed

HAUS5	0.27	8.36E-03	0.0167	Over-expressed
UCK1	0.27	8.84E-03	0.0175	Over-expressed
MTERF3	0.27	9.03E-03	0.0179	Over-expressed
KBTBD3	0.27	9.20E-03	0.0182	Over-expressed
COX10	0.27	9.48E-03	0.0186	Over-expressed
NEK6	0.27	0.011	0.0212	Over-expressed
CSNK2A1	0.27	0.011	0.0212	Over-expressed
TNIP1	0.27	0.0114	0.0219	Over-expressed
SLC20A1	0.27	0.0122	0.0232	Over-expressed
IPO4	0.27	0.0141	0.0264	Over-expressed
TOMM40L	0.27	0.0177	0.0321	Over-expressed
POLR1E	0.27	0.018	0.0327	Over-expressed
NASP	0.27	0.0181	0.0328	Over-expressed
CSNK1E	0.27	0.0184	0.0333	Over-expressed
ZNF8	0.27	0.0195	0.0351	Over-expressed
CCDC130	0.27	0.02	0.0358	Over-expressed
CD27-AS1	0.27	0.0201	0.036	Over-expressed
MSTO1	0.27	0.0205	0.0366	Over-expressed
DHRS7B	0.27	0.0222	0.0393	Over-expressed
CAMKMT	0.27	0.025	0.0437	Over-expressed
PNP	0.27	0.027	0.0468	Over-expressed
GPI	0.27	0.0277	0.048	Over-expressed
ACTR1A	0.26	4.81E-06	2.48E-05	Over-expressed
FAM204A	0.26	5.95E-06	2.99E-05	Over-expressed
HINFP	0.26	4.75E-05	1.83E-04	Over-expressed
HARS2	0.26	1.38E-04	4.66E-04	Over-expressed
TRIT1	0.26	1.39E-04	4.66E-04	Over-expressed
TMEM115	0.26	1.69E-04	5.55E-04	Over-expressed
IER3IP1	0.26	2.49E-04	7.76E-04	Over-expressed
SMPD4	0.26	2.56E-04	7.96E-04	Over-expressed
HPS6	0.26	2.77E-04	8.53E-04	Over-expressed
GOLGA3	0.26	3.12E-04	9.48E-04	Over-expressed
ABT1	0.26	3.81E-04	1.13E-03	Over-expressed
SRF	0.26	6.23E-04	1.72E-03	Over-expressed
RAB11A	0.26	7.04E-04	1.92E-03	Over-expressed
PGAM1	0.26	9.17E-04	2.42E-03	Over-expressed
TMEM199	0.26	9.23E-04	2.43E-03	Over-expressed
PNPLA6	0.26	9.61E-04	2.52E-03	Over-expressed
NSMCE4A	0.26	1.05E-03	2.71E-03	Over-expressed
EDEM2	0.26	1.06E-03	2.73E-03	Over-expressed
SRP19	0.26	1.10E-03	2.82E-03	Over-expressed
TBL2	0.26	1.18E-03	2.99E-03	Over-expressed
SGTA	0.26	1.18E-03	3.00E-03	Over-expressed
RAD23A	0.26	1.23E-03	3.10E-03	Over-expressed
TRIAP1	0.26	1.34E-03	3.35E-03	Over-expressed
ERH	0.26	1.35E-03	3.38E-03	Over-expressed
DRAM2	0.26	1.41E-03	3.51E-03	Over-expressed
CCDC22	0.26	1.81E-03	4.37E-03	Over-expressed
ILF3	0.26	1.85E-03	4.44E-03	Over-expressed
TMEM41A	0.26	2.07E-03	4.90E-03	Over-expressed
DHPS	0.26	2.28E-03	5.35E-03	Over-expressed
CCT8	0.26	2.60E-03	6.01E-03	Over-expressed
ZMAT2	0.26	2.92E-03	6.64E-03	Over-expressed
NDUFAF3	0.26	3.10E-03	7.01E-03	Over-expressed
NOLC1	0.26	3.37E-03	7.55E-03	Over-expressed
MED28	0.26	3.52E-03	7.83E-03	Over-expressed
SH3GL1	0.26	3.68E-03	8.12E-03	Over-expressed

ZNF250	0.26	3.80E-03	8.34E-03	Over-expressed
RETREG3	0.26	4.83E-03	0.0103	Over-expressed
LTBR	0.26	4.86E-03	0.0104	Over-expressed
FAM222B	0.26	5.92E-03	0.0123	Over-expressed
MRPL58	0.26	6.15E-03	0.0128	Over-expressed
WDR12	0.26	6.34E-03	0.0131	Over-expressed
BIN3	0.26	7.54E-03	0.0153	Over-expressed
ZBTB48	0.26	8.27E-03	0.0166	Over-expressed
ARRDC1	0.26	8.42E-03	0.0168	Over-expressed
TEX10	0.26	0.0108	0.0209	Over-expressed
FBRSL1	0.26	0.0112	0.0215	Over-expressed
SMN2	0.26	0.0125	0.0237	Over-expressed
CYB561D2	0.26	0.0134	0.0253	Over-expressed
NDUFV1	0.26	0.0136	0.0256	Over-expressed
TRAM1	0.26	0.0147	0.0273	Over-expressed
FAM98C	0.26	0.0148	0.0275	Over-expressed
NFKBIB	0.26	0.0189	0.0341	Over-expressed
NR1H3	0.26	0.0195	0.035	Over-expressed
SRD5A3	0.26	0.0196	0.0352	Over-expressed
SRPK1	0.26	0.0197	0.0353	Over-expressed
ZNF74	0.26	0.0208	0.0372	Over-expressed
DBI	0.26	0.0212	0.0377	Over-expressed
MAGED2	0.26	0.0223	0.0395	Over-expressed
ILF3-DT	0.26	0.0254	0.0443	Over-expressed
CD83	0.26	0.0258	0.0449	Over-expressed
SCMH1	0.26	0.0284	0.049	Over-expressed
VRK1	0.26	0.0288	0.0495	Over-expressed
SAR1A	0.25	9.57E-06	4.53E-05	Over-expressed
ATG13	0.25	1.13E-05	5.22E-05	Over-expressed
EDC3	0.25	2.19E-05	9.27E-05	Over-expressed
OTUD5	0.25	5.56E-05	2.10E-04	Over-expressed
BAG6	0.25	7.04E-05	2.57E-04	Over-expressed
EWSR1	0.25	1.13E-04	3.91E-04	Over-expressed
GPN1	0.25	1.94E-04	6.28E-04	Over-expressed
CENPT	0.25	2.09E-04	6.67E-04	Over-expressed
COP1	0.25	2.73E-04	8.42E-04	Over-expressed
TAF11	0.25	2.86E-04	8.75E-04	Over-expressed
LARS	0.25	3.10E-04	9.41E-04	Over-expressed
R3HCC1L	0.25	3.56E-04	1.06E-03	Over-expressed
ELOVL1	0.25	3.60E-04	1.07E-03	Over-expressed
MED8	0.25	8.95E-04	2.36E-03	Over-expressed
SFPQ	0.25	9.64E-04	2.52E-03	Over-expressed
ZNF271P	0.25	9.73E-04	2.54E-03	Over-expressed
TMEM14C	0.25	9.73E-04	2.54E-03	Over-expressed
CALM2	0.25	1.08E-03	2.78E-03	Over-expressed
MRPS35	0.25	1.15E-03	2.94E-03	Over-expressed
KMT5A	0.25	1.24E-03	3.13E-03	Over-expressed
TRAPPC12	0.25	1.28E-03	3.22E-03	Over-expressed
ATP6V1E1	0.25	1.35E-03	3.37E-03	Over-expressed
PYCR2	0.25	1.40E-03	3.48E-03	Over-expressed
SEC13	0.25	1.59E-03	3.90E-03	Over-expressed
CLN6	0.25	1.63E-03	3.97E-03	Over-expressed
UTP18	0.25	1.68E-03	4.08E-03	Over-expressed
TMEM222	0.25	1.93E-03	4.62E-03	Over-expressed
SFT2D3	0.25	1.95E-03	4.66E-03	Over-expressed
ZBTB9	0.25	2.02E-03	4.81E-03	Over-expressed
PHC2	0.25	2.17E-03	5.13E-03	Over-expressed

TMEM250	0.25	2.28E-03	5.35E-03	Over-expressed
COG2	0.25	2.94E-03	6.69E-03	Over-expressed
DDX50	0.25	3.14E-03	7.09E-03	Over-expressed
ERGIC2	0.25	3.88E-03	8.51E-03	Over-expressed
RPUSD4	0.25	4.17E-03	9.07E-03	Over-expressed
E2F4	0.25	4.42E-03	9.55E-03	Over-expressed
YJU2	0.25	5.01E-03	0.0106	Over-expressed
PISD	0.25	5.50E-03	0.0115	Over-expressed
PHF5A	0.25	5.68E-03	0.0119	Over-expressed
TSPAN17	0.25	6.32E-03	0.0131	Over-expressed
PTPA	0.25	6.52E-03	0.0134	Over-expressed
PMS2P1	0.25	6.99E-03	0.0143	Over-expressed
STK24	0.25	8.37E-03	0.0167	Over-expressed
AMD1	0.25	8.54E-03	0.017	Over-expressed
HMBS	0.25	9.24E-03	0.0182	Over-expressed
DCP2	0.25	9.31E-03	0.0183	Over-expressed
SFR1	0.25	0.0102	0.0198	Over-expressed
DTNB	0.25	0.0108	0.021	Over-expressed
PWP2	0.25	0.0121	0.0231	Over-expressed
PIM3	0.25	0.0128	0.0242	Over-expressed
OFD1	0.25	0.0129	0.0244	Over-expressed
SUV39H1	0.25	0.0136	0.0255	Over-expressed
HDAC5	0.25	0.0141	0.0264	Over-expressed
TMEM68	0.25	0.0169	0.0309	Over-expressed
TOM1	0.25	0.0189	0.0341	Over-expressed
PHIP	0.25	0.0212	0.0378	Over-expressed
SLC50A1	0.25	0.023	0.0406	Over-expressed
DNAJC30	0.25	0.0247	0.0433	Over-expressed
HNRNPL	0.24	4.10E-05	1.61E-04	Over-expressed
COMMD3	0.24	6.41E-05	2.37E-04	Over-expressed
NAT10	0.24	2.26E-04	7.14E-04	Over-expressed
CHTOP	0.24	2.29E-04	7.22E-04	Over-expressed
GTF2A2	0.24	2.43E-04	7.62E-04	Over-expressed
UBE2R2	0.24	2.55E-04	7.92E-04	Over-expressed
FAM32A	0.24	2.67E-04	8.24E-04	Over-expressed
ACTR8	0.24	3.77E-04	1.12E-03	Over-expressed
GPKOW	0.24	5.72E-04	1.60E-03	Over-expressed
HARS	0.24	6.80E-04	1.87E-03	Over-expressed
ELP6	0.24	1.07E-03	2.75E-03	Over-expressed
SKP1	0.24	1.27E-03	3.19E-03	Over-expressed
CRIPT	0.24	1.39E-03	3.47E-03	Over-expressed
NUDT3	0.24	1.49E-03	3.67E-03	Over-expressed
TEPSIN	0.24	1.59E-03	3.90E-03	Over-expressed
GNL2	0.24	1.91E-03	4.56E-03	Over-expressed
FTSJ3	0.24	1.91E-03	4.57E-03	Over-expressed
EXOSC3	0.24	2.38E-03	5.55E-03	Over-expressed
WBP1	0.24	2.52E-03	5.84E-03	Over-expressed
TSFM	0.24	2.54E-03	5.88E-03	Over-expressed
MORC2	0.24	2.67E-03	6.15E-03	Over-expressed
ZCCHC17	0.24	2.85E-03	6.53E-03	Over-expressed
TELO2	0.24	3.24E-03	7.29E-03	Over-expressed
INPP5A	0.24	3.93E-03	8.61E-03	Over-expressed
SARS	0.24	4.33E-03	9.39E-03	Over-expressed
ZCCHC10	0.24	4.75E-03	0.0102	Over-expressed
HNRNPAB	0.24	5.00E-03	0.0106	Over-expressed
POLG2	0.24	5.18E-03	0.011	Over-expressed
GNPAT	0.24	6.27E-03	0.013	Over-expressed

ZNF766	0.24	6.48E-03	0.0133	Over-expressed
SLC41A3	0.24	6.83E-03	0.014	Over-expressed
LIAS	0.24	6.85E-03	0.014	Over-expressed
PSPC1	0.24	8.58E-03	0.0171	Over-expressed
MGME1	0.24	9.24E-03	0.0182	Over-expressed
SPNS1	0.24	9.68E-03	0.019	Over-expressed
DYNC2LI1	0.24	9.80E-03	0.0192	Over-expressed
ADCK2	0.24	0.0103	0.0201	Over-expressed
ING4	0.24	0.0105	0.0204	Over-expressed
UTP4	0.24	0.0105	0.0204	Over-expressed
CEP250	0.24	0.0107	0.0206	Over-expressed
METTL18	0.24	0.0117	0.0224	Over-expressed
ATP13A1	0.24	0.0118	0.0225	Over-expressed
RMDN1	0.24	0.0138	0.0258	Over-expressed
TIMM44	0.24	0.0143	0.0266	Over-expressed
GLO1	0.24	0.0155	0.0287	Over-expressed
RTL10	0.24	0.0165	0.0303	Over-expressed
UROD	0.24	0.0205	0.0366	Over-expressed
CHMP3	0.24	0.0209	0.0373	Over-expressed
LDHA	0.24	0.0218	0.0387	Over-expressed
ZNF26	0.24	0.0223	0.0395	Over-expressed
STUB1	0.24	0.0239	0.042	Over-expressed
HNRNPC	0.23	1.26E-05	5.74E-05	Over-expressed
IK	0.23	4.53E-05	1.75E-04	Over-expressed
STRADA	0.23	5.19E-05	1.97E-04	Over-expressed
UTP6	0.23	1.04E-03	2.69E-03	Over-expressed
MORF4L1	0.23	1.24E-03	3.14E-03	Over-expressed
RAMAC	0.23	1.28E-03	3.22E-03	Over-expressed
RBM8A	0.23	1.51E-03	3.72E-03	Over-expressed
GTF2F1	0.23	1.92E-03	4.60E-03	Over-expressed
RANBP3	0.23	2.18E-03	5.16E-03	Over-expressed
TRIM27	0.23	2.45E-03	5.70E-03	Over-expressed
USP5	0.23	2.51E-03	5.83E-03	Over-expressed
KRI1	0.23	2.66E-03	6.15E-03	Over-expressed
DDX55	0.23	2.76E-03	6.34E-03	Over-expressed
LAS1L	0.23	3.40E-03	7.59E-03	Over-expressed
MTHFSD	0.23	3.51E-03	7.81E-03	Over-expressed
ESS2	0.23	3.75E-03	8.25E-03	Over-expressed
PPP6R1	0.23	4.37E-03	9.46E-03	Over-expressed
STX4	0.23	5.15E-03	0.0109	Over-expressed
FOXK2	0.23	5.16E-03	0.0109	Over-expressed
GNRHR2	0.23	5.21E-03	0.011	Over-expressed
CCZ1	0.23	5.43E-03	0.0114	Over-expressed
EPN1	0.23	5.61E-03	0.0117	Over-expressed
CDK5RAP3	0.23	7.40E-03	0.015	Over-expressed
CMC1	0.23	7.69E-03	0.0155	Over-expressed
TMEM69	0.23	7.75E-03	0.0156	Over-expressed
PIGH	0.23	7.92E-03	0.0159	Over-expressed
AHSA1	0.23	0.0107	0.0206	Over-expressed
SETD4	0.23	0.0108	0.0208	Over-expressed
TMEM60	0.23	0.0113	0.0216	Over-expressed
PPOX	0.23	0.0113	0.0217	Over-expressed
CEP164	0.23	0.0142	0.0265	Over-expressed
HNRNPH1	0.23	0.0144	0.0269	Over-expressed
CAMTA1	0.23	0.0184	0.0333	Over-expressed
WARS2	0.23	0.0196	0.0352	Over-expressed
PELO	0.23	0.02	0.0359	Over-expressed

ENTR1	0.23	0.0203	0.0364	Over-expressed
ATPAF2	0.23	0.0234	0.0412	Over-expressed
POLR2J3	0.23	0.0253	0.0442	Over-expressed
EEF2	0.23	0.0255	0.0444	Over-expressed
ZNF767P	0.23	0.0285	0.0491	Over-expressed
DNAJB12	0.22	2.14E-05	9.12E-05	Over-expressed
PTBP1	0.22	1.11E-04	3.83E-04	Over-expressed
MRPL30	0.22	2.02E-04	6.49E-04	Over-expressed
RNF220	0.22	3.44E-04	1.03E-03	Over-expressed
DNTTIP2	0.22	9.72E-04	2.54E-03	Over-expressed
XPNPEP1	0.22	1.01E-03	2.62E-03	Over-expressed
CDC16	0.22	1.15E-03	2.93E-03	Over-expressed
TSN	0.22	2.45E-03	5.69E-03	Over-expressed
STMP1	0.22	3.65E-03	8.05E-03	Over-expressed
WIPI2	0.22	3.86E-03	8.46E-03	Over-expressed
SIRT3	0.22	4.56E-03	9.82E-03	Over-expressed
MAN2B1	0.22	5.51E-03	0.0116	Over-expressed
PIGC	0.22	5.52E-03	0.0116	Over-expressed
STX6	0.22	5.53E-03	0.0116	Over-expressed
AP2M1	0.22	5.53E-03	0.0116	Over-expressed
CANT1	0.22	6.09E-03	0.0126	Over-expressed
KNOP1	0.22	6.50E-03	0.0134	Over-expressed
CNOT3	0.22	6.79E-03	0.0139	Over-expressed
RPS6KB2	0.22	6.92E-03	0.0142	Over-expressed
HNRNPDL	0.22	7.05E-03	0.0144	Over-expressed
SMIM7	0.22	7.33E-03	0.0149	Over-expressed
ZNF75A	0.22	8.00E-03	0.0161	Over-expressed
SNUPN	0.22	8.08E-03	0.0162	Over-expressed
FTSJ1	0.22	8.58E-03	0.0171	Over-expressed
SLC39A13	0.22	9.12E-03	0.018	Over-expressed
EHD1	0.22	9.16E-03	0.0181	Over-expressed
TXNDC12	0.22	9.49E-03	0.0186	Over-expressed
NIP7	0.22	0.0104	0.0202	Over-expressed
MEPCE	0.22	0.0106	0.0206	Over-expressed
COG4	0.22	0.011	0.0212	Over-expressed
DCAF4	0.22	0.0113	0.0216	Over-expressed
WDR5	0.22	0.0127	0.0241	Over-expressed
CZIB	0.22	0.0128	0.0242	Over-expressed
C10ORF88	0.22	0.0144	0.0269	Over-expressed
PSMB2	0.22	0.0145	0.027	Over-expressed
MED15	0.22	0.0151	0.0279	Over-expressed
GPS1	0.22	0.0153	0.0283	Over-expressed
C22ORF39	0.22	0.0157	0.029	Over-expressed
ALG5	0.22	0.0167	0.0306	Over-expressed
NUP43	0.22	0.0175	0.0318	Over-expressed
ZNF565	0.22	0.0179	0.0324	Over-expressed
ZNF764	0.22	0.0184	0.0333	Over-expressed
NUP88	0.22	0.019	0.0343	Over-expressed
METTL21A	0.22	0.0236	0.0415	Over-expressed
ZNF84	0.22	0.025	0.0437	Over-expressed
AP1S1	0.22	0.0253	0.0441	Over-expressed
TAPBP	0.22	0.026	0.0453	Over-expressed
SLC26A11	0.22	0.0281	0.0486	Over-expressed
TARDBP	0.21	6.49E-07	4.43E-06	Over-expressed
YTHDF1	0.21	9.38E-05	3.32E-04	Over-expressed
SUMO1	0.21	1.44E-04	4.80E-04	Over-expressed
TLK2	0.21	1.58E-04	5.24E-04	Over-expressed

BUD13	0.21	1.88E-04	6.10E-04	Over-expressed
METTL17	0.21	3.75E-04	1.11E-03	Over-expressed
CNPPD1	0.21	4.48E-04	1.30E-03	Over-expressed
CNOT2	0.21	5.71E-04	1.60E-03	Over-expressed
AKAP8	0.21	6.50E-04	1.79E-03	Over-expressed
TRPC4AP	0.21	9.19E-04	2.42E-03	Over-expressed
SMARCE1	0.21	1.06E-03	2.73E-03	Over-expressed
TXNDC9	0.21	1.34E-03	3.37E-03	Over-expressed
C12ORF4	0.21	1.40E-03	3.49E-03	Over-expressed
CIR1	0.21	1.41E-03	3.51E-03	Over-expressed
TRAPPC3	0.21	2.02E-03	4.80E-03	Over-expressed
EXOC4	0.21	2.23E-03	5.26E-03	Over-expressed
TEFM	0.21	2.52E-03	5.85E-03	Over-expressed
GMEB2	0.21	3.07E-03	6.94E-03	Over-expressed
U2AF2	0.21	3.67E-03	8.10E-03	Over-expressed
SMAP1	0.21	3.93E-03	8.61E-03	Over-expressed
EIF2D	0.21	4.15E-03	9.03E-03	Over-expressed
UBAC1	0.21	4.19E-03	9.10E-03	Over-expressed
C1ORF52	0.21	4.40E-03	9.51E-03	Over-expressed
FAM53C	0.21	4.84E-03	0.0103	Over-expressed
RABIF	0.21	5.18E-03	0.011	Over-expressed
XRRA1	0.21	5.51E-03	0.0116	Over-expressed
TNIP2	0.21	5.80E-03	0.0121	Over-expressed
RABGGTA	0.21	6.26E-03	0.0129	Over-expressed
HNRNPA3P1	0.21	7.00E-03	0.0143	Over-expressed
CCDC43	0.21	7.17E-03	0.0146	Over-expressed
SGSM3	0.21	8.34E-03	0.0167	Over-expressed
VPS4A	0.21	0.0127	0.0241	Over-expressed
NDUFB5	0.21	0.0128	0.0242	Over-expressed
MRPL18	0.21	0.0138	0.0258	Over-expressed
GDI1	0.21	0.0143	0.0266	Over-expressed
GSS	0.21	0.0154	0.0284	Over-expressed
UBL4A	0.21	0.0193	0.0348	Over-expressed
RHOT2	0.21	0.0211	0.0376	Over-expressed
ZDHHC18	0.21	0.0222	0.0393	Over-expressed
YWHAQ	0.21	0.0242	0.0425	Over-expressed
TDG	0.21	0.0252	0.0439	Over-expressed
CIAO1	0.2	4.93E-05	1.89E-04	Over-expressed
TIAL1	0.2	4.80E-04	1.38E-03	Over-expressed
BRAP	0.2	5.59E-04	1.57E-03	Over-expressed
MAPKAPK5	0.2	7.59E-04	2.05E-03	Over-expressed
ORMDL1	0.2	1.23E-03	3.10E-03	Over-expressed
PRR14	0.2	1.23E-03	3.12E-03	Over-expressed
SRSF6	0.2	1.34E-03	3.35E-03	Over-expressed
SAFB	0.2	1.69E-03	4.10E-03	Over-expressed
DYNC1LI1	0.2	3.06E-03	6.94E-03	Over-expressed
ZNF335	0.2	3.77E-03	8.28E-03	Over-expressed
MTMR14	0.2	4.78E-03	0.0102	Over-expressed
ZFYVE27	0.2	4.92E-03	0.0105	Over-expressed
RBM6	0.2	4.98E-03	0.0106	Over-expressed
TBC1D25	0.2	5.11E-03	0.0108	Over-expressed
RFT1	0.2	5.42E-03	0.0114	Over-expressed
RPL7L1	0.2	5.53E-03	0.0116	Over-expressed
PRKAB1	0.2	5.90E-03	0.0123	Over-expressed
HGS	0.2	7.14E-03	0.0145	Over-expressed
CRTC2	0.2	7.67E-03	0.0155	Over-expressed
PSMF1	0.2	8.63E-03	0.0172	Over-expressed

EIF4A3	0.2	8.72E-03	0.0173	Over-expressed
RCC1L	0.2	9.55E-03	0.0188	Over-expressed
C7ORF26	0.2	9.86E-03	0.0193	Over-expressed
PRKRA	0.2	0.0105	0.0205	Over-expressed
UBE2E1	0.2	0.0129	0.0244	Over-expressed
SFSWAP	0.2	0.0138	0.0259	Over-expressed
TMA16	0.2	0.0154	0.0284	Over-expressed
GTPBP3	0.2	0.0155	0.0286	Over-expressed
WDR43	0.2	0.0157	0.029	Over-expressed
NKAP	0.2	0.0174	0.0317	Over-expressed
AIDA	0.2	0.0179	0.0326	Over-expressed
TSPAN31	0.2	0.0182	0.0329	Over-expressed
SSRP1	0.2	0.019	0.0343	Over-expressed
LSM6	0.2	0.0264	0.0459	Over-expressed
POMT1	0.2	0.0291	0.05	Over-expressed
ISY1	0.19	9.57E-04	2.51E-03	Over-expressed
UTP11	0.19	3.23E-03	7.26E-03	Over-expressed
ANAPC5	0.19	3.53E-03	7.83E-03	Over-expressed
NGDN	0.19	4.14E-03	9.02E-03	Over-expressed
METTL6	0.19	4.91E-03	0.0105	Over-expressed
ARFGAP2	0.19	5.69E-03	0.0119	Over-expressed
OSER1	0.19	7.64E-03	0.0154	Over-expressed
AAMP	0.19	7.91E-03	0.0159	Over-expressed
APTX	0.19	8.95E-03	0.0177	Over-expressed
RABGEF1	0.19	0.0108	0.0209	Over-expressed
KTI12	0.19	0.011	0.0212	Over-expressed
RXYLT1	0.19	0.0116	0.0222	Over-expressed
EDC4	0.19	0.0117	0.0223	Over-expressed
RECQL5	0.19	0.013	0.0245	Over-expressed
SCAF1	0.19	0.0133	0.0251	Over-expressed
UBC	0.19	0.0137	0.0256	Over-expressed
LYPLA2P1	0.19	0.0169	0.0309	Over-expressed
TARS	0.19	0.0178	0.0324	Over-expressed
SGSH	0.19	0.0183	0.0332	Over-expressed
TOR2A	0.19	0.021	0.0374	Over-expressed
PKIG	0.19	0.0211	0.0376	Over-expressed
PSMA6	0.19	0.0218	0.0386	Over-expressed
MRPS27	0.19	0.0224	0.0396	Over-expressed
WDR45	0.19	0.0262	0.0456	Over-expressed
PSMC3	0.19	0.0274	0.0475	Over-expressed
METTL8	0.19	0.0287	0.0494	Over-expressed
PPP2R3C	0.19	0.0289	0.0498	Over-expressed
ARMH3	0.18	3.61E-04	1.08E-03	Over-expressed
VPS33B	0.18	2.68E-03	6.17E-03	Over-expressed
SETD6	0.18	2.86E-03	6.53E-03	Over-expressed
RAB1B	0.18	4.45E-03	9.62E-03	Over-expressed
BLOC1S2	0.18	4.78E-03	0.0102	Over-expressed
SRSF7	0.18	5.00E-03	0.0106	Over-expressed
RBM19	0.18	0.0114	0.0219	Over-expressed
GRSF1	0.18	0.0117	0.0224	Over-expressed
CDC37	0.18	0.0118	0.0226	Over-expressed
ZNF282	0.18	0.0124	0.0235	Over-expressed
SPCS2	0.18	0.013	0.0245	Over-expressed
IST1	0.18	0.0148	0.0275	Over-expressed
PRKCSH	0.18	0.0165	0.0303	Over-expressed
PSMG2	0.18	0.0171	0.0312	Over-expressed
DDX31	0.18	0.0189	0.034	Over-expressed

PSMD7	0.18	0.0209	0.0373	Over-expressed
MLF2	0.18	0.0217	0.0385	Over-expressed
SLC35B1	0.18	0.022	0.039	Over-expressed
HMG20B	0.18	0.022	0.0391	Over-expressed
KCMF1	0.17	4.63E-04	1.34E-03	Over-expressed
CCDC174	0.17	2.15E-03	5.08E-03	Over-expressed
GTF2H4	0.17	7.89E-03	0.0159	Over-expressed
RBM39	0.17	0.0111	0.0213	Over-expressed
ZNF263	0.17	0.0136	0.0255	Over-expressed
MTRF1L	0.17	0.0145	0.0269	Over-expressed
OSGEP	0.17	0.0145	0.027	Over-expressed
NOL11	0.17	0.0162	0.0298	Over-expressed
ZBTB17	0.17	0.0211	0.0376	Over-expressed
DROSHA	0.17	0.0227	0.04	Over-expressed
NAE1	0.17	0.0235	0.0414	Over-expressed
ARHGDIA	0.17	0.0245	0.0429	Over-expressed
PTPN18	0.17	0.0248	0.0434	Over-expressed
PKNOX1	0.16	4.18E-03	9.08E-03	Over-expressed
DAXX	0.16	5.02E-03	0.0107	Over-expressed
GPBP1	0.16	5.79E-03	0.0121	Over-expressed
GDI2	0.16	6.71E-03	0.0138	Over-expressed
PSMA1	0.16	7.06E-03	0.0144	Over-expressed
CLK3	0.16	0.01	0.0196	Over-expressed
NKIRAS2	0.16	0.0152	0.0281	Over-expressed
TPM3	0.16	0.0159	0.0292	Over-expressed
CSTF1	0.16	0.017	0.031	Over-expressed
SPATA2	0.16	0.0204	0.0365	Over-expressed
HMGXB3	0.16	0.0207	0.037	Over-expressed
CSK	0.16	0.0224	0.0397	Over-expressed
WDR89	0.16	0.0247	0.0433	Over-expressed
DPH3	0.16	0.0254	0.0444	Over-expressed
EXOC6	0.16	0.0271	0.047	Over-expressed
ZNF3	0.16	0.0278	0.0481	Over-expressed
C2ORF49	0.16	0.028	0.0484	Over-expressed
MRPL42	0.16	0.0288	0.0496	Over-expressed
RBM45	0.15	6.20E-03	0.0128	Over-expressed
MUS81	0.15	8.41E-03	0.0168	Over-expressed
NOL10	0.15	8.90E-03	0.0176	Over-expressed
AP2A2	0.15	0.0116	0.0222	Over-expressed
HNRNPU	0.15	0.014	0.0262	Over-expressed
NAIF1	0.15	0.0182	0.033	Over-expressed
AAR2	0.15	0.02	0.0358	Over-expressed
BUB3	0.15	0.021	0.0375	Over-expressed
NSUN4	0.15	0.0244	0.0428	Over-expressed
COA5	0.15	0.0286	0.0493	Over-expressed
BET1L	0.14	0.014	0.0262	Over-expressed
TRIP4	0.14	0.0142	0.0266	Over-expressed
VPS29	0.14	0.0156	0.0288	Over-expressed
ANAPC16	0.14	0.0225	0.0397	Over-expressed
METTL2A	0.14	0.0285	0.0491	Over-expressed
EIF2B1	0.13	0.0223	0.0395	Over-expressed
XRCC5	0.13	0.0238	0.0418	Over-expressed
LSM12	0.13	0.0276	0.0478	Over-expressed
RBM14	0.12	0.0125	0.0238	Over-expressed
CEP63	-0.12	0.0254	0.0444	Under-expressed
CUL3	-0.13	0.0278	0.0481	Under-expressed
HNRNPK	-0.14	0.0185	0.0335	Under-expressed

PPID	-0.14	0.0197	0.0354	Under-expressed
TOMM70	-0.14	0.026	0.0452	Under-expressed
TBC1D15	-0.14	0.0268	0.0465	Under-expressed
PSMD11	-0.14	0.0271	0.047	Under-expressed
QRICH1	-0.14	0.0284	0.049	Under-expressed
SLC25A17	-0.15	0.0134	0.0253	Under-expressed
GSPT1	-0.15	0.0168	0.0308	Under-expressed
SYNRG	-0.15	0.0237	0.0417	Under-expressed
C16ORF72	-0.16	7.87E-03	0.0158	Under-expressed
TFG	-0.16	0.0127	0.024	Under-expressed
ARL8B	-0.16	0.0148	0.0274	Under-expressed
C2ORF42	-0.16	0.0165	0.0302	Under-expressed
VPS39	-0.16	0.0219	0.0389	Under-expressed
MIEF1	-0.16	0.0252	0.0439	Under-expressed
ZNF592	-0.16	0.0252	0.044	Under-expressed
SENP5	-0.17	7.22E-03	0.0147	Under-expressed
ZFAND6	-0.17	7.37E-03	0.0149	Under-expressed
TINF2	-0.17	0.0119	0.0228	Under-expressed
COPG1	-0.17	0.0123	0.0235	Under-expressed
NSUN2	-0.17	0.0129	0.0244	Under-expressed
MAIP1	-0.17	0.0145	0.0269	Under-expressed
ATG16L1	-0.17	0.0149	0.0276	Under-expressed
PAK2	-0.17	0.0152	0.0281	Under-expressed
DNAJC21	-0.17	0.018	0.0327	Under-expressed
CHTF8	-0.17	0.0212	0.0377	Under-expressed
IPO8	-0.17	0.0215	0.0382	Under-expressed
ARF6	-0.17	0.0224	0.0397	Under-expressed
DHDDS	-0.17	0.0241	0.0423	Under-expressed
SBDS	-0.17	0.025	0.0438	Under-expressed
N4BP1	-0.18	1.54E-03	3.79E-03	Under-expressed
PPP6C	-0.18	2.23E-03	5.26E-03	Under-expressed
BCL2L13	-0.18	3.32E-03	7.44E-03	Under-expressed
DHX8	-0.18	3.47E-03	7.73E-03	Under-expressed
CNOT4	-0.18	3.52E-03	7.81E-03	Under-expressed
ANKRD40	-0.18	7.85E-03	0.0158	Under-expressed
BCAS2	-0.18	9.67E-03	0.019	Under-expressed
ARHGAP17	-0.18	0.011	0.0212	Under-expressed
EML3	-0.18	0.0119	0.0226	Under-expressed
CYB561A3	-0.18	0.013	0.0245	Under-expressed
GUF1	-0.18	0.0134	0.0253	Under-expressed
USP10	-0.18	0.014	0.0262	Under-expressed
HUS1	-0.18	0.0142	0.0265	Under-expressed
CAMK2G	-0.18	0.0147	0.0274	Under-expressed
ABCB7	-0.18	0.0171	0.0312	Under-expressed
C12ORF29	-0.18	0.0179	0.0325	Under-expressed
SIAH1	-0.18	0.0191	0.0345	Under-expressed
ZNF324	-0.18	0.0238	0.0418	Under-expressed
BCAP29	-0.18	0.0239	0.0421	Under-expressed
NUP42	-0.18	0.0262	0.0455	Under-expressed
MKRN2	-0.18	0.0267	0.0464	Under-expressed
TSC2	-0.18	0.0286	0.0493	Under-expressed
RAB7A	-0.19	1.04E-04	3.64E-04	Under-expressed
THRAP3	-0.19	1.13E-03	2.89E-03	Under-expressed
UBA5	-0.19	1.97E-03	4.69E-03	Under-expressed
PSEN1	-0.19	5.30E-03	0.0112	Under-expressed
ZBTB3	-0.19	6.79E-03	0.0139	Under-expressed
ZNF24	-0.19	6.95E-03	0.0142	Under-expressed

CTCF	-0.19	7.07E-03	0.0144	Under-expressed
CSDE1	-0.19	0.0103	0.0201	Under-expressed
FGFR1OP2	-0.19	0.0108	0.0208	Under-expressed
C7ORF25	-0.19	0.012	0.0228	Under-expressed
RPTOR	-0.19	0.0126	0.024	Under-expressed
EAPP	-0.19	0.0127	0.024	Under-expressed
INTS7	-0.19	0.0131	0.0247	Under-expressed
UBA3	-0.19	0.014	0.0262	Under-expressed
BZW1	-0.19	0.0156	0.0287	Under-expressed
SAP30L	-0.19	0.0164	0.03	Under-expressed
EI24	-0.19	0.0164	0.0302	Under-expressed
RAB22A	-0.19	0.0175	0.0319	Under-expressed
SLC35B3	-0.19	0.0182	0.0329	Under-expressed
RMC1	-0.19	0.0199	0.0357	Under-expressed
TBRG1	-0.19	0.0207	0.0369	Under-expressed
SHOC2	-0.19	0.0208	0.0372	Under-expressed
ZFYVE16	-0.19	0.0258	0.0449	Under-expressed
HS2ST1	-0.19	0.0276	0.0477	Under-expressed
LYRM7	-0.19	0.0278	0.0481	Under-expressed
ZDHHC5	-0.2	6.96E-04	1.90E-03	Under-expressed
RAB5B	-0.2	2.71E-03	6.24E-03	Under-expressed
TM9SF1	-0.2	2.78E-03	6.38E-03	Under-expressed
C3ORF38	-0.2	3.42E-03	7.63E-03	Under-expressed
NFX1	-0.2	4.34E-03	9.41E-03	Under-expressed
KLHL12	-0.2	4.88E-03	0.0104	Under-expressed
MMADHC	-0.2	5.47E-03	0.0115	Under-expressed
SMAD4	-0.2	7.31E-03	0.0148	Under-expressed
RO60	-0.2	7.78E-03	0.0157	Under-expressed
EZH1	-0.2	8.05E-03	0.0162	Under-expressed
WWP2	-0.2	9.57E-03	0.0188	Under-expressed
DPP9	-0.2	0.0141	0.0264	Under-expressed
USP24	-0.2	0.0146	0.0271	Under-expressed
KDM5A	-0.2	0.0148	0.0274	Under-expressed
PPP1CB	-0.2	0.0148	0.0275	Under-expressed
MAPKAPK2	-0.2	0.0169	0.0309	Under-expressed
RAB10	-0.2	0.0247	0.0433	Under-expressed
SPG21	-0.21	6.17E-04	1.71E-03	Under-expressed
BAG5	-0.21	1.74E-03	4.21E-03	Under-expressed
NMT1	-0.21	1.84E-03	4.42E-03	Under-expressed
TMEM167B	-0.21	2.25E-03	5.30E-03	Under-expressed
PPP1R10	-0.21	2.86E-03	6.53E-03	Under-expressed
WDR5B	-0.21	2.87E-03	6.56E-03	Under-expressed
ZNF79	-0.21	3.17E-03	7.15E-03	Under-expressed
PSMD12	-0.21	4.10E-03	8.94E-03	Under-expressed
WBP4	-0.21	4.29E-03	9.30E-03	Under-expressed
PUM2	-0.21	4.67E-03	0.01	Under-expressed
SETD1B	-0.21	4.78E-03	0.0102	Under-expressed
CDC27	-0.21	4.82E-03	0.0103	Under-expressed
RNF40	-0.21	6.15E-03	0.0128	Under-expressed
CYFIP1	-0.21	6.37E-03	0.0131	Under-expressed
FAM20B	-0.21	7.17E-03	0.0146	Under-expressed
KDSR	-0.21	7.19E-03	0.0146	Under-expressed
TNPO1	-0.21	8.65E-03	0.0172	Under-expressed
SYAP1	-0.21	0.0109	0.021	Under-expressed
ALG2	-0.21	0.0111	0.0214	Under-expressed
AMBRA1	-0.21	0.0112	0.0215	Under-expressed
PPP1R2	-0.21	0.0118	0.0225	Under-expressed

ATP6V1B2	-0.21	0.0135	0.0253	Under-expressed
CPSF7	-0.21	0.0155	0.0287	Under-expressed
YIPF5	-0.21	0.0161	0.0296	Under-expressed
RAB3GAP2	-0.21	0.0186	0.0336	Under-expressed
PTGR2	-0.21	0.0196	0.0352	Under-expressed
PEX11B	-0.21	0.0204	0.0366	Under-expressed
ALKBH8	-0.21	0.0215	0.0382	Under-expressed
PIAS1	-0.21	0.0258	0.0449	Under-expressed
EHD4	-0.21	0.0268	0.0465	Under-expressed
SERTAD2	-0.21	0.0276	0.0478	Under-expressed
VPS8	-0.22	7.21E-04	1.96E-03	Under-expressed
RTF1	-0.22	1.14E-03	2.92E-03	Under-expressed
METTL15	-0.22	1.68E-03	4.09E-03	Under-expressed
HDHD2	-0.22	1.79E-03	4.32E-03	Under-expressed
PPIG	-0.22	1.85E-03	4.45E-03	Under-expressed
CPSF2	-0.22	2.86E-03	6.53E-03	Under-expressed
TMED2	-0.22	3.11E-03	7.04E-03	Under-expressed
MLX	-0.22	3.22E-03	7.25E-03	Under-expressed
SAP130	-0.22	4.21E-03	9.14E-03	Under-expressed
CFAP20	-0.22	6.05E-03	0.0126	Under-expressed
SLC9A8	-0.22	6.07E-03	0.0126	Under-expressed
PAPOLA	-0.22	6.19E-03	0.0128	Under-expressed
LUC7L2	-0.22	6.21E-03	0.0129	Under-expressed
GIGYF2	-0.22	7.20E-03	0.0146	Under-expressed
KIAA1191	-0.22	9.44E-03	0.0186	Under-expressed
PSMD10	-0.22	0.0102	0.0199	Under-expressed
SMIM13	-0.22	0.0105	0.0203	Under-expressed
CHMP5	-0.22	0.0113	0.0218	Under-expressed
GMFB	-0.22	0.0118	0.0226	Under-expressed
REEP3	-0.22	0.0122	0.0232	Under-expressed
PGBD2	-0.22	0.0141	0.0264	Under-expressed
CBLL1	-0.22	0.0154	0.0285	Under-expressed
VIPAS39	-0.22	0.0155	0.0287	Under-expressed
BCKDK	-0.22	0.0161	0.0297	Under-expressed
USP34	-0.22	0.0164	0.0301	Under-expressed
PNPT1	-0.22	0.0177	0.0322	Under-expressed
SPCS3	-0.22	0.0204	0.0365	Under-expressed
TNKS1BP1	-0.22	0.0212	0.0378	Under-expressed
BPGM	-0.22	0.0218	0.0386	Under-expressed
PRRC2C	-0.22	0.0244	0.0427	Under-expressed
CAMK2D	-0.22	0.0251	0.0439	Under-expressed
GOSR1	-0.23	9.19E-05	3.26E-04	Under-expressed
C6ORF89	-0.23	4.11E-04	1.20E-03	Under-expressed
SELENOT	-0.23	5.74E-04	1.60E-03	Under-expressed
RAB14	-0.23	7.88E-04	2.12E-03	Under-expressed
GANAB	-0.23	1.70E-03	4.13E-03	Under-expressed
GNS	-0.23	2.05E-03	4.87E-03	Under-expressed
SNAP23	-0.23	2.20E-03	5.19E-03	Under-expressed
TYW1	-0.23	2.96E-03	6.73E-03	Under-expressed
UTP15	-0.23	3.34E-03	7.48E-03	Under-expressed
SCFD2	-0.23	3.41E-03	7.62E-03	Under-expressed
TCHP	-0.23	3.65E-03	8.06E-03	Under-expressed
AP2A1	-0.23	3.86E-03	8.48E-03	Under-expressed
CDK12	-0.23	4.16E-03	9.04E-03	Under-expressed
HAUS2	-0.23	4.38E-03	9.48E-03	Under-expressed
SPECC1L	-0.23	4.69E-03	0.0101	Under-expressed
ZNF646	-0.23	5.47E-03	0.0115	Under-expressed

DNAJA2	-0.23	6.25E-03	0.0129	Under-expressed
GATAD2B	-0.23	8.54E-03	0.017	Under-expressed
TRMT61B	-0.23	8.75E-03	0.0174	Under-expressed
ARL2BP	-0.23	0.0114	0.0219	Under-expressed
JKAMP	-0.23	0.013	0.0246	Under-expressed
CTIF	-0.23	0.0136	0.0256	Under-expressed
TRIM33	-0.23	0.0157	0.029	Under-expressed
SELENON	-0.23	0.0168	0.0308	Under-expressed
FLCN	-0.23	0.017	0.031	Under-expressed
ATF1	-0.23	0.0181	0.0328	Under-expressed
ZZEF1	-0.23	0.0192	0.0345	Under-expressed
INVS	-0.23	0.0206	0.0369	Under-expressed
FLYWCH1	-0.23	0.0239	0.0421	Under-expressed
CMIP	-0.23	0.0246	0.0431	Under-expressed
CCNDBP1	-0.23	0.0251	0.0439	Under-expressed
MFSD4B	-0.23	0.0259	0.0451	Under-expressed
PEX14	-0.23	0.0264	0.0459	Under-expressed
RIPK1	-0.24	3.97E-05	1.56E-04	Under-expressed
SETD3	-0.24	5.89E-04	1.64E-03	Under-expressed
RAB8A	-0.24	7.50E-04	2.03E-03	Under-expressed
ABRAXAS2	-0.24	8.01E-04	2.15E-03	Under-expressed
DELE1	-0.24	8.75E-04	2.32E-03	Under-expressed
ASB8	-0.24	8.87E-04	2.35E-03	Under-expressed
VPS26B	-0.24	9.62E-04	2.52E-03	Under-expressed
MUL1	-0.24	1.11E-03	2.83E-03	Under-expressed
PPP2R5C	-0.24	1.63E-03	3.97E-03	Under-expressed
ELOA	-0.24	1.76E-03	4.26E-03	Under-expressed
FOPNL	-0.24	1.85E-03	4.44E-03	Under-expressed
ASB7	-0.24	2.22E-03	5.24E-03	Under-expressed
MEF2D	-0.24	3.51E-03	7.80E-03	Under-expressed
ZNF200	-0.24	3.56E-03	7.88E-03	Under-expressed
ARCN1	-0.24	3.58E-03	7.92E-03	Under-expressed
FAM193A	-0.24	3.83E-03	8.41E-03	Under-expressed
TENT4A	-0.24	4.13E-03	8.99E-03	Under-expressed
ZNF786	-0.24	4.49E-03	9.68E-03	Under-expressed
NSL1	-0.24	5.20E-03	0.011	Under-expressed
CLN5	-0.24	5.79E-03	0.0121	Under-expressed
BAZ1B	-0.24	5.88E-03	0.0123	Under-expressed
PARP16	-0.24	6.90E-03	0.0141	Under-expressed
SEC16A	-0.24	7.93E-03	0.0159	Under-expressed
WDR36	-0.24	8.20E-03	0.0164	Under-expressed
PPP1R13B	-0.24	8.34E-03	0.0167	Under-expressed
RAB28	-0.24	9.35E-03	0.0184	Under-expressed
TMEM104	-0.24	0.0105	0.0204	Under-expressed
EGLN1	-0.24	0.0108	0.0208	Under-expressed
KMT5B	-0.24	0.0133	0.025	Under-expressed
ABI1	-0.24	0.0152	0.0281	Under-expressed
GID4	-0.24	0.0213	0.0379	Under-expressed
FBXW11	-0.24	0.022	0.0391	Under-expressed
ZNF182	-0.24	0.0241	0.0423	Under-expressed
ZNF267	-0.24	0.0248	0.0435	Under-expressed
SUGP2	-0.24	0.0251	0.0438	Under-expressed
USP7	-0.25	8.87E-05	3.16E-04	Under-expressed
SECISBP2	-0.25	2.54E-04	7.90E-04	Under-expressed
ATMIN	-0.25	3.74E-04	1.11E-03	Under-expressed
CAB39	-0.25	3.88E-04	1.14E-03	Under-expressed
CWC25	-0.25	4.13E-04	1.21E-03	Under-expressed

THAP12	-0.25	4.79E-04	1.37E-03	Under-expressed
CUL1	-0.25	6.83E-04	1.87E-03	Under-expressed
RNF20	-0.25	9.89E-04	2.58E-03	Under-expressed
SCFD1	-0.25	9.90E-04	2.58E-03	Under-expressed
CMTR1	-0.25	1.01E-03	2.62E-03	Under-expressed
NCBP1	-0.25	1.09E-03	2.81E-03	Under-expressed
TMEM131	-0.25	1.21E-03	3.06E-03	Under-expressed
SF3A1	-0.25	1.37E-03	3.42E-03	Under-expressed
UBN1	-0.25	1.46E-03	3.61E-03	Under-expressed
BLOC1S6	-0.25	1.59E-03	3.90E-03	Under-expressed
ZSCAN21	-0.25	1.68E-03	4.09E-03	Under-expressed
MICU1	-0.25	1.81E-03	4.35E-03	Under-expressed
ARPP19	-0.25	2.25E-03	5.30E-03	Under-expressed
DCAF12	-0.25	2.59E-03	6.00E-03	Under-expressed
USPL1	-0.25	2.88E-03	6.57E-03	Under-expressed
ANGEL2	-0.25	3.17E-03	7.15E-03	Under-expressed
MON1B	-0.25	3.32E-03	7.44E-03	Under-expressed
DNAJC11	-0.25	3.39E-03	7.58E-03	Under-expressed
MORF4L2	-0.25	3.44E-03	7.67E-03	Under-expressed
HERC4	-0.25	3.51E-03	7.80E-03	Under-expressed
STYX	-0.25	3.63E-03	8.03E-03	Under-expressed
SLC30A5	-0.25	3.71E-03	8.17E-03	Under-expressed
CEP120	-0.25	4.20E-03	9.12E-03	Under-expressed
CAPN7	-0.25	4.25E-03	9.21E-03	Under-expressed
NDFIP1	-0.25	4.67E-03	0.01	Under-expressed
FAM219A	-0.25	4.84E-03	0.0103	Under-expressed
OTUD7B	-0.25	5.02E-03	0.0107	Under-expressed
SF3B1	-0.25	6.18E-03	0.0128	Under-expressed
CNDP2	-0.25	6.22E-03	0.0129	Under-expressed
USP14	-0.25	6.66E-03	0.0137	Under-expressed
SIN3A	-0.25	7.60E-03	0.0154	Under-expressed
FAM111A	-0.25	8.20E-03	0.0164	Under-expressed
FBXO9	-0.25	8.48E-03	0.0169	Under-expressed
GRB10	-0.25	8.52E-03	0.017	Under-expressed
UBA6	-0.25	9.53E-03	0.0187	Under-expressed
ACAD10	-0.25	0.0108	0.0209	Under-expressed
HIRA	-0.25	0.0109	0.0211	Under-expressed
ZNF326	-0.25	0.0109	0.0211	Under-expressed
SMG8	-0.25	0.0122	0.0232	Under-expressed
ZNF655	-0.25	0.0124	0.0236	Under-expressed
TTC7A	-0.25	0.013	0.0246	Under-expressed
CLPB	-0.25	0.0142	0.0264	Under-expressed
FOXJ3	-0.25	0.0143	0.0267	Under-expressed
CEP57	-0.25	0.0143	0.0268	Under-expressed
APPL2	-0.25	0.0154	0.0284	Under-expressed
QTRT2	-0.25	0.017	0.031	Under-expressed
CNOT7	-0.25	0.0184	0.0333	Under-expressed
PLA2G12A	-0.25	0.021	0.0374	Under-expressed
KIF1BP	-0.25	0.0222	0.0393	Under-expressed
MBTD1	-0.25	0.0283	0.0489	Under-expressed
UBE4A	-0.26	1.05E-04	3.65E-04	Under-expressed
ZW10	-0.26	2.09E-04	6.67E-04	Under-expressed
MRFAP1L1	-0.26	2.68E-04	8.27E-04	Under-expressed
GRIPAP1	-0.26	3.00E-04	9.13E-04	Under-expressed
UBAP1	-0.26	4.21E-04	1.23E-03	Under-expressed
OCIAD1	-0.26	6.91E-04	1.89E-03	Under-expressed
GHITM	-0.26	7.50E-04	2.03E-03	Under-expressed

MFSD11	-0.26	7.86E-04	2.12E-03	Under-expressed
UBE3A	-0.26	9.31E-04	2.45E-03	Under-expressed
KPNA6	-0.26	1.07E-03	2.75E-03	Under-expressed
TRAPPC13	-0.26	1.41E-03	3.50E-03	Under-expressed
COQ10B	-0.26	1.48E-03	3.66E-03	Under-expressed
ARHGEF7	-0.26	1.68E-03	4.09E-03	Under-expressed
MSL2	-0.26	1.70E-03	4.13E-03	Under-expressed
ZNF639	-0.26	1.74E-03	4.22E-03	Under-expressed
PDE6D	-0.26	1.82E-03	4.38E-03	Under-expressed
DNAJC24	-0.26	2.32E-03	5.43E-03	Under-expressed
TLK1	-0.26	2.84E-03	6.51E-03	Under-expressed
MFSD8	-0.26	2.92E-03	6.66E-03	Under-expressed
NIPBL	-0.26	2.96E-03	6.72E-03	Under-expressed
SEC63	-0.26	2.97E-03	6.74E-03	Under-expressed
HMG20A	-0.26	3.63E-03	8.01E-03	Under-expressed
POMGNT2	-0.26	3.71E-03	8.17E-03	Under-expressed
BACE1	-0.26	4.37E-03	9.45E-03	Under-expressed
ATG2A	-0.26	5.07E-03	0.0108	Under-expressed
CLPTM1	-0.26	5.15E-03	0.0109	Under-expressed
ENOX2	-0.26	5.25E-03	0.0111	Under-expressed
FAM172A	-0.26	5.57E-03	0.0117	Under-expressed
MBNL1	-0.26	6.01E-03	0.0125	Under-expressed
CUL4A	-0.26	6.15E-03	0.0128	Under-expressed
DARS2	-0.26	6.80E-03	0.0139	Under-expressed
ZC3H11A	-0.26	7.00E-03	0.0143	Under-expressed
SNN	-0.26	7.37E-03	0.0149	Under-expressed
ODR4	-0.26	7.57E-03	0.0153	Under-expressed
NIT1	-0.26	7.76E-03	0.0156	Under-expressed
REV1	-0.26	8.51E-03	0.017	Under-expressed
SCAI	-0.26	9.08E-03	0.018	Under-expressed
GFPT1	-0.26	9.57E-03	0.0188	Under-expressed
GDPGP1	-0.26	0.011	0.0212	Under-expressed
HIRIP3	-0.26	0.0112	0.0216	Under-expressed
BCL2L2	-0.26	0.0123	0.0233	Under-expressed
MTMR3	-0.26	0.0126	0.0239	Under-expressed
BTBD7	-0.26	0.0128	0.0243	Under-expressed
UBN2	-0.26	0.0128	0.0243	Under-expressed
TMEM128	-0.26	0.0132	0.0248	Under-expressed
ORC3	-0.26	0.014	0.0262	Under-expressed
NUDT16	-0.26	0.0147	0.0274	Under-expressed
GRK5	-0.26	0.0149	0.0277	Under-expressed
NDEL1	-0.26	0.0155	0.0286	Under-expressed
SDHB	-0.26	0.0156	0.0287	Under-expressed
NAB1	-0.26	0.0157	0.029	Under-expressed
MTRF1	-0.26	0.0175	0.0319	Under-expressed
KDM1B	-0.26	0.0193	0.0348	Under-expressed
OCRL	-0.26	0.0194	0.035	Under-expressed
AHDC1	-0.26	0.0221	0.0391	Under-expressed
TTC5	-0.26	0.0268	0.0465	Under-expressed
ITPKB	-0.26	0.0274	0.0474	Under-expressed
DIP2A	-0.26	0.0276	0.0477	Under-expressed
TGFBR1	-0.26	0.0279	0.0482	Under-expressed
LSG1	-0.27	2.04E-06	1.18E-05	Under-expressed
FAM120A	-0.27	1.67E-05	7.32E-05	Under-expressed
CLTC	-0.27	1.85E-05	7.99E-05	Under-expressed
UBR4	-0.27	1.12E-04	3.87E-04	Under-expressed
PDHX	-0.27	2.26E-04	7.14E-04	Under-expressed

CGGBP1	-0.27	2.77E-04	8.53E-04	Under-expressed
TYK2	-0.27	3.23E-04	9.75E-04	Under-expressed
CYTH1	-0.27	7.69E-04	2.07E-03	Under-expressed
DCAKD	-0.27	9.27E-04	2.44E-03	Under-expressed
KDM4B	-0.27	1.07E-03	2.75E-03	Under-expressed
GNA13	-0.27	1.08E-03	2.77E-03	Under-expressed
RAB1A	-0.27	1.14E-03	2.92E-03	Under-expressed
ARIH1	-0.27	1.29E-03	3.23E-03	Under-expressed
HBS1L	-0.27	1.48E-03	3.65E-03	Under-expressed
ATPAF1	-0.27	1.50E-03	3.70E-03	Under-expressed
TOLLIP	-0.27	1.65E-03	4.02E-03	Under-expressed
TMX1	-0.27	1.83E-03	4.39E-03	Under-expressed
LUZP6	-0.27	2.20E-03	5.20E-03	Under-expressed
DNAJC14	-0.27	2.26E-03	5.30E-03	Under-expressed
DCAF6	-0.27	2.54E-03	5.88E-03	Under-expressed
LINC01278	-0.27	2.65E-03	6.12E-03	Under-expressed
DIAPH1	-0.27	2.73E-03	6.28E-03	Under-expressed
CD164	-0.27	2.90E-03	6.61E-03	Under-expressed
PRKAA1	-0.27	3.05E-03	6.90E-03	Under-expressed
SDAD1	-0.27	3.44E-03	7.66E-03	Under-expressed
FBXW7	-0.27	3.59E-03	7.95E-03	Under-expressed
RAP1A	-0.27	3.96E-03	8.67E-03	Under-expressed
TMEM170A	-0.27	4.01E-03	8.75E-03	Under-expressed
TIMM21	-0.27	4.42E-03	9.55E-03	Under-expressed
ZNF562	-0.27	4.69E-03	0.0101	Under-expressed
PTPN9	-0.27	5.29E-03	0.0112	Under-expressed
PDS5A	-0.27	5.89E-03	0.0123	Under-expressed
NAA35	-0.27	5.95E-03	0.0124	Under-expressed
TMEM209	-0.27	6.50E-03	0.0134	Under-expressed
ASF1A	-0.27	6.95E-03	0.0142	Under-expressed
ACBD3	-0.27	7.02E-03	0.0143	Under-expressed
CHD2	-0.27	7.25E-03	0.0147	Under-expressed
WDFY1	-0.27	7.48E-03	0.0151	Under-expressed
LUZP1	-0.27	8.18E-03	0.0164	Under-expressed
SNX27	-0.27	9.04E-03	0.0179	Under-expressed
ACER3	-0.27	9.56E-03	0.0188	Under-expressed
CHD1	-0.27	9.82E-03	0.0192	Under-expressed
RPRD2	-0.27	0.0105	0.0204	Under-expressed
RPS6KC1	-0.27	0.0108	0.0209	Under-expressed
RBM7	-0.27	0.015	0.0278	Under-expressed
FOXO3	-0.27	0.0155	0.0286	Under-expressed
FAM3C	-0.27	0.0163	0.03	Under-expressed
GPCPD1	-0.27	0.0165	0.0302	Under-expressed
CSRNP1	-0.27	0.0173	0.0315	Under-expressed
SLC46A1	-0.27	0.0173	0.0315	Under-expressed
KLHL9	-0.27	0.0177	0.0322	Under-expressed
TMEM129	-0.27	0.0185	0.0334	Under-expressed
SHPK	-0.27	0.0186	0.0336	Under-expressed
NEDD1	-0.27	0.0222	0.0393	Under-expressed
ARHGAP12	-0.27	0.023	0.0406	Under-expressed
ZNF189	-0.27	0.0239	0.0421	Under-expressed
C2CD3	-0.27	0.0246	0.0432	Under-expressed
POLR1A	-0.27	0.0248	0.0433	Under-expressed
PPFIBP2	-0.27	0.0257	0.0447	Under-expressed
SSBP3	-0.27	0.0262	0.0455	Under-expressed
AFAP1L1	-0.27	0.0269	0.0467	Under-expressed
GNAI2	-0.28	8.65E-05	3.09E-04	Under-expressed

USP15	-0.28	1.55E-04	5.14E-04	Under-expressed
RNF141	-0.28	2.22E-04	7.02E-04	Under-expressed
ADAR	-0.28	3.45E-04	1.03E-03	Under-expressed
PCGF5	-0.28	4.10E-04	1.20E-03	Under-expressed
MARF1	-0.28	5.45E-04	1.53E-03	Under-expressed
ERI2	-0.28	6.82E-04	1.87E-03	Under-expressed
DUSP3	-0.28	8.74E-04	2.32E-03	Under-expressed
PHF12	-0.28	1.10E-03	2.81E-03	Under-expressed
MED1	-0.28	1.12E-03	2.85E-03	Under-expressed
CWF19L2	-0.28	1.18E-03	3.00E-03	Under-expressed
MBTPS1	-0.28	1.29E-03	3.25E-03	Under-expressed
C5ORF22	-0.28	1.31E-03	3.29E-03	Under-expressed
CDC5L	-0.28	1.37E-03	3.41E-03	Under-expressed
DIS3	-0.28	1.52E-03	3.75E-03	Under-expressed
CDKN1B	-0.28	1.93E-03	4.61E-03	Under-expressed
GOLGA2	-0.28	1.95E-03	4.65E-03	Under-expressed
CLDN12	-0.28	2.14E-03	5.07E-03	Under-expressed
STRIP1	-0.28	2.31E-03	5.40E-03	Under-expressed
FCHSD2	-0.28	2.36E-03	5.51E-03	Under-expressed
ANKRD13C	-0.28	2.60E-03	6.02E-03	Under-expressed
MACO1	-0.28	2.84E-03	6.51E-03	Under-expressed
EIF4G2	-0.28	3.36E-03	7.52E-03	Under-expressed
LRRC37A16P	-0.28	3.45E-03	7.70E-03	Under-expressed
ZFAND3	-0.28	3.63E-03	8.02E-03	Under-expressed
PRRC1	-0.28	3.69E-03	8.12E-03	Under-expressed
RNF213	-0.28	3.88E-03	8.50E-03	Under-expressed
HARBI1	-0.28	3.92E-03	8.58E-03	Under-expressed
NBR1	-0.28	4.08E-03	8.89E-03	Under-expressed
PPP2R5A	-0.28	5.22E-03	0.011	Under-expressed
RABGAP1	-0.28	5.26E-03	0.0111	Under-expressed
PIGB	-0.28	5.66E-03	0.0118	Under-expressed
SENP6	-0.28	7.91E-03	0.0159	Under-expressed
PANK4	-0.28	8.42E-03	0.0168	Under-expressed
BNIP3L	-0.28	9.18E-03	0.0181	Under-expressed
SGPP1	-0.28	9.23E-03	0.0182	Under-expressed
CHP1	-0.28	9.50E-03	0.0187	Under-expressed
ZNF546	-0.28	0.0101	0.0197	Under-expressed
ZNF181	-0.28	0.0109	0.0211	Under-expressed
UNKL	-0.28	0.0113	0.0217	Under-expressed
ATF6B	-0.28	0.0123	0.0235	Under-expressed
PDIA4	-0.28	0.0127	0.024	Under-expressed
IKZF5	-0.28	0.0137	0.0256	Under-expressed
C16ORF70	-0.28	0.0157	0.0289	Under-expressed
SEPTIN11	-0.28	0.0166	0.0304	Under-expressed
ABHD4	-0.28	0.0168	0.0307	Under-expressed
PMS2CL	-0.28	0.0173	0.0314	Under-expressed
VPS13B	-0.28	0.0181	0.0329	Under-expressed
KIAA0513	-0.28	0.0191	0.0345	Under-expressed
DOP1A	-0.28	0.0197	0.0354	Under-expressed
NCOA6	-0.28	0.0199	0.0357	Under-expressed
ZNF184	-0.28	0.0229	0.0405	Under-expressed
IMPAD1	-0.28	0.0268	0.0466	Under-expressed
ZNF785	-0.28	0.0279	0.0482	Under-expressed
CPTP	-0.28	0.0284	0.049	Under-expressed
GORASP1	-0.29	2.72E-08	2.93E-07	Under-expressed
DDX5	-0.29	3.24E-05	1.31E-04	Under-expressed
DNAJC16	-0.29	6.58E-05	2.42E-04	Under-expressed

UBE3B	-0.29	1.06E-04	3.70E-04	Under-expressed
BSDC1	-0.29	1.50E-04	5.00E-04	Under-expressed
SEC22C	-0.29	2.23E-04	7.06E-04	Under-expressed
PURA	-0.29	2.80E-04	8.59E-04	Under-expressed
MYNN	-0.29	3.02E-04	9.21E-04	Under-expressed
OSTF1	-0.29	3.81E-04	1.13E-03	Under-expressed
PGGT1B	-0.29	3.85E-04	1.14E-03	Under-expressed
RBM18	-0.29	4.77E-04	1.37E-03	Under-expressed
INO80	-0.29	5.39E-04	1.52E-03	Under-expressed
MTMR12	-0.29	5.78E-04	1.61E-03	Under-expressed
RNF4	-0.29	9.01E-04	2.38E-03	Under-expressed
COL4A3BP	-0.29	1.04E-03	2.69E-03	Under-expressed
PIGO	-0.29	1.35E-03	3.38E-03	Under-expressed
SEC31A	-0.29	1.52E-03	3.74E-03	Under-expressed
DHX29	-0.29	1.63E-03	3.97E-03	Under-expressed
ZBTB7A	-0.29	1.88E-03	4.51E-03	Under-expressed
ANKFY1	-0.29	2.06E-03	4.90E-03	Under-expressed
ERGIC1	-0.29	2.60E-03	6.01E-03	Under-expressed
CHMP1B	-0.29	2.63E-03	6.07E-03	Under-expressed
RPRD1B	-0.29	2.66E-03	6.14E-03	Under-expressed
KRR1	-0.29	2.86E-03	6.54E-03	Under-expressed
G3BP2	-0.29	2.88E-03	6.58E-03	Under-expressed
GRAMD2B	-0.29	3.28E-03	7.37E-03	Under-expressed
CNOT6L	-0.29	3.29E-03	7.37E-03	Under-expressed
RCHY1	-0.29	3.63E-03	8.03E-03	Under-expressed
SMC5	-0.29	3.89E-03	8.53E-03	Under-expressed
COQ6	-0.29	4.23E-03	9.19E-03	Under-expressed
TRIM32	-0.29	5.56E-03	0.0116	Under-expressed
CATSPER2P1	-0.29	5.77E-03	0.012	Under-expressed
CNEP1R1	-0.29	7.90E-03	0.0159	Under-expressed
SCAF8	-0.29	8.58E-03	0.0171	Under-expressed
CPNE3	-0.29	8.79E-03	0.0175	Under-expressed
BCAS3	-0.29	9.04E-03	0.0179	Under-expressed
USP46	-0.29	9.08E-03	0.018	Under-expressed
SP100	-0.29	9.41E-03	0.0185	Under-expressed
FASTKD5	-0.29	0.0108	0.0209	Under-expressed
FAM122B	-0.29	0.0146	0.0272	Under-expressed
SBF1	-0.29	0.0148	0.0275	Under-expressed
ZDHHC17	-0.29	0.0175	0.0319	Under-expressed
FAM117A	-0.29	0.0181	0.0328	Under-expressed
DLL4	-0.29	0.0183	0.0331	Under-expressed
ZNF180	-0.29	0.0211	0.0376	Under-expressed
API5	-0.3	4.29E-07	3.09E-06	Under-expressed
MRPL49	-0.3	5.08E-06	2.60E-05	Under-expressed
USP19	-0.3	1.12E-05	5.18E-05	Under-expressed
THAP6	-0.3	2.61E-05	1.09E-04	Under-expressed
SLTM	-0.3	5.94E-05	2.22E-04	Under-expressed
FAM160A2	-0.3	6.80E-05	2.49E-04	Under-expressed
RC3H1	-0.3	1.70E-04	5.57E-04	Under-expressed
CRBN	-0.3	1.95E-04	6.30E-04	Under-expressed
RIOK3	-0.3	2.67E-04	8.24E-04	Under-expressed
WDR20	-0.3	2.85E-04	8.74E-04	Under-expressed
CEPT1	-0.3	3.93E-04	1.16E-03	Under-expressed
GPBP1L1	-0.3	4.27E-04	1.24E-03	Under-expressed
SYPL1	-0.3	4.68E-04	1.35E-03	Under-expressed
CUL2	-0.3	4.98E-04	1.42E-03	Under-expressed
SEPTIN7	-0.3	5.84E-04	1.62E-03	Under-expressed

APPL1	-0.3	6.68E-04	1.84E-03	Under-expressed
MICU2	-0.3	7.03E-04	1.92E-03	Under-expressed
TMED10	-0.3	9.52E-04	2.50E-03	Under-expressed
EID1	-0.3	1.16E-03	2.96E-03	Under-expressed
DTD2	-0.3	1.27E-03	3.19E-03	Under-expressed
PKD1	-0.3	1.39E-03	3.46E-03	Under-expressed
MAPKBP1	-0.3	1.89E-03	4.53E-03	Under-expressed
CYB5RL	-0.3	2.13E-03	5.04E-03	Under-expressed
PSMD5	-0.3	2.60E-03	6.02E-03	Under-expressed
LRRC57	-0.3	2.63E-03	6.08E-03	Under-expressed
HACD3	-0.3	3.16E-03	7.13E-03	Under-expressed
PNKD	-0.3	3.23E-03	7.27E-03	Under-expressed
FAM45A	-0.3	3.28E-03	7.36E-03	Under-expressed
GTF2E1	-0.3	3.32E-03	7.44E-03	Under-expressed
OXSR1	-0.3	3.55E-03	7.87E-03	Under-expressed
SLC35A1	-0.3	3.62E-03	8.00E-03	Under-expressed
AKTIP	-0.3	3.62E-03	8.01E-03	Under-expressed
STAM	-0.3	4.03E-03	8.80E-03	Under-expressed
TRAK1	-0.3	4.47E-03	9.66E-03	Under-expressed
AKT2	-0.3	4.77E-03	0.0102	Under-expressed
ICE1	-0.3	4.98E-03	0.0106	Under-expressed
GBA	-0.3	5.10E-03	0.0108	Under-expressed
MED21	-0.3	5.22E-03	0.011	Under-expressed
GXYLT1	-0.3	5.23E-03	0.011	Under-expressed
FBXO4	-0.3	5.31E-03	0.0112	Under-expressed
CLCN6	-0.3	5.51E-03	0.0116	Under-expressed
PSIP1	-0.3	7.41E-03	0.015	Under-expressed
PDIA5	-0.3	7.72E-03	0.0156	Under-expressed
KIAA0556	-0.3	9.24E-03	0.0182	Under-expressed
ATXN7L3B	-0.3	9.39E-03	0.0185	Under-expressed
SPOPL	-0.3	9.80E-03	0.0192	Under-expressed
DENND5A	-0.3	9.90E-03	0.0193	Under-expressed
MDM2	-0.3	9.90E-03	0.0193	Under-expressed
IFT88	-0.3	0.0111	0.0213	Under-expressed
ITGB1	-0.3	0.0132	0.0248	Under-expressed
HACL1	-0.3	0.0146	0.0272	Under-expressed
ZBTB33	-0.3	0.0156	0.0288	Under-expressed
TICAM1	-0.3	0.0163	0.03	Under-expressed
POLR3GL	-0.3	0.0166	0.0305	Under-expressed
CREB3L2	-0.3	0.0177	0.0322	Under-expressed
ECI2	-0.3	0.024	0.0421	Under-expressed
SLC17A5	-0.3	0.0249	0.0436	Under-expressed
TAF1B	-0.3	0.0282	0.0487	Under-expressed
STAT6	-0.3	0.029	0.0499	Under-expressed
STX12	-0.31	6.64E-06	3.29E-05	Under-expressed
PEX13	-0.31	1.54E-05	6.80E-05	Under-expressed
RAB21	-0.31	2.28E-05	9.63E-05	Under-expressed
WDR92	-0.31	2.43E-05	1.02E-04	Under-expressed
CMPK1	-0.31	3.85E-05	1.52E-04	Under-expressed
TXNDC11	-0.31	3.90E-05	1.54E-04	Under-expressed
ESYT1	-0.31	4.50E-05	1.74E-04	Under-expressed
TAX1BP1	-0.31	1.35E-04	4.56E-04	Under-expressed
FBXO7	-0.31	1.37E-04	4.63E-04	Under-expressed
ELK1	-0.31	1.70E-04	5.58E-04	Under-expressed
RAB6A	-0.31	2.07E-04	6.62E-04	Under-expressed
ZFYVE26	-0.31	2.89E-04	8.85E-04	Under-expressed
UBP1	-0.31	3.03E-04	9.22E-04	Under-expressed

ZMYM6	-0.31	3.09E-04	9.39E-04	Under-expressed
ING1	-0.31	3.72E-04	1.11E-03	Under-expressed
PRDM2	-0.31	4.32E-04	1.26E-03	Under-expressed
COPB2	-0.31	4.56E-04	1.32E-03	Under-expressed
FAM149B1	-0.31	4.70E-04	1.35E-03	Under-expressed
FBXO11	-0.31	5.04E-04	1.44E-03	Under-expressed
PHRF1	-0.31	5.83E-04	1.62E-03	Under-expressed
DNAJA3	-0.31	5.88E-04	1.63E-03	Under-expressed
RHOT1	-0.31	7.92E-04	2.13E-03	Under-expressed
USP42	-0.31	8.18E-04	2.19E-03	Under-expressed
STAT3	-0.31	8.63E-04	2.29E-03	Under-expressed
ZNF746	-0.31	9.31E-04	2.45E-03	Under-expressed
AKAP1	-0.31	1.04E-03	2.68E-03	Under-expressed
ARMC8	-0.31	1.06E-03	2.74E-03	Under-expressed
SOCS5	-0.31	1.52E-03	3.74E-03	Under-expressed
NAXD	-0.31	1.60E-03	3.91E-03	Under-expressed
ELF2	-0.31	2.28E-03	5.36E-03	Under-expressed
CEP44	-0.31	2.78E-03	6.38E-03	Under-expressed
ENOSF1	-0.31	3.14E-03	7.09E-03	Under-expressed
C11ORF71	-0.31	3.70E-03	8.14E-03	Under-expressed
TCF20	-0.31	3.83E-03	8.40E-03	Under-expressed
B3GLCT	-0.31	5.19E-03	0.011	Under-expressed
RFX5	-0.31	5.50E-03	0.0115	Under-expressed
PPP1R21	-0.31	5.75E-03	0.012	Under-expressed
MAP4K2	-0.31	6.60E-03	0.0136	Under-expressed
FGFR1OP	-0.31	6.75E-03	0.0139	Under-expressed
GSKIP	-0.31	7.43E-03	0.0151	Under-expressed
DBR1	-0.31	9.36E-03	0.0184	Under-expressed
NIN	-0.31	0.0112	0.0215	Under-expressed
URB2	-0.31	0.0131	0.0247	Under-expressed
PPP1R12A	-0.31	0.0136	0.0256	Under-expressed
FIGNL1	-0.31	0.0163	0.03	Under-expressed
SLF1	-0.31	0.0204	0.0365	Under-expressed
RGMB	-0.31	0.0206	0.0368	Under-expressed
SLC25A10	-0.31	0.0253	0.0442	Under-expressed
SLC37A4	-0.31	0.0276	0.0478	Under-expressed
C16ORF58	-0.32	2.98E-05	1.22E-04	Under-expressed
AGGF1	-0.32	4.13E-05	1.62E-04	Under-expressed
ADD1	-0.32	4.55E-05	1.76E-04	Under-expressed
TENT4B	-0.32	5.84E-05	2.19E-04	Under-expressed
PCF11	-0.32	9.11E-05	3.23E-04	Under-expressed
ADIPOR1	-0.32	9.91E-05	3.48E-04	Under-expressed
PICALM	-0.32	1.39E-04	4.66E-04	Under-expressed
PDSS2	-0.32	1.40E-04	4.71E-04	Under-expressed
MSH3	-0.32	1.54E-04	5.12E-04	Under-expressed
FKBP15	-0.32	2.16E-04	6.88E-04	Under-expressed
PDS5B	-0.32	2.28E-04	7.18E-04	Under-expressed
KDM2A	-0.32	2.29E-04	7.22E-04	Under-expressed
NRBF2	-0.32	2.33E-04	7.33E-04	Under-expressed
MOAP1	-0.32	2.76E-04	8.50E-04	Under-expressed
GPALPP1	-0.32	2.82E-04	8.65E-04	Under-expressed
WASL	-0.32	3.57E-04	1.07E-03	Under-expressed
BBS4	-0.32	3.97E-04	1.17E-03	Under-expressed
MTO1	-0.32	4.07E-04	1.19E-03	Under-expressed
EXOC2	-0.32	4.18E-04	1.22E-03	Under-expressed
ZFHX3	-0.32	4.66E-04	1.34E-03	Under-expressed
ETFA	-0.32	4.70E-04	1.35E-03	Under-expressed

COG8	-0.32	5.75E-04	1.61E-03	Under-expressed
SIDT2	-0.32	6.67E-04	1.83E-03	Under-expressed
KPNA3	-0.32	6.79E-04	1.86E-03	Under-expressed
UFM1	-0.32	9.66E-04	2.52E-03	Under-expressed
THAP1	-0.32	9.68E-04	2.53E-03	Under-expressed
MLH1	-0.32	1.10E-03	2.82E-03	Under-expressed
TASOR	-0.32	1.31E-03	3.29E-03	Under-expressed
XPR1	-0.32	1.42E-03	3.52E-03	Under-expressed
CERS2	-0.32	1.42E-03	3.53E-03	Under-expressed
PDCD10	-0.32	1.47E-03	3.64E-03	Under-expressed
NDUFAF1	-0.32	1.55E-03	3.81E-03	Under-expressed
CDADC1	-0.32	1.75E-03	4.24E-03	Under-expressed
MIER3	-0.32	1.88E-03	4.51E-03	Under-expressed
UQCRC2	-0.32	1.92E-03	4.59E-03	Under-expressed
ZNF12	-0.32	2.38E-03	5.55E-03	Under-expressed
ANO10	-0.32	2.46E-03	5.73E-03	Under-expressed
NDST1	-0.32	2.90E-03	6.60E-03	Under-expressed
ATR	-0.32	3.38E-03	7.57E-03	Under-expressed
AASDHPPT	-0.32	3.49E-03	7.77E-03	Under-expressed
ARFGAP3	-0.32	3.94E-03	8.64E-03	Under-expressed
CASP8	-0.32	3.98E-03	8.70E-03	Under-expressed
ISG20L2	-0.32	4.46E-03	9.62E-03	Under-expressed
RLIM	-0.32	4.52E-03	9.74E-03	Under-expressed
NUP160	-0.32	4.67E-03	0.01	Under-expressed
LOC220729	-0.32	5.40E-03	0.0114	Under-expressed
TUBGCP6	-0.32	6.16E-03	0.0128	Under-expressed
PNPLA4	-0.32	6.23E-03	0.0129	Under-expressed
ATF7	-0.32	6.26E-03	0.0129	Under-expressed
TACC2	-0.32	6.35E-03	0.0131	Under-expressed
PTPRF	-0.32	7.29E-03	0.0148	Under-expressed
SEC14L1	-0.32	8.61E-03	0.0171	Under-expressed
ARMC1	-0.32	8.79E-03	0.0175	Under-expressed
RTTN	-0.32	0.0125	0.0237	Under-expressed
DGLUCY	-0.32	0.013	0.0245	Under-expressed
MAP3K9	-0.32	0.0169	0.0308	Under-expressed
PXDC1	-0.32	0.0206	0.0368	Under-expressed
CALHM2	-0.32	0.0243	0.0427	Under-expressed
SPICE1	-0.32	0.0286	0.0493	Under-expressed
RABL3	-0.33	2.03E-07	1.64E-06	Under-expressed
RPP14	-0.33	2.67E-06	1.48E-05	Under-expressed
GOLGB1	-0.33	1.24E-05	5.68E-05	Under-expressed
HP1BP3	-0.33	3.38E-05	1.36E-04	Under-expressed
USP47	-0.33	3.89E-05	1.54E-04	Under-expressed
TOR1A	-0.33	4.07E-05	1.60E-04	Under-expressed
ILRUN	-0.33	4.27E-05	1.67E-04	Under-expressed
RBBP6	-0.33	4.80E-05	1.84E-04	Under-expressed
TTC31	-0.33	6.54E-05	2.41E-04	Under-expressed
BNIP2	-0.33	1.02E-04	3.58E-04	Under-expressed
RNF130	-0.33	1.15E-04	3.97E-04	Under-expressed
EIF4H	-0.33	1.19E-04	4.09E-04	Under-expressed
TRAF3IP1	-0.33	1.25E-04	4.26E-04	Under-expressed
ZBTB6	-0.33	1.80E-04	5.88E-04	Under-expressed
CMAS	-0.33	3.64E-04	1.08E-03	Under-expressed
RNF146	-0.33	3.99E-04	1.17E-03	Under-expressed
SMAP2	-0.33	5.39E-04	1.52E-03	Under-expressed
ALG13	-0.33	5.42E-04	1.53E-03	Under-expressed
RYBP	-0.33	5.74E-04	1.60E-03	Under-expressed

RFK	-0.33	8.24E-04	2.20E-03	Under-expressed
ARL6IP5	-0.33	8.60E-04	2.29E-03	Under-expressed
FAM126B	-0.33	9.67E-04	2.53E-03	Under-expressed
RSBN1	-0.33	1.04E-03	2.69E-03	Under-expressed
SFMBT1	-0.33	1.05E-03	2.71E-03	Under-expressed
WDR37	-0.33	1.15E-03	2.92E-03	Under-expressed
LEMD3	-0.33	1.46E-03	3.60E-03	Under-expressed
TCTA	-0.33	1.46E-03	3.61E-03	Under-expressed
LGALS8	-0.33	1.58E-03	3.87E-03	Under-expressed
PIP4K2C	-0.33	1.66E-03	4.04E-03	Under-expressed
YLPM1	-0.33	1.69E-03	4.11E-03	Under-expressed
PRICKLE3	-0.33	1.89E-03	4.52E-03	Under-expressed
DR1	-0.33	1.91E-03	4.58E-03	Under-expressed
CNIH1	-0.33	1.96E-03	4.67E-03	Under-expressed
CTNNB1	-0.33	2.45E-03	5.70E-03	Under-expressed
TRIM21	-0.33	2.89E-03	6.59E-03	Under-expressed
FCF1	-0.33	3.09E-03	7.00E-03	Under-expressed
AIFM1	-0.33	3.36E-03	7.53E-03	Under-expressed
ARID4B	-0.33	3.46E-03	7.71E-03	Under-expressed
CRKL	-0.33	3.66E-03	8.07E-03	Under-expressed
ELMO2	-0.33	3.74E-03	8.23E-03	Under-expressed
PTPN1	-0.33	3.97E-03	8.69E-03	Under-expressed
SMARCA5	-0.33	4.12E-03	8.98E-03	Under-expressed
AMMECR1	-0.33	4.78E-03	0.0102	Under-expressed
CCNG1	-0.33	4.91E-03	0.0105	Under-expressed
ZMYM2	-0.33	4.97E-03	0.0106	Under-expressed
MXI1	-0.33	5.48E-03	0.0115	Under-expressed
SPRYD4	-0.33	5.52E-03	0.0116	Under-expressed
DEXI	-0.33	6.45E-03	0.0133	Under-expressed
DDHD1	-0.33	6.51E-03	0.0134	Under-expressed
YPEL1	-0.33	6.53E-03	0.0134	Under-expressed
TRAM2	-0.33	6.87E-03	0.0141	Under-expressed
KIFAP3	-0.33	9.99E-03	0.0195	Under-expressed
RBM12B	-0.33	0.0126	0.0239	Under-expressed
SLC35E3	-0.33	0.0127	0.0241	Under-expressed
NUAK1	-0.33	0.0139	0.026	Under-expressed
IKZF4	-0.33	0.0146	0.0272	Under-expressed
BCLAF3	-0.33	0.0161	0.0296	Under-expressed
ZNF10	-0.33	0.0169	0.0309	Under-expressed
SESTD1	-0.33	0.0185	0.0334	Under-expressed
MCF2L	-0.33	0.0196	0.0352	Under-expressed
LIMA1	-0.33	0.0226	0.0399	Under-expressed
ZMIZ1	-0.33	0.0235	0.0414	Under-expressed
CEP19	-0.33	0.0281	0.0486	Under-expressed
CUL5	-0.34	4.51E-05	1.74E-04	Under-expressed
NUP98	-0.34	5.74E-05	2.15E-04	Under-expressed
TRIM4	-0.34	8.28E-05	2.97E-04	Under-expressed
LIN54	-0.34	1.10E-04	3.80E-04	Under-expressed
FBXO33	-0.34	1.21E-04	4.13E-04	Under-expressed
THUMPD1	-0.34	1.62E-04	5.34E-04	Under-expressed
TRMT10A	-0.34	1.84E-04	5.97E-04	Under-expressed
FAM185A	-0.34	1.92E-04	6.24E-04	Under-expressed
ZFR	-0.34	2.02E-04	6.48E-04	Under-expressed
ZNFX1	-0.34	2.72E-04	8.37E-04	Under-expressed
SCAF4	-0.34	2.89E-04	8.85E-04	Under-expressed
FAM177A1	-0.34	3.12E-04	9.48E-04	Under-expressed
ZZZ3	-0.34	3.36E-04	1.01E-03	Under-expressed

SLC30A9	-0.34	3.91E-04	1.15E-03	Under-expressed
NECAP1	-0.34	3.93E-04	1.16E-03	Under-expressed
RSU1	-0.34	3.95E-04	1.16E-03	Under-expressed
LOC646214	-0.34	5.09E-04	1.45E-03	Under-expressed
RBM27	-0.34	5.40E-04	1.52E-03	Under-expressed
CASP9	-0.34	5.86E-04	1.63E-03	Under-expressed
ZNF319	-0.34	9.37E-04	2.46E-03	Under-expressed
KTN1	-0.34	1.05E-03	2.70E-03	Under-expressed
UBQLN2	-0.34	1.15E-03	2.93E-03	Under-expressed
RELCH	-0.34	1.15E-03	2.94E-03	Under-expressed
SGPL1	-0.34	1.16E-03	2.96E-03	Under-expressed
CEP57L1	-0.34	1.79E-03	4.33E-03	Under-expressed
EIF3A	-0.34	2.12E-03	5.02E-03	Under-expressed
CASP7	-0.34	2.63E-03	6.08E-03	Under-expressed
ACOX3	-0.34	2.67E-03	6.15E-03	Under-expressed
CREBZF	-0.34	3.54E-03	7.85E-03	Under-expressed
DCTN6	-0.34	4.08E-03	8.89E-03	Under-expressed
GOLGA7	-0.34	4.14E-03	9.01E-03	Under-expressed
WASHC3	-0.34	4.19E-03	9.10E-03	Under-expressed
NARS2	-0.34	4.30E-03	9.33E-03	Under-expressed
TOPBP1	-0.34	4.51E-03	9.73E-03	Under-expressed
AHCTF1	-0.34	4.67E-03	0.01	Under-expressed
NADK	-0.34	5.71E-03	0.0119	Under-expressed
BBS2	-0.34	7.05E-03	0.0144	Under-expressed
RBFOX2	-0.34	7.26E-03	0.0147	Under-expressed
RFLNB	-0.34	8.13E-03	0.0163	Under-expressed
MED12	-0.34	8.65E-03	0.0172	Under-expressed
FOXRED2	-0.34	8.88E-03	0.0176	Under-expressed
ZNF45	-0.34	8.88E-03	0.0176	Under-expressed
TBC1D12	-0.34	9.17E-03	0.0181	Under-expressed
NCOR1	-0.34	9.29E-03	0.0183	Under-expressed
RAI14	-0.34	9.74E-03	0.0191	Under-expressed
SOWAHC	-0.34	0.0104	0.0203	Under-expressed
ZNF518A	-0.34	0.0112	0.0216	Under-expressed
SMURF2	-0.34	0.0116	0.0222	Under-expressed
TMEM245	-0.34	0.014	0.0262	Under-expressed
NR1D1	-0.34	0.0141	0.0264	Under-expressed
AFDN	-0.34	0.0143	0.0266	Under-expressed
PHF3	-0.34	0.016	0.0294	Under-expressed
PEX11A	-0.34	0.0163	0.03	Under-expressed
ZNF264	-0.34	0.0169	0.0308	Under-expressed
GIMAP4	-0.34	0.0241	0.0423	Under-expressed
MRPL19	-0.35	5.43E-08	5.28E-07	Under-expressed
TRAPPC6B	-0.35	6.79E-07	4.60E-06	Under-expressed
DCAF10	-0.35	2.82E-06	1.55E-05	Under-expressed
EIF5	-0.35	6.49E-06	3.22E-05	Under-expressed
PPP4R3A	-0.35	2.33E-05	9.80E-05	Under-expressed
RAF1	-0.35	3.29E-05	1.33E-04	Under-expressed
FAM76A	-0.35	6.98E-05	2.55E-04	Under-expressed
HINT3	-0.35	1.01E-04	3.54E-04	Under-expressed
ZC3H14	-0.35	1.52E-04	5.06E-04	Under-expressed
THAP5	-0.35	1.79E-04	5.85E-04	Under-expressed
AREL1	-0.35	2.23E-04	7.06E-04	Under-expressed
RBMXL1	-0.35	3.13E-04	9.51E-04	Under-expressed
ZNF747	-0.35	3.42E-04	1.03E-03	Under-expressed
PTCD2	-0.35	3.47E-04	1.04E-03	Under-expressed
PRR14L	-0.35	4.03E-04	1.18E-03	Under-expressed

CAAP1	-0.35	4.04E-04	1.18E-03	Under-expressed
FBXL17	-0.35	4.58E-04	1.32E-03	Under-expressed
DLD	-0.35	6.46E-04	1.78E-03	Under-expressed
ALMS1	-0.35	8.08E-04	2.17E-03	Under-expressed
POGLUT1	-0.35	8.20E-04	2.19E-03	Under-expressed
JMY	-0.35	8.81E-04	2.33E-03	Under-expressed
B4GALT1	-0.35	9.64E-04	2.52E-03	Under-expressed
BRMS1L	-0.35	9.77E-04	2.55E-03	Under-expressed
FGFRL1	-0.35	9.96E-04	2.59E-03	Under-expressed
IGIP	-0.35	1.20E-03	3.04E-03	Under-expressed
UBE4B	-0.35	1.55E-03	3.80E-03	Under-expressed
AKIRIN1	-0.35	1.55E-03	3.81E-03	Under-expressed
PAN2	-0.35	1.61E-03	3.93E-03	Under-expressed
KIAA1671	-0.35	2.39E-03	5.58E-03	Under-expressed
ANKRD42	-0.35	2.62E-03	6.05E-03	Under-expressed
ARSA	-0.35	3.97E-03	8.69E-03	Under-expressed
KIAA1217	-0.35	4.38E-03	9.48E-03	Under-expressed
ARMCX5	-0.35	4.85E-03	0.0104	Under-expressed
DUSP7	-0.35	4.98E-03	0.0106	Under-expressed
PLXNB2	-0.35	6.11E-03	0.0127	Under-expressed
PRKD3	-0.35	6.80E-03	0.0139	Under-expressed
LINC00174	-0.35	7.68E-03	0.0155	Under-expressed
ZNF516	-0.35	7.72E-03	0.0156	Under-expressed
DNAJC27	-0.35	7.90E-03	0.0159	Under-expressed
HYLS1	-0.35	8.22E-03	0.0165	Under-expressed
MAP3K14	-0.35	8.67E-03	0.0172	Under-expressed
EXD2	-0.35	9.44E-03	0.0186	Under-expressed
FN3K	-0.35	0.013	0.0245	Under-expressed
RHOU	-0.35	0.0134	0.0251	Under-expressed
CCDC14	-0.35	0.014	0.0262	Under-expressed
KDM6A	-0.35	0.0164	0.0301	Under-expressed
MRPL39	-0.35	0.0171	0.0311	Under-expressed
ZNF92	-0.35	0.0186	0.0336	Under-expressed
NEMP1	-0.35	0.0199	0.0357	Under-expressed
LTB4R	-0.35	0.0208	0.0371	Under-expressed
LACTB2	-0.35	0.0213	0.0378	Under-expressed
ADNP	-0.35	0.0262	0.0455	Under-expressed
MBD4	-0.36	8.44E-08	7.72E-07	Under-expressed
SETX	-0.36	1.54E-06	9.20E-06	Under-expressed
ZMPSTE24	-0.36	1.63E-06	9.68E-06	Under-expressed
GCC1	-0.36	2.18E-06	1.24E-05	Under-expressed
TMBIM6	-0.36	3.18E-06	1.72E-05	Under-expressed
EPS15	-0.36	5.67E-06	2.86E-05	Under-expressed
CLCC1	-0.36	7.99E-06	3.85E-05	Under-expressed
USP8	-0.36	8.25E-06	3.97E-05	Under-expressed
PLEKHM1	-0.36	8.91E-06	4.25E-05	Under-expressed
TBC1D5	-0.36	1.06E-05	4.94E-05	Under-expressed
TMEM254	-0.36	1.68E-05	7.37E-05	Under-expressed
CEP350	-0.36	4.41E-05	1.71E-04	Under-expressed
RAB5A	-0.36	4.97E-05	1.90E-04	Under-expressed
SCYL3	-0.36	5.93E-05	2.21E-04	Under-expressed
RNF13	-0.36	6.57E-05	2.42E-04	Under-expressed
ARHGAP35	-0.36	9.12E-05	3.23E-04	Under-expressed
SIPA1L1	-0.36	1.11E-04	3.84E-04	Under-expressed
TBCCD1	-0.36	1.39E-04	4.66E-04	Under-expressed
CCDC50	-0.36	1.43E-04	4.79E-04	Under-expressed
KAT7	-0.36	1.44E-04	4.82E-04	Under-expressed

SNX1	-0.36	1.75E-04	5.74E-04	Under-expressed
YME1L1	-0.36	1.92E-04	6.22E-04	Under-expressed
PLEKHA3	-0.36	2.04E-04	6.55E-04	Under-expressed
PPP2R5B	-0.36	2.50E-04	7.80E-04	Under-expressed
SUZ12	-0.36	3.19E-04	9.65E-04	Under-expressed
LOC100129034	-0.36	3.34E-04	1.01E-03	Under-expressed
LAMP1	-0.36	3.58E-04	1.07E-03	Under-expressed
WDR82	-0.36	5.01E-04	1.43E-03	Under-expressed
NPTN	-0.36	5.19E-04	1.47E-03	Under-expressed
RAB12	-0.36	5.32E-04	1.50E-03	Under-expressed
BRD2	-0.36	6.12E-04	1.69E-03	Under-expressed
CTNND1	-0.36	6.52E-04	1.80E-03	Under-expressed
ZNF823	-0.36	7.93E-04	2.13E-03	Under-expressed
LANCL1	-0.36	8.27E-04	2.21E-03	Under-expressed
NMRK1	-0.36	8.50E-04	2.26E-03	Under-expressed
TWF1	-0.36	8.83E-04	2.34E-03	Under-expressed
ARV1	-0.36	9.07E-04	2.39E-03	Under-expressed
GEN1	-0.36	9.13E-04	2.41E-03	Under-expressed
UPF2	-0.36	9.53E-04	2.50E-03	Under-expressed
PAFAH2	-0.36	1.07E-03	2.75E-03	Under-expressed
MAP3K7	-0.36	1.78E-03	4.31E-03	Under-expressed
PALB2	-0.36	1.79E-03	4.32E-03	Under-expressed
TASOR2	-0.36	1.84E-03	4.42E-03	Under-expressed
AGK	-0.36	2.01E-03	4.78E-03	Under-expressed
SIAH2	-0.36	2.10E-03	4.99E-03	Under-expressed
TRIM23	-0.36	2.22E-03	5.23E-03	Under-expressed
NXT2	-0.36	2.57E-03	5.96E-03	Under-expressed
ERI1	-0.36	2.82E-03	6.46E-03	Under-expressed
APMAP	-0.36	2.85E-03	6.51E-03	Under-expressed
SLC49A4	-0.36	2.92E-03	6.65E-03	Under-expressed
CTPS1	-0.36	3.25E-03	7.31E-03	Under-expressed
ABL1	-0.36	3.77E-03	8.28E-03	Under-expressed
HSPA13	-0.36	4.07E-03	8.89E-03	Under-expressed
TBC1D16	-0.36	5.14E-03	0.0109	Under-expressed
BAZ2B	-0.36	5.67E-03	0.0119	Under-expressed
GNB5	-0.36	6.60E-03	0.0136	Under-expressed
ACVR1B	-0.36	7.58E-03	0.0153	Under-expressed
SPIN3	-0.36	7.73E-03	0.0156	Under-expressed
BRPF3	-0.36	7.98E-03	0.016	Under-expressed
RAB6C	-0.36	9.65E-03	0.0189	Under-expressed
STK38L	-0.36	0.0104	0.0202	Under-expressed
KLHL21	-0.36	0.011	0.0212	Under-expressed
MAGI3	-0.36	0.0111	0.0215	Under-expressed
KIAA0040	-0.36	0.0112	0.0216	Under-expressed
BCDIN3D-AS1	-0.36	0.0116	0.0222	Under-expressed
TJP2	-0.36	0.0125	0.0238	Under-expressed
MAP4K5	-0.36	0.0164	0.03	Under-expressed
SMIM19	-0.36	0.0213	0.038	Under-expressed
IFNGR1	-0.36	0.0247	0.0433	Under-expressed
SLC35G1	-0.36	0.0253	0.0441	Under-expressed
BTBD6	-0.36	0.0279	0.0482	Under-expressed
NAPEPLD	-0.37	1.16E-06	7.24E-06	Under-expressed
DCUN1D4	-0.37	1.52E-05	6.75E-05	Under-expressed
LARP7	-0.37	2.16E-05	9.19E-05	Under-expressed
PHKB	-0.37	2.61E-05	1.09E-04	Under-expressed
NMD3	-0.37	3.14E-05	1.27E-04	Under-expressed
SPTY2D1	-0.37	3.65E-05	1.45E-04	Under-expressed

LIN7C	-0.37	4.94E-05	1.89E-04	Under-expressed
TRAPPC10	-0.37	5.91E-05	2.21E-04	Under-expressed
POFUT1	-0.37	6.62E-05	2.44E-04	Under-expressed
CLDND1	-0.37	8.17E-05	2.94E-04	Under-expressed
CAST	-0.37	8.71E-05	3.11E-04	Under-expressed
STIM2	-0.37	9.02E-05	3.20E-04	Under-expressed
OMA1	-0.37	1.23E-04	4.22E-04	Under-expressed
ALS2	-0.37	1.37E-04	4.62E-04	Under-expressed
MFN1	-0.37	1.46E-04	4.88E-04	Under-expressed
RNF115	-0.37	1.76E-04	5.77E-04	Under-expressed
GDAP2	-0.37	2.41E-04	7.56E-04	Under-expressed
CDK13	-0.37	2.77E-04	8.52E-04	Under-expressed
NUP58	-0.37	2.96E-04	9.04E-04	Under-expressed
SUCLA2	-0.37	3.97E-04	1.17E-03	Under-expressed
TUBGCP3	-0.37	4.06E-04	1.19E-03	Under-expressed
LRRC8D	-0.37	4.61E-04	1.33E-03	Under-expressed
ZNF500	-0.37	6.53E-04	1.80E-03	Under-expressed
ZCCHC4	-0.37	6.82E-04	1.87E-03	Under-expressed
PRDX3	-0.37	7.23E-04	1.96E-03	Under-expressed
COPA	-0.37	7.49E-04	2.03E-03	Under-expressed
PER2	-0.37	9.45E-04	2.48E-03	Under-expressed
GON4L	-0.37	9.61E-04	2.52E-03	Under-expressed
CRADD	-0.37	9.89E-04	2.58E-03	Under-expressed
TOP1	-0.37	1.08E-03	2.78E-03	Under-expressed
PLPP6	-0.37	1.52E-03	3.75E-03	Under-expressed
PDLIM2	-0.37	1.53E-03	3.77E-03	Under-expressed
TPCN2	-0.37	1.60E-03	3.92E-03	Under-expressed
ZNF782	-0.37	1.61E-03	3.93E-03	Under-expressed
ATL3	-0.37	1.76E-03	4.26E-03	Under-expressed
PPIL4	-0.37	1.81E-03	4.37E-03	Under-expressed
CBLB	-0.37	1.87E-03	4.48E-03	Under-expressed
ZBTB49	-0.37	1.89E-03	4.54E-03	Under-expressed
ZC3H7A	-0.37	1.97E-03	4.70E-03	Under-expressed
SMPD1	-0.37	2.32E-03	5.44E-03	Under-expressed
SRPK2	-0.37	2.39E-03	5.57E-03	Under-expressed
COPB1	-0.37	2.42E-03	5.63E-03	Under-expressed
SRGAP2	-0.37	2.55E-03	5.90E-03	Under-expressed
BPTF	-0.37	2.71E-03	6.23E-03	Under-expressed
TPST2	-0.37	3.18E-03	7.16E-03	Under-expressed
OSBPL9	-0.37	3.43E-03	7.65E-03	Under-expressed
PGAM5	-0.37	4.88E-03	0.0104	Under-expressed
SCLT1	-0.37	6.43E-03	0.0133	Under-expressed
ADAM15	-0.37	6.85E-03	0.014	Under-expressed
SEC61A2	-0.37	6.98E-03	0.0143	Under-expressed
STAG3L3	-0.37	0.0102	0.0198	Under-expressed
ZBTB8A	-0.37	0.0118	0.0225	Under-expressed
B2M	-0.37	0.0127	0.0241	Under-expressed
IGSF8	-0.37	0.016	0.0294	Under-expressed
MCAM	-0.37	0.0164	0.0301	Under-expressed
PPP1R3D	-0.37	0.0185	0.0334	Under-expressed
KLF6	-0.37	0.0244	0.0429	Under-expressed
SPARC	-0.37	0.0258	0.0449	Under-expressed
IDI1	-0.37	0.0272	0.0472	Under-expressed
NEAT1	-0.37	0.0286	0.0493	Under-expressed
UBQLN1	-0.38	8.27E-09	1.04E-07	Under-expressed
TAOK2	-0.38	1.06E-06	6.71E-06	Under-expressed
TSPYL1	-0.38	5.95E-06	2.99E-05	Under-expressed

FAN1	-0.38	6.04E-06	3.02E-05	Under-expressed
TBC1D9B	-0.38	1.85E-05	8.02E-05	Under-expressed
CDC40	-0.38	2.50E-05	1.05E-04	Under-expressed
SON	-0.38	2.78E-05	1.14E-04	Under-expressed
SDHC	-0.38	3.02E-05	1.23E-04	Under-expressed
FBXO28	-0.38	3.49E-05	1.40E-04	Under-expressed
OXNAD1	-0.38	3.57E-05	1.43E-04	Under-expressed
PHACTR4	-0.38	5.41E-05	2.05E-04	Under-expressed
UACA	-0.38	6.16E-05	2.29E-04	Under-expressed
SRRM2	-0.38	8.81E-05	3.14E-04	Under-expressed
DDX3X	-0.38	1.15E-04	3.97E-04	Under-expressed
MTMR10	-0.38	1.42E-04	4.76E-04	Under-expressed
ZNF407	-0.38	1.46E-04	4.88E-04	Under-expressed
FBXL4	-0.38	1.83E-04	5.97E-04	Under-expressed
EHMT1	-0.38	2.03E-04	6.53E-04	Under-expressed
MOCS2	-0.38	2.56E-04	7.97E-04	Under-expressed
MCEE	-0.38	2.65E-04	8.20E-04	Under-expressed
GOPC	-0.38	3.11E-04	9.45E-04	Under-expressed
ACTR2	-0.38	3.19E-04	9.65E-04	Under-expressed
IARS2	-0.38	3.20E-04	9.69E-04	Under-expressed
AGPAT3	-0.38	3.27E-04	9.87E-04	Under-expressed
PI4KA	-0.38	3.47E-04	1.04E-03	Under-expressed
STRN3	-0.38	3.83E-04	1.13E-03	Under-expressed
KDM4C	-0.38	4.62E-04	1.33E-03	Under-expressed
HTATSF1	-0.38	5.18E-04	1.47E-03	Under-expressed
DGKQ	-0.38	6.34E-04	1.75E-03	Under-expressed
DNAJC28	-0.38	1.07E-03	2.76E-03	Under-expressed
DIPK2B	-0.38	1.11E-03	2.85E-03	Under-expressed
ADARB1	-0.38	1.39E-03	3.47E-03	Under-expressed
ZNF17	-0.38	1.58E-03	3.88E-03	Under-expressed
LRBA	-0.38	2.97E-03	6.75E-03	Under-expressed
KIF21A	-0.38	3.22E-03	7.25E-03	Under-expressed
ITPK1	-0.38	3.43E-03	7.64E-03	Under-expressed
CTDSPL2	-0.38	3.88E-03	8.50E-03	Under-expressed
CRAMP1	-0.38	4.08E-03	8.90E-03	Under-expressed
SIGMAR1	-0.38	4.19E-03	9.10E-03	Under-expressed
FH	-0.38	4.67E-03	0.01	Under-expressed
TIGAR	-0.38	5.89E-03	0.0123	Under-expressed
TNFRSF10B	-0.38	6.60E-03	0.0136	Under-expressed
EXOC3L1	-0.38	7.00E-03	0.0143	Under-expressed
EEPD1	-0.38	7.10E-03	0.0145	Under-expressed
RFC3	-0.38	9.12E-03	0.018	Under-expressed
CRLF3	-0.38	9.71E-03	0.019	Under-expressed
ANXA6	-0.38	9.93E-03	0.0194	Under-expressed
SPRY4	-0.38	0.0107	0.0206	Under-expressed
TPK1	-0.38	0.0115	0.0221	Under-expressed
SMPDL3A	-0.38	0.0133	0.0251	Under-expressed
TFCP2	-0.38	0.0171	0.0312	Under-expressed
CASK	-0.38	0.0188	0.034	Under-expressed
TREX1	-0.38	0.0269	0.0467	Under-expressed
RNF123	-0.39	6.30E-08	5.98E-07	Under-expressed
SEC62	-0.39	1.04E-07	9.23E-07	Under-expressed
ZBTB14	-0.39	6.03E-07	4.15E-06	Under-expressed
TRIM26	-0.39	4.52E-06	2.35E-05	Under-expressed
ITCH	-0.39	7.19E-06	3.52E-05	Under-expressed
ZSWIM8	-0.39	7.91E-06	3.82E-05	Under-expressed
HNRNPH2	-0.39	8.86E-06	4.23E-05	Under-expressed

COX18	-0.39	9.47E-06	4.48E-05	Under-expressed
PIGN	-0.39	1.06E-05	4.94E-05	Under-expressed
NSUN3	-0.39	1.14E-05	5.28E-05	Under-expressed
SRRM1	-0.39	1.62E-05	7.16E-05	Under-expressed
TOP1P1	-0.39	1.63E-05	7.17E-05	Under-expressed
SLC30A7	-0.39	1.64E-05	7.23E-05	Under-expressed
STAM2	-0.39	1.78E-05	7.73E-05	Under-expressed
USP9X	-0.39	2.05E-05	8.77E-05	Under-expressed
YY1	-0.39	2.16E-05	9.18E-05	Under-expressed
MAGT1	-0.39	2.46E-05	1.03E-04	Under-expressed
FAM114A2	-0.39	3.98E-05	1.57E-04	Under-expressed
RAB18	-0.39	4.08E-05	1.60E-04	Under-expressed
PTPRJ	-0.39	5.03E-05	1.92E-04	Under-expressed
PLBD2	-0.39	5.33E-05	2.02E-04	Under-expressed
LRRFIP1	-0.39	6.27E-05	2.33E-04	Under-expressed
PRKAR1A	-0.39	7.49E-05	2.72E-04	Under-expressed
RWDD2B	-0.39	1.10E-04	3.82E-04	Under-expressed
FNIP1	-0.39	1.20E-04	4.11E-04	Under-expressed
SLX4	-0.39	1.74E-04	5.70E-04	Under-expressed
ERCC6	-0.39	1.98E-04	6.37E-04	Under-expressed
MTRR	-0.39	1.98E-04	6.40E-04	Under-expressed
MTF1	-0.39	2.68E-04	8.27E-04	Under-expressed
USP13	-0.39	3.94E-04	1.16E-03	Under-expressed
TRIM14	-0.39	4.04E-04	1.18E-03	Under-expressed
UAP1	-0.39	5.19E-04	1.47E-03	Under-expressed
SCAF11	-0.39	7.14E-04	1.94E-03	Under-expressed
KIAA0355	-0.39	1.04E-03	2.70E-03	Under-expressed
CDK17	-0.39	1.14E-03	2.91E-03	Under-expressed
USP45	-0.39	1.41E-03	3.51E-03	Under-expressed
RAD50	-0.39	1.52E-03	3.75E-03	Under-expressed
RAB8B	-0.39	1.60E-03	3.92E-03	Under-expressed
LRRC8C	-0.39	1.82E-03	4.37E-03	Under-expressed
NFKBIA	-0.39	1.97E-03	4.70E-03	Under-expressed
PLEKHH3	-0.39	1.98E-03	4.72E-03	Under-expressed
JADE3	-0.39	1.98E-03	4.72E-03	Under-expressed
ZMYM5	-0.39	2.86E-03	6.54E-03	Under-expressed
ING3	-0.39	2.87E-03	6.56E-03	Under-expressed
ESAM	-0.39	2.97E-03	6.74E-03	Under-expressed
ASAH1	-0.39	3.07E-03	6.94E-03	Under-expressed
PCTP	-0.39	3.87E-03	8.48E-03	Under-expressed
STIMATE	-0.39	4.30E-03	9.33E-03	Under-expressed
PFKFB2	-0.39	4.31E-03	9.34E-03	Under-expressed
DNHD1	-0.39	4.90E-03	0.0105	Under-expressed
CEP85	-0.39	5.25E-03	0.0111	Under-expressed
ERAP1	-0.39	6.44E-03	0.0133	Under-expressed
TEF	-0.39	7.24E-03	0.0147	Under-expressed
LMNTD2	-0.39	7.71E-03	0.0156	Under-expressed
LINC01000	-0.39	8.75E-03	0.0174	Under-expressed
NRROS	-0.39	9.76E-03	0.0191	Under-expressed
HERC2P2	-0.39	0.0116	0.0223	Under-expressed
ZNF615	-0.39	0.0121	0.0231	Under-expressed
TTC30A	-0.39	0.0125	0.0237	Under-expressed
ZNF304	-0.39	0.0125	0.0237	Under-expressed
MYO6	-0.39	0.0132	0.0248	Under-expressed
MAP3K10	-0.39	0.0152	0.0281	Under-expressed
RBBP8	-0.39	0.0161	0.0296	Under-expressed
PRPS2	-0.39	0.0165	0.0302	Under-expressed

CHST15	-0.39	0.0166	0.0304	Under-expressed
INPP1	-0.39	0.0181	0.0328	Under-expressed
ELL2	-0.39	0.0215	0.0382	Under-expressed
NHLRC1	-0.39	0.0238	0.0419	Under-expressed
SLC4A7	-0.39	0.0259	0.0451	Under-expressed
FPR3	-0.39	0.0276	0.0477	Under-expressed
FBF1	-0.39	0.0283	0.0488	Under-expressed
RMDN3	-0.4	7.37E-09	9.48E-08	Under-expressed
ZSCAN32	-0.4	2.14E-07	1.71E-06	Under-expressed
DCAF8	-0.4	4.20E-07	3.04E-06	Under-expressed
DCAF1	-0.4	1.56E-06	9.34E-06	Under-expressed
UBR7	-0.4	1.89E-06	1.10E-05	Under-expressed
STAT5B	-0.4	4.39E-06	2.30E-05	Under-expressed
MED13	-0.4	4.52E-06	2.35E-05	Under-expressed
SERINC3	-0.4	4.59E-06	2.38E-05	Under-expressed
AKT1	-0.4	8.59E-06	4.12E-05	Under-expressed
TMEM30A	-0.4	1.38E-05	6.20E-05	Under-expressed
DLST	-0.4	2.46E-05	1.03E-04	Under-expressed
USP33	-0.4	2.75E-05	1.14E-04	Under-expressed
TMTC3	-0.4	2.76E-05	1.14E-04	Under-expressed
AFG3L2	-0.4	2.88E-05	1.18E-04	Under-expressed
ATXN3	-0.4	4.25E-05	1.66E-04	Under-expressed
HBP1	-0.4	4.88E-05	1.87E-04	Under-expressed
ANKRD28	-0.4	6.78E-05	2.49E-04	Under-expressed
PRPS1	-0.4	9.66E-05	3.40E-04	Under-expressed
ZNF721	-0.4	1.03E-04	3.61E-04	Under-expressed
TMEM19	-0.4	1.31E-04	4.46E-04	Under-expressed
RAB2B	-0.4	1.38E-04	4.65E-04	Under-expressed
TUBGCP4	-0.4	3.05E-04	9.29E-04	Under-expressed
LBX2-AS1	-0.4	3.22E-04	9.73E-04	Under-expressed
MAP2K1	-0.4	3.55E-04	1.06E-03	Under-expressed
CLASP1	-0.4	4.06E-04	1.19E-03	Under-expressed
ELK4	-0.4	5.58E-04	1.57E-03	Under-expressed
PUS7L	-0.4	5.73E-04	1.60E-03	Under-expressed
ZBTB44	-0.4	5.98E-04	1.66E-03	Under-expressed
APAF1	-0.4	6.39E-04	1.76E-03	Under-expressed
ATRX	-0.4	7.32E-04	1.99E-03	Under-expressed
FAM13B	-0.4	7.41E-04	2.01E-03	Under-expressed
DHX40	-0.4	7.53E-04	2.04E-03	Under-expressed
HYOU1	-0.4	8.61E-04	2.29E-03	Under-expressed
SGMS1	-0.4	8.81E-04	2.33E-03	Under-expressed
EPC2	-0.4	9.29E-04	2.44E-03	Under-expressed
SEC23IP	-0.4	1.01E-03	2.61E-03	Under-expressed
CFAP97	-0.4	1.27E-03	3.19E-03	Under-expressed
SMCHD1	-0.4	1.47E-03	3.64E-03	Under-expressed
NCKAP1	-0.4	1.65E-03	4.02E-03	Under-expressed
NFKB1	-0.4	1.83E-03	4.39E-03	Under-expressed
MTSS2	-0.4	2.16E-03	5.12E-03	Under-expressed
ARHGEF40	-0.4	2.19E-03	5.17E-03	Under-expressed
KANSL1	-0.4	2.59E-03	5.99E-03	Under-expressed
TSC22D1	-0.4	2.60E-03	6.02E-03	Under-expressed
ZNF442	-0.4	2.92E-03	6.64E-03	Under-expressed
NDE1	-0.4	2.99E-03	6.78E-03	Under-expressed
DIAPH2	-0.4	3.06E-03	6.92E-03	Under-expressed
RBM23	-0.4	3.33E-03	7.46E-03	Under-expressed
PTER	-0.4	3.52E-03	7.83E-03	Under-expressed
MFSD14B	-0.4	3.77E-03	8.28E-03	Under-expressed

PHLDB2	-0.4	4.64E-03	9.98E-03	Under-expressed
ZNF253	-0.4	5.09E-03	0.0108	Under-expressed
FLI1	-0.4	5.38E-03	0.0113	Under-expressed
LRIF1	-0.4	6.22E-03	0.0129	Under-expressed
ZNF765	-0.4	8.29E-03	0.0166	Under-expressed
WWC3	-0.4	8.92E-03	0.0177	Under-expressed
MSRA	-0.4	9.27E-03	0.0183	Under-expressed
FLVCR2	-0.4	0.0109	0.0211	Under-expressed
VEGFC	-0.4	0.0129	0.0243	Under-expressed
FAM98B	-0.4	0.0138	0.0259	Under-expressed
ATP11A	-0.4	0.0144	0.0268	Under-expressed
TMPPE	-0.4	0.0148	0.0275	Under-expressed
AGPAT5	-0.4	0.0168	0.0307	Under-expressed
FUT10	-0.4	0.0172	0.0313	Under-expressed
KCTD12	-0.4	0.0204	0.0365	Under-expressed
PDXDC2P-NPIPB14P	-0.4	0.0233	0.0411	Under-expressed
ANXA7	-0.41	1.52E-08	1.76E-07	Under-expressed
EIF4G1	-0.41	1.04E-07	9.26E-07	Under-expressed
PDCD6IP	-0.41	1.13E-07	9.91E-07	Under-expressed
LARP4	-0.41	2.21E-07	1.76E-06	Under-expressed
AP1G1	-0.41	2.82E-07	2.17E-06	Under-expressed
NUMB	-0.41	3.66E-07	2.69E-06	Under-expressed
AZI2	-0.41	4.12E-07	2.99E-06	Under-expressed
RAD23B	-0.41	1.07E-06	6.79E-06	Under-expressed
SLC38A10	-0.41	2.13E-06	1.22E-05	Under-expressed
SIKE1	-0.41	2.51E-06	1.40E-05	Under-expressed
GFM2	-0.41	6.81E-06	3.35E-05	Under-expressed
EVI5L	-0.41	8.22E-06	3.95E-05	Under-expressed
CASKIN2	-0.41	1.90E-05	8.16E-05	Under-expressed
ZBTB11	-0.41	2.83E-05	1.16E-04	Under-expressed
NSMCE3	-0.41	3.53E-05	1.41E-04	Under-expressed
CLEC16A	-0.41	5.95E-05	2.22E-04	Under-expressed
RARA	-0.41	6.47E-05	2.39E-04	Under-expressed
TCAIM	-0.41	7.88E-05	2.85E-04	Under-expressed
SOS1	-0.41	8.93E-05	3.17E-04	Under-expressed
RBM41	-0.41	8.99E-05	3.19E-04	Under-expressed
HIBCH	-0.41	9.53E-05	3.36E-04	Under-expressed
PARP4	-0.41	9.82E-05	3.45E-04	Under-expressed
GNPTAB	-0.41	1.19E-04	4.08E-04	Under-expressed
GTF2IRD2	-0.41	1.54E-04	5.12E-04	Under-expressed
NDFIP2	-0.41	2.10E-04	6.70E-04	Under-expressed
EIF2AK2	-0.41	2.11E-04	6.73E-04	Under-expressed
ZNF318	-0.41	2.71E-04	8.36E-04	Under-expressed
ZNF616	-0.41	2.92E-04	8.92E-04	Under-expressed
FBXO48	-0.41	2.95E-04	9.02E-04	Under-expressed
UVSSA	-0.41	3.40E-04	1.02E-03	Under-expressed
DCAF17	-0.41	3.79E-04	1.12E-03	Under-expressed
BRCC3	-0.41	4.63E-04	1.34E-03	Under-expressed
NAF1	-0.41	5.65E-04	1.58E-03	Under-expressed
MTMR4	-0.41	7.13E-04	1.94E-03	Under-expressed
TRIM66	-0.41	7.44E-04	2.02E-03	Under-expressed
GOLPH3	-0.41	1.30E-03	3.26E-03	Under-expressed
RGCC	-0.41	1.54E-03	3.78E-03	Under-expressed
ZNF280D	-0.41	1.56E-03	3.83E-03	Under-expressed
ZNF621	-0.41	1.73E-03	4.20E-03	Under-expressed
SH3BP2	-0.41	1.83E-03	4.39E-03	Under-expressed
ASPRV1	-0.41	2.68E-03	6.17E-03	Under-expressed

ZNF697	-0.41	3.19E-03	7.19E-03	Under-expressed
SMC2	-0.41	3.85E-03	8.45E-03	Under-expressed
LRRC70	-0.41	4.46E-03	9.62E-03	Under-expressed
ANKHD1-EIF4EBP3	-0.41	5.04E-03	0.0107	Under-expressed
RAPGEF3	-0.41	5.68E-03	0.0119	Under-expressed
BBS1	-0.41	7.27E-03	0.0148	Under-expressed
JUN	-0.41	7.30E-03	0.0148	Under-expressed
SOX17	-0.41	0.014	0.0261	Under-expressed
SLC66A3	-0.41	0.0189	0.0341	Under-expressed
MAP1B	-0.41	0.025	0.0438	Under-expressed
ASAP2	-0.41	0.0251	0.0438	Under-expressed
CDKN2B	-0.41	0.0281	0.0485	Under-expressed
NAA30	-0.42	9.67E-08	8.69E-07	Under-expressed
PLAA	-0.42	1.02E-07	9.10E-07	Under-expressed
PPP3R1	-0.42	2.97E-07	2.26E-06	Under-expressed
DCAF5	-0.42	4.31E-07	3.10E-06	Under-expressed
EEF2K	-0.42	7.19E-07	4.83E-06	Under-expressed
GSK3B	-0.42	7.49E-07	4.99E-06	Under-expressed
DCUN1D3	-0.42	7.82E-06	3.79E-05	Under-expressed
TRIM37	-0.42	1.18E-05	5.42E-05	Under-expressed
VPS36	-0.42	5.11E-05	1.95E-04	Under-expressed
NUMA1	-0.42	5.48E-05	2.07E-04	Under-expressed
SHQ1	-0.42	6.31E-05	2.34E-04	Under-expressed
LIPA	-0.42	7.24E-05	2.64E-04	Under-expressed
ZNF644	-0.42	7.86E-05	2.84E-04	Under-expressed
CEP170B	-0.42	9.29E-05	3.29E-04	Under-expressed
EAF1	-0.42	1.19E-04	4.07E-04	Under-expressed
FBXO38	-0.42	1.20E-04	4.11E-04	Under-expressed
CREBL2	-0.42	1.39E-04	4.67E-04	Under-expressed
R3HDM2	-0.42	1.68E-04	5.51E-04	Under-expressed
ZNF770	-0.42	1.91E-04	6.21E-04	Under-expressed
RAB23	-0.42	2.50E-04	7.80E-04	Under-expressed
F8A1	-0.42	3.06E-04	9.31E-04	Under-expressed
ZNF484	-0.42	3.16E-04	9.59E-04	Under-expressed
TTC33	-0.42	3.46E-04	1.04E-03	Under-expressed
STAG2	-0.42	4.34E-04	1.26E-03	Under-expressed
MAT2B	-0.42	4.43E-04	1.28E-03	Under-expressed
NCSTN	-0.42	4.45E-04	1.29E-03	Under-expressed
MMACHC	-0.42	4.78E-04	1.37E-03	Under-expressed
GNA12	-0.42	6.77E-04	1.86E-03	Under-expressed
LMBRD1	-0.42	7.87E-04	2.12E-03	Under-expressed
ZNF37A	-0.42	9.52E-04	2.50E-03	Under-expressed
DMXL2	-0.42	1.02E-03	2.65E-03	Under-expressed
FOXJ2	-0.42	1.08E-03	2.78E-03	Under-expressed
DENND4A	-0.42	1.56E-03	3.84E-03	Under-expressed
PREX1	-0.42	1.59E-03	3.90E-03	Under-expressed
ATP7A	-0.42	1.69E-03	4.11E-03	Under-expressed
MAF	-0.42	1.71E-03	4.16E-03	Under-expressed
C16ORF87	-0.42	2.69E-03	6.20E-03	Under-expressed
BCL6B	-0.42	3.07E-03	6.94E-03	Under-expressed
PRKAB2	-0.42	3.42E-03	7.63E-03	Under-expressed
ZNF224	-0.42	3.44E-03	7.67E-03	Under-expressed
BMP2K	-0.42	3.68E-03	8.11E-03	Under-expressed
DNASE2	-0.42	4.52E-03	9.75E-03	Under-expressed
TRIQK	-0.42	4.69E-03	0.0101	Under-expressed
QDPR	-0.42	5.47E-03	0.0115	Under-expressed
PHACTR2	-0.42	5.69E-03	0.0119	Under-expressed

NACC2	-0.42	5.74E-03	0.012	Under-expressed
DDHD2	-0.42	5.81E-03	0.0121	Under-expressed
LONRF1	-0.42	8.89E-03	0.0176	Under-expressed
DUSP6	-0.42	9.14E-03	0.0181	Under-expressed
TTC7B	-0.42	0.0101	0.0198	Under-expressed
PLK2	-0.42	0.0115	0.022	Under-expressed
HIVEP3	-0.42	0.0128	0.0242	Under-expressed
FAH	-0.42	0.013	0.0245	Under-expressed
ZBED3	-0.42	0.0156	0.0288	Under-expressed
CBL	-0.42	0.0161	0.0297	Under-expressed
ZNF597	-0.42	0.017	0.031	Under-expressed
TRIM52	-0.42	0.0197	0.0353	Under-expressed
ABCA7	-0.42	0.0227	0.0401	Under-expressed
PRSS36	-0.42	0.0233	0.041	Under-expressed
ZNF57	-0.42	0.0246	0.0432	Under-expressed
CD9	-0.42	0.0253	0.0442	Under-expressed
KPNA1	-0.43	1.95E-10	4.27E-09	Under-expressed
TECPR2	-0.43	2.26E-07	1.79E-06	Under-expressed
SLC39A9	-0.43	4.61E-07	3.29E-06	Under-expressed
HLCS	-0.43	1.76E-06	1.03E-05	Under-expressed
RAB5C	-0.43	1.91E-06	1.11E-05	Under-expressed
EXOC8	-0.43	3.15E-06	1.71E-05	Under-expressed
SYVN1	-0.43	4.47E-06	2.34E-05	Under-expressed
HADHB	-0.43	4.96E-06	2.55E-05	Under-expressed
POT1	-0.43	6.69E-06	3.30E-05	Under-expressed
SCARB2	-0.43	7.03E-06	3.45E-05	Under-expressed
AP4E1	-0.43	1.29E-05	5.84E-05	Under-expressed
SIK3	-0.43	1.30E-05	5.89E-05	Under-expressed
DNAJC3	-0.43	1.46E-05	6.51E-05	Under-expressed
ABHD13	-0.43	1.70E-05	7.43E-05	Under-expressed
XPC	-0.43	2.16E-05	9.19E-05	Under-expressed
PBRM1	-0.43	2.82E-05	1.16E-04	Under-expressed
GALNT1	-0.43	2.90E-05	1.19E-04	Under-expressed
ARHGEF9	-0.43	3.23E-05	1.30E-04	Under-expressed
RCBTB2	-0.43	4.50E-05	1.74E-04	Under-expressed
PDE8A	-0.43	6.39E-05	2.36E-04	Under-expressed
PPP2CB	-0.43	8.76E-05	3.12E-04	Under-expressed
RTN4	-0.43	1.94E-04	6.27E-04	Under-expressed
FDX1	-0.43	2.07E-04	6.63E-04	Under-expressed
KRIT1	-0.43	2.12E-04	6.75E-04	Under-expressed
DOCK4	-0.43	2.69E-04	8.31E-04	Under-expressed
CYB5R4	-0.43	3.24E-04	9.78E-04	Under-expressed
GALK2	-0.43	4.02E-04	1.18E-03	Under-expressed
GSAP	-0.43	4.34E-04	1.26E-03	Under-expressed
CSTF2T	-0.43	5.23E-04	1.48E-03	Under-expressed
CEP70	-0.43	5.31E-04	1.50E-03	Under-expressed
PARP11	-0.43	5.44E-04	1.53E-03	Under-expressed
PNPO	-0.43	7.64E-04	2.06E-03	Under-expressed
LTB4R2	-0.43	7.99E-04	2.14E-03	Under-expressed
TNKS2	-0.43	8.29E-04	2.21E-03	Under-expressed
VCL	-0.43	1.37E-03	3.42E-03	Under-expressed
APBB3	-0.43	1.79E-03	4.32E-03	Under-expressed
LOC652276	-0.43	2.43E-03	5.66E-03	Under-expressed
DOCK7	-0.43	2.73E-03	6.27E-03	Under-expressed
CEMIP2	-0.43	3.56E-03	7.89E-03	Under-expressed
MTR	-0.43	4.79E-03	0.0103	Under-expressed
ADCY3	-0.43	5.91E-03	0.0123	Under-expressed

RUSC1-AS1	-0.43	6.41E-03	0.0132	Under-expressed
NRBP2	-0.43	8.89E-03	0.0176	Under-expressed
JCAD	-0.43	9.53E-03	0.0187	Under-expressed
IQCG	-0.43	0.0106	0.0205	Under-expressed
LRIG2	-0.43	0.0114	0.0218	Under-expressed
BCL2	-0.43	0.0147	0.0273	Under-expressed
WHRN	-0.43	0.0155	0.0286	Under-expressed
SLC43A3	-0.43	0.0161	0.0296	Under-expressed
TENT5A	-0.43	0.0162	0.0297	Under-expressed
AMER1	-0.43	0.0167	0.0306	Under-expressed
ADAMTS7	-0.43	0.0209	0.0373	Under-expressed
CPM	-0.43	0.0231	0.0408	Under-expressed
TOR1B	-0.44	4.26E-08	4.29E-07	Under-expressed
ZNF652	-0.44	1.99E-07	1.61E-06	Under-expressed
ZNF106	-0.44	7.42E-07	4.95E-06	Under-expressed
TUBGCP5	-0.44	1.07E-06	6.74E-06	Under-expressed
TK2	-0.44	1.08E-06	6.83E-06	Under-expressed
ZBED1	-0.44	1.51E-06	9.04E-06	Under-expressed
ZSCAN29	-0.44	4.38E-06	2.30E-05	Under-expressed
C18ORF25	-0.44	5.20E-06	2.65E-05	Under-expressed
FRS2	-0.44	8.94E-06	4.26E-05	Under-expressed
TMEM135	-0.44	9.51E-06	4.50E-05	Under-expressed
MAP3K3	-0.44	1.01E-05	4.72E-05	Under-expressed
ZADH2	-0.44	1.29E-05	5.84E-05	Under-expressed
USP30	-0.44	1.87E-05	8.06E-05	Under-expressed
LCMT2	-0.44	2.44E-05	1.02E-04	Under-expressed
ZNF674	-0.44	2.55E-05	1.06E-04	Under-expressed
MPP5	-0.44	3.50E-05	1.40E-04	Under-expressed
NFYB	-0.44	3.91E-05	1.54E-04	Under-expressed
DNAAF2	-0.44	5.64E-05	2.13E-04	Under-expressed
ZRANB1	-0.44	5.80E-05	2.18E-04	Under-expressed
INTS6	-0.44	6.00E-05	2.24E-04	Under-expressed
PREPL	-0.44	7.04E-05	2.57E-04	Under-expressed
ARHGEF10L	-0.44	8.38E-05	3.00E-04	Under-expressed
ICOSLG	-0.44	9.68E-05	3.41E-04	Under-expressed
NOP9	-0.44	1.23E-04	4.21E-04	Under-expressed
FBXW2	-0.44	1.26E-04	4.30E-04	Under-expressed
PCCB	-0.44	1.33E-04	4.50E-04	Under-expressed
RAB3GAP1	-0.44	1.37E-04	4.62E-04	Under-expressed
EEA1	-0.44	1.61E-04	5.31E-04	Under-expressed
QKI	-0.44	2.01E-04	6.47E-04	Under-expressed
PEX7	-0.44	2.06E-04	6.59E-04	Under-expressed
HECA	-0.44	2.08E-04	6.63E-04	Under-expressed
USP31	-0.44	2.30E-04	7.23E-04	Under-expressed
HIVEP2	-0.44	2.59E-04	8.03E-04	Under-expressed
DUSP16	-0.44	2.65E-04	8.19E-04	Under-expressed
ACYP2	-0.44	3.71E-04	1.10E-03	Under-expressed
SLC25A13	-0.44	4.21E-04	1.23E-03	Under-expressed
TAF9B	-0.44	4.55E-04	1.32E-03	Under-expressed
TANK	-0.44	5.91E-04	1.64E-03	Under-expressed
SDCBP	-0.44	6.00E-04	1.66E-03	Under-expressed
ZNF687	-0.44	7.66E-04	2.07E-03	Under-expressed
SNAPC3	-0.44	8.53E-04	2.27E-03	Under-expressed
NR2F2	-0.44	9.37E-04	2.46E-03	Under-expressed
KAT6A	-0.44	1.11E-03	2.83E-03	Under-expressed
DST	-0.44	1.36E-03	3.40E-03	Under-expressed
PRRC2B	-0.44	1.39E-03	3.47E-03	Under-expressed

TAB3	-0.44	1.41E-03	3.51E-03	Under-expressed
FAM234B	-0.44	1.42E-03	3.51E-03	Under-expressed
CD34	-0.44	1.70E-03	4.13E-03	Under-expressed
ELMSAN1	-0.44	1.75E-03	4.23E-03	Under-expressed
SH2D4A	-0.44	2.22E-03	5.23E-03	Under-expressed
SYNPO	-0.44	2.50E-03	5.80E-03	Under-expressed
NUP153	-0.44	2.64E-03	6.09E-03	Under-expressed
KCTD9	-0.44	2.71E-03	6.24E-03	Under-expressed
INSIG2	-0.44	2.97E-03	6.75E-03	Under-expressed
TMEM173	-0.44	5.21E-03	0.011	Under-expressed
DTX2P1-UPK3BP1-PMS2P11	-0.44	5.34E-03	0.0113	Under-expressed
TRIM34	-0.44	5.50E-03	0.0115	Under-expressed
IGFBP7	-0.44	5.71E-03	0.0119	Under-expressed
MEIS3P1	-0.44	8.31E-03	0.0166	Under-expressed
C2ORF16	-0.44	9.11E-03	0.018	Under-expressed
SMAD7	-0.44	0.0114	0.0218	Under-expressed
RECK	-0.44	0.0157	0.029	Under-expressed
DHODH	-0.44	0.0184	0.0333	Under-expressed
TLR5	-0.44	0.0279	0.0483	Under-expressed
AHCYL1	-0.45	6.66E-09	8.69E-08	Under-expressed
PCYT1A	-0.45	2.11E-08	2.35E-07	Under-expressed
CCDC47	-0.45	9.82E-08	8.80E-07	Under-expressed
SYNJ2BP	-0.45	1.45E-07	1.24E-06	Under-expressed
ZNF148	-0.45	2.08E-07	1.67E-06	Under-expressed
VPS4B	-0.45	1.42E-06	8.59E-06	Under-expressed
TIRAP	-0.45	1.74E-06	1.03E-05	Under-expressed
CCSER2	-0.45	2.06E-06	1.18E-05	Under-expressed
TBK1	-0.45	2.57E-06	1.43E-05	Under-expressed
VPS26C	-0.45	3.11E-06	1.69E-05	Under-expressed
YES1	-0.45	3.78E-06	2.01E-05	Under-expressed
FPGT	-0.45	5.17E-06	2.64E-05	Under-expressed
OSTM1	-0.45	5.19E-06	2.65E-05	Under-expressed
TPP1	-0.45	7.38E-06	3.60E-05	Under-expressed
FTO	-0.45	7.59E-06	3.69E-05	Under-expressed
ZNF330	-0.45	1.11E-05	5.17E-05	Under-expressed
ALG10	-0.45	1.39E-05	6.24E-05	Under-expressed
ARFIP1	-0.45	2.14E-05	9.12E-05	Under-expressed
MBTPS2	-0.45	2.70E-05	1.12E-04	Under-expressed
HELZ	-0.45	3.47E-05	1.39E-04	Under-expressed
SNTB2	-0.45	3.71E-05	1.47E-04	Under-expressed
MLXIP	-0.45	3.73E-05	1.48E-04	Under-expressed
ZNF91	-0.45	7.43E-05	2.70E-04	Under-expressed
KLHL36	-0.45	9.28E-05	3.28E-04	Under-expressed
LACTB	-0.45	9.86E-05	3.46E-04	Under-expressed
ERC1	-0.45	1.12E-04	3.87E-04	Under-expressed
PSD4	-0.45	1.17E-04	4.04E-04	Under-expressed
WDR19	-0.45	1.77E-04	5.79E-04	Under-expressed
RALGAPA1	-0.45	1.85E-04	6.00E-04	Under-expressed
CPPED1	-0.45	2.00E-04	6.45E-04	Under-expressed
IFIT5	-0.45	2.07E-04	6.62E-04	Under-expressed
ZNF609	-0.45	2.17E-04	6.89E-04	Under-expressed
IL17RC	-0.45	2.25E-04	7.13E-04	Under-expressed
TMOD3	-0.45	2.27E-04	7.16E-04	Under-expressed
NUBPL	-0.45	2.36E-04	7.41E-04	Under-expressed
MCCC1	-0.45	2.36E-04	7.41E-04	Under-expressed
DNAJB5	-0.45	2.44E-04	7.63E-04	Under-expressed
MAPK1	-0.45	2.53E-04	7.88E-04	Under-expressed

SPTLC2	-0.45	2.84E-04	8.71E-04	Under-expressed
C1RL	-0.45	3.34E-04	1.01E-03	Under-expressed
AGTPBP1	-0.45	3.86E-04	1.14E-03	Under-expressed
CASD1	-0.45	4.00E-04	1.17E-03	Under-expressed
TMEM14A	-0.45	4.86E-04	1.39E-03	Under-expressed
SESN1	-0.45	5.32E-04	1.50E-03	Under-expressed
SLC24A1	-0.45	6.62E-04	1.82E-03	Under-expressed
TNKS	-0.45	6.96E-04	1.90E-03	Under-expressed
TGDS	-0.45	7.89E-04	2.12E-03	Under-expressed
DCBLD2	-0.45	8.11E-04	2.17E-03	Under-expressed
RXRA	-0.45	8.67E-04	2.30E-03	Under-expressed
ITGA6	-0.45	1.06E-03	2.74E-03	Under-expressed
LRP1	-0.45	1.32E-03	3.31E-03	Under-expressed
ATXN1	-0.45	1.50E-03	3.69E-03	Under-expressed
CHPT1	-0.45	1.64E-03	4.00E-03	Under-expressed
PCDH12	-0.45	1.69E-03	4.10E-03	Under-expressed
RALGPS2	-0.45	1.78E-03	4.30E-03	Under-expressed
RB1	-0.45	2.18E-03	5.15E-03	Under-expressed
IDS	-0.45	2.24E-03	5.28E-03	Under-expressed
BOC	-0.45	3.49E-03	7.77E-03	Under-expressed
DIXDC1	-0.45	4.01E-03	8.76E-03	Under-expressed
SLC19A2	-0.45	4.85E-03	0.0104	Under-expressed
CROT	-0.45	5.06E-03	0.0107	Under-expressed
JRKL	-0.45	5.57E-03	0.0117	Under-expressed
MLLT10	-0.45	6.93E-03	0.0142	Under-expressed
C19ORF57	-0.45	8.52E-03	0.017	Under-expressed
CSRP1	-0.45	0.0131	0.0246	Under-expressed
SMC3	-0.45	0.0188	0.0339	Under-expressed
ARAP2	-0.45	0.023	0.0406	Under-expressed
RNF185	-0.46	1.04E-10	2.56E-09	Under-expressed
TADA2B	-0.46	1.83E-10	4.04E-09	Under-expressed
CALCOCO2	-0.46	4.15E-09	5.78E-08	Under-expressed
TCEANC	-0.46	2.44E-08	2.66E-07	Under-expressed
KLF3	-0.46	6.48E-08	6.13E-07	Under-expressed
LRRC8A	-0.46	9.95E-08	8.89E-07	Under-expressed
KIAA0232	-0.46	1.63E-07	1.37E-06	Under-expressed
MKLN1	-0.46	4.72E-07	3.35E-06	Under-expressed
EIF2AK4	-0.46	4.98E-07	3.52E-06	Under-expressed
FAM160B1	-0.46	7.42E-07	4.95E-06	Under-expressed
NFATC2IP	-0.46	1.23E-06	7.59E-06	Under-expressed
TAF5L	-0.46	1.25E-06	7.67E-06	Under-expressed
ATP2C1	-0.46	1.26E-06	7.77E-06	Under-expressed
TMEM175	-0.46	1.42E-06	8.59E-06	Under-expressed
IBTK	-0.46	2.08E-06	1.19E-05	Under-expressed
APPBP2	-0.46	2.55E-06	1.43E-05	Under-expressed
PUS10	-0.46	2.91E-06	1.60E-05	Under-expressed
SLC25A44	-0.46	3.18E-06	1.72E-05	Under-expressed
CDC73	-0.46	3.96E-06	2.10E-05	Under-expressed
MFAP3	-0.46	6.27E-06	3.12E-05	Under-expressed
RSBN1L	-0.46	6.97E-06	3.43E-05	Under-expressed
TRIM68	-0.46	7.26E-06	3.55E-05	Under-expressed
TBC1D14	-0.46	9.90E-06	4.65E-05	Under-expressed
TMLHE	-0.46	1.04E-05	4.88E-05	Under-expressed
SARAF	-0.46	1.06E-05	4.93E-05	Under-expressed
ESYT2	-0.46	1.06E-05	4.96E-05	Under-expressed
RNF111	-0.46	1.46E-05	6.51E-05	Under-expressed
GPR4	-0.46	2.78E-05	1.15E-04	Under-expressed

MYD88	-0.46	2.98E-05	1.22E-04	Under-expressed
VPS50	-0.46	4.17E-05	1.63E-04	Under-expressed
MYH9	-0.46	4.26E-05	1.66E-04	Under-expressed
6-Mar	-0.46	5.90E-05	2.21E-04	Under-expressed
BRD4	-0.46	6.55E-05	2.41E-04	Under-expressed
PPM1D	-0.46	8.04E-05	2.90E-04	Under-expressed
MAPK6	-0.46	9.21E-05	3.26E-04	Under-expressed
TMF1	-0.46	9.74E-05	3.43E-04	Under-expressed
MFSD14A	-0.46	1.00E-04	3.50E-04	Under-expressed
KLHL5	-0.46	1.23E-04	4.22E-04	Under-expressed
TMED5	-0.46	1.35E-04	4.57E-04	Under-expressed
MTMR1	-0.46	1.42E-04	4.74E-04	Under-expressed
INIP	-0.46	1.81E-04	5.89E-04	Under-expressed
MAN2A2	-0.46	2.31E-04	7.26E-04	Under-expressed
RBBP9	-0.46	2.46E-04	7.70E-04	Under-expressed
AGO3	-0.46	2.75E-04	8.47E-04	Under-expressed
TP53INP2	-0.46	3.49E-04	1.04E-03	Under-expressed
ASCC3	-0.46	4.32E-04	1.26E-03	Under-expressed
GPATCH8	-0.46	4.49E-04	1.30E-03	Under-expressed
PANX1	-0.46	4.53E-04	1.31E-03	Under-expressed
ATM	-0.46	5.36E-04	1.51E-03	Under-expressed
RFFL	-0.46	6.75E-04	1.85E-03	Under-expressed
VPS37A	-0.46	6.84E-04	1.87E-03	Under-expressed
ANKIB1	-0.46	8.32E-04	2.22E-03	Under-expressed
DHCR24	-0.46	1.61E-03	3.94E-03	Under-expressed
TCF12	-0.46	1.76E-03	4.26E-03	Under-expressed
DHFR	-0.46	2.11E-03	5.01E-03	Under-expressed
LAP3	-0.46	3.23E-03	7.26E-03	Under-expressed
TEC	-0.46	3.71E-03	8.16E-03	Under-expressed
PGAP1	-0.46	5.47E-03	0.0115	Under-expressed
DDTL	-0.46	7.58E-03	0.0153	Under-expressed
CCDC88A	-0.46	7.68E-03	0.0155	Under-expressed
CFI	-0.46	9.08E-03	0.018	Under-expressed
ZNF254	-0.46	0.0109	0.0211	Under-expressed
NADK2	-0.46	0.012	0.0229	Under-expressed
DDO	-0.46	0.0127	0.0241	Under-expressed
IFT80	-0.46	0.0179	0.0325	Under-expressed
TLN2	-0.46	0.0198	0.0356	Under-expressed
FBXL7	-0.46	0.0222	0.0393	Under-expressed
SIK1	-0.46	0.0233	0.0411	Under-expressed
ABCC3	-0.46	0.0246	0.0431	Under-expressed
NEK9	-0.47	2.06E-07	1.66E-06	Under-expressed
DHX36	-0.47	1.16E-06	7.23E-06	Under-expressed
TLN1	-0.47	1.43E-06	8.61E-06	Under-expressed
IREB2	-0.47	2.13E-06	1.22E-05	Under-expressed
YIPF6	-0.47	2.56E-06	1.43E-05	Under-expressed
MON2	-0.47	2.76E-06	1.52E-05	Under-expressed
TARS2	-0.47	3.61E-06	1.93E-05	Under-expressed
GNAQ	-0.47	6.91E-06	3.40E-05	Under-expressed
FUBP3	-0.47	7.99E-06	3.85E-05	Under-expressed
EIF4G3	-0.47	9.60E-06	4.54E-05	Under-expressed
XIAP	-0.47	1.34E-05	6.04E-05	Under-expressed
TPP2	-0.47	1.63E-05	7.19E-05	Under-expressed
RNF103	-0.47	1.96E-05	8.44E-05	Under-expressed
FOXO4	-0.47	2.32E-05	9.75E-05	Under-expressed
TOB2	-0.47	2.74E-05	1.13E-04	Under-expressed
RBAK	-0.47	2.84E-05	1.17E-04	Under-expressed

LMBR1	-0.47	3.88E-05	1.53E-04	Under-expressed
EPG5	-0.47	4.43E-05	1.72E-04	Under-expressed
ZNF417	-0.47	6.17E-05	2.29E-04	Under-expressed
SUN1	-0.47	7.16E-05	2.61E-04	Under-expressed
BDP1	-0.47	9.84E-05	3.46E-04	Under-expressed
TTC14	-0.47	1.31E-04	4.44E-04	Under-expressed
ATP9B	-0.47	1.61E-04	5.31E-04	Under-expressed
TIPARP	-0.47	1.75E-04	5.71E-04	Under-expressed
FBXO34	-0.47	2.30E-04	7.25E-04	Under-expressed
ZNF443	-0.47	2.48E-04	7.75E-04	Under-expressed
KMT2D	-0.47	2.71E-04	8.36E-04	Under-expressed
LPP	-0.47	3.18E-04	9.64E-04	Under-expressed
FYN	-0.47	3.45E-04	1.04E-03	Under-expressed
ARID1B	-0.47	3.99E-04	1.17E-03	Under-expressed
MTHFS	-0.47	5.65E-04	1.58E-03	Under-expressed
PDIA3P1	-0.47	5.98E-04	1.66E-03	Under-expressed
SPTSSA	-0.47	7.61E-04	2.05E-03	Under-expressed
MSN	-0.47	8.55E-04	2.28E-03	Under-expressed
RECQL	-0.47	1.06E-03	2.74E-03	Under-expressed
MIB1	-0.47	1.37E-03	3.41E-03	Under-expressed
NSD3	-0.47	1.40E-03	3.48E-03	Under-expressed
CHD6	-0.47	1.42E-03	3.53E-03	Under-expressed
NCAPD3	-0.47	2.79E-03	6.39E-03	Under-expressed
CEP192	-0.47	3.41E-03	7.62E-03	Under-expressed
SERPINB9	-0.47	3.49E-03	7.76E-03	Under-expressed
ZNF433	-0.47	3.88E-03	8.50E-03	Under-expressed
BAHCC1	-0.47	4.60E-03	9.90E-03	Under-expressed
NAV1	-0.47	4.60E-03	9.91E-03	Under-expressed
WDFY3	-0.47	5.20E-03	0.011	Under-expressed
RND3	-0.47	6.96E-03	0.0142	Under-expressed
MRAS	-0.47	8.60E-03	0.0171	Under-expressed
TLE4	-0.47	0.0151	0.0281	Under-expressed
GUCY1B1	-0.47	0.0215	0.0383	Under-expressed
PSTPIP2	-0.47	0.0219	0.0389	Under-expressed
C5	-0.47	0.027	0.0469	Under-expressed
TNFAIP1	-0.48	1.94E-10	4.25E-09	Under-expressed
SACM1L	-0.48	2.10E-10	4.55E-09	Under-expressed
GOLGA1	-0.48	5.13E-10	9.78E-09	Under-expressed
ARNT	-0.48	1.83E-07	1.50E-06	Under-expressed
MFN2	-0.48	3.88E-07	2.82E-06	Under-expressed
SPRTN	-0.48	7.29E-07	4.88E-06	Under-expressed
PIGV	-0.48	1.06E-06	6.70E-06	Under-expressed
FKTN	-0.48	2.26E-06	1.28E-05	Under-expressed
KLHL7	-0.48	2.54E-06	1.42E-05	Under-expressed
PPM1B	-0.48	4.26E-06	2.24E-05	Under-expressed
AASDH	-0.48	4.30E-06	2.26E-05	Under-expressed
FRYL	-0.48	6.56E-06	3.25E-05	Under-expressed
NKIRAS1	-0.48	8.65E-06	4.14E-05	Under-expressed
LDLRAP1	-0.48	9.19E-06	4.37E-05	Under-expressed
RAB33B	-0.48	1.88E-05	8.10E-05	Under-expressed
FEM1C	-0.48	2.50E-05	1.04E-04	Under-expressed
ST20-AS1	-0.48	2.72E-05	1.12E-04	Under-expressed
KRCC1	-0.48	2.79E-05	1.15E-04	Under-expressed
TMEM123	-0.48	3.02E-05	1.23E-04	Under-expressed
FAM120B	-0.48	3.15E-05	1.28E-04	Under-expressed
BICRAL	-0.48	3.33E-05	1.34E-04	Under-expressed
SFXN1	-0.48	4.75E-05	1.83E-04	Under-expressed

ZNF799	-0.48	6.64E-05	2.44E-04	Under-expressed
MTMR9	-0.48	7.90E-05	2.85E-04	Under-expressed
CALCOCO1	-0.48	8.69E-05	3.10E-04	Under-expressed
ARSK	-0.48	1.05E-04	3.65E-04	Under-expressed
G2E3	-0.48	1.44E-04	4.81E-04	Under-expressed
ASMTL	-0.48	1.64E-04	5.38E-04	Under-expressed
C16ORF46	-0.48	1.93E-04	6.24E-04	Under-expressed
USP35	-0.48	2.03E-04	6.53E-04	Under-expressed
CRYZ	-0.48	2.61E-04	8.10E-04	Under-expressed
BACH1	-0.48	2.65E-04	8.18E-04	Under-expressed
MED14	-0.48	3.91E-04	1.15E-03	Under-expressed
ZC3H12C	-0.48	1.11E-03	2.85E-03	Under-expressed
PLEKHA5	-0.48	1.14E-03	2.90E-03	Under-expressed
NIBAN1	-0.48	1.30E-03	3.28E-03	Under-expressed
ANTXR2	-0.48	1.36E-03	3.40E-03	Under-expressed
PATJ	-0.48	1.57E-03	3.86E-03	Under-expressed
STBD1	-0.48	1.78E-03	4.30E-03	Under-expressed
MTURN	-0.48	2.30E-03	5.38E-03	Under-expressed
HSPA12B	-0.48	2.49E-03	5.77E-03	Under-expressed
GCDH	-0.48	2.49E-03	5.78E-03	Under-expressed
GALNT10	-0.48	3.00E-03	6.81E-03	Under-expressed
TMOD2	-0.48	3.65E-03	8.05E-03	Under-expressed
LRRC34	-0.48	4.65E-03	9.99E-03	Under-expressed
ARAP3	-0.48	4.77E-03	0.0102	Under-expressed
ATL1	-0.48	6.92E-03	0.0142	Under-expressed
GJA1	-0.48	0.0101	0.0198	Under-expressed
ZNF563	-0.48	0.0183	0.0332	Under-expressed
HEPH	-0.48	0.0221	0.0391	Under-expressed
OLFML2A	-0.48	0.0259	0.0451	Under-expressed
OSBP	-0.49	8.57E-10	1.50E-08	Under-expressed
ZFP91	-0.49	1.39E-07	1.19E-06	Under-expressed
STIM1	-0.49	2.31E-07	1.82E-06	Under-expressed
CPOX	-0.49	3.39E-07	2.53E-06	Under-expressed
PPP4R3B	-0.49	9.88E-07	6.31E-06	Under-expressed
RANBP9	-0.49	1.37E-06	8.32E-06	Under-expressed
ATL2	-0.49	1.41E-06	8.55E-06	Under-expressed
AMFR	-0.49	1.75E-06	1.03E-05	Under-expressed
TRAPPC11	-0.49	2.06E-06	1.18E-05	Under-expressed
DNAJC13	-0.49	2.54E-06	1.42E-05	Under-expressed
SMG1	-0.49	4.49E-06	2.34E-05	Under-expressed
DYRK1A	-0.49	4.78E-06	2.47E-05	Under-expressed
RCOR1	-0.49	9.41E-06	4.46E-05	Under-expressed
BRWD1	-0.49	1.31E-05	5.95E-05	Under-expressed
PIAS2	-0.49	1.47E-05	6.54E-05	Under-expressed
ARID1A	-0.49	2.27E-05	9.57E-05	Under-expressed
SENP8	-0.49	3.48E-05	1.40E-04	Under-expressed
WDR24	-0.49	4.70E-05	1.81E-04	Under-expressed
SCAPER	-0.49	9.49E-05	3.35E-04	Under-expressed
PHLPP1	-0.49	1.14E-04	3.93E-04	Under-expressed
USO1	-0.49	1.28E-04	4.36E-04	Under-expressed
HMGXB4	-0.49	3.15E-04	9.55E-04	Under-expressed
IRF2BPL	-0.49	3.35E-04	1.01E-03	Under-expressed
GK5	-0.49	3.76E-04	1.11E-03	Under-expressed
SLK	-0.49	3.94E-04	1.16E-03	Under-expressed
SPIN1	-0.49	1.75E-03	4.23E-03	Under-expressed
RASSF5	-0.49	2.92E-03	6.64E-03	Under-expressed
ZNF154	-0.49	6.11E-03	0.0127	Under-expressed

FAM43A	-0.49	6.46E-03	0.0133	Under-expressed
GORAB	-0.49	8.72E-03	0.0173	Under-expressed
MIS18BP1	-0.49	0.01	0.0196	Under-expressed
TBC1D4	-0.49	0.0105	0.0204	Under-expressed
SH3RF1	-0.49	0.0114	0.0218	Under-expressed
RADX	-0.49	0.0169	0.0309	Under-expressed
PDE12	-0.5	1.24E-09	2.06E-08	Under-expressed
TMEM33	-0.5	3.20E-09	4.64E-08	Under-expressed
SPAG9	-0.5	1.11E-08	1.34E-07	Under-expressed
GOLGA5	-0.5	3.32E-08	3.48E-07	Under-expressed
KIF1B	-0.5	8.77E-07	5.71E-06	Under-expressed
FAM199X	-0.5	8.82E-07	5.74E-06	Under-expressed
TEX2	-0.5	9.98E-07	6.37E-06	Under-expressed
ZHX3	-0.5	1.74E-06	1.03E-05	Under-expressed
MACF1	-0.5	2.08E-06	1.19E-05	Under-expressed
CLASP2	-0.5	6.66E-06	3.29E-05	Under-expressed
PEX3	-0.5	1.18E-05	5.43E-05	Under-expressed
GGCX	-0.5	1.22E-05	5.59E-05	Under-expressed
EPM2AIP1	-0.5	1.97E-05	8.47E-05	Under-expressed
PRKACA	-0.5	3.56E-05	1.42E-04	Under-expressed
FAM76B	-0.5	6.94E-05	2.54E-04	Under-expressed
PLVAP	-0.5	9.21E-05	3.26E-04	Under-expressed
AHNAK	-0.5	9.51E-05	3.35E-04	Under-expressed
ZNF627	-0.5	1.00E-04	3.50E-04	Under-expressed
MEF2C	-0.5	1.19E-04	4.10E-04	Under-expressed
PDK2	-0.5	1.27E-04	4.34E-04	Under-expressed
EFR3A	-0.5	1.27E-04	4.34E-04	Under-expressed
PEAK1	-0.5	1.37E-04	4.61E-04	Under-expressed
NEMF	-0.5	1.48E-04	4.93E-04	Under-expressed
CMTM6	-0.5	2.02E-04	6.48E-04	Under-expressed
UBA7	-0.5	2.44E-04	7.64E-04	Under-expressed
ATP2B4	-0.5	2.52E-04	7.85E-04	Under-expressed
ZBTB1	-0.5	2.59E-04	8.05E-04	Under-expressed
GAB1	-0.5	2.81E-04	8.63E-04	Under-expressed
MED13L	-0.5	4.70E-04	1.35E-03	Under-expressed
RAB27A	-0.5	5.05E-04	1.44E-03	Under-expressed
ZBTB39	-0.5	8.35E-04	2.23E-03	Under-expressed
COQ10A	-0.5	9.27E-04	2.44E-03	Under-expressed
BAZ1A	-0.5	1.03E-03	2.67E-03	Under-expressed
DUSP10	-0.5	1.03E-03	2.67E-03	Under-expressed
MAPRE2	-0.5	1.08E-03	2.78E-03	Under-expressed
ZNF440	-0.5	1.10E-03	2.82E-03	Under-expressed
ZNF354B	-0.5	1.31E-03	3.29E-03	Under-expressed
ZNF595	-0.5	1.42E-03	3.52E-03	Under-expressed
TYW1B	-0.5	2.78E-03	6.38E-03	Under-expressed
RASAL2	-0.5	4.57E-03	9.84E-03	Under-expressed
RGPD4	-0.5	4.95E-03	0.0105	Under-expressed
CDK11A	-0.5	5.70E-03	0.0119	Under-expressed
DNAJB4	-0.5	6.76E-03	0.0139	Under-expressed
GALC	-0.5	9.66E-03	0.0189	Under-expressed
TOX2	-0.5	9.75E-03	0.0191	Under-expressed
APP	-0.5	0.0113	0.0218	Under-expressed
ANKRD24	-0.5	0.0128	0.0242	Under-expressed
FSTL1	-0.5	0.0146	0.0271	Under-expressed
SLC16A1	-0.5	0.0166	0.0303	Under-expressed
GPIHBP1	-0.5	0.0176	0.032	Under-expressed
PSAT1	-0.5	0.024	0.0422	Under-expressed

ATP6V1A	-0.51	4.19E-10	8.29E-09	Under-expressed
CREBBP	-0.51	1.19E-08	1.43E-07	Under-expressed
PPM1A	-0.51	1.68E-08	1.93E-07	Under-expressed
SENP2	-0.51	4.75E-08	4.70E-07	Under-expressed
RNF11	-0.51	2.30E-07	1.81E-06	Under-expressed
SNIP1	-0.51	2.76E-07	2.12E-06	Under-expressed
WASHC4	-0.51	2.89E-07	2.21E-06	Under-expressed
SOCS4	-0.51	5.94E-07	4.10E-06	Under-expressed
UPRT	-0.51	6.94E-07	4.68E-06	Under-expressed
SLMAP	-0.51	1.20E-06	7.48E-06	Under-expressed
KDM4A	-0.51	1.22E-06	7.58E-06	Under-expressed
PDIK1L	-0.51	1.99E-06	1.15E-05	Under-expressed
KIAA0586	-0.51	4.00E-06	2.12E-05	Under-expressed
PARP14	-0.51	5.60E-06	2.83E-05	Under-expressed
PHC3	-0.51	6.08E-06	3.04E-05	Under-expressed
NCOA4	-0.51	1.13E-05	5.23E-05	Under-expressed
CLINT1	-0.51	1.42E-05	6.38E-05	Under-expressed
HECTD4	-0.51	2.40E-05	1.01E-04	Under-expressed
PIBF1	-0.51	3.61E-05	1.44E-04	Under-expressed
FARP2	-0.51	3.82E-05	1.51E-04	Under-expressed
ATAD2B	-0.51	4.87E-05	1.87E-04	Under-expressed
CSGALNACT2	-0.51	5.07E-05	1.93E-04	Under-expressed
PANK3	-0.51	5.59E-05	2.11E-04	Under-expressed
DICER1	-0.51	5.86E-05	2.20E-04	Under-expressed
ETFRF1	-0.51	9.62E-05	3.39E-04	Under-expressed
SHTN1	-0.51	9.90E-05	3.47E-04	Under-expressed
RHOBTB2	-0.51	2.57E-04	7.98E-04	Under-expressed
FBXO30	-0.51	3.57E-04	1.07E-03	Under-expressed
BPHL	-0.51	4.04E-04	1.19E-03	Under-expressed
UNC13B	-0.51	4.69E-04	1.35E-03	Under-expressed
ZBTB41	-0.51	4.84E-04	1.39E-03	Under-expressed
TADA2A	-0.51	6.91E-04	1.89E-03	Under-expressed
NKTR	-0.51	7.22E-04	1.96E-03	Under-expressed
BDH2	-0.51	7.55E-04	2.04E-03	Under-expressed
SPRY2	-0.51	9.67E-04	2.53E-03	Under-expressed
PIP5K1A	-0.51	1.01E-03	2.62E-03	Under-expressed
SFXN5	-0.51	1.22E-03	3.10E-03	Under-expressed
DPYSL2	-0.51	1.37E-03	3.42E-03	Under-expressed
ZNF836	-0.51	2.41E-03	5.63E-03	Under-expressed
P2RY8	-0.51	2.83E-03	6.48E-03	Under-expressed
SAMD9	-0.51	3.26E-03	7.32E-03	Under-expressed
L3MBTL4	-0.51	3.68E-03	8.11E-03	Under-expressed
ATF7IP2	-0.51	4.25E-03	9.23E-03	Under-expressed
IRS2	-0.51	4.46E-03	9.63E-03	Under-expressed
RARB	-0.51	4.59E-03	9.88E-03	Under-expressed
GJA4	-0.51	4.70E-03	0.0101	Under-expressed
PBX3	-0.51	4.94E-03	0.0105	Under-expressed
TCP11L2	-0.51	6.29E-03	0.013	Under-expressed
STXBP1	-0.51	7.71E-03	0.0156	Under-expressed
ACE	-0.51	9.26E-03	0.0183	Under-expressed
IFI16	-0.51	0.0117	0.0223	Under-expressed
GNG7	-0.51	0.0165	0.0303	Under-expressed
PTPRC	-0.51	0.0289	0.0497	Under-expressed
STX17	-0.52	3.07E-10	6.30E-09	Under-expressed
ZFYVE1	-0.52	1.36E-08	1.61E-07	Under-expressed
TRMT1L	-0.52	3.08E-08	3.26E-07	Under-expressed
CDC42BPB	-0.52	4.23E-08	4.27E-07	Under-expressed

PCNX1	-0.52	5.33E-08	5.22E-07	Under-expressed
SRP54	-0.52	1.08E-07	9.50E-07	Under-expressed
DCLRE1A	-0.52	3.32E-07	2.48E-06	Under-expressed
MFSD1	-0.52	4.32E-07	3.11E-06	Under-expressed
MIER1	-0.52	5.89E-07	4.07E-06	Under-expressed
SSH1	-0.52	1.75E-06	1.03E-05	Under-expressed
ANKRD12	-0.52	7.83E-06	3.79E-05	Under-expressed
UBR2	-0.52	9.81E-06	4.62E-05	Under-expressed
GOT2	-0.52	1.15E-05	5.32E-05	Under-expressed
AKAP11	-0.52	1.32E-05	5.99E-05	Under-expressed
FAM210B	-0.52	1.63E-05	7.17E-05	Under-expressed
PCCA	-0.52	2.90E-05	1.19E-04	Under-expressed
TADA1	-0.52	3.56E-05	1.42E-04	Under-expressed
GAB2	-0.52	4.16E-05	1.63E-04	Under-expressed
SS18L1	-0.52	4.27E-05	1.67E-04	Under-expressed
VAMP1	-0.52	5.99E-05	2.23E-04	Under-expressed
CRAT	-0.52	6.28E-05	2.33E-04	Under-expressed
AFF1	-0.52	7.04E-05	2.57E-04	Under-expressed
ZNF510	-0.52	7.76E-05	2.81E-04	Under-expressed
NPAT	-0.52	8.07E-05	2.91E-04	Under-expressed
PLEKHM1P1	-0.52	8.76E-05	3.12E-04	Under-expressed
SP3	-0.52	8.99E-05	3.19E-04	Under-expressed
TMEM161B	-0.52	1.04E-04	3.64E-04	Under-expressed
CCDC121	-0.52	1.62E-04	5.34E-04	Under-expressed
FANCM	-0.52	2.44E-04	7.64E-04	Under-expressed
TNS2	-0.52	3.95E-04	1.16E-03	Under-expressed
USHBP1	-0.52	4.36E-04	1.27E-03	Under-expressed
MZF1-AS1	-0.52	4.72E-04	1.36E-03	Under-expressed
ALG10B	-0.52	5.71E-04	1.60E-03	Under-expressed
TSPYL4	-0.52	8.10E-04	2.17E-03	Under-expressed
PRKCH	-0.52	8.11E-04	2.17E-03	Under-expressed
MARS2	-0.52	1.42E-03	3.52E-03	Under-expressed
CLEC14A	-0.52	1.52E-03	3.74E-03	Under-expressed
BCOR	-0.52	1.60E-03	3.91E-03	Under-expressed
SLC20A2	-0.52	1.78E-03	4.30E-03	Under-expressed
C1ORF226	-0.52	2.30E-03	5.39E-03	Under-expressed
LRRC32	-0.52	2.42E-03	5.64E-03	Under-expressed
PDGFRB	-0.52	2.96E-03	6.73E-03	Under-expressed
MYO5C	-0.52	3.35E-03	7.49E-03	Under-expressed
ANKRD26	-0.52	3.45E-03	7.69E-03	Under-expressed
ALPK1	-0.52	5.03E-03	0.0107	Under-expressed
PCNX4	-0.52	0.0103	0.02	Under-expressed
PXMP2	-0.52	0.0103	0.02	Under-expressed
TSHZ3	-0.52	0.011	0.0211	Under-expressed
GATA6	-0.52	0.0146	0.0272	Under-expressed
DGKE	-0.52	0.0149	0.0277	Under-expressed
LPCAT2	-0.52	0.0152	0.0281	Under-expressed
LURAP1L	-0.52	0.0171	0.0311	Under-expressed
SRBD1	-0.53	8.56E-09	1.07E-07	Under-expressed
UVRAG	-0.53	3.36E-08	3.51E-07	Under-expressed
ANKH	-0.53	2.88E-07	2.20E-06	Under-expressed
NFE2L1	-0.53	3.71E-07	2.73E-06	Under-expressed
SPPL2A	-0.53	4.61E-07	3.29E-06	Under-expressed
APLP2	-0.53	7.32E-07	4.90E-06	Under-expressed
EVI5	-0.53	7.98E-07	5.25E-06	Under-expressed
SETD2	-0.53	8.07E-07	5.31E-06	Under-expressed
SP2	-0.53	1.35E-06	8.25E-06	Under-expressed

ABCD3	-0.53	1.52E-06	9.09E-06	Under-expressed
DLG1	-0.53	1.85E-06	1.08E-05	Under-expressed
GABPA	-0.53	2.23E-06	1.27E-05	Under-expressed
C5ORF51	-0.53	2.28E-06	1.29E-05	Under-expressed
IL13RA1	-0.53	3.45E-06	1.85E-05	Under-expressed
ATF7IP	-0.53	3.72E-06	1.98E-05	Under-expressed
TCEANC2	-0.53	4.57E-06	2.37E-05	Under-expressed
HDAC6	-0.53	1.55E-05	6.87E-05	Under-expressed
TNFAIP8L1	-0.53	2.51E-05	1.05E-04	Under-expressed
DNAJB9	-0.53	5.04E-05	1.92E-04	Under-expressed
ZMYM1	-0.53	5.62E-05	2.12E-04	Under-expressed
DDX59	-0.53	6.13E-05	2.28E-04	Under-expressed
RAB11FIP2	-0.53	6.69E-05	2.46E-04	Under-expressed
ENTPD7	-0.53	7.96E-05	2.87E-04	Under-expressed
ADGRL4	-0.53	1.38E-04	4.64E-04	Under-expressed
PINK1	-0.53	1.69E-04	5.55E-04	Under-expressed
DENND5B	-0.53	2.32E-04	7.31E-04	Under-expressed
SENP7	-0.53	2.36E-04	7.41E-04	Under-expressed
GALNT11	-0.53	2.44E-04	7.63E-04	Under-expressed
C1ORF109	-0.53	2.69E-04	8.30E-04	Under-expressed
EPS8	-0.53	3.78E-04	1.12E-03	Under-expressed
PMS2P2	-0.53	4.13E-04	1.21E-03	Under-expressed
LARGE1	-0.53	5.41E-04	1.52E-03	Under-expressed
DLC1	-0.53	6.95E-04	1.90E-03	Under-expressed
ATP8B1	-0.53	7.58E-04	2.05E-03	Under-expressed
ZFPM1	-0.53	8.33E-04	2.22E-03	Under-expressed
PORCN	-0.53	1.04E-03	2.69E-03	Under-expressed
ANXA1	-0.53	1.15E-03	2.93E-03	Under-expressed
RGPD1	-0.53	2.11E-03	5.01E-03	Under-expressed
RAPGEF5	-0.53	2.32E-03	5.44E-03	Under-expressed
ATP8A1	-0.53	3.15E-03	7.11E-03	Under-expressed
ASAP3	-0.53	3.48E-03	7.74E-03	Under-expressed
LRRC4	-0.53	7.53E-03	0.0152	Under-expressed
CLU	-0.53	9.14E-03	0.0181	Under-expressed
GABARAPL1	-0.53	9.21E-03	0.0182	Under-expressed
PDE4B	-0.53	0.0113	0.0218	Under-expressed
ONECUT2	-0.53	0.0179	0.0325	Under-expressed
PPP1R3C	-0.53	0.0232	0.0409	Under-expressed
PTCH1	-0.53	0.0286	0.0493	Under-expressed
SLAIN2	-0.54	2.55E-10	5.42E-09	Under-expressed
FEM1B	-0.54	1.50E-08	1.75E-07	Under-expressed
ACAD8	-0.54	2.47E-08	2.69E-07	Under-expressed
ATG4A	-0.54	3.10E-08	3.27E-07	Under-expressed
MCFD2	-0.54	4.09E-08	4.17E-07	Under-expressed
GFOD2	-0.54	6.90E-08	6.48E-07	Under-expressed
DIS3L	-0.54	2.56E-07	1.98E-06	Under-expressed
VAMP4	-0.54	2.58E-07	2.00E-06	Under-expressed
SLC35B4	-0.54	3.87E-07	2.82E-06	Under-expressed
MPPE1	-0.54	6.39E-07	4.37E-06	Under-expressed
DENND4C	-0.54	8.42E-07	5.50E-06	Under-expressed
ZMYND11	-0.54	1.14E-06	7.12E-06	Under-expressed
JAK1	-0.54	1.22E-06	7.53E-06	Under-expressed
NF1	-0.54	1.48E-06	8.87E-06	Under-expressed
TMEM106B	-0.54	1.68E-06	9.94E-06	Under-expressed
TBC1D23	-0.54	2.27E-06	1.28E-05	Under-expressed
SPTBN1	-0.54	2.57E-06	1.43E-05	Under-expressed
KAT6B	-0.54	3.84E-06	2.04E-05	Under-expressed

USP16	-0.54	4.05E-06	2.14E-05	Under-expressed
ABCB10	-0.54	4.86E-06	2.50E-05	Under-expressed
ZC3H7B	-0.54	4.91E-06	2.53E-05	Under-expressed
AKAP13	-0.54	4.96E-06	2.55E-05	Under-expressed
RALBP1	-0.54	7.56E-06	3.68E-05	Under-expressed
WDR11	-0.54	1.57E-05	6.95E-05	Under-expressed
SLC31A2	-0.54	2.93E-05	1.20E-04	Under-expressed
CD46	-0.54	3.74E-05	1.48E-04	Under-expressed
MFSD9	-0.54	6.11E-05	2.27E-04	Under-expressed
MAP3K20	-0.54	9.47E-05	3.34E-04	Under-expressed
TET2	-0.54	1.21E-04	4.16E-04	Under-expressed
C21ORF91	-0.54	1.61E-04	5.31E-04	Under-expressed
USP6NL	-0.54	1.97E-04	6.36E-04	Under-expressed
FAM8A1	-0.54	2.35E-04	7.39E-04	Under-expressed
LMO2	-0.54	4.00E-04	1.17E-03	Under-expressed
CCP110	-0.54	4.63E-04	1.33E-03	Under-expressed
C4ORF33	-0.54	5.60E-04	1.57E-03	Under-expressed
IPPK	-0.54	5.75E-04	1.61E-03	Under-expressed
PLEKHA6	-0.54	5.87E-04	1.63E-03	Under-expressed
PER1	-0.54	9.45E-04	2.48E-03	Under-expressed
ALDH4A1	-0.54	1.27E-03	3.20E-03	Under-expressed
ZFX	-0.54	1.83E-03	4.40E-03	Under-expressed
ZNF493	-0.54	2.03E-03	4.82E-03	Under-expressed
PCDH1	-0.54	2.28E-03	5.35E-03	Under-expressed
SCHIP1	-0.54	2.90E-03	6.61E-03	Under-expressed
ELMO1	-0.54	4.92E-03	0.0105	Under-expressed
CD40	-0.54	5.65E-03	0.0118	Under-expressed
CIDEB	-0.54	6.89E-03	0.0141	Under-expressed
ADGRA2	-0.54	7.20E-03	0.0146	Under-expressed
DDIT4	-0.54	0.0121	0.023	Under-expressed
PC	-0.54	0.0242	0.0424	Under-expressed
SRPRA	-0.55	2.83E-10	5.89E-09	Under-expressed
VPS13D	-0.55	4.31E-10	8.49E-09	Under-expressed
PRORP	-0.55	2.06E-09	3.15E-08	Under-expressed
MIA2	-0.55	3.89E-09	5.49E-08	Under-expressed
GINM1	-0.55	8.72E-09	1.09E-07	Under-expressed
GLYR1	-0.55	1.97E-08	2.21E-07	Under-expressed
SNX18	-0.55	3.70E-08	3.82E-07	Under-expressed
USP28	-0.55	2.34E-07	1.84E-06	Under-expressed
SMC6	-0.55	2.52E-07	1.96E-06	Under-expressed
PPP1R15B	-0.55	3.29E-07	2.47E-06	Under-expressed
C5ORF24	-0.55	4.67E-07	3.32E-06	Under-expressed
THNSL1	-0.55	6.30E-07	4.31E-06	Under-expressed
CTR9	-0.55	6.89E-07	4.66E-06	Under-expressed
FECH	-0.55	1.23E-06	7.62E-06	Under-expressed
CCDC25	-0.55	1.24E-06	7.64E-06	Under-expressed
SNX9	-0.55	1.29E-06	7.93E-06	Under-expressed
LRP6	-0.55	3.20E-06	1.73E-05	Under-expressed
LDLR	-0.55	9.39E-06	4.45E-05	Under-expressed
TM7SF3	-0.55	1.73E-05	7.56E-05	Under-expressed
GANC	-0.55	1.78E-05	7.75E-05	Under-expressed
KHNYN	-0.55	1.81E-05	7.87E-05	Under-expressed
PHF6	-0.55	2.34E-05	9.83E-05	Under-expressed
CD93	-0.55	2.87E-05	1.18E-04	Under-expressed
RNF38	-0.55	3.16E-05	1.28E-04	Under-expressed
HEATR5B	-0.55	3.69E-05	1.47E-04	Under-expressed
TP53BP2	-0.55	4.47E-05	1.73E-04	Under-expressed

GABPB1-AS1	-0.55	5.71E-05	2.14E-04	Under-expressed
PARP3	-0.55	6.23E-05	2.31E-04	Under-expressed
ABCA1	-0.55	3.78E-04	1.12E-03	Under-expressed
VPS13A	-0.55	7.05E-04	1.92E-03	Under-expressed
MYO5B	-0.55	8.60E-04	2.29E-03	Under-expressed
SMARCAD1	-0.55	9.61E-04	2.52E-03	Under-expressed
FAM241A	-0.55	1.29E-03	3.24E-03	Under-expressed
CD200	-0.55	1.62E-03	3.96E-03	Under-expressed
SIMC1	-0.55	2.21E-03	5.22E-03	Under-expressed
ZDHHC23	-0.55	2.25E-03	5.30E-03	Under-expressed
CBR1	-0.55	2.73E-03	6.27E-03	Under-expressed
GCH1	-0.55	3.27E-03	7.34E-03	Under-expressed
CEP152	-0.55	5.01E-03	0.0106	Under-expressed
HIF1A	-0.55	6.21E-03	0.0129	Under-expressed
GLYCTK	-0.55	8.05E-03	0.0162	Under-expressed
PXDN	-0.55	8.24E-03	0.0165	Under-expressed
PRKN	-0.55	0.0119	0.0226	Under-expressed
PTPRM	-0.55	0.0132	0.0249	Under-expressed
SUN2	-0.56	8.21E-09	1.03E-07	Under-expressed
HERC2	-0.56	1.98E-08	2.22E-07	Under-expressed
EXOC5	-0.56	2.32E-08	2.54E-07	Under-expressed
NAGA	-0.56	7.20E-07	4.83E-06	Under-expressed
TRIM38	-0.56	8.86E-07	5.75E-06	Under-expressed
ZC3H6	-0.56	1.46E-06	8.80E-06	Under-expressed
MINDY3	-0.56	3.08E-06	1.68E-05	Under-expressed
FERMT2	-0.56	3.53E-06	1.89E-05	Under-expressed
PTPN11	-0.56	5.88E-06	2.96E-05	Under-expressed
SHANK3	-0.56	1.69E-05	7.40E-05	Under-expressed
ZCCHC24	-0.56	1.82E-05	7.91E-05	Under-expressed
GTF2IRD2B	-0.56	1.93E-05	8.31E-05	Under-expressed
PPP2R5E	-0.56	2.17E-05	9.22E-05	Under-expressed
PLS3	-0.56	3.45E-05	1.39E-04	Under-expressed
C11ORF54	-0.56	3.91E-05	1.54E-04	Under-expressed
BBS9	-0.56	4.02E-05	1.58E-04	Under-expressed
HELZ2	-0.56	4.59E-05	1.77E-04	Under-expressed
MAP3K7CL	-0.56	4.74E-05	1.82E-04	Under-expressed
ARHGEF11	-0.56	6.38E-05	2.36E-04	Under-expressed
PIK3C2B	-0.56	9.35E-05	3.31E-04	Under-expressed
MTUS1	-0.56	1.49E-04	4.97E-04	Under-expressed
WRN	-0.56	4.48E-04	1.30E-03	Under-expressed
DOP1B	-0.56	5.80E-04	1.62E-03	Under-expressed
EGFLAM	-0.56	7.99E-04	2.15E-03	Under-expressed
TKFC	-0.56	1.05E-03	2.71E-03	Under-expressed
LNP1	-0.56	1.42E-03	3.53E-03	Under-expressed
CALHM5	-0.56	1.62E-03	3.95E-03	Under-expressed
NUDT7	-0.56	1.86E-03	4.45E-03	Under-expressed
PPP1R16B	-0.56	2.10E-03	4.99E-03	Under-expressed
TIAM1	-0.56	2.40E-03	5.60E-03	Under-expressed
NUDT6	-0.56	2.58E-03	5.97E-03	Under-expressed
STARD4	-0.56	2.69E-03	6.19E-03	Under-expressed
CARD6	-0.56	3.21E-03	7.23E-03	Under-expressed
ARHGAP10	-0.56	4.47E-03	9.65E-03	Under-expressed
MSRB3	-0.56	0.0107	0.0207	Under-expressed
PROSER3	-0.56	0.0114	0.0218	Under-expressed
CBS	-0.56	0.0156	0.0288	Under-expressed
GPAT3	-0.56	0.0191	0.0345	Under-expressed
MYO7A	-0.56	0.0225	0.0398	Under-expressed

FYTTD1	-0.57	7.67E-11	1.98E-09	Under-expressed
DAG1	-0.57	8.38E-10	1.48E-08	Under-expressed
ZNF654	-0.57	8.99E-10	1.56E-08	Under-expressed
SEC22B	-0.57	2.62E-09	3.89E-08	Under-expressed
SLC33A1	-0.57	4.55E-09	6.25E-08	Under-expressed
MGAT2	-0.57	1.20E-08	1.43E-07	Under-expressed
RRN3	-0.57	1.58E-08	1.83E-07	Under-expressed
SNX13	-0.57	2.24E-08	2.48E-07	Under-expressed
NCOA1	-0.57	3.25E-08	3.43E-07	Under-expressed
UTP14C	-0.57	3.84E-08	3.95E-07	Under-expressed
MICAL3	-0.57	4.24E-08	4.28E-07	Under-expressed
CLPX	-0.57	4.36E-08	4.37E-07	Under-expressed
SLC9A6	-0.57	5.35E-08	5.24E-07	Under-expressed
MPHOSPH8	-0.57	2.14E-07	1.71E-06	Under-expressed
FUCA1	-0.57	2.15E-07	1.72E-06	Under-expressed
ZFP1	-0.57	2.27E-07	1.80E-06	Under-expressed
ADIPOR2	-0.57	3.31E-07	2.48E-06	Under-expressed
UBE3C	-0.57	6.67E-07	4.54E-06	Under-expressed
KLF11	-0.57	7.92E-07	5.23E-06	Under-expressed
UBXN2B	-0.57	9.48E-07	6.09E-06	Under-expressed
KIAA1147	-0.57	4.48E-06	2.34E-05	Under-expressed
SLC12A6	-0.57	6.48E-06	3.22E-05	Under-expressed
ZNF436	-0.57	8.74E-06	4.17E-05	Under-expressed
HSD17B12	-0.57	1.07E-05	4.99E-05	Under-expressed
PHLPP2	-0.57	1.58E-05	6.99E-05	Under-expressed
KIF16B	-0.57	1.74E-05	7.58E-05	Under-expressed
EIF2AK3	-0.57	2.57E-05	1.07E-04	Under-expressed
RLF	-0.57	3.62E-05	1.44E-04	Under-expressed
RPL23AP53	-0.57	4.28E-05	1.67E-04	Under-expressed
TRUB1	-0.57	4.39E-05	1.70E-04	Under-expressed
ICK	-0.57	8.50E-05	3.04E-04	Under-expressed
BBX	-0.57	1.36E-04	4.60E-04	Under-expressed
TNS1	-0.57	1.59E-04	5.25E-04	Under-expressed
APLF	-0.57	2.04E-04	6.55E-04	Under-expressed
BTN3A1	-0.57	2.63E-04	8.14E-04	Under-expressed
GSR	-0.57	3.18E-04	9.65E-04	Under-expressed
HTR7P1	-0.57	3.22E-04	9.73E-04	Under-expressed
HGSNAT	-0.57	4.55E-04	1.32E-03	Under-expressed
DENND1B	-0.57	4.94E-04	1.41E-03	Under-expressed
AHR	-0.57	7.46E-04	2.02E-03	Under-expressed
BTAF1	-0.57	7.66E-04	2.07E-03	Under-expressed
KIF17	-0.57	8.11E-04	2.17E-03	Under-expressed
FURIN	-0.57	9.46E-04	2.48E-03	Under-expressed
HAGH	-0.57	1.26E-03	3.17E-03	Under-expressed
PTPRN2	-0.57	1.96E-03	4.67E-03	Under-expressed
OSBPL1A	-0.57	2.73E-03	6.28E-03	Under-expressed
TTC39C	-0.57	2.88E-03	6.57E-03	Under-expressed
CDH2	-0.57	3.53E-03	7.83E-03	Under-expressed
NR5A2	-0.57	3.58E-03	7.93E-03	Under-expressed
GPR153	-0.57	3.70E-03	8.14E-03	Under-expressed
TNXB	-0.57	6.14E-03	0.0127	Under-expressed
EIF4E3	-0.57	6.24E-03	0.0129	Under-expressed
SLC26A1	-0.57	7.70E-03	0.0155	Under-expressed
KRBA2	-0.57	0.0104	0.0203	Under-expressed
FITM1	-0.57	0.015	0.0278	Under-expressed
ECHDC3	-0.57	0.0275	0.0476	Under-expressed
MYOF	-0.57	0.0282	0.0487	Under-expressed

ERMAP	-0.58	1.02E-08	1.25E-07	Under-expressed
CYLD	-0.58	2.01E-08	2.25E-07	Under-expressed
OGDH	-0.58	1.53E-07	1.30E-06	Under-expressed
TNRC6B	-0.58	1.81E-07	1.49E-06	Under-expressed
TRIM5	-0.58	2.71E-07	2.09E-06	Under-expressed
SPEN	-0.58	6.55E-07	4.47E-06	Under-expressed
ATP2A2	-0.58	7.86E-07	5.19E-06	Under-expressed
ZFP36L1	-0.58	8.98E-07	5.81E-06	Under-expressed
KCTD3	-0.58	1.19E-06	7.41E-06	Under-expressed
SH2D3C	-0.58	1.66E-06	9.85E-06	Under-expressed
DNAJB14	-0.58	1.91E-06	1.11E-05	Under-expressed
MRTFB	-0.58	4.07E-06	2.15E-05	Under-expressed
ARL6IP1	-0.58	6.03E-06	3.02E-05	Under-expressed
MMAA	-0.58	7.62E-06	3.71E-05	Under-expressed
CBX7	-0.58	8.99E-06	4.28E-05	Under-expressed
LMAN1	-0.58	1.35E-05	6.09E-05	Under-expressed
SWAP70	-0.58	1.50E-05	6.66E-05	Under-expressed
MAPRE3	-0.58	2.18E-05	9.26E-05	Under-expressed
STXBP3	-0.58	2.57E-05	1.07E-04	Under-expressed
CLDN23	-0.58	1.20E-04	4.11E-04	Under-expressed
PAPOLG	-0.58	1.40E-04	4.70E-04	Under-expressed
LGR4	-0.58	1.57E-04	5.20E-04	Under-expressed
MOCS1	-0.58	1.64E-04	5.38E-04	Under-expressed
AP1AR	-0.58	2.17E-04	6.89E-04	Under-expressed
JAZF1	-0.58	3.28E-04	9.88E-04	Under-expressed
RNF128	-0.58	3.66E-04	1.09E-03	Under-expressed
TESK2	-0.58	4.93E-04	1.41E-03	Under-expressed
SERPING1	-0.58	7.80E-04	2.10E-03	Under-expressed
ACBD4	-0.58	1.01E-03	2.62E-03	Under-expressed
RNLS	-0.58	1.04E-03	2.69E-03	Under-expressed
SLC35C1	-0.58	1.10E-03	2.81E-03	Under-expressed
C1RL-AS1	-0.58	1.24E-03	3.13E-03	Under-expressed
LOC100133331	-0.58	2.99E-03	6.79E-03	Under-expressed
NAV2	-0.58	7.94E-03	0.016	Under-expressed
CSNK2A3	-0.58	8.12E-03	0.0163	Under-expressed
TMEM176B	-0.58	0.0128	0.0242	Under-expressed
USP32	-0.59	2.73E-09	4.05E-08	Under-expressed
LNX2	-0.59	1.89E-08	2.15E-07	Under-expressed
ABHD17B	-0.59	2.49E-08	2.71E-07	Under-expressed
SCARF1	-0.59	7.35E-08	6.83E-07	Under-expressed
RNF6	-0.59	8.59E-08	7.85E-07	Under-expressed
TOGARAM1	-0.59	1.02E-06	6.49E-06	Under-expressed
SUCLG2	-0.59	1.89E-06	1.10E-05	Under-expressed
PDLIM5	-0.59	2.44E-06	1.37E-05	Under-expressed
UFL1	-0.59	2.95E-06	1.62E-05	Under-expressed
SMC1A	-0.59	3.18E-06	1.72E-05	Under-expressed
PPIP5K2	-0.59	3.31E-06	1.78E-05	Under-expressed
ANKRD17	-0.59	4.40E-06	2.30E-05	Under-expressed
USP25	-0.59	1.43E-05	6.42E-05	Under-expressed
CNN3	-0.59	1.44E-05	6.42E-05	Under-expressed
CLCN4	-0.59	1.49E-05	6.63E-05	Under-expressed
ITPRIP	-0.59	2.20E-05	9.31E-05	Under-expressed
ANKRA2	-0.59	2.59E-05	1.08E-04	Under-expressed
FOXN2	-0.59	2.73E-05	1.13E-04	Under-expressed
ATXN7L1	-0.59	4.18E-05	1.63E-04	Under-expressed
STARD13	-0.59	6.22E-05	2.31E-04	Under-expressed
TMEM255B	-0.59	1.29E-04	4.39E-04	Under-expressed

PYGL	-0.59	1.57E-04	5.20E-04	Under-expressed
ZEB2	-0.59	2.16E-04	6.88E-04	Under-expressed
ECHDC2	-0.59	2.23E-04	7.06E-04	Under-expressed
SORBS1	-0.59	2.36E-04	7.40E-04	Under-expressed
NOTCH2	-0.59	3.43E-04	1.03E-03	Under-expressed
PPP2R1B	-0.59	4.17E-04	1.22E-03	Under-expressed
CTSF	-0.59	5.37E-04	1.52E-03	Under-expressed
RCL1	-0.59	7.43E-04	2.01E-03	Under-expressed
TSPAN18	-0.59	8.75E-04	2.32E-03	Under-expressed
TWSG1	-0.59	8.90E-04	2.35E-03	Under-expressed
SCCPDH	-0.59	9.27E-04	2.44E-03	Under-expressed
TBC1D19	-0.59	9.67E-04	2.53E-03	Under-expressed
ZNF596	-0.59	9.94E-04	2.59E-03	Under-expressed
ANKEF1	-0.59	1.14E-03	2.91E-03	Under-expressed
ARSG	-0.59	2.71E-03	6.24E-03	Under-expressed
TTN	-0.59	5.28E-03	0.0111	Under-expressed
C1ORF21	-0.59	0.0113	0.0217	Under-expressed
LILRB5	-0.59	0.0116	0.0222	Under-expressed
GLDC	-0.59	0.0208	0.0371	Under-expressed
ETV1	-0.59	0.0268	0.0466	Under-expressed
NPR2	-0.59	0.0285	0.0491	Under-expressed
OPA1	-0.6	3.96E-13	2.06E-11	Under-expressed
PDXDC1	-0.6	5.47E-10	1.03E-08	Under-expressed
TNRC6A	-0.6	1.13E-09	1.90E-08	Under-expressed
NR1D2	-0.6	6.60E-09	8.63E-08	Under-expressed
IDH1	-0.6	2.93E-08	3.12E-07	Under-expressed
LARS2	-0.6	3.39E-08	3.54E-07	Under-expressed
FBXL3	-0.6	5.76E-08	5.53E-07	Under-expressed
UCHL5	-0.6	6.49E-08	6.13E-07	Under-expressed
SNX4	-0.6	9.70E-08	8.71E-07	Under-expressed
TGOLN2	-0.6	1.48E-07	1.26E-06	Under-expressed
ADCY9	-0.6	2.17E-07	1.73E-06	Under-expressed
DCBLD1	-0.6	2.22E-07	1.77E-06	Under-expressed
ISOC1	-0.6	9.24E-07	5.96E-06	Under-expressed
RC3H2	-0.6	1.42E-06	8.56E-06	Under-expressed
ZNF25	-0.6	1.61E-06	9.61E-06	Under-expressed
FCHO2	-0.6	1.62E-06	9.67E-06	Under-expressed
PATL1	-0.6	1.95E-06	1.13E-05	Under-expressed
ZNF397	-0.6	2.05E-06	1.18E-05	Under-expressed
ARID4A	-0.6	2.97E-06	1.63E-05	Under-expressed
OXR1	-0.6	1.13E-05	5.22E-05	Under-expressed
WIPF2	-0.6	1.47E-05	6.54E-05	Under-expressed
SLC35F5	-0.6	1.60E-05	7.06E-05	Under-expressed
KLLN	-0.6	2.30E-05	9.70E-05	Under-expressed
ARHGEF15	-0.6	2.73E-05	1.13E-04	Under-expressed
ACOT2	-0.6	9.72E-05	3.42E-04	Under-expressed
TEAD1	-0.6	1.10E-04	3.80E-04	Under-expressed
HLX	-0.6	2.27E-04	7.16E-04	Under-expressed
FAM126A	-0.6	3.00E-04	9.14E-04	Under-expressed
PPM1K	-0.6	5.39E-04	1.52E-03	Under-expressed
FAM111B	-0.6	6.24E-04	1.72E-03	Under-expressed
IL7	-0.6	1.60E-03	3.91E-03	Under-expressed
TRMT11	-0.6	1.71E-03	4.16E-03	Under-expressed
CPLANE1	-0.6	2.20E-03	5.19E-03	Under-expressed
CHN1	-0.6	2.22E-03	5.23E-03	Under-expressed
FAM47E	-0.6	5.23E-03	0.011	Under-expressed
TRIB2	-0.6	5.53E-03	0.0116	Under-expressed

FAXDC2	-0.6	6.34E-03	0.0131	Under-expressed
KIF21B	-0.6	6.82E-03	0.014	Under-expressed
OCLN	-0.6	7.18E-03	0.0146	Under-expressed
PLAT	-0.6	0.0122	0.0231	Under-expressed
LTBP4	-0.6	0.0146	0.0272	Under-expressed
IFI44L	-0.6	0.0216	0.0383	Under-expressed
WDTC1	-0.61	9.67E-10	1.66E-08	Under-expressed
FAM122A	-0.61	1.65E-09	2.61E-08	Under-expressed
MCCC2	-0.61	3.98E-08	4.07E-07	Under-expressed
MTOR	-0.61	3.99E-08	4.08E-07	Under-expressed
ITFG1	-0.61	5.10E-07	3.59E-06	Under-expressed
BTD	-0.61	1.21E-06	7.48E-06	Under-expressed
CEP104	-0.61	1.35E-06	8.22E-06	Under-expressed
BMI1	-0.61	1.38E-06	8.37E-06	Under-expressed
KDM7A	-0.61	1.64E-06	9.76E-06	Under-expressed
CD99L2	-0.61	3.30E-06	1.78E-05	Under-expressed
AQR	-0.61	3.44E-06	1.85E-05	Under-expressed
HLTF	-0.61	1.34E-05	6.04E-05	Under-expressed
PGM1	-0.61	1.77E-05	7.71E-05	Under-expressed
CDKN2AIP	-0.61	2.68E-05	1.11E-04	Under-expressed
CCNT2	-0.61	4.15E-05	1.63E-04	Under-expressed
ETS2	-0.61	8.68E-05	3.10E-04	Under-expressed
ZNF268	-0.61	1.26E-04	4.30E-04	Under-expressed
EMBP1	-0.61	1.98E-04	6.38E-04	Under-expressed
NLN	-0.61	2.99E-04	9.12E-04	Under-expressed
FGD5	-0.61	3.07E-04	9.35E-04	Under-expressed
NHSL1	-0.61	3.17E-04	9.60E-04	Under-expressed
ZNF460	-0.61	4.73E-04	1.36E-03	Under-expressed
ABCA2	-0.61	6.09E-04	1.69E-03	Under-expressed
SLC8A1	-0.61	1.07E-03	2.75E-03	Under-expressed
CDC14A	-0.61	1.09E-03	2.79E-03	Under-expressed
ZNF136	-0.61	1.13E-03	2.88E-03	Under-expressed
ACOT4	-0.61	1.34E-03	3.36E-03	Under-expressed
STS	-0.61	1.37E-03	3.42E-03	Under-expressed
ZMAT3	-0.61	1.46E-03	3.61E-03	Under-expressed
SAMD9L	-0.61	1.64E-03	3.99E-03	Under-expressed
TNFSF10	-0.61	1.84E-03	4.42E-03	Under-expressed
HERC6	-0.61	2.28E-03	5.35E-03	Under-expressed
ADAM17	-0.61	3.53E-03	7.83E-03	Under-expressed
RCAN2	-0.61	5.58E-03	0.0117	Under-expressed
HSP90AB2P	-0.61	0.0118	0.0226	Under-expressed
EFCAB14	-0.62	4.10E-11	1.16E-09	Under-expressed
C2ORF69	-0.62	5.79E-10	1.08E-08	Under-expressed
MGRN1	-0.62	1.77E-09	2.77E-08	Under-expressed
UBR3	-0.62	1.86E-09	2.89E-08	Under-expressed
PEX19	-0.62	2.26E-09	3.43E-08	Under-expressed
ATG4C	-0.62	1.42E-08	1.66E-07	Under-expressed
ZNF800	-0.62	3.32E-08	3.48E-07	Under-expressed
CLIP1	-0.62	4.41E-08	4.41E-07	Under-expressed
ATF6	-0.62	6.05E-08	5.78E-07	Under-expressed
ITSN2	-0.62	9.46E-08	8.53E-07	Under-expressed
ZNRF2	-0.62	1.35E-07	1.17E-06	Under-expressed
PIGK	-0.62	1.50E-07	1.28E-06	Under-expressed
PKN2	-0.62	7.32E-07	4.90E-06	Under-expressed
TMED7	-0.62	1.05E-06	6.65E-06	Under-expressed
LRP5	-0.62	1.11E-06	6.96E-06	Under-expressed
HIGD1A	-0.62	1.13E-06	7.06E-06	Under-expressed

TMED4	-0.62	1.27E-06	7.78E-06	Under-expressed
PLPBP	-0.62	1.43E-06	8.62E-06	Under-expressed
EDEM3	-0.62	1.50E-06	9.03E-06	Under-expressed
PRCP	-0.62	2.06E-06	1.18E-05	Under-expressed
IGF2R	-0.62	2.51E-06	1.40E-05	Under-expressed
CENPC	-0.62	4.71E-06	2.44E-05	Under-expressed
CHM	-0.62	5.49E-06	2.78E-05	Under-expressed
FOXO1	-0.62	7.44E-06	3.63E-05	Under-expressed
USP18	-0.62	9.69E-05	3.41E-04	Under-expressed
GCLC	-0.62	9.90E-05	3.47E-04	Under-expressed
ALDH1B1	-0.62	1.07E-04	3.71E-04	Under-expressed
FKBP7	-0.62	1.13E-04	3.91E-04	Under-expressed
CISH	-0.62	1.81E-04	5.89E-04	Under-expressed
ZFP36	-0.62	5.14E-04	1.46E-03	Under-expressed
VHL	-0.62	5.87E-04	1.63E-03	Under-expressed
MRVI1	-0.62	7.19E-04	1.96E-03	Under-expressed
MTCP1	-0.62	1.03E-03	2.67E-03	Under-expressed
SPIN4	-0.62	1.46E-03	3.61E-03	Under-expressed
NOTCH3	-0.62	2.06E-03	4.89E-03	Under-expressed
GBP4	-0.62	2.52E-03	5.85E-03	Under-expressed
RSAD2	-0.62	3.23E-03	7.27E-03	Under-expressed
OAS2	-0.62	4.05E-03	8.84E-03	Under-expressed
RBL1	-0.62	5.52E-03	0.0116	Under-expressed
CRIM1	-0.62	0.0124	0.0235	Under-expressed
FOXC1	-0.62	0.0164	0.0301	Under-expressed
SLC35A5	-0.63	5.92E-12	2.16E-10	Under-expressed
NHLRC3	-0.63	3.11E-09	4.53E-08	Under-expressed
RMND5A	-0.63	8.85E-09	1.10E-07	Under-expressed
SERINC1	-0.63	1.61E-07	1.35E-06	Under-expressed
PEX12	-0.63	1.66E-07	1.39E-06	Under-expressed
UBR1	-0.63	1.81E-07	1.49E-06	Under-expressed
OSBPL8	-0.63	2.67E-07	2.06E-06	Under-expressed
TBC1D2B	-0.63	3.53E-07	2.62E-06	Under-expressed
EPB41	-0.63	3.87E-07	2.82E-06	Under-expressed
ATG2B	-0.63	4.60E-07	3.29E-06	Under-expressed
PTK2B	-0.63	6.98E-07	4.70E-06	Under-expressed
ERBIN	-0.63	8.42E-07	5.50E-06	Under-expressed
TJP1	-0.63	1.89E-06	1.10E-05	Under-expressed
PRKACB	-0.63	1.98E-06	1.15E-05	Under-expressed
FBXO3	-0.63	2.66E-06	1.48E-05	Under-expressed
FAM107B	-0.63	3.88E-06	2.06E-05	Under-expressed
CCM2L	-0.63	2.45E-05	1.02E-04	Under-expressed
CEP76	-0.63	3.61E-05	1.44E-04	Under-expressed
GRHPR	-0.63	3.82E-05	1.51E-04	Under-expressed
TRMT2B	-0.63	6.32E-05	2.34E-04	Under-expressed
KMT2E	-0.63	8.24E-05	2.96E-04	Under-expressed
GPR155	-0.63	1.24E-04	4.25E-04	Under-expressed
SLC23A2	-0.63	1.35E-04	4.56E-04	Under-expressed
FKBP14	-0.63	3.11E-04	9.44E-04	Under-expressed
KRT222	-0.63	5.92E-04	1.64E-03	Under-expressed
B4GALT6	-0.63	1.41E-03	3.51E-03	Under-expressed
MOCOS	-0.63	1.83E-03	4.40E-03	Under-expressed
KCNMA1	-0.63	2.35E-03	5.49E-03	Under-expressed
KCNN3	-0.63	3.36E-03	7.53E-03	Under-expressed
ZDHHC2	-0.63	4.38E-03	9.48E-03	Under-expressed
SERPINF1	-0.63	0.0104	0.0202	Under-expressed
SKIDA1	-0.63	0.0109	0.0211	Under-expressed

PIK3CG	-0.63	0.0157	0.029	Under-expressed
GFM1	-0.64	4.29E-15	3.86E-13	Under-expressed
ABHD5	-0.64	1.72E-10	3.87E-09	Under-expressed
EDEM1	-0.64	5.58E-09	7.46E-08	Under-expressed
NR3C1	-0.64	7.42E-09	9.53E-08	Under-expressed
COG3	-0.64	1.26E-08	1.50E-07	Under-expressed
UBL3	-0.64	2.41E-08	2.63E-07	Under-expressed
BIRC6	-0.64	5.14E-08	5.06E-07	Under-expressed
ROCK1	-0.64	8.89E-08	8.09E-07	Under-expressed
NOTCH4	-0.64	9.95E-08	8.89E-07	Under-expressed
FMR1	-0.64	1.22E-07	1.07E-06	Under-expressed
APBB2	-0.64	1.59E-07	1.34E-06	Under-expressed
DMXL1	-0.64	2.52E-07	1.96E-06	Under-expressed
ZCCHC14	-0.64	3.22E-07	2.42E-06	Under-expressed
TTC17	-0.64	3.93E-07	2.86E-06	Under-expressed
FOXN3	-0.64	3.99E-07	2.90E-06	Under-expressed
PEPD	-0.64	5.03E-07	3.55E-06	Under-expressed
TUBE1	-0.64	5.55E-07	3.87E-06	Under-expressed
CCDC82	-0.64	9.08E-07	5.87E-06	Under-expressed
IRF2	-0.64	1.08E-06	6.82E-06	Under-expressed
CASTOR1	-0.64	3.71E-06	1.98E-05	Under-expressed
ZHX1	-0.64	4.30E-06	2.26E-05	Under-expressed
LATS2	-0.64	4.94E-06	2.54E-05	Under-expressed
SKI	-0.64	5.22E-06	2.66E-05	Under-expressed
BBS10	-0.64	8.71E-06	4.17E-05	Under-expressed
MTSS1	-0.64	1.15E-05	5.29E-05	Under-expressed
COG6	-0.64	1.69E-05	7.43E-05	Under-expressed
DISP1	-0.64	1.84E-05	7.96E-05	Under-expressed
MBNL2	-0.64	1.88E-05	8.10E-05	Under-expressed
PHF8	-0.64	2.38E-05	1.00E-04	Under-expressed
JDP2	-0.64	2.53E-05	1.05E-04	Under-expressed
KLF12	-0.64	1.31E-04	4.45E-04	Under-expressed
SH3BGRL2	-0.64	1.38E-04	4.63E-04	Under-expressed
TIGD2	-0.64	1.94E-04	6.29E-04	Under-expressed
IL1R1	-0.64	2.99E-04	9.12E-04	Under-expressed
SLC25A25	-0.64	3.48E-04	1.04E-03	Under-expressed
ZNF396	-0.64	3.57E-04	1.07E-03	Under-expressed
TRAF3IP2	-0.64	4.85E-04	1.39E-03	Under-expressed
CDKN1A	-0.64	3.53E-03	7.84E-03	Under-expressed
WWTR1	-0.64	5.20E-03	0.011	Under-expressed
EDN1	-0.64	5.91E-03	0.0123	Under-expressed
DTX4	-0.64	9.70E-03	0.019	Under-expressed
FRZB	-0.64	0.0104	0.0203	Under-expressed
PHKA1	-0.64	0.0198	0.0355	Under-expressed
BDKRB2	-0.64	0.0245	0.0429	Under-expressed
MTMR6	-0.65	1.03E-09	1.77E-08	Under-expressed
LIMD1	-0.65	1.46E-09	2.36E-08	Under-expressed
SCRN3	-0.65	1.73E-09	2.71E-08	Under-expressed
CNST	-0.65	4.85E-09	6.61E-08	Under-expressed
SUOX	-0.65	4.66E-08	4.62E-07	Under-expressed
PTAR1	-0.65	5.24E-08	5.14E-07	Under-expressed
UHRF1BP1L	-0.65	1.92E-07	1.56E-06	Under-expressed
ATXN7	-0.65	2.30E-07	1.81E-06	Under-expressed
UBXN4	-0.65	3.37E-07	2.51E-06	Under-expressed
CDC37L1	-0.65	4.82E-07	3.42E-06	Under-expressed
FN1	-0.65	5.61E-07	3.90E-06	Under-expressed
YTHDC2	-0.65	9.08E-07	5.87E-06	Under-expressed

RSF1	-0.65	1.13E-06	7.06E-06	Under-expressed
UBE2H	-0.65	1.17E-06	7.25E-06	Under-expressed
HSDL2	-0.65	2.97E-06	1.63E-05	Under-expressed
ARMT1	-0.65	4.64E-06	2.41E-05	Under-expressed
SDC1	-0.65	1.34E-05	6.04E-05	Under-expressed
LMO7	-0.65	1.73E-05	7.54E-05	Under-expressed
LDLRAD4	-0.65	3.02E-05	1.23E-04	Under-expressed
WDR47	-0.65	3.49E-05	1.40E-04	Under-expressed
NPHP3	-0.65	4.34E-05	1.69E-04	Under-expressed
SUSD6	-0.65	4.38E-05	1.70E-04	Under-expressed
SGK3	-0.65	4.65E-05	1.79E-04	Under-expressed
SH3BGRL	-0.65	4.69E-05	1.80E-04	Under-expressed
POR	-0.65	5.89E-05	2.21E-04	Under-expressed
ZNF217	-0.65	2.07E-04	6.62E-04	Under-expressed
COL4A1	-0.65	2.88E-04	8.81E-04	Under-expressed
TAF2	-0.65	3.23E-04	9.75E-04	Under-expressed
A1BG-AS1	-0.65	3.59E-04	1.07E-03	Under-expressed
TCAF1	-0.65	7.01E-04	1.91E-03	Under-expressed
RNASE4	-0.65	8.71E-04	2.31E-03	Under-expressed
SLFN5	-0.65	1.11E-03	2.84E-03	Under-expressed
ATP7B	-0.65	1.23E-03	3.10E-03	Under-expressed
ADAMTSL4	-0.65	1.32E-03	3.30E-03	Under-expressed
PAMR1	-0.65	2.16E-03	5.10E-03	Under-expressed
FAAH	-0.65	2.31E-03	5.42E-03	Under-expressed
TNFSF13	-0.65	3.85E-03	8.44E-03	Under-expressed
FGD1	-0.65	6.22E-03	0.0129	Under-expressed
ANTXR1	-0.65	0.0113	0.0216	Under-expressed
GADD45G	-0.65	0.0186	0.0336	Under-expressed
KCTD21	-0.66	8.79E-10	1.53E-08	Under-expressed
TRIP11	-0.66	1.10E-09	1.86E-08	Under-expressed
NDUFS1	-0.66	1.60E-09	2.53E-08	Under-expressed
BCAR3	-0.66	4.28E-09	5.94E-08	Under-expressed
TRIM62	-0.66	7.76E-09	9.86E-08	Under-expressed
TOPORS	-0.66	2.42E-08	2.64E-07	Under-expressed
PEX26	-0.66	1.63E-07	1.37E-06	Under-expressed
PPFIA1	-0.66	2.78E-07	2.13E-06	Under-expressed
EPM2A	-0.66	5.19E-07	3.64E-06	Under-expressed
ZNF720	-0.66	5.28E-07	3.69E-06	Under-expressed
RMI1	-0.66	8.27E-07	5.42E-06	Under-expressed
KLHL24	-0.66	1.03E-06	6.53E-06	Under-expressed
COL18A1	-0.66	2.07E-06	1.19E-05	Under-expressed
TUT7	-0.66	3.37E-06	1.81E-05	Under-expressed
GIMAP8	-0.66	4.69E-06	2.43E-05	Under-expressed
TGFBR2	-0.66	4.81E-06	2.48E-05	Under-expressed
PHF2	-0.66	7.00E-06	3.44E-05	Under-expressed
RDX	-0.66	7.18E-06	3.52E-05	Under-expressed
TCF4	-0.66	8.12E-06	3.91E-05	Under-expressed
CFL2	-0.66	9.25E-06	4.39E-05	Under-expressed
ZNF684	-0.66	1.44E-05	6.42E-05	Under-expressed
SPRED2	-0.66	2.30E-05	9.70E-05	Under-expressed
ENG	-0.66	3.05E-05	1.24E-04	Under-expressed
SRFBP1	-0.66	5.69E-05	2.14E-04	Under-expressed
PANK1	-0.66	8.01E-05	2.89E-04	Under-expressed
CYTH3	-0.66	1.03E-04	3.59E-04	Under-expressed
STAG3L2	-0.66	4.78E-04	1.37E-03	Under-expressed
COPZ2	-0.66	1.84E-03	4.41E-03	Under-expressed
SNRK	-0.67	6.51E-12	2.35E-10	Under-expressed

TAOK3	-0.67	1.95E-09	3.01E-08	Under-expressed
PPP1R12B	-0.67	3.01E-09	4.39E-08	Under-expressed
DNAJC25	-0.67	1.97E-08	2.21E-07	Under-expressed
FBXO8	-0.67	3.70E-08	3.82E-07	Under-expressed
SPG11	-0.67	4.58E-08	4.55E-07	Under-expressed
PJA2	-0.67	6.21E-08	5.90E-07	Under-expressed
ZNF75D	-0.67	7.07E-08	6.62E-07	Under-expressed
RANBP6	-0.67	1.40E-07	1.20E-06	Under-expressed
BMPR2	-0.67	8.79E-07	5.72E-06	Under-expressed
NSUN6	-0.67	9.19E-07	5.93E-06	Under-expressed
OAF	-0.67	1.43E-06	8.62E-06	Under-expressed
B3GNT2	-0.67	1.63E-06	9.71E-06	Under-expressed
TNS3	-0.67	1.89E-06	1.10E-05	Under-expressed
VPS13C	-0.67	1.99E-06	1.15E-05	Under-expressed
THRA	-0.67	3.45E-06	1.85E-05	Under-expressed
SAMD8	-0.67	3.71E-06	1.98E-05	Under-expressed
BLZF1	-0.67	5.39E-06	2.74E-05	Under-expressed
AKAP9	-0.67	9.28E-06	4.40E-05	Under-expressed
RGL1	-0.67	9.49E-06	4.49E-05	Under-expressed
INTS2	-0.67	9.69E-06	4.57E-05	Under-expressed
PTPN21	-0.67	1.05E-05	4.90E-05	Under-expressed
MDM1	-0.67	1.28E-05	5.83E-05	Under-expressed
ZNF776	-0.67	1.56E-05	6.88E-05	Under-expressed
MOB1A	-0.67	1.87E-05	8.07E-05	Under-expressed
C3ORF18	-0.67	2.01E-05	8.62E-05	Under-expressed
ACAD11	-0.67	2.66E-05	1.10E-04	Under-expressed
IFIT3	-0.67	4.36E-05	1.69E-04	Under-expressed
DHX58	-0.67	4.41E-05	1.71E-04	Under-expressed
CSAD	-0.67	8.45E-05	3.03E-04	Under-expressed
IGFBP4	-0.67	1.38E-04	4.63E-04	Under-expressed
TMEM170B	-0.67	1.40E-04	4.69E-04	Under-expressed
RNF152	-0.67	1.58E-04	5.22E-04	Under-expressed
IRAK3	-0.67	4.75E-04	1.36E-03	Under-expressed
FRMD6	-0.67	9.07E-04	2.39E-03	Under-expressed
PLCXD2	-0.67	1.02E-03	2.64E-03	Under-expressed
LNX1	-0.67	1.16E-03	2.96E-03	Under-expressed
DUSP1	-0.67	1.50E-03	3.69E-03	Under-expressed
CSF1	-0.67	4.25E-03	9.21E-03	Under-expressed
GPRIN3	-0.67	4.57E-03	9.84E-03	Under-expressed
ALS2CL	-0.67	4.92E-03	0.0105	Under-expressed
CIART	-0.67	9.39E-03	0.0185	Under-expressed
APC	-0.68	1.46E-09	2.35E-08	Under-expressed
TBC1D24	-0.68	2.66E-09	3.95E-08	Under-expressed
SOCS6	-0.68	1.26E-08	1.50E-07	Under-expressed
PALM2-AKAP2	-0.68	1.91E-08	2.16E-07	Under-expressed
SEC23A	-0.68	1.84E-07	1.50E-06	Under-expressed
LARP1B	-0.68	1.95E-07	1.58E-06	Under-expressed
LNPK	-0.68	3.39E-07	2.53E-06	Under-expressed
ARHGAP5	-0.68	3.98E-07	2.90E-06	Under-expressed
ZFAND4	-0.68	4.34E-07	3.12E-06	Under-expressed
NCK1	-0.68	1.31E-06	8.02E-06	Under-expressed
IFIH1	-0.68	2.42E-06	1.36E-05	Under-expressed
IDE	-0.68	2.45E-06	1.38E-05	Under-expressed
ADAL	-0.68	2.99E-06	1.64E-05	Under-expressed
N4BP2L1	-0.68	4.43E-06	2.32E-05	Under-expressed
CASC4	-0.68	4.58E-06	2.38E-05	Under-expressed
KATNAL1	-0.68	5.20E-06	2.65E-05	Under-expressed

RASGRP3	-0.68	6.72E-06	3.32E-05	Under-expressed
TAL1	-0.68	9.86E-06	4.64E-05	Under-expressed
MAML3	-0.68	1.04E-05	4.84E-05	Under-expressed
LRIG1	-0.68	1.18E-05	5.43E-05	Under-expressed
MYO5A	-0.68	1.44E-05	6.42E-05	Under-expressed
RHOJ	-0.68	2.65E-05	1.10E-04	Under-expressed
GPRASP2	-0.68	2.66E-05	1.11E-04	Under-expressed
HEATR3	-0.68	3.02E-05	1.23E-04	Under-expressed
SSX2IP	-0.68	4.52E-05	1.75E-04	Under-expressed
DHRS1	-0.68	6.39E-05	2.36E-04	Under-expressed
ADCY4	-0.68	8.56E-05	3.06E-04	Under-expressed
HSPG2	-0.68	1.04E-04	3.63E-04	Under-expressed
ENPP4	-0.68	1.05E-04	3.65E-04	Under-expressed
EBF1	-0.68	1.42E-04	4.76E-04	Under-expressed
DCK	-0.68	3.64E-04	1.08E-03	Under-expressed
LIMS2	-0.68	6.06E-04	1.68E-03	Under-expressed
ANKRD44	-0.68	8.10E-04	2.17E-03	Under-expressed
MSMO1	-0.68	1.19E-03	3.02E-03	Under-expressed
AKT3	-0.68	1.74E-03	4.22E-03	Under-expressed
GBP1	-0.68	1.79E-03	4.32E-03	Under-expressed
LOC100130872	-0.68	3.50E-03	7.79E-03	Under-expressed
ANG	-0.68	0.0137	0.0257	Under-expressed
CTAGE9	-0.68	0.0281	0.0485	Under-expressed
XRN1	-0.69	1.28E-10	3.04E-09	Under-expressed
RHBDD1	-0.69	3.21E-10	6.56E-09	Under-expressed
SDHD	-0.69	6.45E-10	1.18E-08	Under-expressed
ZBTB7B	-0.69	4.98E-09	6.76E-08	Under-expressed
RAP2C	-0.69	5.51E-09	7.39E-08	Under-expressed
ZFYVE9	-0.69	7.67E-09	9.81E-08	Under-expressed
AK3	-0.69	8.38E-09	1.05E-07	Under-expressed
IVD	-0.69	1.02E-08	1.25E-07	Under-expressed
SLC10A7	-0.69	7.88E-08	7.27E-07	Under-expressed
ZBTB21	-0.69	1.55E-07	1.31E-06	Under-expressed
SDE2	-0.69	3.84E-07	2.81E-06	Under-expressed
NT5DC1	-0.69	4.72E-07	3.35E-06	Under-expressed
GTF2IRD2P1	-0.69	5.62E-07	3.90E-06	Under-expressed
ATRN	-0.69	6.18E-07	4.24E-06	Under-expressed
NFE2L2	-0.69	6.49E-07	4.43E-06	Under-expressed
TMEM131L	-0.69	1.24E-06	7.64E-06	Under-expressed
TRIL	-0.69	2.23E-06	1.26E-05	Under-expressed
SLC25A43	-0.69	7.81E-06	3.78E-05	Under-expressed
C1ORF74	-0.69	8.72E-06	4.17E-05	Under-expressed
UBXN7	-0.69	1.80E-05	7.82E-05	Under-expressed
RHOB	-0.69	2.85E-05	1.17E-04	Under-expressed
JMJD1C	-0.69	4.35E-05	1.69E-04	Under-expressed
NFIB	-0.69	1.06E-04	3.70E-04	Under-expressed
PDZD8	-0.69	3.42E-04	1.03E-03	Under-expressed
CRYL1	-0.69	4.33E-04	1.26E-03	Under-expressed
SYNM	-0.69	8.54E-04	2.27E-03	Under-expressed
CEACAM1	-0.69	9.97E-04	2.59E-03	Under-expressed
CAV2	-0.69	0.0111	0.0214	Under-expressed
MOSMO	-0.7	5.94E-10	1.10E-08	Under-expressed
TTPAL	-0.7	1.13E-09	1.90E-08	Under-expressed
SEC24A	-0.7	2.89E-09	4.24E-08	Under-expressed
DCAF11	-0.7	7.75E-09	9.86E-08	Under-expressed
AGO4	-0.7	1.14E-08	1.38E-07	Under-expressed
LAMP2	-0.7	1.99E-08	2.23E-07	Under-expressed

ACO1	-0.7	3.69E-08	3.82E-07	Under-expressed
ANO6	-0.7	4.16E-08	4.21E-07	Under-expressed
TRAF6	-0.7	5.93E-08	5.68E-07	Under-expressed
MLYCD	-0.7	1.81E-07	1.49E-06	Under-expressed
ARSD	-0.7	5.04E-07	3.55E-06	Under-expressed
TBC1D2	-0.7	9.71E-07	6.22E-06	Under-expressed
KIAA2026	-0.7	3.09E-06	1.68E-05	Under-expressed
ENTPD4	-0.7	3.78E-06	2.01E-05	Under-expressed
DYNLT3	-0.7	5.40E-06	2.74E-05	Under-expressed
DDAH1	-0.7	7.21E-06	3.53E-05	Under-expressed
LRRC40	-0.7	8.03E-06	3.87E-05	Under-expressed
ZXDB	-0.7	2.25E-05	9.51E-05	Under-expressed
SLC39A14	-0.7	3.15E-05	1.28E-04	Under-expressed
PTPN3	-0.7	5.83E-05	2.19E-04	Under-expressed
SAMD4B	-0.7	5.89E-05	2.20E-04	Under-expressed
PODXL	-0.7	1.20E-04	4.11E-04	Under-expressed
CDK14	-0.7	1.26E-04	4.30E-04	Under-expressed
MR1	-0.7	1.34E-04	4.53E-04	Under-expressed
LONRF3	-0.7	3.23E-04	9.75E-04	Under-expressed
CPE	-0.7	7.21E-04	1.96E-03	Under-expressed
SVIL	-0.7	7.41E-04	2.01E-03	Under-expressed
XAF1	-0.7	1.03E-03	2.67E-03	Under-expressed
LYVE1	-0.7	1.60E-03	3.91E-03	Under-expressed
MBOAT1	-0.7	1.85E-03	4.43E-03	Under-expressed
GLT8D2	-0.7	2.27E-03	5.33E-03	Under-expressed
CYP2J2	-0.7	0.0157	0.029	Under-expressed
GEM	-0.7	0.022	0.0391	Under-expressed
TPMT	-0.71	1.74E-10	3.88E-09	Under-expressed
RAB43	-0.71	2.12E-10	4.58E-09	Under-expressed
MAN2B2	-0.71	2.65E-10	5.60E-09	Under-expressed
TAB2	-0.71	6.64E-10	1.20E-08	Under-expressed
HADH	-0.71	6.71E-08	6.32E-07	Under-expressed
SNX19	-0.71	1.27E-07	1.10E-06	Under-expressed
ATXN1L	-0.71	3.45E-07	2.57E-06	Under-expressed
SLC25A46	-0.71	4.81E-07	3.41E-06	Under-expressed
HMBOX1	-0.71	5.39E-07	3.76E-06	Under-expressed
DPY19L1	-0.71	6.28E-07	4.30E-06	Under-expressed
CCDC15	-0.71	1.90E-06	1.10E-05	Under-expressed
MIGA1	-0.71	2.51E-06	1.40E-05	Under-expressed
TTC8	-0.71	2.67E-06	1.48E-05	Under-expressed
CDH13	-0.71	3.37E-06	1.82E-05	Under-expressed
ZNF367	-0.71	1.44E-05	6.45E-05	Under-expressed
SLC30A6	-0.71	2.90E-05	1.19E-04	Under-expressed
NLRP1	-0.71	4.82E-05	1.85E-04	Under-expressed
LPIN1	-0.71	6.81E-05	2.50E-04	Under-expressed
RBBP4	-0.71	1.73E-04	5.67E-04	Under-expressed
ZNF236	-0.71	2.21E-04	7.00E-04	Under-expressed
SP4	-0.71	2.87E-04	8.79E-04	Under-expressed
KIAA1107	-0.71	3.05E-04	9.30E-04	Under-expressed
ZBTB26	-0.71	1.53E-03	3.76E-03	Under-expressed
NBPF15	-0.71	4.00E-03	8.74E-03	Under-expressed
NR1H4	-0.71	9.12E-03	0.018	Under-expressed
AGTR1	-0.71	0.0253	0.0442	Under-expressed
RANBP10	-0.72	1.09E-13	7.13E-12	Under-expressed
TAPT1	-0.72	5.25E-12	1.95E-10	Under-expressed
FLT1	-0.72	7.05E-10	1.27E-08	Under-expressed
HECTD1	-0.72	1.83E-09	2.84E-08	Under-expressed

PNPLA8	-0.72	3.88E-08	3.99E-07	Under-expressed
C9ORF64	-0.72	5.39E-08	5.26E-07	Under-expressed
ZEB1	-0.72	7.24E-08	6.75E-07	Under-expressed
CHAMP1	-0.72	1.45E-07	1.24E-06	Under-expressed
ZNF41	-0.72	2.43E-07	1.89E-06	Under-expressed
TIFA	-0.72	1.27E-06	7.80E-06	Under-expressed
DUSP19	-0.72	3.58E-06	1.92E-05	Under-expressed
GCLM	-0.72	6.56E-06	3.25E-05	Under-expressed
ZFAND5	-0.72	7.04E-06	3.45E-05	Under-expressed
GPATCH2L	-0.72	1.02E-05	4.77E-05	Under-expressed
NEK4	-0.72	1.33E-05	6.00E-05	Under-expressed
CNTLN	-0.72	1.49E-05	6.60E-05	Under-expressed
ZNRF2P1	-0.72	4.40E-05	1.71E-04	Under-expressed
SLC5A3	-0.72	7.48E-05	2.71E-04	Under-expressed
BHLHB9	-0.72	1.35E-04	4.56E-04	Under-expressed
PEX11G	-0.72	2.62E-04	8.13E-04	Under-expressed
IKZF2	-0.72	5.63E-04	1.58E-03	Under-expressed
PARM1	-0.72	5.71E-04	1.60E-03	Under-expressed
EHD3	-0.72	8.38E-04	2.24E-03	Under-expressed
SHROOM3	-0.72	1.01E-03	2.62E-03	Under-expressed
CHSY3	-0.72	1.42E-03	3.52E-03	Under-expressed
GBP3	-0.72	2.29E-03	5.37E-03	Under-expressed
KCNAB2	-0.72	3.05E-03	6.92E-03	Under-expressed
DLL1	-0.72	7.23E-03	0.0147	Under-expressed
CYP27A1	-0.72	9.62E-03	0.0189	Under-expressed
CDH11	-0.72	0.0184	0.0333	Under-expressed
DEPDC5	-0.73	8.11E-13	3.90E-11	Under-expressed
USP38	-0.73	1.82E-10	4.04E-09	Under-expressed
RIC1	-0.73	1.35E-09	2.20E-08	Under-expressed
ARHGEF12	-0.73	1.94E-08	2.18E-07	Under-expressed
PIK3C2A	-0.73	3.18E-08	3.35E-07	Under-expressed
ARHGAP21	-0.73	4.28E-08	4.31E-07	Under-expressed
LACC1	-0.73	9.00E-08	8.18E-07	Under-expressed
CBR4	-0.73	1.46E-07	1.25E-06	Under-expressed
SIK2	-0.73	1.72E-07	1.43E-06	Under-expressed
CMTR2	-0.73	2.04E-07	1.65E-06	Under-expressed
ERLIN2	-0.73	2.06E-07	1.66E-06	Under-expressed
ASH1L	-0.73	3.64E-07	2.68E-06	Under-expressed
RETSAT	-0.73	5.10E-07	3.59E-06	Under-expressed
ROBO4	-0.73	7.64E-07	5.07E-06	Under-expressed
ARFGEF2	-0.73	1.12E-06	7.05E-06	Under-expressed
OTUD4	-0.73	2.02E-06	1.16E-05	Under-expressed
MYCT1	-0.73	3.25E-06	1.75E-05	Under-expressed
LINC00667	-0.73	5.35E-06	2.72E-05	Under-expressed
CDC42EP3	-0.73	1.87E-05	8.09E-05	Under-expressed
DNASE1L1	-0.73	3.49E-05	1.40E-04	Under-expressed
FAM122C	-0.73	5.21E-05	1.98E-04	Under-expressed
PAPSS2	-0.73	7.99E-05	2.88E-04	Under-expressed
ITPR2	-0.73	8.14E-05	2.93E-04	Under-expressed
TACC1	-0.73	2.26E-04	7.15E-04	Under-expressed
ERRFI1	-0.73	3.75E-04	1.11E-03	Under-expressed
ADRA2B	-0.73	4.12E-04	1.20E-03	Under-expressed
SIRT1	-0.73	5.27E-04	1.49E-03	Under-expressed
C3	-0.73	3.61E-03	7.99E-03	Under-expressed
WDR7	-0.74	1.64E-13	1.02E-11	Under-expressed
CALD1	-0.74	3.19E-13	1.76E-11	Under-expressed
C12ORF66	-0.74	2.89E-12	1.16E-10	Under-expressed

SEC24B	-0.74	4.94E-10	9.48E-09	Under-expressed
NEK1	-0.74	5.47E-10	1.03E-08	Under-expressed
SPRY3	-0.74	5.79E-10	1.08E-08	Under-expressed
CLIC4	-0.74	1.03E-09	1.76E-08	Under-expressed
ACAP2	-0.74	3.57E-09	5.10E-08	Under-expressed
KMT2C	-0.74	3.99E-09	5.60E-08	Under-expressed
ZNF441	-0.74	1.43E-08	1.68E-07	Under-expressed
ZNF275	-0.74	8.04E-08	7.40E-07	Under-expressed
TIMP3	-0.74	1.93E-07	1.57E-06	Under-expressed
EP300	-0.74	6.76E-07	4.58E-06	Under-expressed
PLCG2	-0.74	1.69E-06	1.00E-05	Under-expressed
ALDH7A1	-0.74	4.91E-06	2.53E-05	Under-expressed
ADAMTS4	-0.74	6.43E-06	3.20E-05	Under-expressed
SPRY1	-0.74	6.77E-06	3.34E-05	Under-expressed
KCTD18	-0.74	7.09E-06	3.48E-05	Under-expressed
PECR	-0.74	2.48E-05	1.04E-04	Under-expressed
ARHGAP23	-0.74	2.67E-05	1.11E-04	Under-expressed
GNAI1	-0.74	3.84E-05	1.52E-04	Under-expressed
TM4SF18	-0.74	4.03E-05	1.58E-04	Under-expressed
METTL7A	-0.74	4.36E-05	1.70E-04	Under-expressed
ATP10D	-0.74	4.97E-05	1.90E-04	Under-expressed
RBPMS2	-0.74	1.67E-03	4.06E-03	Under-expressed
CXXC5	-0.74	1.79E-03	4.32E-03	Under-expressed
NEXN	-0.74	3.57E-03	7.91E-03	Under-expressed
SNAI2	-0.74	6.97E-03	0.0142	Under-expressed
FGF13	-0.74	9.65E-03	0.0189	Under-expressed
CTBS	-0.75	1.43E-10	3.33E-09	Under-expressed
PARP9	-0.75	1.16E-09	1.94E-08	Under-expressed
CELF1	-0.75	3.29E-09	4.74E-08	Under-expressed
CCDC126	-0.75	8.74E-09	1.09E-07	Under-expressed
SOS2	-0.75	1.11E-08	1.35E-07	Under-expressed
ACBD5	-0.75	3.03E-08	3.22E-07	Under-expressed
CPT1A	-0.75	4.10E-08	4.17E-07	Under-expressed
ACOX1	-0.75	4.60E-08	4.56E-07	Under-expressed
ZBTB38	-0.75	6.39E-07	4.37E-06	Under-expressed
ARHGAP31	-0.75	7.17E-07	4.81E-06	Under-expressed
ITPRID2	-0.75	1.61E-06	9.59E-06	Under-expressed
GPHN	-0.75	1.88E-06	1.10E-05	Under-expressed
DNAH1	-0.75	4.14E-06	2.18E-05	Under-expressed
JAK2	-0.75	4.75E-06	2.45E-05	Under-expressed
ARHGAP29	-0.75	5.96E-06	2.99E-05	Under-expressed
OAS3	-0.75	9.07E-06	4.32E-05	Under-expressed
HEG1	-0.75	1.20E-05	5.49E-05	Under-expressed
CRPPA	-0.75	1.86E-05	8.03E-05	Under-expressed
LHFPL6	-0.75	2.94E-05	1.20E-04	Under-expressed
CMPK2	-0.75	9.50E-05	3.35E-04	Under-expressed
PNPLA7	-0.75	2.76E-04	8.50E-04	Under-expressed
SYTL2	-0.75	3.04E-04	9.26E-04	Under-expressed
ENPP1	-0.75	3.22E-04	9.73E-04	Under-expressed
IFT81	-0.75	7.37E-04	2.00E-03	Under-expressed
TLR1	-0.75	7.69E-04	2.07E-03	Under-expressed
ACKR3	-0.75	8.88E-04	2.35E-03	Under-expressed
PTGR1	-0.75	8.65E-03	0.0172	Under-expressed
ELOVL2	-0.75	9.56E-03	0.0188	Under-expressed
FYCO1	-0.76	7.73E-12	2.72E-10	Under-expressed
PIK3R4	-0.76	6.47E-11	1.70E-09	Under-expressed
FBXL5	-0.76	1.12E-10	2.70E-09	Under-expressed

TMEM184C	-0.76	6.16E-10	1.13E-08	Under-expressed
TOR1AIP1	-0.76	5.36E-09	7.24E-08	Under-expressed
GOLGA4	-0.76	6.15E-09	8.11E-08	Under-expressed
ZBTB18	-0.76	1.77E-08	2.02E-07	Under-expressed
ZCCHC2	-0.76	4.58E-08	4.55E-07	Under-expressed
SH3BP4	-0.76	1.38E-07	1.19E-06	Under-expressed
CRY1	-0.76	1.90E-07	1.55E-06	Under-expressed
ZBTB43	-0.76	2.20E-07	1.75E-06	Under-expressed
ARRDC3	-0.76	3.29E-06	1.77E-05	Under-expressed
RGP1	-0.76	6.32E-06	3.15E-05	Under-expressed
BDNF-AS	-0.76	1.13E-05	5.22E-05	Under-expressed
NEK7	-0.76	1.37E-05	6.16E-05	Under-expressed
SEPTIN10	-0.76	1.63E-05	7.18E-05	Under-expressed
SLC25A42	-0.76	2.54E-04	7.90E-04	Under-expressed
GAS6	-0.76	4.78E-04	1.37E-03	Under-expressed
NPIPB5	-0.76	7.41E-04	2.01E-03	Under-expressed
AQP11	-0.76	8.57E-04	2.28E-03	Under-expressed
CD109	-0.76	2.00E-03	4.75E-03	Under-expressed
AMACR	-0.76	2.68E-03	6.18E-03	Under-expressed
AGMAT	-0.76	3.66E-03	8.08E-03	Under-expressed
APLNR	-0.76	5.44E-03	0.0114	Under-expressed
AKR7L	-0.76	8.64E-03	0.0172	Under-expressed
SYT7	-0.76	9.37E-03	0.0184	Under-expressed
REEP6	-0.76	0.0127	0.0241	Under-expressed
SLC25A18	-0.76	0.0233	0.0411	Under-expressed
AP5M1	-0.77	5.50E-14	3.88E-12	Under-expressed
TANGO6	-0.77	2.12E-10	4.58E-09	Under-expressed
LYSMD3	-0.77	1.51E-09	2.42E-08	Under-expressed
SYNJ1	-0.77	3.46E-09	4.96E-08	Under-expressed
NRIP1	-0.77	1.10E-08	1.34E-07	Under-expressed
FNDC3A	-0.77	2.66E-08	2.88E-07	Under-expressed
TECPR1	-0.77	4.29E-07	3.09E-06	Under-expressed
CHAC2	-0.77	5.56E-07	3.87E-06	Under-expressed
KIDINS220	-0.77	1.13E-06	7.10E-06	Under-expressed
PRIMPOL	-0.77	1.37E-06	8.30E-06	Under-expressed
MBP	-0.77	1.69E-06	1.00E-05	Under-expressed
DDX60L	-0.77	2.08E-06	1.19E-05	Under-expressed
ATP8B4	-0.77	5.40E-06	2.74E-05	Under-expressed
USP53	-0.77	6.09E-06	3.04E-05	Under-expressed
ELF1	-0.77	1.12E-05	5.21E-05	Under-expressed
TAF1	-0.77	1.77E-05	7.69E-05	Under-expressed
PID1	-0.77	5.67E-05	2.13E-04	Under-expressed
F2R	-0.77	2.26E-04	7.14E-04	Under-expressed
RASA1	-0.77	2.64E-04	8.16E-04	Under-expressed
SHISA4	-0.77	6.27E-04	1.73E-03	Under-expressed
NID2	-0.77	6.60E-04	1.82E-03	Under-expressed
FAM221A	-0.77	2.18E-03	5.16E-03	Under-expressed
KHK	-0.77	4.91E-03	0.0105	Under-expressed
DISP2	-0.77	5.81E-03	0.0121	Under-expressed
DCXR	-0.77	5.97E-03	0.0124	Under-expressed
PRMT9	-0.78	3.80E-10	7.58E-09	Under-expressed
SEL1L	-0.78	5.39E-10	1.02E-08	Under-expressed
HIBADH	-0.78	1.11E-09	1.87E-08	Under-expressed
MYCBP2	-0.78	4.27E-09	5.93E-08	Under-expressed
CRY2	-0.78	5.23E-09	7.08E-08	Under-expressed
LRCH1	-0.78	5.76E-09	7.66E-08	Under-expressed
MAGI1	-0.78	1.44E-08	1.68E-07	Under-expressed

KLHL15	-0.78	1.61E-08	1.86E-07	Under-expressed
DHFR2	-0.78	3.45E-08	3.59E-07	Under-expressed
RCBTB1	-0.78	3.47E-08	3.61E-07	Under-expressed
NBPF1	-0.78	5.53E-08	5.36E-07	Under-expressed
TRIM44	-0.78	6.02E-08	5.76E-07	Under-expressed
MBIP	-0.78	1.82E-07	1.50E-06	Under-expressed
H6PD	-0.78	1.87E-07	1.53E-06	Under-expressed
ACAA2	-0.78	3.49E-07	2.60E-06	Under-expressed
PIKFYVE	-0.78	7.63E-07	5.07E-06	Under-expressed
KALRN	-0.78	1.66E-06	9.84E-06	Under-expressed
LMLN	-0.78	2.69E-06	1.49E-05	Under-expressed
GTF2H3	-0.78	2.93E-06	1.60E-05	Under-expressed
SLC25A30	-0.78	5.20E-06	2.65E-05	Under-expressed
THRB	-0.78	6.27E-06	3.13E-05	Under-expressed
SGMS2	-0.78	2.43E-05	1.02E-04	Under-expressed
TRIO	-0.78	3.04E-05	1.23E-04	Under-expressed
STEAP3	-0.78	5.68E-05	2.14E-04	Under-expressed
DIPK2A	-0.78	8.92E-05	3.17E-04	Under-expressed
SGK1	-0.78	9.99E-05	3.50E-04	Under-expressed
PLEKHA7	-0.78	1.39E-04	4.66E-04	Under-expressed
SMO	-0.78	4.62E-04	1.33E-03	Under-expressed
FAM20A	-0.78	4.98E-04	1.42E-03	Under-expressed
IFI44	-0.78	5.80E-04	1.62E-03	Under-expressed
DIP2C	-0.78	1.16E-03	2.95E-03	Under-expressed
FADS2	-0.78	9.75E-03	0.0191	Under-expressed
EPAS1	-0.79	5.80E-12	2.12E-10	Under-expressed
HERC1	-0.79	1.43E-11	4.64E-10	Under-expressed
VAMP7	-0.79	1.45E-10	3.36E-09	Under-expressed
SH3D19	-0.79	2.38E-09	3.59E-08	Under-expressed
IMPACT	-0.79	2.48E-09	3.71E-08	Under-expressed
CHD9	-0.79	6.28E-09	8.24E-08	Under-expressed
ANKRD50	-0.79	7.00E-08	6.57E-07	Under-expressed
CITED2	-0.79	2.32E-07	1.82E-06	Under-expressed
TM9SF2	-0.79	3.10E-07	2.35E-06	Under-expressed
SMAD1	-0.79	6.50E-07	4.44E-06	Under-expressed
ARHGAP32	-0.79	6.92E-07	4.67E-06	Under-expressed
NFIX	-0.79	7.48E-07	4.98E-06	Under-expressed
MOSPD2	-0.79	7.52E-07	5.00E-06	Under-expressed
CAPN5	-0.79	7.83E-07	5.18E-06	Under-expressed
SMG1P3	-0.79	2.76E-05	1.14E-04	Under-expressed
NID1	-0.79	5.71E-05	2.15E-04	Under-expressed
C4ORF19	-0.79	1.16E-04	4.01E-04	Under-expressed
MGLL	-0.79	1.46E-04	4.87E-04	Under-expressed
APOL3	-0.79	2.11E-04	6.72E-04	Under-expressed
RIDA	-0.79	2.22E-04	7.03E-04	Under-expressed
RGPD3	-0.79	2.79E-04	8.57E-04	Under-expressed
TMTC1	-0.79	9.16E-04	2.41E-03	Under-expressed
ITM2A	-0.79	1.58E-03	3.87E-03	Under-expressed
SOCS2	-0.79	2.30E-03	5.39E-03	Under-expressed
CCL14	-0.79	2.65E-03	6.12E-03	Under-expressed
WLS	-0.79	4.15E-03	9.03E-03	Under-expressed
NUDT12	-0.79	4.35E-03	9.43E-03	Under-expressed
ITIH2	-0.79	0.013	0.0245	Under-expressed
PLIN1	-0.79	0.0177	0.0322	Under-expressed
LIG4	-0.8	2.44E-09	3.67E-08	Under-expressed
SAV1	-0.8	4.12E-09	5.75E-08	Under-expressed
LOC100130093	-0.8	4.90E-07	3.47E-06	Under-expressed

RCAN1	-0.8	5.36E-07	3.74E-06	Under-expressed
CLMN	-0.8	7.41E-07	4.95E-06	Under-expressed
GPR146	-0.8	2.27E-06	1.28E-05	Under-expressed
PRRG1	-0.8	3.23E-06	1.75E-05	Under-expressed
POGLUT3	-0.8	5.40E-06	2.74E-05	Under-expressed
WWC2	-0.8	1.21E-05	5.55E-05	Under-expressed
LRRCC1	-0.8	1.33E-04	4.50E-04	Under-expressed
KITLG	-0.8	1.34E-04	4.53E-04	Under-expressed
SYNJ2	-0.8	3.33E-04	1.00E-03	Under-expressed
HAAO	-0.8	3.63E-04	1.08E-03	Under-expressed
STAG3L1	-0.8	4.53E-04	1.31E-03	Under-expressed
RTN4RL2	-0.8	1.10E-03	2.82E-03	Under-expressed
MOB3B	-0.8	1.81E-03	4.37E-03	Under-expressed
NR4A2	-0.8	1.83E-03	4.40E-03	Under-expressed
TMEM25	-0.8	2.81E-03	6.45E-03	Under-expressed
ATF5	-0.8	0.0103	0.0201	Under-expressed
MXRA5	-0.8	0.015	0.0278	Under-expressed
ABCB1	-0.8	0.0152	0.0282	Under-expressed
ATOH8	-0.8	0.0229	0.0404	Under-expressed
CCPG1	-0.81	1.98E-10	4.31E-09	Under-expressed
MIA3	-0.81	6.25E-10	1.14E-08	Under-expressed
EMSY	-0.81	6.51E-10	1.18E-08	Under-expressed
NDRG2	-0.81	1.13E-08	1.36E-07	Under-expressed
KLHL2	-0.81	2.79E-08	2.99E-07	Under-expressed
PLEKHM3	-0.81	2.83E-08	3.02E-07	Under-expressed
APOL6	-0.81	1.66E-07	1.39E-06	Under-expressed
MPHOSPH9	-0.81	1.76E-07	1.46E-06	Under-expressed
MORC3	-0.81	2.28E-07	1.80E-06	Under-expressed
ARL5B	-0.81	2.64E-07	2.04E-06	Under-expressed
STARD8	-0.81	3.05E-07	2.31E-06	Under-expressed
KLF13	-0.81	3.20E-07	2.42E-06	Under-expressed
ALAS1	-0.81	3.56E-07	2.64E-06	Under-expressed
GLCCI1	-0.81	4.52E-06	2.35E-05	Under-expressed
SIAE	-0.81	8.69E-06	4.16E-05	Under-expressed
CASP8AP2	-0.81	2.10E-05	8.97E-05	Under-expressed
FRK	-0.81	2.77E-05	1.14E-04	Under-expressed
CD82	-0.81	4.45E-05	1.73E-04	Under-expressed
CPNE8	-0.81	8.71E-05	3.11E-04	Under-expressed
MDFIC	-0.81	1.75E-04	5.72E-04	Under-expressed
1-Mar	-0.81	2.38E-04	7.47E-04	Under-expressed
PLXDC2	-0.81	5.04E-04	1.44E-03	Under-expressed
AMY2B	-0.81	5.63E-04	1.58E-03	Under-expressed
DOCK10	-0.81	7.87E-04	2.12E-03	Under-expressed
MAOB	-0.81	9.89E-04	2.58E-03	Under-expressed
TMPRSS2	-0.81	6.26E-03	0.0129	Under-expressed
CIPC	-0.82	1.25E-10	2.98E-09	Under-expressed
PIK3R3	-0.82	1.52E-10	3.50E-09	Under-expressed
DTWD2	-0.82	3.48E-10	7.04E-09	Under-expressed
CARF	-0.82	1.93E-07	1.56E-06	Under-expressed
SUGT1P3	-0.82	2.72E-07	2.09E-06	Under-expressed
LRRC58	-0.82	3.71E-07	2.73E-06	Under-expressed
EFNB2	-0.82	9.17E-07	5.92E-06	Under-expressed
SLC25A20	-0.82	1.78E-06	1.04E-05	Under-expressed
CD302	-0.82	3.18E-06	1.72E-05	Under-expressed
SPDYE3	-0.82	4.49E-06	2.34E-05	Under-expressed
RIPOR2	-0.82	8.63E-06	4.14E-05	Under-expressed
DNAJC22	-0.82	1.50E-05	6.67E-05	Under-expressed

KANK1	-0.82	5.85E-05	2.19E-04	Under-expressed
FASN	-0.82	8.48E-05	3.04E-04	Under-expressed
CELSR2	-0.82	1.31E-04	4.44E-04	Under-expressed
LINC00526	-0.82	2.11E-04	6.72E-04	Under-expressed
HSPA4L	-0.82	3.62E-04	1.08E-03	Under-expressed
CD55	-0.82	5.49E-04	1.54E-03	Under-expressed
SRGAP1	-0.82	1.83E-03	4.40E-03	Under-expressed
DBT	-0.83	1.28E-13	8.18E-12	Under-expressed
SH2B3	-0.83	2.07E-12	8.78E-11	Under-expressed
LTN1	-0.83	4.06E-07	2.94E-06	Under-expressed
LPIN2	-0.83	6.68E-07	4.54E-06	Under-expressed
RPS6KA2	-0.83	1.71E-06	1.01E-05	Under-expressed
LPL	-0.83	2.29E-06	1.29E-05	Under-expressed
WNT3	-0.83	2.68E-06	1.49E-05	Under-expressed
PHYH	-0.83	1.79E-05	7.79E-05	Under-expressed
PDPR	-0.83	2.00E-05	8.57E-05	Under-expressed
MINDY2	-0.83	2.83E-05	1.16E-04	Under-expressed
CAP2	-0.83	3.40E-05	1.37E-04	Under-expressed
MRC1	-0.83	2.71E-04	8.36E-04	Under-expressed
CD14	-0.83	3.58E-04	1.07E-03	Under-expressed
GK3P	-0.83	5.88E-04	1.63E-03	Under-expressed
HERC5	-0.83	9.78E-04	2.55E-03	Under-expressed
TMEM30B	-0.83	1.97E-03	4.69E-03	Under-expressed
SLC16A11	-0.83	0.0159	0.0293	Under-expressed
ZYG11B	-0.84	7.63E-14	5.20E-12	Under-expressed
RESF1	-0.84	5.15E-11	1.43E-09	Under-expressed
FLT4	-0.84	1.03E-08	1.25E-07	Under-expressed
UEVLD	-0.84	3.39E-08	3.54E-07	Under-expressed
MEF2A	-0.84	4.45E-08	4.45E-07	Under-expressed
DYSF	-0.84	8.14E-08	7.48E-07	Under-expressed
SLC2A13	-0.84	1.03E-07	9.19E-07	Under-expressed
ABCA5	-0.84	1.91E-07	1.56E-06	Under-expressed
IRS1	-0.84	2.93E-07	2.23E-06	Under-expressed
KLHL28	-0.84	5.53E-07	3.85E-06	Under-expressed
TRANK1	-0.84	7.30E-07	4.88E-06	Under-expressed
CLUHP3	-0.84	2.88E-06	1.58E-05	Under-expressed
CYB5A	-0.84	4.08E-06	2.15E-05	Under-expressed
ZFYVE28	-0.84	4.14E-06	2.18E-05	Under-expressed
THBD	-0.84	1.05E-05	4.91E-05	Under-expressed
RNASEL	-0.84	2.53E-05	1.05E-04	Under-expressed
FILIP1L	-0.84	2.88E-05	1.18E-04	Under-expressed
CADM1	-0.84	4.23E-05	1.65E-04	Under-expressed
NFASC	-0.84	6.61E-05	2.43E-04	Under-expressed
PKD2	-0.84	6.97E-05	2.55E-04	Under-expressed
GJA5	-0.84	7.66E-05	2.77E-04	Under-expressed
GIMAP2	-0.84	1.38E-04	4.64E-04	Under-expressed
NTN4	-0.84	2.79E-04	8.58E-04	Under-expressed
CA13	-0.84	3.23E-04	9.75E-04	Under-expressed
CLEC3B	-0.84	8.77E-04	2.33E-03	Under-expressed
GJB2	-0.84	1.06E-03	2.73E-03	Under-expressed
SPTBN2	-0.84	1.84E-03	4.41E-03	Under-expressed
COL14A1	-0.84	2.49E-03	5.78E-03	Under-expressed
HLF	-0.84	4.00E-03	8.75E-03	Under-expressed
DEPDC7	-0.84	4.05E-03	8.84E-03	Under-expressed
MGST1	-0.84	8.29E-03	0.0166	Under-expressed
FMO5	-0.84	9.79E-03	0.0192	Under-expressed
OCIAD2	-0.84	0.0106	0.0205	Under-expressed

KANK2	-0.85	1.73E-10	3.88E-09	Under-expressed
HIPK1	-0.85	2.75E-10	5.78E-09	Under-expressed
EXOC3L2	-0.85	1.11E-08	1.35E-07	Under-expressed
SHROOM4	-0.85	9.14E-08	8.30E-07	Under-expressed
CDC42BPA	-0.85	1.58E-07	1.34E-06	Under-expressed
CLCN3	-0.85	1.77E-07	1.47E-06	Under-expressed
KIAA1109	-0.85	2.45E-07	1.92E-06	Under-expressed
SRD5A1	-0.85	3.72E-07	2.73E-06	Under-expressed
TMCC3	-0.85	4.20E-07	3.04E-06	Under-expressed
APOLD1	-0.85	4.57E-07	3.27E-06	Under-expressed
BTN3A3	-0.85	7.02E-07	4.73E-06	Under-expressed
SKIL	-0.85	1.30E-05	5.92E-05	Under-expressed
LOC729603	-0.85	4.16E-05	1.63E-04	Under-expressed
CRYBG1	-0.85	1.39E-04	4.68E-04	Under-expressed
CMAHP	-0.85	4.18E-04	1.22E-03	Under-expressed
PLA1A	-0.85	5.50E-04	1.54E-03	Under-expressed
AGL	-0.86	1.61E-12	7.20E-11	Under-expressed
CPT2	-0.86	3.17E-11	9.36E-10	Under-expressed
ELOVL5	-0.86	1.70E-10	3.83E-09	Under-expressed
PDPK1	-0.86	1.14E-08	1.38E-07	Under-expressed
PIK3CA	-0.86	4.48E-08	4.46E-07	Under-expressed
TMEM38B	-0.86	5.73E-08	5.50E-07	Under-expressed
SH3BP5	-0.86	2.37E-07	1.86E-06	Under-expressed
RSRC1	-0.86	1.74E-05	7.59E-05	Under-expressed
DYRK3	-0.86	3.62E-05	1.44E-04	Under-expressed
ADAMTS1	-0.86	5.47E-05	2.07E-04	Under-expressed
DDX21	-0.86	6.60E-04	1.82E-03	Under-expressed
TFPI	-0.86	4.86E-03	0.0104	Under-expressed
LAMC3	-0.86	0.0125	0.0237	Under-expressed
NIPSNAP3A	-0.87	5.30E-10	1.01E-08	Under-expressed
HEATR5A	-0.87	7.13E-10	1.28E-08	Under-expressed
PEX1	-0.87	8.25E-10	1.46E-08	Under-expressed
ITGA1	-0.87	1.00E-09	1.72E-08	Under-expressed
ERG	-0.87	8.07E-09	1.02E-07	Under-expressed
TLR4	-0.87	1.84E-08	2.09E-07	Under-expressed
HPS5	-0.87	9.80E-08	8.79E-07	Under-expressed
KLF10	-0.87	2.46E-07	1.92E-06	Under-expressed
GIMAP6	-0.87	6.19E-07	4.24E-06	Under-expressed
CSRP2	-0.87	2.39E-06	1.35E-05	Under-expressed
RNFT1	-0.87	4.57E-06	2.37E-05	Under-expressed
ANO8	-0.87	1.69E-05	7.41E-05	Under-expressed
CACNA1C	-0.87	2.81E-05	1.16E-04	Under-expressed
EYA3	-0.87	1.04E-04	3.62E-04	Under-expressed
GUCY1A1	-0.87	1.04E-04	3.63E-04	Under-expressed
THSD1	-0.87	1.16E-04	4.01E-04	Under-expressed
ABI3BP	-0.87	3.69E-04	1.10E-03	Under-expressed
SLC16A14	-0.87	5.42E-04	1.53E-03	Under-expressed
GMPR	-0.87	1.98E-03	4.73E-03	Under-expressed
GC	-0.87	3.27E-03	7.34E-03	Under-expressed
OSGIN1	-0.87	5.47E-03	0.0115	Under-expressed
ADH6	-0.87	0.0158	0.029	Under-expressed
ERCC4	-0.88	1.62E-12	7.20E-11	Under-expressed
AOC3	-0.88	2.04E-08	2.28E-07	Under-expressed
TXNDC16	-0.88	2.58E-08	2.79E-07	Under-expressed
ARHGEF6	-0.88	1.03E-07	9.18E-07	Under-expressed
BOD1L1	-0.88	3.65E-07	2.69E-06	Under-expressed
SCPEP1	-0.88	4.37E-06	2.29E-05	Under-expressed

PDZD2	-0.88	1.39E-05	6.25E-05	Under-expressed
CHN2	-0.88	5.10E-05	1.94E-04	Under-expressed
VWF	-0.88	1.30E-04	4.43E-04	Under-expressed
HCFC2	-0.89	1.80E-13	1.12E-11	Under-expressed
SEPSECS	-0.89	3.15E-11	9.33E-10	Under-expressed
ATP11B	-0.89	4.78E-10	9.23E-09	Under-expressed
TRPM7	-0.89	1.11E-09	1.88E-08	Under-expressed
LNPEP	-0.89	1.34E-08	1.59E-07	Under-expressed
ITGA7	-0.89	2.26E-08	2.49E-07	Under-expressed
PAXIP1-AS2	-0.89	2.96E-08	3.16E-07	Under-expressed
TIE1	-0.89	5.52E-08	5.35E-07	Under-expressed
FAM107A	-0.89	2.35E-06	1.33E-05	Under-expressed
PACSIN2	-0.89	3.07E-06	1.67E-05	Under-expressed
KDM8	-0.89	6.81E-06	3.35E-05	Under-expressed
PMEL	-0.89	1.14E-05	5.25E-05	Under-expressed
SEMA3G	-0.89	2.67E-05	1.11E-04	Under-expressed
RAMP3	-0.89	4.30E-04	1.25E-03	Under-expressed
GPT2	-0.89	5.41E-04	1.53E-03	Under-expressed
ADSSL1	-0.89	5.47E-04	1.54E-03	Under-expressed
SYBU	-0.89	1.17E-03	2.97E-03	Under-expressed
EPHA1	-0.89	4.57E-03	9.84E-03	Under-expressed
RGN	-0.89	5.59E-03	0.0117	Under-expressed
PCOLCE2	-0.89	9.00E-03	0.0178	Under-expressed
PCYOX1	-0.9	4.66E-13	2.40E-11	Under-expressed
IPP	-0.9	4.86E-10	9.36E-09	Under-expressed
ZC3H13	-0.9	1.07E-09	1.82E-08	Under-expressed
ADGRF5	-0.9	1.33E-09	2.18E-08	Under-expressed
CYYR1	-0.9	9.39E-08	8.48E-07	Under-expressed
SYNE2	-0.9	1.90E-07	1.55E-06	Under-expressed
MAGI2	-0.9	6.68E-07	4.54E-06	Under-expressed
NAP1L5	-0.9	6.82E-07	4.62E-06	Under-expressed
RAB30	-0.9	7.07E-07	4.76E-06	Under-expressed
CCDC146	-0.9	7.16E-07	4.81E-06	Under-expressed
CYP4V2	-0.9	1.05E-06	6.65E-06	Under-expressed
VPS35L	-0.9	1.65E-06	9.77E-06	Under-expressed
RNF169	-0.9	6.19E-06	3.09E-05	Under-expressed
STX1B	-0.9	1.32E-05	5.99E-05	Under-expressed
ZNF420	-0.9	2.03E-05	8.68E-05	Under-expressed
SNX12	-0.9	3.49E-05	1.40E-04	Under-expressed
RAI2	-0.9	4.77E-05	1.83E-04	Under-expressed
SEPTIN4	-0.9	1.06E-04	3.68E-04	Under-expressed
FBN1	-0.9	1.20E-04	4.13E-04	Under-expressed
SLIT3	-0.9	2.38E-04	7.46E-04	Under-expressed
CTAGE15	-0.9	1.25E-03	3.15E-03	Under-expressed
WASF3	-0.9	0.0168	0.0308	Under-expressed
APOH	-0.9	0.0172	0.0313	Under-expressed
ATP13A3	-0.91	1.68E-11	5.34E-10	Under-expressed
SBF2	-0.91	1.29E-07	1.12E-06	Under-expressed
HHAT	-0.91	1.78E-07	1.47E-06	Under-expressed
CREG1	-0.91	3.76E-07	2.76E-06	Under-expressed
EMCN	-0.91	1.42E-06	8.57E-06	Under-expressed
NAMPT	-0.91	2.64E-06	1.47E-05	Under-expressed
MAOA	-0.91	5.42E-06	2.75E-05	Under-expressed
CD1D	-0.91	1.33E-05	6.01E-05	Under-expressed
IFIT1	-0.91	4.73E-05	1.82E-04	Under-expressed
S1PR3	-0.91	1.78E-04	5.81E-04	Under-expressed
LOC730101	-0.91	2.24E-04	7.09E-04	Under-expressed

OASL	-0.91	3.06E-04	9.33E-04	Under-expressed
TMEM220	-0.91	1.05E-03	2.72E-03	Under-expressed
ITIH1	-0.91	0.0143	0.0266	Under-expressed
SLC31A1	-0.92	8.15E-13	3.90E-11	Under-expressed
SMIM14	-0.92	7.46E-10	1.33E-08	Under-expressed
NFIA	-0.92	1.37E-09	2.23E-08	Under-expressed
FZD4	-0.92	1.45E-09	2.34E-08	Under-expressed
POLK	-0.92	3.65E-09	5.18E-08	Under-expressed
GBE1	-0.92	3.93E-09	5.53E-08	Under-expressed
ALDH5A1	-0.92	2.33E-07	1.83E-06	Under-expressed
BHLHE40	-0.92	4.60E-07	3.28E-06	Under-expressed
SLC9B2	-0.92	2.44E-05	1.02E-04	Under-expressed
RASGRF2	-0.92	3.80E-05	1.51E-04	Under-expressed
ABCC6	-0.92	5.19E-05	1.97E-04	Under-expressed
GCSH	-0.92	9.03E-05	3.20E-04	Under-expressed
TCIM	-0.92	3.21E-04	9.73E-04	Under-expressed
STRIP2	-0.92	1.01E-03	2.61E-03	Under-expressed
ADAMTSL2	-0.92	0.0104	0.0203	Under-expressed
AHCYL2	-0.93	7.14E-12	2.55E-10	Under-expressed
MAN1A2	-0.93	1.19E-11	3.95E-10	Under-expressed
L2HGDH	-0.93	2.82E-11	8.48E-10	Under-expressed
TSHZ1	-0.93	3.46E-11	1.01E-09	Under-expressed
SLC40A1	-0.93	6.94E-10	1.25E-08	Under-expressed
WDR44	-0.93	1.25E-09	2.07E-08	Under-expressed
ETS1	-0.93	2.04E-08	2.28E-07	Under-expressed
ACAT1	-0.93	2.73E-07	2.10E-06	Under-expressed
PTPRG	-0.93	5.85E-07	4.04E-06	Under-expressed
FAT1	-0.93	1.81E-06	1.06E-05	Under-expressed
USP2	-0.93	1.71E-05	7.48E-05	Under-expressed
PDE7B	-0.93	1.81E-05	7.85E-05	Under-expressed
ADHFE1	-0.93	2.53E-04	7.88E-04	Under-expressed
SLC2A12	-0.93	3.62E-04	1.08E-03	Under-expressed
MYH11	-0.93	7.69E-04	2.07E-03	Under-expressed
NIPAL2	-0.93	8.68E-04	2.30E-03	Under-expressed
TF	-0.93	0.0241	0.0423	Under-expressed
ALDH9A1	-0.94	1.25E-15	1.21E-13	Under-expressed
OSBPL11	-0.94	2.47E-15	2.27E-13	Under-expressed
USF3	-0.94	5.32E-10	1.01E-08	Under-expressed
PLEKHG1	-0.94	2.52E-09	3.77E-08	Under-expressed
KLHL26	-0.94	6.10E-08	5.82E-07	Under-expressed
FRMD4B	-0.94	1.16E-07	1.01E-06	Under-expressed
ERO1B	-0.94	1.58E-07	1.33E-06	Under-expressed
HIVEP1	-0.94	4.26E-07	3.07E-06	Under-expressed
GNG12	-0.94	5.04E-07	3.55E-06	Under-expressed
ZNF425	-0.94	9.55E-07	6.13E-06	Under-expressed
ARHGAP24	-0.94	1.45E-05	6.45E-05	Under-expressed
MYSM1	-0.94	1.74E-05	7.60E-05	Under-expressed
VSTM4	-0.94	1.21E-04	4.13E-04	Under-expressed
SH3RF2	-0.94	4.60E-03	9.90E-03	Under-expressed
TMEM192	-0.95	3.52E-13	1.89E-11	Under-expressed
DCUN1D1	-0.95	3.51E-10	7.08E-09	Under-expressed
NBEAL1	-0.95	2.65E-08	2.86E-07	Under-expressed
MYCL	-0.95	1.65E-06	9.77E-06	Under-expressed
GIMAP7	-0.95	2.00E-06	1.15E-05	Under-expressed
SARDH	-0.95	1.24E-03	3.13E-03	Under-expressed
RBP5	-0.95	5.53E-03	0.0116	Under-expressed
MMRN2	-0.96	1.66E-10	3.76E-09	Under-expressed

PDE4DIP	-0.96	1.78E-10	3.96E-09	Under-expressed
SCYL2	-0.96	1.46E-09	2.36E-08	Under-expressed
ETFBKMT	-0.96	5.75E-09	7.65E-08	Under-expressed
REV3L	-0.96	2.59E-08	2.80E-07	Under-expressed
DPP8	-0.96	9.33E-08	8.43E-07	Under-expressed
TSC22D2	-0.96	1.17E-07	1.02E-06	Under-expressed
CEP97	-0.96	2.36E-07	1.85E-06	Under-expressed
METTL14	-0.96	2.55E-07	1.98E-06	Under-expressed
SYNE3	-0.96	6.79E-07	4.60E-06	Under-expressed
PCSK6	-0.96	2.18E-06	1.24E-05	Under-expressed
SATB1	-0.96	4.54E-05	1.75E-04	Under-expressed
PBLD	-0.96	5.58E-05	2.10E-04	Under-expressed
MAST4	-0.96	7.58E-05	2.75E-04	Under-expressed
AMOTL1	-0.96	1.77E-04	5.79E-04	Under-expressed
CGN	-0.96	2.06E-04	6.61E-04	Under-expressed
NOMO2	-0.96	2.60E-04	8.08E-04	Under-expressed
DPY19L3	-0.96	2.93E-04	8.95E-04	Under-expressed
NAGS	-0.96	2.69E-03	6.19E-03	Under-expressed
ITSN1	-0.97	3.17E-13	1.76E-11	Under-expressed
PGM2	-0.97	9.72E-11	2.41E-09	Under-expressed
TBC1D8B	-0.97	1.36E-09	2.23E-08	Under-expressed
NOS3	-0.97	1.16E-07	1.01E-06	Under-expressed
SLC39A8	-0.97	1.16E-07	1.01E-06	Under-expressed
DPYD	-0.97	4.22E-06	2.22E-05	Under-expressed
TGFBR3	-0.97	5.53E-06	2.80E-05	Under-expressed
LATS1	-0.97	7.35E-06	3.59E-05	Under-expressed
MNS1	-0.97	7.87E-06	3.81E-05	Under-expressed
LRCH3	-0.97	1.44E-05	6.44E-05	Under-expressed
RIF1	-0.97	2.03E-05	8.69E-05	Under-expressed
CAVIN2	-0.97	7.21E-05	2.63E-04	Under-expressed
CLIC5	-0.97	9.72E-05	3.42E-04	Under-expressed
DGAT2	-0.97	7.05E-04	1.92E-03	Under-expressed
KMO	-0.97	3.14E-03	7.10E-03	Under-expressed
SLC38A3	-0.97	5.38E-03	0.0113	Under-expressed
ITIH3	-0.97	0.0122	0.0231	Under-expressed
LYST	-0.98	5.70E-11	1.54E-09	Under-expressed
NEURL1B	-0.98	3.94E-10	7.84E-09	Under-expressed
TRIM35	-0.98	4.92E-09	6.70E-08	Under-expressed
MICAL2	-0.98	1.97E-07	1.60E-06	Under-expressed
IFIT2	-0.98	8.42E-07	5.50E-06	Under-expressed
FKBP5	-0.98	8.77E-07	5.71E-06	Under-expressed
CCDC186	-0.98	1.07E-06	6.76E-06	Under-expressed
ST6GAL1	-0.98	2.35E-06	1.33E-05	Under-expressed
DOCK1	-0.98	5.34E-05	2.02E-04	Under-expressed
COL15A1	-0.98	6.46E-05	2.38E-04	Under-expressed
FBXO27	-0.98	3.57E-04	1.07E-03	Under-expressed
IGFBP5	-0.98	4.05E-04	1.19E-03	Under-expressed
PLIN4	-0.98	6.91E-04	1.89E-03	Under-expressed
ABCG2	-0.98	7.46E-04	2.02E-03	Under-expressed
UGP2	-0.99	3.38E-13	1.83E-11	Under-expressed
MEGF9	-0.99	3.43E-11	1.01E-09	Under-expressed
SAR1B	-0.99	1.22E-10	2.90E-09	Under-expressed
PPARA	-0.99	4.59E-10	8.93E-09	Under-expressed
SYNE1	-0.99	3.60E-09	5.12E-08	Under-expressed
KIF13A	-0.99	1.68E-07	1.40E-06	Under-expressed
PROS1	-0.99	5.70E-07	3.95E-06	Under-expressed
PAFAH1B2	-0.99	1.16E-06	7.23E-06	Under-expressed

TUT4	-0.99	2.01E-05	8.63E-05	Under-expressed
TFR2	-0.99	1.06E-03	2.73E-03	Under-expressed
TM6SF2	-0.99	1.21E-03	3.06E-03	Under-expressed
EHBP1	-1	6.74E-17	9.13E-15	Under-expressed
DYNLL2	-1	2.52E-11	7.66E-10	Under-expressed
FAM214A	-1	4.85E-11	1.35E-09	Under-expressed
GASK1B	-1	4.46E-08	4.45E-07	Under-expressed
TTBK2	-1	4.66E-08	4.62E-07	Under-expressed
ABHD15	-1	1.05E-07	9.27E-07	Under-expressed
ABHD6	-1	4.39E-07	3.15E-06	Under-expressed
GMCL1	-1	9.62E-06	4.54E-05	Under-expressed
ELOVL6	-1	3.35E-05	1.35E-04	Under-expressed
PON3	-1	7.59E-05	2.75E-04	Under-expressed
PDP2	-1.01	3.32E-13	1.82E-11	Under-expressed
SASH1	-1.01	1.57E-12	7.08E-11	Under-expressed
TRAPPC8	-1.01	5.58E-10	1.05E-08	Under-expressed
C9ORF72	-1.01	6.08E-10	1.12E-08	Under-expressed
GJD3	-1.01	2.70E-08	2.91E-07	Under-expressed
TXNIP	-1.01	5.41E-08	5.27E-07	Under-expressed
MYO18A	-1.01	5.97E-08	5.71E-07	Under-expressed
2-Mar	-1.01	2.65E-07	2.05E-06	Under-expressed
ADAM10	-1.01	7.53E-07	5.01E-06	Under-expressed
RGS5	-1.01	2.97E-06	1.63E-05	Under-expressed
ADGRL2	-1.01	1.03E-05	4.81E-05	Under-expressed
CFH	-1.01	3.49E-04	1.04E-03	Under-expressed
COL4A4	-1.01	5.96E-04	1.65E-03	Under-expressed
SERPINA5	-1.01	7.80E-04	2.10E-03	Under-expressed
TTC9	-1.01	8.07E-04	2.17E-03	Under-expressed
CD36	-1.01	1.18E-03	3.00E-03	Under-expressed
GIPC2	-1.01	1.23E-03	3.10E-03	Under-expressed
ABCC2	-1.01	4.46E-03	9.63E-03	Under-expressed
F12	-1.01	9.86E-03	0.0193	Under-expressed
MYO1B	-1.02	1.88E-12	8.25E-11	Under-expressed
FRY	-1.02	3.68E-09	5.22E-08	Under-expressed
TRIM56	-1.02	1.80E-07	1.48E-06	Under-expressed
ARHGEF26	-1.02	8.79E-06	4.20E-05	Under-expressed
OPHN1	-1.02	1.38E-05	6.22E-05	Under-expressed
CDH23	-1.02	2.13E-05	9.07E-05	Under-expressed
CCNI	-1.02	7.53E-05	2.73E-04	Under-expressed
INHBE	-1.02	1.13E-03	2.89E-03	Under-expressed
F2	-1.02	8.09E-03	0.0162	Under-expressed
ALB	-1.02	0.0121	0.0231	Under-expressed
SLC35D1	-1.03	6.00E-14	4.18E-12	Under-expressed
ABHD2	-1.03	8.76E-11	2.20E-09	Under-expressed
IQGAP2	-1.03	1.77E-10	3.94E-09	Under-expressed
RMDN2	-1.03	5.55E-10	1.04E-08	Under-expressed
ALDH2	-1.03	1.76E-08	2.01E-07	Under-expressed
TMEM133	-1.03	7.30E-08	6.80E-07	Under-expressed
TMEM204	-1.03	2.25E-07	1.79E-06	Under-expressed
EMP1	-1.03	8.13E-07	5.35E-06	Under-expressed
FGD4	-1.03	2.19E-06	1.25E-05	Under-expressed
FBLN7	-1.03	3.04E-06	1.66E-05	Under-expressed
SGCB	-1.03	8.75E-05	3.12E-04	Under-expressed
FNDC4	-1.03	1.64E-04	5.38E-04	Under-expressed
MBNL3	-1.03	5.29E-04	1.50E-03	Under-expressed
KLB	-1.03	3.19E-03	7.19E-03	Under-expressed
HSD17B4	-1.04	2.37E-10	5.08E-09	Under-expressed

SYNPO2	-1.04	6.25E-09	8.22E-08	Under-expressed
SATB2	-1.04	4.84E-07	3.43E-06	Under-expressed
CBLN3	-1.04	5.01E-07	3.54E-06	Under-expressed
OLFM2	-1.04	7.43E-06	3.62E-05	Under-expressed
PALMD	-1.04	1.99E-05	8.54E-05	Under-expressed
ETNK2	-1.04	6.26E-04	1.73E-03	Under-expressed
APOB	-1.04	1.11E-03	2.84E-03	Under-expressed
SETD7	-1.05	5.19E-13	2.63E-11	Under-expressed
ZNF791	-1.05	4.15E-08	4.20E-07	Under-expressed
PEAR1	-1.05	1.06E-07	9.40E-07	Under-expressed
KCTD20	-1.05	5.12E-07	3.60E-06	Under-expressed
SEMA6D	-1.05	2.18E-05	9.27E-05	Under-expressed
IQCN	-1.05	4.85E-05	1.86E-04	Under-expressed
ARHGEF37	-1.05	1.13E-04	3.91E-04	Under-expressed
KLKB1	-1.05	5.75E-04	1.61E-03	Under-expressed
FGF2	-1.05	1.48E-03	3.65E-03	Under-expressed
VWA8	-1.06	8.13E-13	3.90E-11	Under-expressed
RAPGEF2	-1.06	5.13E-12	1.91E-10	Under-expressed
HIPK2	-1.06	6.43E-12	2.33E-10	Under-expressed
CPEB2	-1.06	5.44E-11	1.49E-09	Under-expressed
TOM1L1	-1.06	2.00E-10	4.35E-09	Under-expressed
CTSO	-1.06	3.23E-09	4.67E-08	Under-expressed
NBPF11	-1.06	6.82E-09	8.86E-08	Under-expressed
ST6GALNAC3	-1.06	1.51E-07	1.29E-06	Under-expressed
SLCO2B1	-1.06	1.23E-06	7.60E-06	Under-expressed
RHOBTB3	-1.06	2.19E-06	1.25E-05	Under-expressed
TSC22D3	-1.06	2.38E-06	1.34E-05	Under-expressed
C1S	-1.06	2.50E-06	1.40E-05	Under-expressed
KCNJ8	-1.06	5.29E-06	2.69E-05	Under-expressed
LTBP1	-1.06	6.91E-05	2.53E-04	Under-expressed
GLUD2	-1.06	5.31E-04	1.50E-03	Under-expressed
ARNTL	-1.07	2.20E-14	1.71E-12	Under-expressed
KLHL20	-1.07	6.28E-14	4.32E-12	Under-expressed
DHRS4-AS1	-1.07	4.13E-13	2.14E-11	Under-expressed
RAPH1	-1.07	3.58E-12	1.39E-10	Under-expressed
NNT	-1.07	3.30E-10	6.73E-09	Under-expressed
MAFB	-1.07	4.58E-10	8.93E-09	Under-expressed
ITPR1	-1.07	1.22E-08	1.46E-07	Under-expressed
RBM43	-1.07	7.27E-08	6.78E-07	Under-expressed
LMOD1	-1.07	3.71E-07	2.73E-06	Under-expressed
NRN1	-1.07	1.68E-05	7.36E-05	Under-expressed
FCN3	-1.07	1.62E-03	3.94E-03	Under-expressed
CPEB4	-1.08	6.58E-10	1.19E-08	Under-expressed
PRKAG2	-1.08	6.68E-10	1.21E-08	Under-expressed
DOCK9	-1.08	8.49E-10	1.49E-08	Under-expressed
HECW2	-1.08	8.53E-10	1.50E-08	Under-expressed
ZNF619	-1.08	2.49E-09	3.72E-08	Under-expressed
SORD	-1.08	7.38E-07	4.93E-06	Under-expressed
TRIM22	-1.08	1.16E-06	7.24E-06	Under-expressed
PDE3B	-1.08	1.99E-06	1.15E-05	Under-expressed
HTR2B	-1.08	2.21E-05	9.36E-05	Under-expressed
ALDH1A1	-1.08	2.44E-04	7.63E-04	Under-expressed
SPATA41	-1.08	5.40E-04	1.52E-03	Under-expressed
CYP2D7	-1.08	4.51E-03	9.73E-03	Under-expressed
SEC24D	-1.09	5.99E-11	1.59E-09	Under-expressed
SECISBP2L	-1.09	1.50E-09	2.41E-08	Under-expressed
TOB1	-1.09	6.78E-09	8.82E-08	Under-expressed

FAM13A	-1.09	3.14E-07	2.37E-06	Under-expressed
EPB41L5	-1.09	5.97E-07	4.11E-06	Under-expressed
MMRN1	-1.09	1.86E-06	1.08E-05	Under-expressed
DAAM1	-1.09	3.01E-06	1.65E-05	Under-expressed
FOLH1	-1.09	1.22E-05	5.60E-05	Under-expressed
ADCY1	-1.09	1.77E-03	4.28E-03	Under-expressed
SLC6A12	-1.09	3.18E-03	7.17E-03	Under-expressed
CYP2D6	-1.09	0.02	0.0358	Under-expressed
PGRMC1	-1.1	3.74E-13	1.98E-11	Under-expressed
GADD45A	-1.1	3.75E-11	1.08E-09	Under-expressed
MAP3K2	-1.1	4.48E-11	1.25E-09	Under-expressed
ABTB2	-1.1	9.82E-11	2.43E-09	Under-expressed
PRKCE	-1.1	3.05E-10	6.28E-09	Under-expressed
MTHFD1	-1.1	2.10E-09	3.21E-08	Under-expressed
FHL1	-1.1	7.89E-09	1.00E-07	Under-expressed
MYORG	-1.1	1.06E-08	1.29E-07	Under-expressed
ARL13B	-1.1	1.30E-08	1.55E-07	Under-expressed
SEC16B	-1.1	1.58E-07	1.34E-06	Under-expressed
STARD5	-1.1	6.13E-07	4.21E-06	Under-expressed
CYBRD1	-1.1	1.28E-06	7.86E-06	Under-expressed
PCDH17	-1.1	1.35E-06	8.25E-06	Under-expressed
MYO16	-1.1	3.52E-05	1.41E-04	Under-expressed
TBX15	-1.1	2.57E-04	7.99E-04	Under-expressed
SLC30A1	-1.11	3.81E-11	1.09E-09	Under-expressed
TBCEL	-1.11	5.23E-11	1.45E-09	Under-expressed
BROX	-1.11	6.19E-08	5.88E-07	Under-expressed
TNPO3	-1.11	3.19E-07	2.41E-06	Under-expressed
ACADSB	-1.11	5.65E-07	3.92E-06	Under-expressed
ZNF521	-1.11	7.71E-07	5.11E-06	Under-expressed
RP2	-1.11	1.40E-06	8.49E-06	Under-expressed
STAG1	-1.11	1.95E-06	1.13E-05	Under-expressed
TSKU	-1.11	1.54E-05	6.81E-05	Under-expressed
MAP7	-1.11	5.29E-05	2.01E-04	Under-expressed
FBLN5	-1.11	5.32E-05	2.02E-04	Under-expressed
ST6GALNAC2	-1.11	1.77E-04	5.78E-04	Under-expressed
MMUT	-1.12	2.01E-12	8.62E-11	Under-expressed
NEDD4	-1.12	6.98E-09	9.02E-08	Under-expressed
MAN2A1	-1.12	3.63E-08	3.77E-07	Under-expressed
EPHX2	-1.12	9.36E-08	8.46E-07	Under-expressed
LIN52	-1.12	3.02E-07	2.30E-06	Under-expressed
CDC14B	-1.12	4.13E-07	2.99E-06	Under-expressed
SETBP1	-1.12	8.28E-07	5.43E-06	Under-expressed
SLC7A2	-1.12	1.19E-05	5.45E-05	Under-expressed
IFNLR1	-1.12	1.90E-05	8.16E-05	Under-expressed
PI4K2B	-1.13	1.72E-17	2.77E-15	Under-expressed
SLC35A3	-1.13	1.13E-15	1.11E-13	Under-expressed
ABHD18	-1.13	1.84E-10	4.06E-09	Under-expressed
ERCC6L2	-1.13	1.08E-09	1.83E-08	Under-expressed
SORL1	-1.13	2.96E-08	3.16E-07	Under-expressed
RFTN1	-1.13	2.56E-07	1.98E-06	Under-expressed
SIRPB1	-1.13	1.62E-06	9.65E-06	Under-expressed
LDHD	-1.13	1.07E-04	3.71E-04	Under-expressed
AZGP1	-1.13	2.13E-03	5.05E-03	Under-expressed
TMPRSS6	-1.13	2.90E-03	6.61E-03	Under-expressed
RBP4	-1.13	3.12E-03	7.06E-03	Under-expressed
F7	-1.13	3.34E-03	7.48E-03	Under-expressed
PITPNM2	-1.14	1.99E-13	1.22E-11	Under-expressed

BCKDHB	-1.14	5.40E-12	1.99E-10	Under-expressed
ZKSCAN1	-1.14	1.04E-11	3.53E-10	Under-expressed
CDH5	-1.14	1.65E-11	5.30E-10	Under-expressed
LDB2	-1.14	3.07E-10	6.30E-09	Under-expressed
DDX60	-1.14	1.06E-07	9.40E-07	Under-expressed
DPP4	-1.14	1.64E-05	7.23E-05	Under-expressed
PNPLA3	-1.14	8.22E-05	2.95E-04	Under-expressed
AMDHD1	-1.14	9.25E-04	2.44E-03	Under-expressed
ZNF680	-1.15	1.17E-14	9.61E-13	Under-expressed
COBLL1	-1.15	5.98E-10	1.11E-08	Under-expressed
SC5D	-1.15	8.82E-10	1.54E-08	Under-expressed
EGFR	-1.15	1.07E-07	9.44E-07	Under-expressed
TLCD4	-1.15	1.09E-07	9.58E-07	Under-expressed
CACNB2	-1.15	1.01E-06	6.43E-06	Under-expressed
EFHD1	-1.15	3.63E-05	1.45E-04	Under-expressed
BDH1	-1.15	1.75E-04	5.74E-04	Under-expressed
PIPOX	-1.15	5.45E-04	1.53E-03	Under-expressed
ALDH1L1	-1.15	0.0137	0.0256	Under-expressed
ETFDH	-1.16	3.89E-16	4.25E-14	Under-expressed
MERTK	-1.16	1.81E-07	1.49E-06	Under-expressed
F5	-1.16	2.35E-05	9.89E-05	Under-expressed
RNF144B	-1.17	5.48E-13	2.76E-11	Under-expressed
ADGRA3	-1.17	3.02E-12	1.20E-10	Under-expressed
KLHL8	-1.17	2.62E-11	7.93E-10	Under-expressed
ACACB	-1.17	1.63E-08	1.88E-07	Under-expressed
STT3B	-1.17	2.30E-08	2.53E-07	Under-expressed
FAS	-1.17	2.33E-08	2.55E-07	Under-expressed
CGNL1	-1.17	1.49E-07	1.27E-06	Under-expressed
G0S2	-1.17	1.20E-04	4.11E-04	Under-expressed
CYP4A11	-1.17	5.07E-03	0.0108	Under-expressed
TTR	-1.17	8.05E-03	0.0162	Under-expressed
MTM1	-1.18	6.93E-12	2.48E-10	Under-expressed
SLC25A40	-1.18	3.35E-08	3.51E-07	Under-expressed
ATF2	-1.18	1.93E-07	1.56E-06	Under-expressed
SYTL4	-1.18	6.75E-07	4.58E-06	Under-expressed
MIR100HG	-1.18	2.41E-06	1.36E-05	Under-expressed
LEPR	-1.18	2.35E-04	7.40E-04	Under-expressed
ATE1	-1.19	9.98E-09	1.23E-07	Under-expressed
MASP2	-1.19	0.0121	0.0231	Under-expressed
ACADM	-1.2	5.69E-13	2.82E-11	Under-expressed
RBL2	-1.2	3.50E-12	1.37E-10	Under-expressed
S1PR1	-1.2	3.99E-11	1.13E-09	Under-expressed
ADAT1	-1.2	9.93E-11	2.45E-09	Under-expressed
MANEA	-1.2	1.33E-08	1.57E-07	Under-expressed
MAN1A1	-1.2	5.37E-08	5.25E-07	Under-expressed
DOCK5	-1.2	1.41E-07	1.21E-06	Under-expressed
ABCA10	-1.2	2.20E-05	9.32E-05	Under-expressed
TPPP	-1.2	8.20E-05	2.95E-04	Under-expressed
ELN	-1.2	1.77E-04	5.77E-04	Under-expressed
ALAD	-1.21	2.84E-13	1.64E-11	Under-expressed
ARL15	-1.21	9.92E-11	2.45E-09	Under-expressed
PLPP3	-1.21	1.20E-09	2.01E-08	Under-expressed
TAOK1	-1.21	1.22E-08	1.46E-07	Under-expressed
ST3GAL6	-1.21	6.03E-08	5.76E-07	Under-expressed
GTF2I	-1.21	1.39E-06	8.44E-06	Under-expressed
NR2C2	-1.21	5.03E-06	2.58E-05	Under-expressed
MAMDC4	-1.21	5.50E-06	2.79E-05	Under-expressed

RBBP5	-1.22	1.28E-10	3.04E-09	Under-expressed
TRIM2	-1.22	2.09E-10	4.54E-09	Under-expressed
TPI1P2	-1.22	2.50E-10	5.30E-09	Under-expressed
BAG4	-1.22	1.33E-08	1.57E-07	Under-expressed
CYP2U1	-1.22	2.76E-08	2.96E-07	Under-expressed
ZMAT1	-1.22	5.24E-07	3.67E-06	Under-expressed
FICD	-1.23	3.09E-13	1.74E-11	Under-expressed
RORA	-1.23	2.89E-12	1.16E-10	Under-expressed
CPEB3	-1.23	7.97E-11	2.04E-09	Under-expressed
TOR1AIP2	-1.23	7.88E-10	1.40E-08	Under-expressed
SAMHD1	-1.23	5.65E-09	7.52E-08	Under-expressed
SOX7	-1.23	1.33E-08	1.58E-07	Under-expressed
PRRG4	-1.23	1.36E-08	1.60E-07	Under-expressed
SELENOP	-1.23	3.06E-08	3.25E-07	Under-expressed
SLC16A13	-1.23	8.00E-08	7.37E-07	Under-expressed
KBTBD4	-1.23	3.25E-07	2.44E-06	Under-expressed
MATN2	-1.23	2.73E-06	1.51E-05	Under-expressed
THSD4	-1.23	2.92E-04	8.93E-04	Under-expressed
BAAT	-1.23	6.59E-04	1.81E-03	Under-expressed
ARHGAP42	-1.24	3.63E-11	1.05E-09	Under-expressed
PER3	-1.24	2.85E-10	5.94E-09	Under-expressed
SORBS2	-1.24	7.13E-08	6.67E-07	Under-expressed
CA2	-1.24	6.19E-07	4.24E-06	Under-expressed
PPL	-1.24	8.31E-06	3.99E-05	Under-expressed
EPHX1	-1.24	9.61E-06	4.54E-05	Under-expressed
PTPRB	-1.25	9.69E-10	1.67E-08	Under-expressed
CREBRF	-1.25	1.21E-08	1.45E-07	Under-expressed
PPP4R4	-1.25	1.90E-07	1.55E-06	Under-expressed
RUNDC3B	-1.25	7.90E-06	3.82E-05	Under-expressed
NTN1	-1.25	1.69E-05	7.42E-05	Under-expressed
SLC4A4	-1.25	4.55E-05	1.76E-04	Under-expressed
ANGPTL3	-1.25	2.95E-03	6.71E-03	Under-expressed
HEY2	-1.26	1.72E-10	3.87E-09	Under-expressed
SLC47A1	-1.26	1.63E-04	5.36E-04	Under-expressed
SERPIND1	-1.26	3.04E-03	6.89E-03	Under-expressed
MID2	-1.27	4.46E-08	4.45E-07	Under-expressed
PLSCR4	-1.27	8.09E-08	7.44E-07	Under-expressed
LMTK2	-1.27	1.50E-06	8.99E-06	Under-expressed
PCSK5	-1.27	6.36E-06	3.17E-05	Under-expressed
CYP2C9	-1.27	3.95E-03	8.65E-03	Under-expressed
EDNRB	-1.28	6.22E-11	1.64E-09	Under-expressed
TSTD2	-1.28	5.53E-08	5.36E-07	Under-expressed
LIMS1	-1.28	9.76E-06	4.60E-05	Under-expressed
PLCL2	-1.29	1.78E-09	2.78E-08	Under-expressed
DMD	-1.29	2.88E-07	2.20E-06	Under-expressed
FGGY	-1.29	3.11E-05	1.26E-04	Under-expressed
CLTRN	-1.29	8.87E-05	3.16E-04	Under-expressed
ANGPTL1	-1.29	2.52E-04	7.86E-04	Under-expressed
HPX	-1.29	1.83E-03	4.39E-03	Under-expressed
SLC1A2	-1.29	6.45E-03	0.0133	Under-expressed
CFHR3	-1.29	7.47E-03	0.0151	Under-expressed
SEMA5A	-1.3	1.60E-08	1.85E-07	Under-expressed
FILIP1	-1.3	1.67E-08	1.91E-07	Under-expressed
GRAMD1C	-1.3	1.95E-07	1.58E-06	Under-expressed
SELENBP1	-1.3	1.05E-06	6.69E-06	Under-expressed
PIEZO2	-1.3	2.21E-05	9.35E-05	Under-expressed
ATP2B2	-1.3	2.89E-04	8.85E-04	Under-expressed

CAT	-1.31	4.42E-14	3.19E-12	Under-expressed
CLOCK	-1.31	1.23E-09	2.05E-08	Under-expressed
SLC25A15	-1.31	1.30E-09	2.14E-08	Under-expressed
RREB1	-1.31	8.50E-09	1.07E-07	Under-expressed
MYO9A	-1.31	1.71E-07	1.43E-06	Under-expressed
PLCH2	-1.31	2.05E-04	6.56E-04	Under-expressed
MAT1A	-1.31	1.17E-03	2.97E-03	Under-expressed
ATP11C	-1.32	1.64E-14	1.31E-12	Under-expressed
HERC3	-1.32	2.87E-11	8.57E-10	Under-expressed
CES2	-1.32	2.65E-06	1.47E-05	Under-expressed
ADH1A	-1.32	2.71E-03	6.23E-03	Under-expressed
SLC25A34	-1.33	8.20E-09	1.03E-07	Under-expressed
MCC	-1.33	1.82E-07	1.50E-06	Under-expressed
KNL1	-1.33	1.98E-07	1.60E-06	Under-expressed
CDA	-1.33	8.61E-05	3.08E-04	Under-expressed
COL27A1	-1.33	1.81E-04	5.91E-04	Under-expressed
LONP2	-1.34	1.98E-19	8.24E-17	Under-expressed
APOOL	-1.34	3.52E-13	1.89E-11	Under-expressed
PCK2	-1.34	6.43E-08	6.09E-07	Under-expressed
FAM45BP	-1.34	7.37E-08	6.84E-07	Under-expressed
SHMT1	-1.34	1.31E-07	1.13E-06	Under-expressed
GPAM	-1.34	6.14E-05	2.28E-04	Under-expressed
SMARCA2	-1.35	5.31E-14	3.79E-12	Under-expressed
FAM124B	-1.35	5.92E-09	7.84E-08	Under-expressed
IL33	-1.35	1.20E-07	1.05E-06	Under-expressed
LRRC3	-1.35	3.42E-07	2.55E-06	Under-expressed
SLC27A2	-1.35	1.76E-06	1.03E-05	Under-expressed
FAM149A	-1.35	5.91E-06	2.97E-05	Under-expressed
PRG4	-1.35	1.30E-03	3.28E-03	Under-expressed
ENTPD5	-1.36	8.86E-13	4.22E-11	Under-expressed
GALNT15	-1.36	9.56E-09	1.18E-07	Under-expressed
CYP4X1	-1.36	2.26E-07	1.79E-06	Under-expressed
NBPF14	-1.36	6.08E-07	4.18E-06	Under-expressed
ROCK2	-1.36	9.75E-07	6.24E-06	Under-expressed
ARHGEF35	-1.36	3.72E-05	1.48E-04	Under-expressed
SLC27A5	-1.36	2.14E-04	6.82E-04	Under-expressed
FAM114A1	-1.37	1.92E-12	8.34E-11	Under-expressed
KLF15	-1.37	3.06E-06	1.67E-05	Under-expressed
HOMER2	-1.37	6.02E-05	2.25E-04	Under-expressed
KAT2B	-1.38	9.05E-16	8.94E-14	Under-expressed
DHTKD1	-1.38	2.06E-14	1.62E-12	Under-expressed
GNE	-1.38	8.89E-13	4.22E-11	Under-expressed
SLC51A	-1.38	3.31E-03	7.43E-03	Under-expressed
KLHDC10	-1.39	8.25E-14	5.59E-12	Under-expressed
CALCRL	-1.39	8.12E-11	2.07E-09	Under-expressed
PXK	-1.39	8.53E-09	1.07E-07	Under-expressed
ACOX2	-1.39	1.33E-05	6.01E-05	Under-expressed
LMBRD2	-1.4	8.26E-17	1.06E-14	Under-expressed
TRO	-1.4	2.95E-07	2.24E-06	Under-expressed
SNED1	-1.41	8.16E-17	1.06E-14	Under-expressed
ADAMTS17	-1.41	2.01E-05	8.60E-05	Under-expressed
ACKR2	-1.42	1.01E-05	4.73E-05	Under-expressed
RSC1A1	-1.43	2.64E-13	1.54E-11	Under-expressed
DAAM2	-1.43	1.23E-09	2.05E-08	Under-expressed
COL5A3	-1.43	2.22E-08	2.47E-07	Under-expressed
INSIG1	-1.43	4.27E-07	3.08E-06	Under-expressed
ZFHX4	-1.43	1.22E-06	7.53E-06	Under-expressed

TXLNG	-1.43	3.42E-06	1.84E-05	Under-expressed
ITIH4	-1.43	1.17E-04	4.04E-04	Under-expressed
AKR7A3	-1.43	7.28E-04	1.98E-03	Under-expressed
PDE2A	-1.44	8.66E-12	3.00E-10	Under-expressed
BTNL9	-1.44	4.08E-09	5.72E-08	Under-expressed
NR3C2	-1.46	3.73E-12	1.44E-10	Under-expressed
CFHR1	-1.46	2.68E-03	6.17E-03	Under-expressed
PIK3R1	-1.47	1.70E-16	2.09E-14	Under-expressed
TLR3	-1.47	2.83E-09	4.18E-08	Under-expressed
SLC38A7	-1.48	4.86E-10	9.36E-09	Under-expressed
FAM168A	-1.48	4.21E-09	5.85E-08	Under-expressed
SHPRH	-1.48	3.25E-07	2.44E-06	Under-expressed
PPP1R3B	-1.49	1.07E-14	8.86E-13	Under-expressed
MDN1	-1.49	1.93E-07	1.56E-06	Under-expressed
CYP4F11	-1.49	1.99E-05	8.55E-05	Under-expressed
KDR	-1.5	9.28E-15	7.77E-13	Under-expressed
ARRDC4	-1.5	4.48E-10	8.77E-09	Under-expressed
ECM2	-1.5	1.46E-09	2.35E-08	Under-expressed
PPP4R2	-1.5	7.62E-09	9.76E-08	Under-expressed
ASXL2	-1.5	2.29E-08	2.52E-07	Under-expressed
ZBTB16	-1.5	4.04E-06	2.14E-05	Under-expressed
RAD54L2	-1.51	1.82E-11	5.70E-10	Under-expressed
CMBL	-1.51	2.31E-07	1.82E-06	Under-expressed
ADRA1B	-1.51	2.40E-07	1.88E-06	Under-expressed
REPS2	-1.52	1.54E-09	2.44E-08	Under-expressed
STRN	-1.52	1.50E-08	1.75E-07	Under-expressed
KLF9	-1.53	1.01E-11	3.45E-10	Under-expressed
PALM2	-1.53	1.70E-08	1.94E-07	Under-expressed
RSPRY1	-1.53	1.83E-07	1.50E-06	Under-expressed
ACSS3	-1.53	1.48E-04	4.93E-04	Under-expressed
F8	-1.54	5.70E-15	5.01E-13	Under-expressed
ENPEP	-1.54	2.29E-11	7.07E-10	Under-expressed
SLC16A2	-1.54	1.16E-09	1.94E-08	Under-expressed
GNA11	-1.54	1.62E-08	1.87E-07	Under-expressed
MFHAS1	-1.54	4.00E-07	2.91E-06	Under-expressed
FBP1	-1.54	1.05E-05	4.89E-05	Under-expressed
ARG1	-1.54	1.45E-03	3.58E-03	Under-expressed
PPM1L	-1.55	4.02E-12	1.52E-10	Under-expressed
SCP2	-1.56	3.72E-18	8.15E-16	Under-expressed
TMEM47	-1.56	6.15E-11	1.63E-09	Under-expressed
DIO1	-1.56	2.16E-04	6.87E-04	Under-expressed
APOC3	-1.56	2.79E-03	6.40E-03	Under-expressed
HFE	-1.57	2.69E-12	1.10E-10	Under-expressed
SPARCL1	-1.57	1.65E-07	1.38E-06	Under-expressed
ADRB2	-1.57	4.34E-06	2.27E-05	Under-expressed
RORC	-1.58	1.27E-05	5.78E-05	Under-expressed
GNMT	-1.58	7.51E-04	2.03E-03	Under-expressed
MASP1	-1.59	3.59E-08	3.73E-07	Under-expressed
C6	-1.59	6.07E-05	2.26E-04	Under-expressed
ELFN1	-1.6	1.14E-06	7.12E-06	Under-expressed
BMERB1	-1.61	8.70E-15	7.33E-13	Under-expressed
PSD3	-1.61	1.18E-12	5.42E-11	Under-expressed
RNF168	-1.61	1.73E-10	3.88E-09	Under-expressed
CES1	-1.61	8.19E-05	2.94E-04	Under-expressed
PAH	-1.61	8.24E-05	2.96E-04	Under-expressed
RNF125	-1.62	1.91E-12	8.32E-11	Under-expressed
KBTBD7	-1.62	2.91E-11	8.66E-10	Under-expressed

SEC14L2	-1.62	3.27E-10	6.68E-09	Under-expressed
RAPGEF4	-1.63	3.16E-13	1.76E-11	Under-expressed
FMO4	-1.63	2.70E-10	5.70E-09	Under-expressed
HIP1	-1.63	5.01E-10	9.58E-09	Under-expressed
MYLK	-1.64	7.21E-15	6.20E-13	Under-expressed
SLC2A10	-1.64	8.39E-10	1.48E-08	Under-expressed
ABCC11	-1.64	2.55E-09	3.81E-08	Under-expressed
AOX1	-1.64	2.27E-05	9.57E-05	Under-expressed
PHYHD1	-1.64	4.40E-05	1.71E-04	Under-expressed
SLC28A1	-1.64	4.65E-05	1.79E-04	Under-expressed
UGT2B4	-1.67	5.69E-05	2.14E-04	Under-expressed
PRKAR2A	-1.68	6.12E-10	1.13E-08	Under-expressed
MAP3K5	-1.69	6.37E-12	2.32E-10	Under-expressed
CCDC170	-1.69	2.43E-08	2.66E-07	Under-expressed
ALPL	-1.69	1.85E-05	7.99E-05	Under-expressed
APBA1	-1.71	8.19E-10	1.45E-08	Under-expressed
C4BPA	-1.71	1.38E-05	6.21E-05	Under-expressed
CFHR2	-1.71	1.07E-03	2.75E-03	Under-expressed
DENND2C	-1.72	1.97E-13	1.21E-11	Under-expressed
GATM	-1.72	7.72E-11	1.99E-09	Under-expressed
HMGN5	-1.72	1.87E-08	2.12E-07	Under-expressed
SLC41A2	-1.74	4.01E-12	1.52E-10	Under-expressed
SLCO2A1	-1.74	5.55E-07	3.87E-06	Under-expressed
CYP1B1	-1.75	5.85E-09	7.77E-08	Under-expressed
HJV	-1.75	8.12E-04	2.17E-03	Under-expressed
8-Mar	-1.76	3.76E-10	7.52E-09	Under-expressed
MPDZ	-1.77	1.11E-17	1.96E-15	Under-expressed
BHMT2	-1.77	3.09E-06	1.68E-05	Under-expressed
CDO1	-1.77	1.29E-04	4.40E-04	Under-expressed
SHE	-1.8	7.05E-15	6.12E-13	Under-expressed
CYP4F3	-1.8	5.83E-06	2.93E-05	Under-expressed
IL6R	-1.81	1.54E-11	4.97E-10	Under-expressed
RALGAPA2	-1.82	2.91E-10	6.01E-09	Under-expressed
IGF1	-1.82	3.56E-09	5.09E-08	Under-expressed
ALDH8A1	-1.82	5.21E-06	2.65E-05	Under-expressed
APOA5	-1.84	1.50E-04	5.00E-04	Under-expressed
RASGEF1B	-1.85	7.93E-12	2.76E-10	Under-expressed
TEK	-1.87	3.29E-10	6.71E-09	Under-expressed
SPATA13	-1.87	2.81E-08	3.01E-07	Under-expressed
PCK1	-1.87	2.00E-04	6.46E-04	Under-expressed
SDS	-1.87	3.22E-04	9.73E-04	Under-expressed
PON1	-1.88	3.72E-05	1.48E-04	Under-expressed
ACSL1	-1.89	1.59E-12	7.14E-11	Under-expressed
EHHADH	-1.89	3.85E-12	1.48E-10	Under-expressed
MICU3	-1.91	1.02E-10	2.51E-09	Under-expressed
ERN1	-1.92	5.29E-11	1.46E-09	Under-expressed
HMGCS2	-1.92	1.43E-05	6.40E-05	Under-expressed
ZKSCAN8	-1.93	5.34E-07	3.73E-06	Under-expressed
ALDH6A1	-1.94	2.41E-13	1.43E-11	Under-expressed
AUTS2	-1.94	1.41E-11	4.58E-10	Under-expressed
ANO1	-1.94	1.08E-09	1.83E-08	Under-expressed
ABCB4	-1.95	5.62E-08	5.43E-07	Under-expressed
UGT2B15	-1.95	1.55E-05	6.85E-05	Under-expressed
GPD1	-1.96	1.27E-07	1.10E-06	Under-expressed
ACSM2A	-1.96	1.02E-05	4.78E-05	Under-expressed
ABAT	-1.97	1.03E-12	4.81E-11	Under-expressed
PDK4	-1.98	8.01E-11	2.05E-09	Under-expressed

CTH	-1.98	1.82E-09	2.83E-08	Under-expressed
ZNF281	-1.99	2.33E-10	5.02E-09	Under-expressed
ALDOB	-1.99	7.88E-05	2.85E-04	Under-expressed
MYRIP	-2	1.28E-05	5.81E-05	Under-expressed
APOC4	-2	2.67E-04	8.24E-04	Under-expressed
G6PC	-2.02	8.12E-06	3.91E-05	Under-expressed
CPS1	-2.03	4.78E-04	1.37E-03	Under-expressed
DPYS	-2.04	7.36E-06	3.59E-05	Under-expressed
SLC46A3	-2.05	1.11E-13	7.20E-12	Under-expressed
SERPINC1	-2.05	1.05E-04	3.65E-04	Under-expressed
RETREG1	-2.08	9.87E-07	6.31E-06	Under-expressed
LIPG	-2.09	1.11E-10	2.68E-09	Under-expressed
ADAMTSL3	-2.09	3.59E-08	3.73E-07	Under-expressed
SLC38A4	-2.1	1.36E-06	8.26E-06	Under-expressed
NCOA2	-2.11	1.04E-09	1.77E-08	Under-expressed
IRF6	-2.11	6.88E-08	6.47E-07	Under-expressed
GHR	-2.12	1.72E-11	5.45E-10	Under-expressed
MFAP3L	-2.12	1.48E-10	3.40E-09	Under-expressed
RDH16	-2.12	2.02E-06	1.17E-05	Under-expressed
USP12	-2.15	1.07E-11	3.62E-10	Under-expressed
UHMK1	-2.17	5.85E-13	2.87E-11	Under-expressed
FNIP2	-2.19	4.91E-13	2.52E-11	Under-expressed
NFIC	-2.2	1.57E-12	7.08E-11	Under-expressed
DMGDH	-2.24	3.20E-09	4.64E-08	Under-expressed
HACD2	-2.24	2.31E-08	2.54E-07	Under-expressed
HIPK3	-2.27	1.93E-09	2.98E-08	Under-expressed
BHMT	-2.27	3.54E-06	1.89E-05	Under-expressed
GOLIM4	-2.28	2.36E-10	5.07E-09	Under-expressed
PPTC7	-2.28	2.13E-09	3.26E-08	Under-expressed
AQP9	-2.28	3.92E-06	2.08E-05	Under-expressed
FRRS1	-2.31	5.65E-10	1.06E-08	Under-expressed
UPB1	-2.31	2.03E-06	1.17E-05	Under-expressed
ABCC9	-2.34	7.44E-16	7.56E-14	Under-expressed
ADH4	-2.36	2.95E-04	9.02E-04	Under-expressed
CPED1	-2.4	3.04E-14	2.31E-12	Under-expressed
TDO2	-2.4	7.88E-08	7.27E-07	Under-expressed
FMO3	-2.43	5.62E-08	5.43E-07	Under-expressed
HRG	-2.45	1.24E-05	5.65E-05	Under-expressed
LINC01554	-2.49	6.44E-06	3.20E-05	Under-expressed
ACSM5	-2.51	1.31E-10	3.09E-09	Under-expressed
ABCA6	-2.52	2.30E-09	3.48E-08	Under-expressed
HSD17B6	-2.52	9.09E-09	1.13E-07	Under-expressed
SLC1A1	-2.52	7.00E-08	6.57E-07	Under-expressed
FNDC5	-2.56	6.24E-09	8.22E-08	Under-expressed
SLC6A1	-2.57	2.33E-12	9.72E-11	Under-expressed
IL6ST	-2.6	5.63E-11	1.53E-09	Under-expressed
CHI3L1	-2.61	2.01E-06	1.16E-05	Under-expressed
ADH1B	-2.63	4.05E-07	2.94E-06	Under-expressed
ADH1C	-2.86	3.60E-06	1.93E-05	Under-expressed
GPLD1	-2.97	9.48E-13	4.45E-11	Under-expressed
CYP2C8	-3.06	1.04E-09	1.77E-08	Under-expressed
GFRA1	-3.11	6.76E-09	8.80E-08	Under-expressed
CYP8B1	-3.27	1.91E-08	2.16E-07	Under-expressed
SLC22A1	-3.54	7.44E-11	1.92E-09	Under-expressed
HSD11B1	-3.61	4.34E-11	1.22E-09	Under-expressed
TAT	-3.75	7.42E-12	2.62E-10	Under-expressed

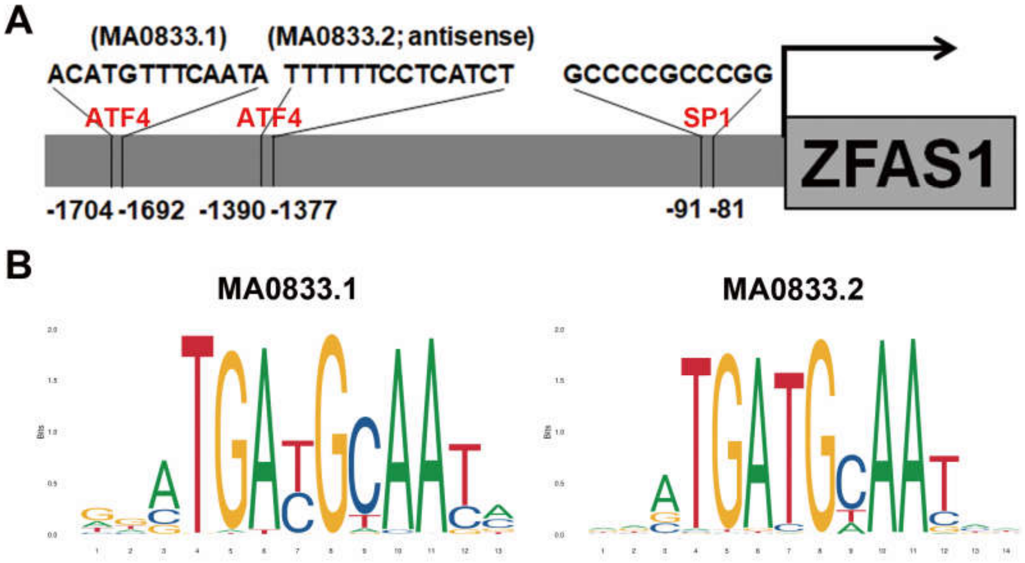

Figure S1. The predicted ATF4-binding sites on ZFAS1 promoter. **(A)** The potential ATF4-binding sites on ZFAS1 promoter at the region -1 to -2000 were predicted using the JASPAR database (http://jaspar.genereg.net/). Default setting was used. A previously identified SP1-binding site on ZFAS1 promoter was also shown. **(B)** The sequence logos for two ATF4- binding sites used for prediction.
